# Supplementary material for: Emergence and Spread of SARS-CoV-2 Lineages B.1.1.7 and P.1 in Italy
Source: Viruses. 2021 Apr 29;13(5):794. doi: 10.3390/v13050794 (PMC8146936; doi:10.3390/v13050794)
Supplement: Supplementary file 1 [file viruses-13-00794-s001.zip › viruses-1196890-suppl/Supplementary Figures.pdf]

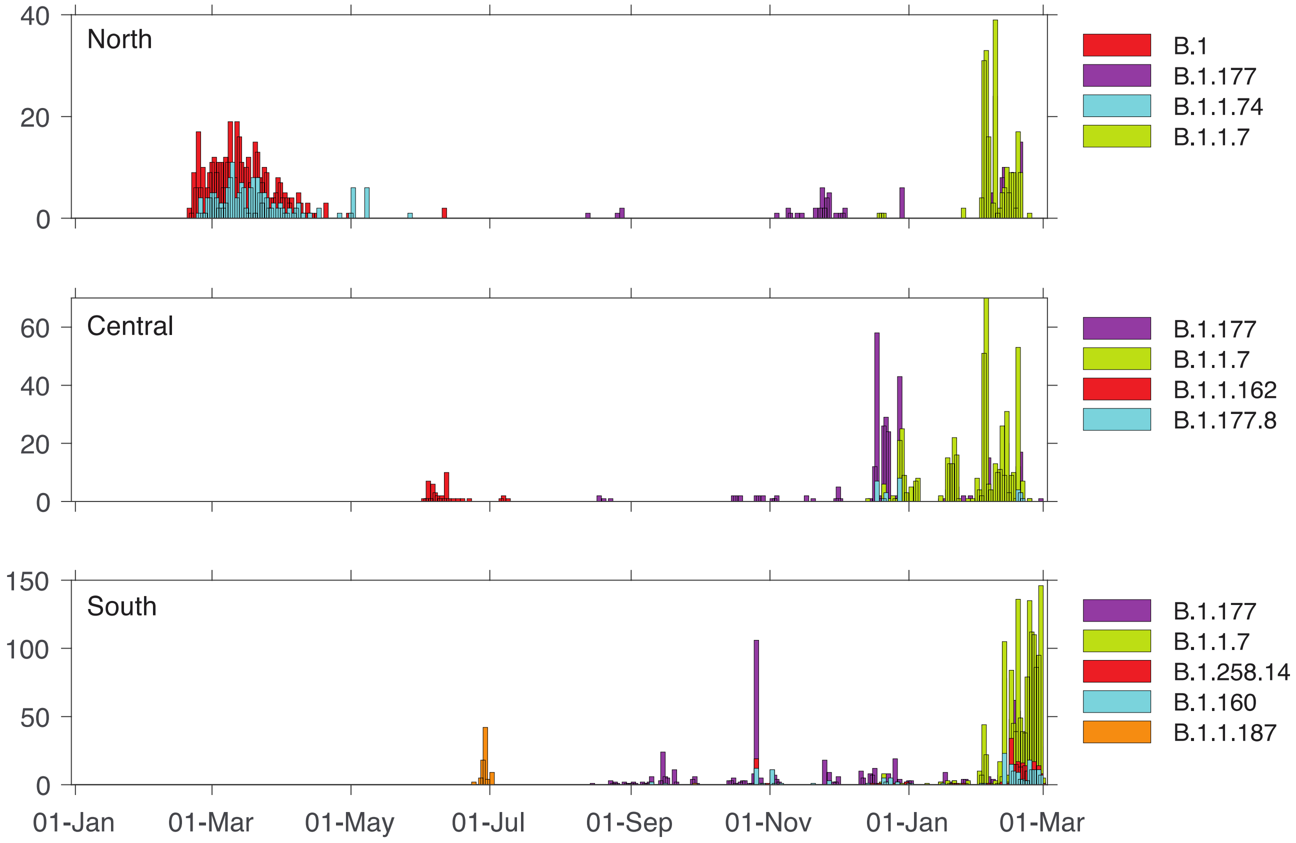

Supplementary Figure S1. Changing frequency of SARS-CoV-2 lineages sampled over time in Italy. Bars show the Number of cases reported between 01 January 2020 – 1 March 2021 in North (top), Central (middle), and South Italy (bottom).

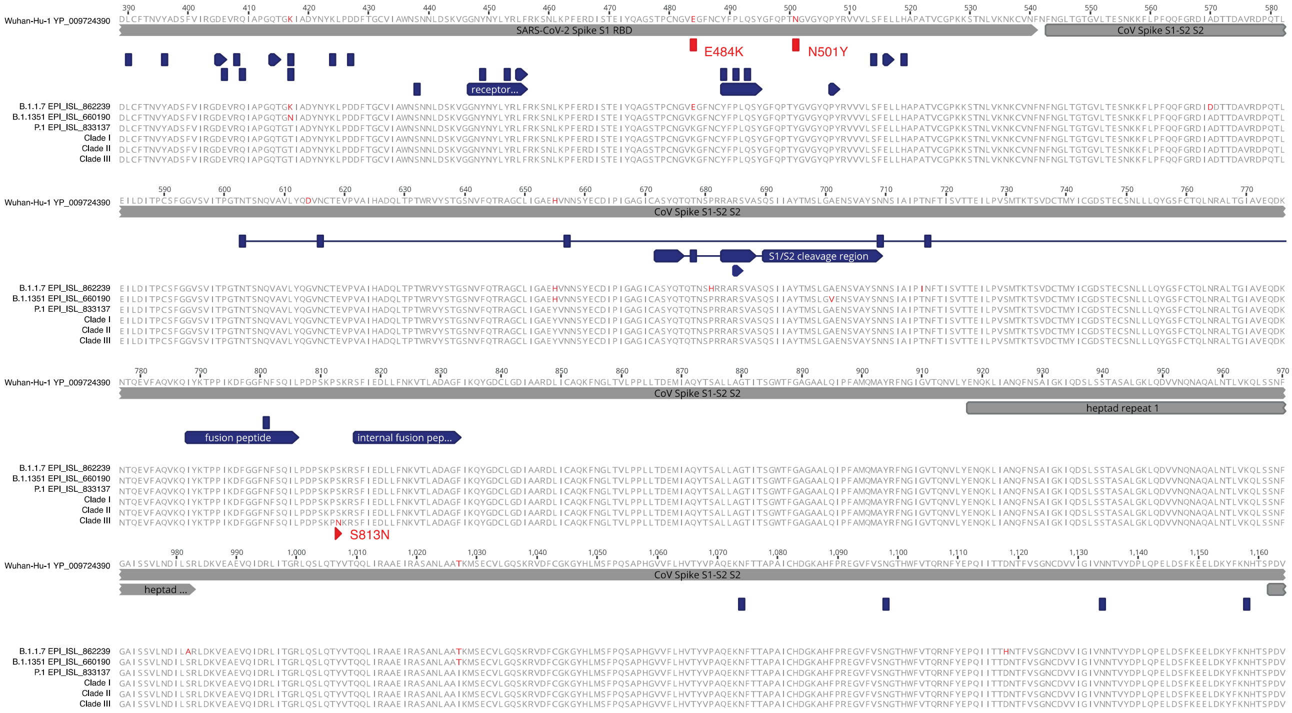

Supplementary Figure S2. Amino acid alignment of the SARS-CoV-2 spike protein. Amino acid alignment of the partial spike protein sequence is shown for reference sequences of different SARS-CoV-2 lineage variants as well as the consensus sequence of clade I – III of P.1 (Fig. 3). Functional features are annotated and amino acid differences are marked in red. The E484K, N501Y and S813N amino acid substitutions are indicated.

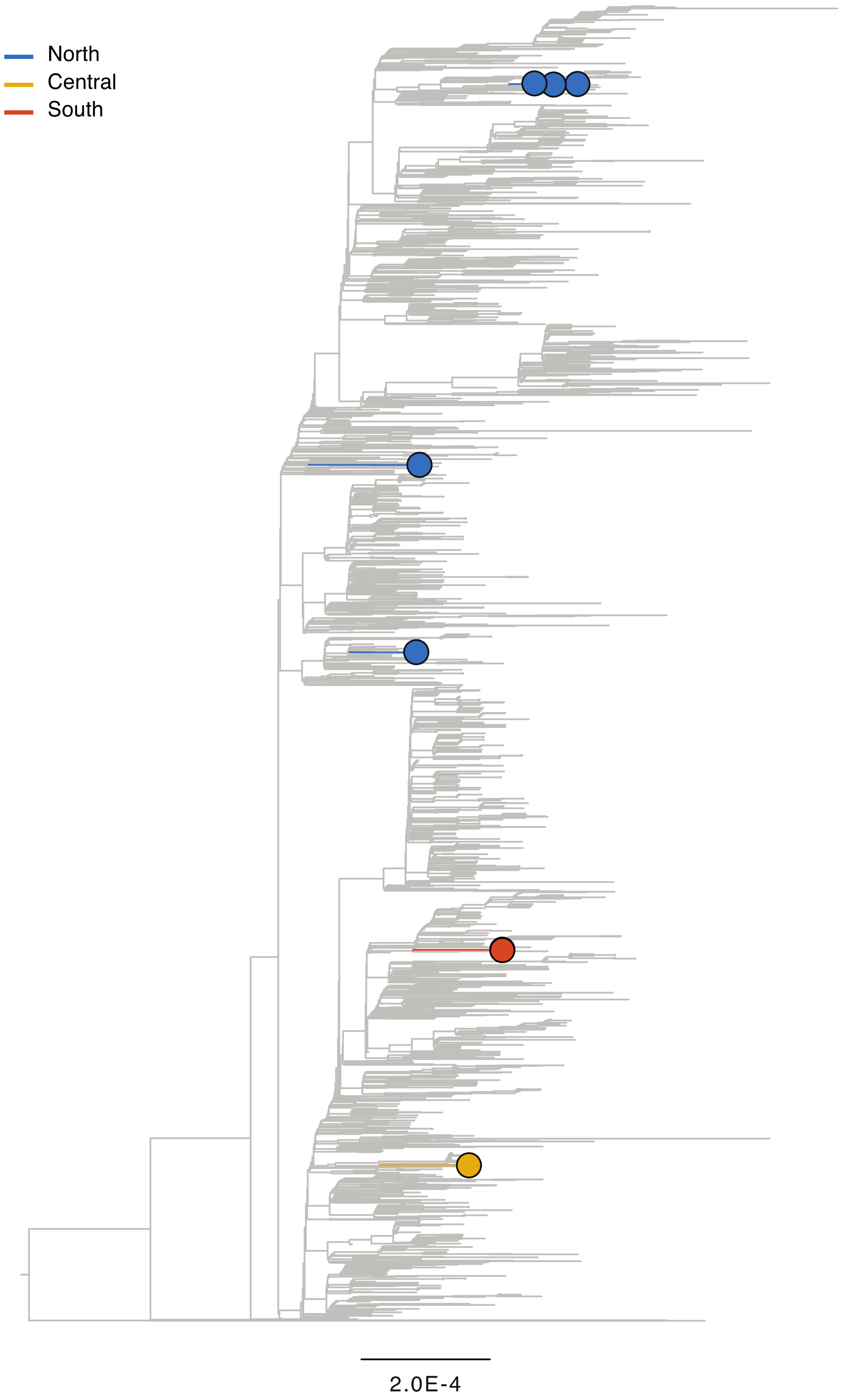

Supplementary Figure S3. Genomic epidemiology of global SARS-CoV-2 lineage B.1.351. Maximum likelihood phylogeny of SARS-CoV-2 full genome sequences (n=2,309 global, n=8 Italian). Branch lengths show the number of nucleotide substitutions per site and branches are coloured according to geographic region of sampling: global = grey, blue = North Italy, yellow = Central Italy, red = South Italy. Global data is shown in grey.
