# Supplementary material for: Emergence and Spread of SARS-CoV-2 Lineages B.1.1.7 and P.1 in Italy
Source: Viruses. 2021 Apr 29;13(5):794. doi: 10.3390/v13050794 (PMC8146936; doi:10.3390/v13050794)
Supplement: Supplementary file 1 [file viruses-13-00794-s001.zip › viruses-1196890-suppl/Supplementary TableS1.pdf]

We gratefully acknowledge the following Authors from the Originating laboratories responsible for obtaining the specimens, as well as the Submitting laboratories where the genome data were generated and shared via GISAID, on which this research is based.

All Submitters of data may be contacted directly via www.gisaid.org

Authors are sorted alphabetically.

Accession ID	Originating Laboratory	Submitting Laboratory	Authors
EPI_ISL_1007642	University of Bari Biomedical Sciences and Human Oncology	University of Bari Biomedical Sciences and Human Oncology	Chironna M., Sallustio A., Loconsole D., Accogli M.
EPI_ISL_1008663	Istituto Zooprofilattico Sperimentale della Puglia e della Basilicata	Istituto Zooprofilattico Sperimentale della Puglia e della Basilicata	Parisi A., Bianco A., Capozzi L., Del Sambro L., Simone D., Chironna M., Loconsole D., Sallustio A., Giannico A.
EPI_ISL_1008664	Ospedale Santa Caterina Novella	Istituto Zooprofilattico Sperimentale della Puglia e della Basilicata	Parisi A., Bianco A., Capozzi L., Del Sambro L., Simone D., Chironna M., Loconsole D., Sallustio A., Ridolfi D.
EPI_ISL_1008665, EPI_ISL_1008666, EPI_ISL_1008667, EPI_ISL_1008668	Istituto Zooprofilattico Sperimentale della Puglia e della Basilicata	Istituto Zooprofilattico Sperimentale della Puglia e della Basilicata	Parisi A., Bianco A., Capozzi L., Del Sambro L., Simone D., Chironna M., Loconsole D., Sallustio A., Ridolfi D.
EPI_ISL_1008669, EPI_ISL_1008670, EPI_ISL_1008671, EPI_ISL_1008672, EPI_ISL_1008673, EPI_ISL_1008674, EPI_ISL_1008675, EPI_ISL_1008676, EPI_ISL_1008677	Istituto Zooprofilattico Sperimentale della Puglia e della Basilicata	Istituto Zooprofilattico Sperimentale della Puglia e della Basilicata	Parisi A., Bianco A., Capozzi L., Del Sambro L., Simone D., Chironna M., Loconsole D., Sallustio A., Giannico A.
EPI_ISL_1008678	Presidio di Brindisi Di Summa - Perrino	Istituto Zooprofilattico Sperimentale della Puglia e della Basilicata	Parisi A., Bianco A., Capozzi L., Del Sambro L., Simone D., Chironna M., Loconsole D., Sallustio A., Ridolfi D.
EPI_ISL_1008679, EPI_ISL_1008680, EPI_ISL_1008681, EPI_ISL_1008682, EPI_ISL_1008683, EPI_ISL_1008684, EPI_ISL_1008685, EPI_ISL_1008686, EPI_ISL_1008687	Istituto Zooprofilattico Sperimentale della Puglia e della Basilicata	Istituto Zooprofilattico Sperimentale della Puglia e della Basilicata	Parisi A., Bianco A., Capozzi L., Del Sambro L., Simone D., Chironna M., Loconsole D., Sallustio A., Ridolfi D.
EPI_ISL_1008688, EPI_ISL_1008689, EPI_ISL_1008690, EPI_ISL_1008691, EPI_ISL_1008692, EPI_ISL_1008693, EPI_ISL_1008694, EPI_ISL_1008695, EPI_ISL_1008696, EPI_ISL_1008697, EPI_ISL_1008698, EPI_ISL_1008699			
see above	Istituto Zooprofilattico Sperimentale della Puglia e della Basilicata	Istituto Zooprofilattico Sperimentale della Puglia e della Basilicata	Parisi A., Bianco A., Capozzi L., Del Sambro L., Simone D., Chironna M., Loconsole D., Sallustio A., Giannico A.
EPI_ISL_1008700	Ospedale Santa Caterina Novella	Istituto Zooprofilattico Sperimentale della Puglia e della Basilicata	Parisi A., Bianco A., Capozzi L., Del Sambro L., Simone D., Chironna M., Loconsole D., Sallustio A., Ridolfi D.
EPI_ISL_1008701, EPI_ISL_1008702	Istituto Zooprofilattico Sperimentale della Puglia e della Basilicata	Istituto Zooprofilattico Sperimentale della Puglia e della Basilicata	Parisi A., Bianco A., Capozzi L., Del Sambro L., Simone D., Chironna M., Loconsole D., Sallustio A., Giannico A.
EPI_ISL_1008703, EPI_ISL_1008704, EPI_ISL_1008705	Presidio di Brindisi Di Summa - Perrino	Istituto Zooprofilattico Sperimentale della Puglia e della Basilicata	Parisi A., Bianco A., Capozzi L., Del Sambro L., Simone D., Chironna M., Loconsole D., Sallustio A., Ridolfi D.
EPI_ISL_1008706	Ospedale Santissima Annunziata	Istituto Zooprofilattico Sperimentale della Puglia e della Basilicata	Parisi A., Bianco A., Capozzi L., Del Sambro L., Simone D., Chironna M., Loconsole D., Sallustio A., Ridolfi D.
EPI_ISL_1008707, EPI_ISL_1008708, EPI_ISL_1008709, EPI_ISL_1008710, EPI_ISL_1008711, EPI_ISL_1008712	Presidio di Brindisi Di Summa - Perrino	Istituto Zooprofilattico Sperimentale della Puglia e della Basilicata	Parisi A., Bianco A., Capozzi L., Del Sambro L., Simone D., Chironna M., Loconsole D., Sallustio A., Ridolfi D.
EPI_ISL_1009018	Istituto Zooprofilattico Sperimentale Lazio e Toscana "M. Aleandri"	INMI Lazzaro Spallanzani IRCCS	CEM Gruber, B Bartolini, E Giombini, M Rueca, O Butera, F Messina, MT Scicluna, G Manna, A Cersini, A Di Caro, MR Capobianchi
EPI_ISL_1009024	Fondazione Policlinico Universitario "A. Gemelli" IRCCS	INMI Lazzaro Spallanzani IRCCS	E Giombini, M. Rueca, B Bartolini, O Butera, C.E.M Gruber, F Messina, P Cattani,M Sanguinetti, A Di Caro, MR Capobianchi
EPI_ISL_1009025	Fondazione Policlinico Universitario "A. Gemelli" IRCCS	INMI Lazzaro Spallanzani IRCCS	B Bartolini, O Butera, C.E.M Gruber, M Rueca, F Messina, E Giombini, P Cattani,M Sanguinetti, MR Capobianchi, A Di Caro
EPI_ISL_1009026	Fondazione Policlinico Universitario "A. Gemelli" IRCCS	INMI Lazzaro Spallanzani IRCCS	F Messina, C.E.M Gruber, B Bartolini, E Giombini, M Rueca, O Butera, P Cattani,M Sanguinetti, A Di Caro, MR Capobianchi
EPI_ISL_1009027	Fondazione Policlinico Universitario "A. Gemelli" IRCCS	INMI Lazzaro Spallanzani IRCCS	M Rueca, O Butera, F Messina, CEM Gruber, B Bartolini, E Giombini, P Cattani,M Sanguinetti, A Di Caro, MR Capobianchi
EPI_ISL_1009028	AOU Policlinico Umberto I; Sapienza Università di Roma	INMI Lazzaro Spallanzani IRCCS	CEM Gruber, B Bartolini, E Giombini, M Rueca, O Butera, F Messina, G Antonelli, O Turriziani, A Di Caro, MR Capobianchi
EPI_ISL_1009029	AOU Policlinico Umberto I; Sapienza Università di Roma	INMI Lazzaro Spallanzani IRCCS	B Bartolini, O Butera, C.E.M Gruber, M Rueca, F Messina, E Giombini, G Antonelli, O Turriziani, MR Capobianchi, A Di Caro
EPI_ISL_1009030	AOU Policlinico Umberto I; Sapienza Università di Roma	INMI Lazzaro Spallanzani IRCCS	M Rueca, O Butera, F Messina, CEM Gruber, B Bartolini, E Giombini, G Antonelli, O Turriziani, A Di Caro, MR Capobianchi
EPI_ISL_1009031	AOU Policlinico Umberto I; Sapienza Università di Roma	INMI Lazzaro Spallanzani IRCCS	E Giombini, M. Rueca, B Bartolini, O Butera, C.E.M Gruber, F Messina, G Antonelli, O Turriziani, MR Capobianchi, A Di Caro
EPI_ISL_1012923, EPI_ISL_1012924	Department of Infectious Diseases, Istituto Superiore di Sanità, Rome, Italy; ASST Sette Laghi, Varese, Italy	Istituto Superiore di Sanità (ISS)	Paola Stefanelli, Angela Di Martino, Alessandra Lo Presti, Stefano Fiore, Fabrizio Maggi, Federica Novazzi, Andreina Baj, Angelo Genoni, Manuela Marra, Maria Carollo, Marco Crescenzi
EPI_ISL_1013095	Azienda Sanitaria dell'Alto Adige Laboratorio Aziendale di Microbiologia e Virologia	Istituto di Genomica Applicata	Elisabetta Pagani, Irene Bianconi, Elisabetta Giacobazzi, Elisa Masi, Stefanie Wieser, Irena Jurman, Vera Vendramin, Eleonora Paparelli, Davide Scaglione, Michele Morgante
EPI_ISL_1013531, EPI_ISL_1013532, EPI_ISL_1013557, EPI_ISL_1013558, EPI_ISL_1013559, EPI_ISL_1013560, EPI_ISL_1013561, EPI_ISL_1013562, EPI_ISL_1013563, EPI_ISL_1013564, EPI_ISL_1013565			
see above	1.AO Universitaria 'S. Giovanni di Dio e Ruggi D'Aragona, Scuola Medica Salernitana' Hospital / 2.UOC di Virologia e Microbiologia, Università della Campania 'L. Vanvitelli' / 3.AO Universitaria 'Federico II' Napoli Hospital / 4.AORN 'San Giuseppe Moscati' Avellino Hospital / 5.AO 'San Pio - presidio G. Rummo' Benevento Hospital / 6.AO 'Sant'Anna e San Sebastiano' Caserta Hospital / 7.PO 'Maria Santissima Addolorata' Eboli Hospital / 8.Biogem Istituto di Ricerche Genetiche	1. Genome Research Center for Health (CRGS) / 2. Laboratory of Molecular Medicine and Genomics(LMMGe) / 3. Center for Research in Pure and Applied Mathematics (CRMPA)	Giorgio Giurato, Francesca Rizzo, Alessandro Weisz, Gianluigi Franci, Giovanni Nassa, Pasquale Pagliano, Roberta Tarallo, Elena Alexandrova, Ylenia D'Agostino, Carlo Ferravante, Jessica Lamberti, Viola Melone, Domenico Memoli, Valeria Mirici Cappa, Domenico Palumbo, Giovanni Pecoraro, Assunta Sellitto, Oriana Strianese, Ilaria Terenzi, Giuseppe Fenza, Aniello Gentile, Antonello Saccomanno, Sonia Amabile, Teresa Rocco, Annamaria Salvati, Emilia Vaccaro, Massimiliano Galdiero, Michele Cennamo, Giuseppe Portella, Maria Grazia Foti, Mariarosaria Ingino, Maria Landi, Maurizio Fumi, Vincenzo Rocco, Rita Greco, Vittoria Letizia, Arnolfo Petruzziello, Maddalena Schioppa, Gregorio Goffredi, Francesca Marciano, Michele Caraglia, Alessia Cossu, Marianna Scrima, Edmondo Adorisio, Morena D'Avenia, Michela Iacobellis, Rosanna Piluscio, Giorgio Dirani, Vittorio Sambri, Simona Semprini, Silvia Zanoli, Francesco Curcio, Stefania Marzinotto, Andreina Baj, Fausto Sessa.
EPI_ISL_1014237	University of Bari Biomedical Sciences and Human Oncology	University of Bari Biomedical Sciences and Human Oncology	Chironna M., Sallustio A., Loconsole D., Accogli M.
EPI_ISL_1014545	Department of Infectious Diseases, Istituto Superiore di Sanità, Rome, Italy; Università degli Studi di Perugia, Perugia, Italy	Istituto Superiore di Sanità (ISS)	Paola Stefanelli, Alessandra Lo Presti, Angela Di Martino, Stefano Fiore, Antonella Mencacci, Barbara Camilloni, Manuela Marra, Maria Carollo, Marco Crescenzi
EPI_ISL_1014675	Department of Infectious Diseases, Istituto Superiore di Sanità,	Istituto Superiore di Sanità (ISS)	Paola Stefanelli, Alessandra Lo Presti, Angela Di Martino, Stefano Fiore, Antonella Mencacci, Barbara Camilloni, Manuela Marra, Maria Carollo, Marco

	Rome, Italy; Università degli Studi di Perugia, Perugia, Italy		Crescenzi, Luca De Sabato
EPI_ISL_1014690	University of Bari Biomedical Sciences and Human Oncology	University of Bari Biomedical Sciences and Human Oncology	Chironna M., Sallustio A., Loconsole D., Accogli M.
EPI_ISL_1015515, EPI_ISL_1015516, EPI_ISL_1015517, EPI_ISL_1015518, EPI_ISL_1015519, EPI_ISL_1015520, EPI_ISL_1015521, EPI_ISL_1015532, EPI_ISL_1015533, EPI_ISL_1015534, EPI_ISL_1015535, EPI_ISL_1015536, EPI_ISL_1015537, EPI_ISL_1015538, EPI_ISL_1015539, EPI_ISL_1015540, EPI_ISL_1015541, EPI_ISL_1015542, EPI_ISL_1015543, EPI_ISL_1015544, EPI_ISL_1015545, EPI_ISL_1015546, EPI_ISL_1015547, EPI_ISL_1015548, EPI_ISL_1015549, EPI_ISL_1015550, EPI_ISL_1015551, EPI_ISL_1015552, EPI_ISL_1015553, EPI_ISL_1015554, EPI_ISL_1015555, EPI_ISL_1015556, EPI_ISL_1015557, EPI_ISL_1015558, EPI_ISL_1015559, EPI_ISL_1015560			
see above	Azienda Sanitaria dell'Alto Adige Laboratorio Aziendale di Microbiologia e Virologia	Istituto di Genomica Applicata	Elisabetta Pagani, Irene Bianconi, Elisabetta Giacobazzi, Elisa Masi, Stefanie Wieser, Irena Jurman, Vera Vendramin, Gabriele Magris, Eleonora Paparelli, Davide Scaglione, Michele Morgante
EPI_ISL_1020310, EPI_ISL_1020314, EPI_ISL_1020329, EPI_ISL_1020331, EPI_ISL_1020333, EPI_ISL_1020334, EPI_ISL_1020335, EPI_ISL_1020336, EPI_ISL_1020337	Laboratorio di Riferimento Regionale della Sicilia Occidentale per l'Emergenza COVID-19	Laboratorio di Riferimento Regionale della Sicilia Occidentale per l'Emergenza COVID-19	Fabio Tramuto, Carmelo Massimo Maida, Daniela Di Naro, Giulia Randazzo, Walter Mazzucco, Giorgio Graziano, Vincenzo Restivo, Claudio Costantino, Francesco Vitale
EPI_ISL_1023524	Ospedale San Camillo De Lellis di Rieti	INMI Lazzaro Spallanzani IRCCS	Cesare E.M. Gruber, Barbara Bartolini, Emanuela Giombini, Francesco Messina, Martina Rueca, Ornella Butera, Stefano Venarubea, Luca Casertano, Luisa Marchioni, Antonino Di Caro, Maria R. Capobianchi
EPI_ISL_1034144	MEDICO COMPETENTE P.O. L'AQUILA	Istituto Zooprofilattico Sperimentale dell'Abruzzo e Molise "G. Caporale"	Lorusso A, Marcacci M, Di Domenico M, Ancora M, Curini V, Mangone I, Rinaldi A, Scialabba S, Di Pasquale A, Cammà C, Puglia I, Calistri P, Savini G
EPI_ISL_1034280	INMI Lazzaro Spallanzani IRCCS	INMI Lazzaro Spallanzani IRCCS	Cesare E.M. Gruber, Barbara Bartolini, Emanuela Giombini, Francesco Messina, Martina Rueca, Ornella Butera, Emanuele Nicastri, Alessandra D'Abramo, Antonino Di Caro, Maria R. Capobianchi
EPI_ISL_1034748, EPI_ISL_1034749, EPI_ISL_1034750, EPI_ISL_1034751, EPI_ISL_1034752, EPI_ISL_1034753	Center of Advanced Studies and Technology, Molecular Genetics Laboratory	Center of Advanced Studies and Technology, Molecular Genetics Laboratory	Ferrante Rossella, Mandatori Domitilla, De Fabritiis Simone, Damiani Verena, Anaclerio Federico
EPI_ISL_1034793	Virologia Dipartimento di Scienze Biomediche Università di Sassari Viale San Pietro, 43/B - Sassari	Laboratorio Specialistico di Ematologia, Ospedale "San Francesco", via Mannironi 1, 08100 Nuoro	Giovanna Piras, Tatiana Fancello, Maria Monne, Rosanna Asproni, Caterina Serra, Elena Rimini, Salvatore Rubino
EPI_ISL_1034916	Laboratorio Biologia Molecolare Sars Cov2 - UOC Laboratorio Analisi - Servizio Medicina di Laboratorio, Ospedale "San Francesco" - ATS-ASSL Nuoro	Laboratorio specialistico UOC Ematologia - Ospedale "San Francesco" - ATS-ASSL Nuoro	Piras Giovanna, Asproni Rosanna, Malune Paolo, Fiamma Maura, Monne Maria Itria, Palmas Angelo Domenico, Lo Maglio Iana, Mameli Giuseppe
EPI_ISL_1035871	Department of Infectious Diseases, Istituto Superiore di Sanità, Rome, Italy; Università degli Studi di Siena, Siena, Italy	Istituto Superiore di Sanità (ISS)	Paola Stefanelli, Angela Di Martino, Alessandra Lo Presti, Stefano Fiore, Maria Grazia Cusi, Gabriele Anichini, Claudia Gandolfo, Gianni Gori Savellini, Manuela Marra, Maria Carollo, Marco Crescenzi
EPI_ISL_1035873, EPI_ISL_1035874, EPI_ISL_1035875, EPI_ISL_1035876, EPI_ISL_1035877, EPI_ISL_1035878, EPI_ISL_1035879, EPI_ISL_1035880, EPI_ISL_1035881, EPI_ISL_1035882, EPI_ISL_1035883, EPI_ISL_1035884, EPI_ISL_1035885, EPI_ISL_1035886, EPI_ISL_1035887, EPI_ISL_1035888			
see above	Istituto Zooprofilattico Sperimentale del Mezzogiorno	IZSM-U.O.C. Virologia	Maurizio Viscardi, Lorena Cardillo, Pellegrino Cerino, Massimo Zollo,Giovanna Fusco, Esterina De Carlo, Antonio Limone
EPI_ISL_1035893, EPI_ISL_1035894, EPI_ISL_1035895, EPI_ISL_1035896, EPI_ISL_1035897, EPI_ISL_1035898, EPI_ISL_1035899	Istituto Zooprofilattico Sperimentale del Mezzogiorno	IZSM-U.O.C. Virologia	Maurizio Viscardi, Lorena Cardillo, Sergio Brandi, Pellegrino Cerino, Massimo Zollo,Giovanna Fusco, Esterina De Carlo, Antonio Limone
EPI_ISL_1035900, EPI_ISL_1035901, EPI_ISL_1035966	Department of Infectious Diseases, Istituto Superiore di Sanità, Rome, Italy; Università degli Studi di Siena, Siena, Italy	Istituto Superiore di Sanità (ISS)	Paola Stefanelli, Angela Di Martino, Alessandra Lo Presti, Stefano Fiore, Maria Grazia Cusi, Gabriele Anichini, Claudia Gandolfo, Gianni Gori Savellini, Manuela Marra, Maria Carollo, Marco Crescenzi
EPI_ISL_1036140	SIESP TERAMO	Istituto Zooprofilattico Sperimentale dell'Abruzzo e Molise "G. Caporale"	Lorusso A, Marcacci M, Di Domenico M, Ancora M, Curini V, Mangone I, Rinaldi A, Scialabba S, Di Pasquale A, Cammà C, Puglia I, Calistri P, Savini G
EPI_ISL_1036141	SIESP SULM	Istituto Zooprofilattico Sperimentale dell'Abruzzo e Molise "G. Caporale"	Lorusso A, Marcacci M, Di Domenico M, Ancora M, Curini V, Mangone I, Rinaldi A, Scialabba S, Di Pasquale A, Cammà C, Puglia I, Calistri P, Savini G
EPI_ISL_1036142	OSP CIV ATRI MEDICINA INT	Istituto Zooprofilattico Sperimentale dell'Abruzzo e Molise "G. Caporale"	Lorusso A, Marcacci M, Di Domenico M, Ancora M, Curini V, Mangone I, Rinaldi A, Scialabba S, Di Pasquale A, Cammà C, Puglia I, Calistri P, Savini G
EPI_ISL_1036143	OSP SAN SALVATORE MED INT	Istituto Zooprofilattico Sperimentale dell'Abruzzo e Molise "G. Caporale"	Lorusso A, Marcacci M, Di Domenico M, Ancora M, Curini V, Mangone I, Rinaldi A, Scialabba S, Di Pasquale A, Cammà C, Puglia I, Calistri P, Savini G
EPI_ISL_1036144, EPI_ISL_1036145	SIESP SULM	Istituto Zooprofilattico Sperimentale dell'Abruzzo e Molise "G. Caporale"	Lorusso A, Marcacci M, Di Domenico M, Ancora M, Curini V, Mangone I, Rinaldi A, Scialabba S, Di Pasquale A, Cammà C, Puglia I, Calistri P, Savini G
EPI_ISL_1036146	SIESP CH	Istituto Zooprofilattico Sperimentale dell'Abruzzo e Molise "G. Caporale"	Lorusso A, Marcacci M, Di Domenico M, Ancora M, Curini V, Mangone I, Rinaldi A, Scialabba S, Di Pasquale A, Cammà C, Puglia I, Calistri P, Savini G
EPI_ISL_1036147	SIESP DIP PREV CHIETI	Istituto Zooprofilattico Sperimentale dell'Abruzzo e Molise "G. Caporale"	Lorusso A, Marcacci M, Di Domenico M, Ancora M, Curini V, Mangone I, Rinaldi A, Scialabba S, Di Pasquale A, Cammà C, Puglia I, Calistri P, Savini G
EPI_ISL_1036148	USCA AVEZZANO	Istituto Zooprofilattico Sperimentale dell'Abruzzo e Molise "G. Caporale"	Lorusso A, Marcacci M, Di Domenico M, Ancora M, Curini V, Mangone I, Rinaldi A, Scialabba S, Di Pasquale A, Cammà C, Puglia I, Calistri P, Savini G
EPI_ISL_1036149	OSP SAN SALVATORE MED INT	Istituto Zooprofilattico Sperimentale dell'Abruzzo e Molise "G. Caporale"	Lorusso A, Marcacci M, Di Domenico M, Ancora M, Curini V, Mangone I, Rinaldi A, Scialabba S, Di Pasquale A, Cammà C, Puglia I, Calistri P, Savini G
EPI_ISL_1036150	SIESP CH	Istituto Zooprofilattico Sperimentale dell'Abruzzo e Molise "G. Caporale"	Lorusso A, Marcacci M, Di Domenico M, Ancora M, Curini V, Mangone I, Rinaldi A, Scialabba S, Di Pasquale A, Cammà C, Puglia I, Calistri P, Savini G
EPI_ISL_1036151	PRES OSP TAGLIACOZZO	Istituto Zooprofilattico Sperimentale dell'Abruzzo e Molise "G. Caporale"	Lorusso A, Marcacci M, Di Domenico M, Ancora M, Curini V, Mangone I, Rinaldi A, Scialabba S, Di Pasquale A, Cammà C, Puglia I, Calistri P, Savini G
EPI_ISL_1036153	SIESP AQ	Istituto Zooprofilattico Sperimentale dell'Abruzzo e Molise "G. Caporale"	Lorusso A, Marcacci M, Di Domenico M, Ancora M, Curini V, Mangone I, Rinaldi A, Scialabba S, Di Pasquale A, Cammà C, Puglia I, Calistri P, Savini G
EPI_ISL_1036154, EPI_ISL_1036155	SIESP TERAMO	Istituto Zooprofilattico Sperimentale dell'Abruzzo e Molise "G. Caporale"	Lorusso A, Marcacci M, Di Domenico M, Ancora M, Curini V, Mangone I, Rinaldi A, Scialabba S, Di Pasquale A, Cammà C, Puglia I, Calistri P, Savini G
EPI_ISL_1036156	SIESP DIP PREV CHIETI	Istituto Zooprofilattico Sperimentale dell'Abruzzo e Molise "G. Caporale"	Lorusso A, Marcacci M, Di Domenico M, Ancora M, Curini V, Mangone I, Rinaldi A, Scialabba S, Di Pasquale A, Cammà C, Puglia I, Calistri P, Savini G
EPI_ISL_1036157	SIESP TERAMO	Istituto Zooprofilattico Sperimentale dell'Abruzzo e Molise "G. Caporale"	Lorusso A, Marcacci M, Di Domenico M, Ancora M, Curini V, Mangone I, Rinaldi A, Scialabba S, Di Pasquale A, Cammà C, Puglia I, Calistri P, Savini G
EPI_ISL_1036158	OSP SAN SALVATORE MED INT	Istituto Zooprofilattico Sperimentale dell'Abruzzo e Molise "G. Caporale"	Lorusso A, Marcacci M, Di Domenico M, Ancora M, Curini V, Mangone I, Rinaldi A, Scialabba S, Di Pasquale A, Cammà C, Puglia I, Calistri P, Savini G
EPI_ISL_1036159	OSP CIV GIULIANOVA PRONTO SOCC	Istituto Zooprofilattico Sperimentale dell'Abruzzo e Molise "G. Caporale"	Lorusso A, Marcacci M, Di Domenico M, Ancora M, Curini V, Mangone I, Rinaldi A, Scialabba S, Di Pasquale A, Cammà C, Puglia I, Calistri P, Savini G
EPI_ISL_1036160	SIESP SULM	Istituto Zooprofilattico Sperimentale dell'Abruzzo e Molise "G.	Lorusso A, Marcacci M, Di Domenico M, Ancora M, Curini V, Mangone I, Rinaldi A, Scialabba S, Di Pasquale A, Cammà C, Puglia I, Calistri P, Savini G

		Caporale"	
EPI_ISL_1036161	OSP CIV TERAMO RIANIMAZIONE CARDIOCHIR	Istituto Zooprofilattico Sperimentale dell'Abruzzo e Molise "G. Caporale"	Lorusso A, Marcacci M, Di Domenico M, Ancora M, Curini V, Mangone I, Rinaldi A, Scialabba S, Di Pasquale A, Cammà C, Puglia I, Calistri P, Savini G
EPI_ISL_1036162	SIESP TERAMO	Istituto Zooprofilattico Sperimentale dell'Abruzzo e Molise "G. Caporale"	Lorusso A, Marcacci M, Di Domenico M, Ancora M, Curini V, Mangone I, Rinaldi A, Scialabba S, Di Pasquale A, Cammà C, Puglia I, Calistri P, Savini G
EPI_ISL_1036163, EPI_ISL_1036164	SIESP CH	Istituto Zooprofilattico Sperimentale dell'Abruzzo e Molise "G. Caporale"	Lorusso A, Marcacci M, Di Domenico M, Ancora M, Curini V, Mangone I, Rinaldi A, Scialabba S, Di Pasquale A, Cammà C, Puglia I, Calistri P, Savini G
EPI_ISL_1036165	SIESP TERAMO	Istituto Zooprofilattico Sperimentale dell'Abruzzo e Molise "G. Caporale"	Lorusso A, Marcacci M, Di Domenico M, Ancora M, Curini V, Mangone I, Rinaldi A, Scialabba S, Di Pasquale A, Cammà C, Puglia I, Calistri P, Savini G
EPI_ISL_1036166, EPI_ISL_1036167	SIESP CH	Istituto Zooprofilattico Sperimentale dell'Abruzzo e Molise "G. Caporale"	Lorusso A, Marcacci M, Di Domenico M, Ancora M, Curini V, Mangone I, Rinaldi A, Scialabba S, Di Pasquale A, Cammà C, Puglia I, Calistri P, Savini G
EPI_ISL_1036168, EPI_ISL_1036169	SIESP TERAMO	Istituto Zooprofilattico Sperimentale dell'Abruzzo e Molise "G. Caporale"	Lorusso A, Marcacci M, Di Domenico M, Ancora M, Curini V, Mangone I, Rinaldi A, Scialabba S, Di Pasquale A, Cammà C, Puglia I, Calistri P, Savini G
EPI_ISL_1036170, EPI_ISL_1036171	SIESP CH	Istituto Zooprofilattico Sperimentale dell'Abruzzo e Molise "G. Caporale"	Lorusso A, Marcacci M, Di Domenico M, Ancora M, Curini V, Mangone I, Rinaldi A, Scialabba S, Di Pasquale A, Cammà C, Puglia I, Calistri P, Savini G
EPI_ISL_1036172	SIESP TERAMO	Istituto Zooprofilattico Sperimentale dell'Abruzzo e Molise "G. Caporale"	Lorusso A, Marcacci M, Di Domenico M, Ancora M, Curini V, Mangone I, Rinaldi A, Scialabba S, Di Pasquale A, Cammà C, Puglia I, Calistri P, Savini G
EPI_ISL_1036173, EPI_ISL_1036174, EPI_ISL_1036175, EPI_ISL_1036176	SIESP CH	Istituto Zooprofilattico Sperimentale dell'Abruzzo e Molise "G. Caporale"	Lorusso A, Marcacci M, Di Domenico M, Ancora M, Curini V, Mangone I, Rinaldi A, Scialabba S, Di Pasquale A, Cammà C, Puglia I, Calistri P, Savini G
EPI_ISL_1036177	SIESP DIP PREV CHIETI	Istituto Zooprofilattico Sperimentale dell'Abruzzo e Molise "G. Caporale"	Lorusso A, Marcacci M, Di Domenico M, Ancora M, Curini V, Mangone I, Rinaldi A, Scialabba S, Di Pasquale A, Cammà C, Puglia I, Calistri P, Savini G
EPI_ISL_1036178	SIESP SULM	Istituto Zooprofilattico Sperimentale dell'Abruzzo e Molise "G. Caporale"	Lorusso A, Marcacci M, Di Domenico M, Ancora M, Curini V, Mangone I, Rinaldi A, Scialabba S, Di Pasquale A, Cammà C, Puglia I, Calistri P, Savini G
EPI_ISL_1036179	SIESP CH DISTRETTO SANITARIO CHIETI	Istituto Zooprofilattico Sperimentale dell'Abruzzo e Molise "G. Caporale"	Lorusso A, Marcacci M, Di Domenico M, Ancora M, Curini V, Mangone I, Rinaldi A, Scialabba S, Di Pasquale A, Cammà C, Puglia I, Calistri P, Savini G
EPI_ISL_1036180, EPI_ISL_1036181	SIESP TERAMO	Istituto Zooprofilattico Sperimentale dell'Abruzzo e Molise "G. Caporale"	Lorusso A, Marcacci M, Di Domenico M, Ancora M, Curini V, Mangone I, Rinaldi A, Scialabba S, Di Pasquale A, Cammà C, Puglia I, Calistri P, Savini G
EPI_ISL_1036182	SIESP CH	Istituto Zooprofilattico Sperimentale dell'Abruzzo e Molise "G. Caporale"	Lorusso A, Marcacci M, Di Domenico M, Ancora M, Curini V, Mangone I, Rinaldi A, Scialabba S, Di Pasquale A, Cammà C, Puglia I, Calistri P, Savini G
EPI_ISL_1036183, EPI_ISL_1036184	SIESP SULM	Istituto Zooprofilattico Sperimentale dell'Abruzzo e Molise "G. Caporale"	Lorusso A, Marcacci M, Di Domenico M, Ancora M, Curini V, Mangone I, Rinaldi A, Scialabba S, Di Pasquale A, Cammà C, Puglia I, Calistri P, Savini G
EPI_ISL_1036185	SIESP DIP PREV CHIETI	Istituto Zooprofilattico Sperimentale dell'Abruzzo e Molise "G. Caporale"	Lorusso A, Marcacci M, Di Domenico M, Ancora M, Curini V, Mangone I, Rinaldi A, Scialabba S, Di Pasquale A, Cammà C, Puglia I, Calistri P, Savini G
EPI_ISL_1036186	OSP SAN SALVATORE MED INT	Istituto Zooprofilattico Sperimentale dell'Abruzzo e Molise "G. Caporale"	Lorusso A, Marcacci M, Di Domenico M, Ancora M, Curini V, Mangone I, Rinaldi A, Scialabba S, Di Pasquale A, Cammà C, Puglia I, Calistri P, Savini G
EPI_ISL_1036187	SIESP TERAMO	Istituto Zooprofilattico Sperimentale dell'Abruzzo e Molise "G. Caporale"	Lorusso A, Marcacci M, Di Domenico M, Ancora M, Curini V, Mangone I, Rinaldi A, Scialabba S, Di Pasquale A, Cammà C, Puglia I, Calistri P, Savini G
EPI_ISL_1036188	OSP SAN SALVATORE UOC MAL INF	Istituto Zooprofilattico Sperimentale dell'Abruzzo e Molise "G. Caporale"	Lorusso A, Marcacci M, Di Domenico M, Ancora M, Curini V, Mangone I, Rinaldi A, Scialabba S, Di Pasquale A, Cammà C, Puglia I, Calistri P, Savini G
EPI_ISL_1036189	OSP CIV TERAMO PNEUMOLOGIA	Istituto Zooprofilattico Sperimentale dell'Abruzzo e Molise "G. Caporale"	Lorusso A, Marcacci M, Di Domenico M, Ancora M, Curini V, Mangone I, Rinaldi A, Scialabba S, Di Pasquale A, Cammà C, Puglia I, Calistri P, Savini G
EPI_ISL_1036190	SIESP CH	Istituto Zooprofilattico Sperimentale dell'Abruzzo e Molise "G. Caporale"	Lorusso A, Marcacci M, Di Domenico M, Ancora M, Curini V, Mangone I, Rinaldi A, Scialabba S, Di Pasquale A, Cammà C, Puglia I, Calistri P, Savini G
EPI_ISL_1036191	SIESP SULM	Istituto Zooprofilattico Sperimentale dell'Abruzzo e Molise "G. Caporale"	Lorusso A, Marcacci M, Di Domenico M, Ancora M, Curini V, Mangone I, Rinaldi A, Scialabba S, Di Pasquale A, Cammà C, Puglia I, Calistri P, Savini G
EPI_ISL_1036192	SIESP TERAMO	Istituto Zooprofilattico Sperimentale dell'Abruzzo e Molise "G. Caporale"	Lorusso A, Marcacci M, Di Domenico M, Ancora M, Curini V, Mangone I, Rinaldi A, Scialabba S, Di Pasquale A, Cammà C, Puglia I, Calistri P, Savini G
EPI_ISL_1036193, EPI_ISL_1036194, EPI_ISL_1036195, EPI_ISL_1036196, EPI_ISL_1036197	SIESP CH	Istituto Zooprofilattico Sperimentale dell'Abruzzo e Molise "G. Caporale"	Lorusso A, Marcacci M, Di Domenico M, Ancora M, Curini V, Mangone I, Rinaldi A, Scialabba S, Di Pasquale A, Cammà C, Puglia I, Calistri P, Savini G
EPI_ISL_1036198	ASL AVEZZANO-SULMONA-L'AQUILA DIP PREV SERV IGIENE E SAN PUBBLICA	Istituto Zooprofilattico Sperimentale dell'Abruzzo e Molise "G. Caporale"	Lorusso A, Marcacci M, Di Domenico M, Ancora M, Curini V, Mangone I, Rinaldi A, Scialabba S, Di Pasquale A, Cammà C, Puglia I, Calistri P, Savini G
EPI_ISL_1036199, EPI_ISL_1036200, EPI_ISL_1036201	SIESP CH	Istituto Zooprofilattico Sperimentale dell'Abruzzo e Molise "G. Caporale"	Lorusso A, Marcacci M, Di Domenico M, Ancora M, Curini V, Mangone I, Rinaldi A, Scialabba S, Di Pasquale A, Cammà C, Puglia I, Calistri P, Savini G
EPI_ISL_1036202	SIESP TERAMO	Istituto Zooprofilattico Sperimentale dell'Abruzzo e Molise "G. Caporale"	Lorusso A, Marcacci M, Di Domenico M, Ancora M, Curini V, Mangone I, Rinaldi A, Scialabba S, Di Pasquale A, Cammà C, Puglia I, Calistri P, Savini G
EPI_ISL_1036203, EPI_ISL_1036204	SIESP CH	Istituto Zooprofilattico Sperimentale dell'Abruzzo e Molise "G. Caporale"	Lorusso A, Marcacci M, Di Domenico M, Ancora M, Curini V, Mangone I, Rinaldi A, Scialabba S, Di Pasquale A, Cammà C, Puglia I, Calistri P, Savini G
EPI_ISL_1036205	PRES TERR CASOLI	Istituto Zooprofilattico Sperimentale dell'Abruzzo e Molise "G. Caporale"	Lorusso A, Marcacci M, Di Domenico M, Ancora M, Curini V, Mangone I, Rinaldi A, Scialabba S, Di Pasquale A, Cammà C, Puglia I, Calistri P, Savini G
EPI_ISL_1036206	SIESP AQ	Istituto Zooprofilattico Sperimentale dell'Abruzzo e Molise "G. Caporale"	Lorusso A, Marcacci M, Di Domenico M, Ancora M, Curini V, Mangone I, Rinaldi A, Scialabba S, Di Pasquale A, Cammà C, Puglia I, Calistri P, Savini G
EPI_ISL_1036207, EPI_ISL_1036208	SIESP TERAMO	Istituto Zooprofilattico Sperimentale dell'Abruzzo e Molise "G. Caporale"	Lorusso A, Marcacci M, Di Domenico M, Ancora M, Curini V, Mangone I, Rinaldi A, Scialabba S, Di Pasquale A, Cammà C, Puglia I, Calistri P, Savini G
EPI_ISL_1036209	SIESP DIP PREV CHIETI	Istituto Zooprofilattico Sperimentale dell'Abruzzo e Molise "G. Caporale"	Lorusso A, Marcacci M, Di Domenico M, Ancora M, Curini V, Mangone I, Rinaldi A, Scialabba S, Di Pasquale A, Cammà C, Puglia I, Calistri P, Savini G
EPI_ISL_1036210	SIESP TERAMO	Istituto Zooprofilattico Sperimentale dell'Abruzzo e Molise "G. Caporale"	Lorusso A, Marcacci M, Di Domenico M, Ancora M, Curini V, Mangone I, Rinaldi A, Scialabba S, Di Pasquale A, Cammà C, Puglia I, Calistri P, Savini G
EPI_ISL_1036211	ASL AVEZZANO-SULMONA-L'AQUILA	Istituto Zooprofilattico Sperimentale dell'Abruzzo e Molise "G. Caporale"	Lorusso A, Marcacci M, Di Domenico M, Ancora M, Curini V, Mangone I, Rinaldi A, Scialabba S, Di Pasquale A, Cammà C, Puglia I, Calistri P, Savini G

EPI_ISL_1036212, EPI_ISL_1036215, EPI_ISL_1036216	SIESP TERAMO	Istituto Zooprofilattico Sperimentale dell'Abruzzo e Molise "G. Caporale"	Lorusso A, Marcacci M, Di Domenico M, Ancora M, Curini V, Mangone I, Rinaldi A, Scialabba S, Di Pasquale A, Cammà C, Puglia I, Calistri P, Savini G
EPI_ISL_1036217	FONDAZIONE PICCOLA OPERA CARITAS	Istituto Zooprofilattico Sperimentale dell'Abruzzo e Molise "G. Caporale"	Lorusso A, Marcacci M, Di Domenico M, Ancora M, Curini V, Mangone I, Rinaldi A, Scialabba S, Di Pasquale A, Cammà C, Puglia I, Calistri P, Savini G
EPI_ISL_1036221	OSP SAN SALVATORE UOC MAL INF	Istituto Zooprofilattico Sperimentale dell'Abruzzo e Molise "G. Caporale"	Lorusso A, Marcacci M, Di Domenico M, Ancora M, Curini V, Mangone I, Rinaldi A, Scialabba S, Di Pasquale A, Cammà C, Puglia I, Calistri P, Savini G
EPI_ISL_1036222	SIESP TERAMO	Istituto Zooprofilattico Sperimentale dell'Abruzzo e Molise "G. Caporale"	Lorusso A, Marcacci M, Di Domenico M, Ancora M, Curini V, Mangone I, Rinaldi A, Scialabba S, Di Pasquale A, Cammà C, Puglia I, Calistri P, Savini G
EPI_ISL_1036223	OSP SAN SALVATORE MED INT	Istituto Zooprofilattico Sperimentale dell'Abruzzo e Molise "G. Caporale"	Lorusso A, Marcacci M, Di Domenico M, Ancora M, Curini V, Mangone I, Rinaldi A, Scialabba S, Di Pasquale A, Cammà C, Puglia I, Calistri P, Savini G
EPI_ISL_1036224, EPI_ISL_1036225, EPI_ISL_1036226, EPI_ISL_1036227, EPI_ISL_1036228, EPI_ISL_1036229	P.O.CARDARELLI	Istituto Zooprofilattico Sperimentale dell'Abruzzo e Molise "G. Caporale"	Scutellà M, Niro G, Lorusso A, Marcacci M, Di Domenico M, Ancora M, Curini V, Mangone I, Rinaldi A, Scialabba S, Di Pasquale A, Cammà C, Puglia I, Calistri P, Savini G
EPI_ISL_1036238, EPI_ISL_1036239	Center of Advanced Studies and Technology, Molecular Genetics Laboratory	Center of Advanced Studies and Technology, Molecular Genetics Laboratory	Ferrante Rossella, Mandatori Domitilla, De Fabritiis Simone, Damiani Verena, Anaclerio Federico
EPI_ISL_1036755	Unità Operativa di Microbiologia, IRCCS Policlinico di Sant'Orsola, Azienda Ospedaliero-Universitaria di Bologna	Unità di Analisi del Rischio ed Epidemiologia Genomica, Istituto Zooprofilattico Sperimentale dell'Emilia Romagna e della Lombardia (IZSLER)	Giada Rossini, Giuliano Furlini, Tiziana Lazzarotto, Marina Morganti, Ilaria Menozzi, Erika Scaltriti, Stefano Pongolini
EPI_ISL_1039782	INMI Lazzaro Spallanzani IRCCS	INMI Lazzaro Spallanzani IRCCS	CEM Gruber, B Bartolini, E Giombini, F Messina, M Rueca, O Butera, A Di Caro, MR Capobianchi
EPI_ISL_1039783	INMI Lazzaro Spallanzani IRCCS	INMI Lazzaro Spallanzani IRCCS	B Bartolini, M. Rueca, E Giombini, O Butera, C.E.M Gruber, F Messina, MR Capobianchi, A Di Caro
EPI_ISL_1039784	Azienda Ospedaliera San Camillo Forlanini	INMI Lazzaro Spallanzani IRCCS	CEM Gruber, B Bartolini, E Giombini, M Rueca, O Butera, F Messina, D.Gallone, G Parisi, ML Guarino, A Di Caro, MR Capobianchi
EPI_ISL_1039785	Ospedale "F. Spaziani" Frosinone	INMI Lazzaro Spallanzani IRCCS	B Bartolini, E Giombini, C.E.M Gruber, F Messina, M Rueca, O Butera, C. Gargiulo, C Sias, R Pulselli, A Di Caro, MR Capobianchi
EPI_ISL_1040918, EPI_ISL_1040919, EPI_ISL_1040920	Medicine and Surgery, University of Insubria	Medicine and Surgery, University of Insubria	Novazzi,F., Genoni,A., Baj,A., Spezia,P.G., Focosi,D., Zago,C., Colombo,A., Cassani,G., Pasciuta,R., Tamborini,A., Rossi,A., Prestia,M., Capuano,R., Maggi,F.
EPI_ISL_1048822, EPI_ISL_1048823, EPI_ISL_1048824, EPI_ISL_1048825, EPI_ISL_1048826, EPI_ISL_1048827, EPI_ISL_1048828	Azienda Sanitaria dell'Alto Adige Laboratorio Aziendale di Microbiologia e Virologia	Istituto di Genomica Applicata	Elisabetta Pagani, Irene Bianconi, Elisabetta Giacobazzi, Elisa Masi, Stefanie Wieser, Irena Jurman, Vera Vendramin, Gabriele Magris, Eleonora Paparelli, Davide Scaglione, Michele Morgante
EPI_ISL_1049260	Struttura Semplice Dipartimentale di Virologia e Microbiologia molecolare, Azienda Ospedaliero-Universitaria, Policlinico di Modena	U.O. Microbiologia, Laboratorio Unico Centro Servizi - AUSL della Romagna	Pecorari Monica, Gennari William, Fregni Serpini Giulia, Giorgio Dirani, Silvia Zannoli, Vittorio Sambri
EPI_ISL_1049261	U.O. Microbiologia Laboratorio Unico Centro Servizi AUSL della Romagna	U.O. Microbiologia Laboratorio Unico Centro Servizi AUSL della Romagna	Silvia Zannoli, Giorgio Dirani, Vittorio Sambri
EPI_ISL_1055765	Virology Unit, Pisa University Hospital	Virology Unit, AOUP	Marialinda Vatteroni, Susi Frateschi
EPI_ISL_1055792	Virology Unit, Pisa University Hospital	Virology Unit, AOUP, Pisa	Marialinda Vatteroni, Susi Frateschi
EPI_ISL_1055800	Virology Unit, AOUP	Virology Unit, AOUP, Pisa	Marialinda Vatteroni, Susi Frateschi
EPI_ISL_1055811	Virology Unit, AOUP	Virolgy Unit, AOUP	Marialinda Vatteroni, Susi Frateschi
EPI_ISL_1055815	Virology Unit, AOUP	Virology Unit, AOUP, Pisa	Marialinda Vatteroni, Susi Frateschi, Mauro Pistello
EPI_ISL_1055820	Virology Unit, AOUP	Virology Unit, AOUP	Marialinda Vatteroni, Susi Frateschi
EPI_ISL_1055824	Virology Unit, AOUP, Pisa, Italy	Virology Unit, AOUP, Pisa	Marialinda Vatteroni, Susi Frateschi, Mauro Pistello
EPI_ISL_1055825	Virology Unit, AOUP	Virology Unit, AOUP	Marialinda Vatteroni, Susi Frateschi
EPI_ISL_1058040	SIESP CHIETI DRIVE IN LANCIANO	Istituto Zooprofilattico Sperimentale dell'Abruzzo e Molise "G. Caporale"	Lorusso A, Marcacci M, Di Domenico M, Ancora M, Curini V, Mangone I, Rinaldi A, Scialabba S, Di Pasquale A, Cammà C, Puglia I, Calistri P, Savini G
EPI_ISL_1058041	SIESP CHIETI	Istituto Zooprofilattico Sperimentale dell'Abruzzo e Molise "G. Caporale"	Lorusso A, Marcacci M, Di Domenico M, Ancora M, Curini V, Mangone I, Rinaldi A, Scialabba S, Di Pasquale A, Cammà C, Puglia I, Calistri P, Savini G
EPI_ISL_1058042	SIESP CHIETI - DRIVE IN ORTONA	Istituto Zooprofilattico Sperimentale dell'Abruzzo e Molise "G. Caporale"	Lorusso A, Marcacci M, Di Domenico M, Ancora M, Curini V, Mangone I, Rinaldi A, Scialabba S, Di Pasquale A, Cammà C, Puglia I, Calistri P, Savini G
EPI_ISL_1058043	SIESP CHIETI - DRIVE IN CHIETI	Istituto Zooprofilattico Sperimentale dell'Abruzzo e Molise "G. Caporale"	Lorusso A, Marcacci M, Di Domenico M, Ancora M, Curini V, Mangone I, Rinaldi A, Scialabba S, Di Pasquale A, Cammà C, Puglia I, Calistri P, Savini G
EPI_ISL_1058044, EPI_ISL_1058045	SIESP CHIETI - DRIVE IN ORTONA	Istituto Zooprofilattico Sperimentale dell'Abruzzo e Molise "G. Caporale"	Lorusso A, Marcacci M, Di Domenico M, Ancora M, Curini V, Mangone I, Rinaldi A, Scialabba S, Di Pasquale A, Cammà C, Puglia I, Calistri P, Savini G
EPI_ISL_1061067, EPI_ISL_1061091, EPI_ISL_1061092, EPI_ISL_1061093, EPI_ISL_1061094, EPI_ISL_1061095, EPI_ISL_1061096, EPI_ISL_1061097, EPI_ISL_1061098, EPI_ISL_1061100, EPI_ISL_1061101, EPI_ISL_1061102, EPI_ISL_1061103, EPI_ISL_1061104, EPI_ISL_1061105, EPI_ISL_1061106, EPI_ISL_1061107, EPI_ISL_1061108, EPI_ISL_1061109, EPI_ISL_1061110, EPI_ISL_1061111, EPI_ISL_1061112, EPI_ISL_1061113, EPI_ISL_1061114, EPI_ISL_1061115, EPI_ISL_1061116, EPI_ISL_1061117, EPI_ISL_1061118, EPI_ISL_1061119, EPI_ISL_1061120, EPI_ISL_1061121, EPI_ISL_1061122, EPI_ISL_1061123, EPI_ISL_1061124, EPI_ISL_1061125, EPI_ISL_1061126, EPI_ISL_1061127, EPI_ISL_1061128, EPI_ISL_1061129, EPI_ISL_1061130, EPI_ISL_1061141, EPI_ISL_1061150, EPI_ISL_1061151, EPI_ISL_1061152, EPI_ISL_1061153, EPI_ISL_1061154, EPI_ISL_1061157, EPI_ISL_1061192, EPI_ISL_1061206, EPI_ISL_1061207, EPI_ISL_1061208, EPI_ISL_1061209, EPI_ISL_1061210, EPI_ISL_1061225, EPI_ISL_1061252, EPI_ISL_1061253, EPI_ISL_1061254, EPI_ISL_1061255, EPI_ISL_1061256, EPI_ISL_1061257, EPI_ISL_1061258, EPI_ISL_1061259			
see above	Istituto Zooprofilattico Sperimentale del Mezzogiorno (IZSM)	Telethon Institute of Genetics and Medicine (TIGEM)	Antonio Grimaldi, Patrizia Annunziata, Francesco Panariello, Biancamaria Pierri, Valentina Bouche, Chiara Colantuono, Maria Concetta Cuomo, Denise Di Concilio, Lucio Di Filippo, Anna Manfredi, Marcello Salvi, Antonio Limone, Pellegrino Cerino, Andrea Ballabio, Davide Cacchiarelli.
EPI_ISL_1063404, EPI_ISL_1063405, EPI_ISL_1063406, EPI_ISL_1063407, EPI_ISL_1063408, EPI_ISL_1063409, EPI_ISL_1063410, EPI_ISL_1063411, EPI_ISL_1063412, EPI_ISL_1063413, EPI_ISL_1063414, EPI_ISL_1063415, EPI_ISL_1063416, EPI_ISL_1063417, EPI_ISL_1063418, EPI_ISL_1063419, EPI_ISL_1063420, EPI_ISL_1063421, EPI_ISL_1063422, EPI_ISL_1063423, EPI_ISL_1063424, EPI_ISL_1063425, EPI_ISL_1063426, EPI_ISL_1063427, EPI_ISL_1063428, EPI_ISL_1063429, EPI_ISL_1063430, EPI_ISL_1063431, EPI_ISL_1063432, EPI_ISL_1063433, EPI_ISL_1063434, EPI_ISL_1063435, EPI_ISL_1063436, EPI_ISL_1063437, EPI_ISL_1063438, EPI_ISL_1063439, EPI_ISL_1063440, EPI_ISL_1063441, EPI_ISL_1063442, EPI_ISL_1063443, EPI_ISL_1063444, EPI_ISL_1063445, EPI_ISL_1063446, EPI_ISL_1063447, EPI_ISL_1063448, EPI_ISL_1063449, EPI_ISL_1063450, EPI_ISL_1063451, EPI_ISL_1063452, EPI_ISL_1063453, EPI_ISL_1063454, EPI_ISL_1063455, EPI_ISL_1063456, EPI_ISL_1063457, EPI_ISL_1063458, EPI_ISL_1063459, EPI_ISL_1063460, EPI_ISL_1063461, EPI_ISL_1063462, EPI_ISL_1063463, EPI_ISL_1063464, EPI_ISL_1063465, EPI_ISL_1063466, EPI_ISL_1063467, EPI_ISL_1063468, EPI_ISL_1063469, EPI_ISL_1063470, EPI_ISL_1063471, EPI_ISL_1063472, EPI_ISL_1063473, EPI_ISL_1063474, EPI_ISL_1063475, EPI_ISL_1063476, EPI_ISL_1063477, EPI_ISL_1063478, EPI_ISL_1063479, EPI_ISL_1063480, EPI_ISL_1063481, EPI_ISL_1063482, EPI_ISL_1063483, EPI_ISL_1063484, EPI_ISL_1063485, EPI_ISL_1063486, EPI_ISL_1063487, EPI_ISL_1063488, EPI_ISL_1063489, EPI_ISL_1063490, EPI_ISL_1063491, EPI_ISL_1063492, EPI_ISL_1063493			
see above	Azienda Sanitaria dell'Alto Adige Laboratorio Aziendale di Microbiologia e Virologia	Istituto di Genomica Applicata	Elisabetta Pagani, Irene Bianconi, Elisabetta Giacobazzi, Elisa Masi, Stefanie Wieser, Irena Jurman, Vera Vendramin, Gabriele Magris, Eleonora Paparelli, Davide Scaglione, Michele Morgante
EPI_ISL_1063785, EPI_ISL_1063786, EPI_ISL_1063787	SIESP CHIETI - DRIVE IN ORTONA	Istituto Zooprofilattico Sperimentale dell'Abruzzo e Molise "G. Caporale"	Lorusso A, Marcacci M, Di Domenico M, Ancora M, Curini V, Mangone I, Rinaldi A, Scialabba S, Di Pasquale A, Cammà C, Puglia I, Calistri P, Savini G
EPI_ISL_1063788	P.O.CARDARELLI	Istituto Zooprofilattico Sperimentale dell'Abruzzo e Molise "G. Caporale"	Scutellà M, Niro G, Lorusso A, Marcacci M, Di Domenico M, Ancora M, Curini V, Mangone I, Rinaldi A, Scialabba S, Di Pasquale A, Cammà C, Puglia I, Calistri P, Savini G
EPI_ISL_1063909	Laboratorio Genzano - ASL RM 6	INMI Lazzaro Spallanzani IRCCS	B Bartolini, O Butera, C.E.M Gruber, M Rueca, F Messina, E Giombini, G Tramini, E Conti, MR Capobianchi, A Di Caro

EPI_ISL_1063910	Ospedale San Filippo Neri	INMI Lazzaro Spallanzani IRCCS	M Rueca, O Butera, F Messina, CEM Gruber, B Bartolini, E Giombini, M Melandri, ML Schiavone, A Di Caro, MR Capobianchi
EPI_ISL_1063911	INMI Lazzaro Spallanzani IRCCS	INMI Lazzaro Spallanzani IRCCS	E Giombini, M. Rueca, B Bartolini, O Butera, CEM Gruber, F Messina, A Di Caro, MR Capobianchi
EPI_ISL_1063912	Laboratorio Biologia Molecolare Sars Cov2 - UOC Laboratorio Analisi - Servizio Medicina di Laboratorio, Ospedale "San Francesco" - ATS-ASSL Nuoro	Laboratorio specialistico UOC Ematologia - Ospedale "San Francesco" - ATS-ASSL Nuoro	Piras Giovanna, Asproni Rosanna, Malune Paolo, Fiamma Maura, Monne Maria Itria, Palmas Angelo Domenico, Lo Maglio Iana, Mameli Giuseppe
EPI_ISL_1069204	Azienda Sanitaria Locale Napoli 1	Centro Polidiagnostico strumentale AMES	Sirica Roberto
EPI_ISL_1073034	ASL Napoli 1 Centro	AMES Centro Polidiagnostico Strumentale S.r.l.	Giovanni Savarese, Raffaella Ruggiero, Eloisa Evangelista, Antonella Di Carlo, Luisa Circelli, Luigi D'Amore, Roberto Sirica, Antonio Fico
EPI_ISL_1074622, EPI_ISL_1074623	ASL Napoli 1 Centro	AMES Centro Polidiagnostico Strumentale S.r.l.	"Giovanni Savarese, Raffaella Ruggiero, Eloisa Evangelista, Antonella Di Carlo, Luisa Circelli, Luigi D'Amore, Roberto Sirica, Antonio Fico"
EPI_ISL_1079352	Genomic Medicine Laboratory, IRCCS Santa Lucia Foundation	INMI Lazzaro Spallanzani IRCCS	CEM Gruber, B Bartolini, E Giombini, F Messina, M Rueca, O Butera, E Giardina, A Di Caro, MR Capobianchi
EPI_ISL_1079353	IRCCS San Raffaele	INMI Lazzaro Spallanzani IRCCS	B Bartolini, E Giombini, F Messina, M Rueca, O Butera, CEM Gruber, D Russo, D Limongi, MR Capobianchi, A Di Caro
EPI_ISL_1079355	Genomic Medicine Laboratory, IRCCS Santa Lucia Foundation	INMI Lazzaro Spallanzani IRCCS	B Bartolini, E Giombini, F Messina, M Rueca, O Butera, CEM Gruber, E Giardina, MR Capobianchi, A Di Caro
EPI_ISL_1079356	Ospedale S.M. Goretti di Latina - ASL Latina presidio ospedaliero Nord	INMI Lazzaro Spallanzani IRCCS	F Messina, M Rueca, O Butera, CEM Gruber, B Bartolini, E Giombini, L Corso, L Di Biase, MR Capobianchi, A Di Caro
EPI_ISL_1079358	Ospedale S.M. Goretti di Latina - ASL Latina presidio ospedaliero Nord	INMI Lazzaro Spallanzani IRCCS	CEM Gruber, B Bartolini, E Giombini, F Messina, O Butera, M Rueca, L Corso, A Lucci, MR Capobianchi, A Di Caro
EPI_ISL_1079359	Fondazione Policlinico Tor Vergata	INMI Lazzaro Spallanzani IRCCS	CEM Gruber, B Bartolini, E Giombini, F Messina, M Rueca, O Butera, S Grelli, M Ciotti, C D'Agostini, A Di Caro, MR Capobianchi
EPI_ISL_1079360	Ospedale "F. Spaziani" Frosinone	INMI Lazzaro Spallanzani IRCCS	F Messina, M Rueca, O Butera, CEM Gruber, B Bartolini, E Giombini, C. Sias, R Pulselli, MR Capobianchi, A Di Caro
EPI_ISL_1079362	Azienda Ospedaliera San Camillo Forlanini	INMI Lazzaro Spallanzani IRCCS	M Rueca, O Butera, CEM Gruber, B Bartolini, E Giombini, F Messina, D Gallone, A D'Agostino, A Di Caro, MR Capobianchi
EPI_ISL_1079363	Azienda Ospedaliera San Camillo Forlanini	INMI Lazzaro Spallanzani IRCCS	B Bartolini, E Giombini, M Rueca, O Butera, CEM Gruber,F Messina, D Gallone, G Meoni, A Di Caro, MR Capobianchi
EPI_ISL_1079364	Ospedale San Camillo De Lellis di Rieti	INMI Lazzaro Spallanzani IRCCS	B Bartolini, E Giombini, F Messina, M Rueca, O Butera, CEM Gruber, S Venarubea, A De Luca, MR Capobianchi, A Di Caro
EPI_ISL_1079366	Ospedale San Camillo De Lellis di Rieti	INMI Lazzaro Spallanzani IRCCS	E Giombini, F Messina, M Rueca, O Butera, CEM Gruber, B Bartolini, A De Luca, L Casertano, A Di Caro, MR Capobianchi
EPI_ISL_1079368	Ospedale Sandro Pertini ASL Roma2	INMI Lazzaro Spallanzani IRCCS	F Messina, M Rueca, O Butera, CEM Gruber, B Bartolini, E Giombini, MC Cava, G Cappiello, MR Capobianchi, A Di Caro
EPI_ISL_1079370	Ospedale Sandro Pertini ASL Roma2	INMI Lazzaro Spallanzani IRCCS	M Rueca, O Butera, CEM Gruber, B Bartolini, E Giombini, F Messina, MC Cava, S Romano, A Di Caro, MR Capobianchi
EPI_ISL_1079371	Fondazione Policlinico Tor Vergata	INMI Lazzaro Spallanzani IRCCS	CEM Gruber, B Bartolini, E Giombini, F Messina, O Butera, M Rueca, S Grelli, P Paba, D Ombres, MR Capobianchi, A Di Caro
EPI_ISL_1079373	Fondazione Policlinico Tor Vergata	INMI Lazzaro Spallanzani IRCCS	E Giombini, F Messina, M Rueca, O Butera, CEM Gruber, B Bartolini, S Grelli, C Fontana, D Di Cave, A Di Caro, MR Capobianchi
EPI_ISL_1079376	San Gallicano Dermatological Institute I.F.O.	INMI Lazzaro Spallanzani IRCCS	B Bartolini, E Giombini, F Messina, M Rueca, O Butera, CEM Gruber, F Pimpinelli, F Ensoli, G D'Agosto, MR Capobianchi, A Di Caro
EPI_ISL_1079378	San Gallicano Dermatological Institute I.F.O.	INMI Lazzaro Spallanzani IRCCS	E Giombini, F Messina, M Rueca, O Butera, CEM Gruber, B Bartolini, F Ensoli, F Pimpinelli, E Trento, A Di Caro, MR Capobianchi
EPI_ISL_1079380	Sant'Eugenio/CTO ASL Roma 2	INMI Lazzaro Spallanzani IRCCS	F Messina, M Rueca, O Butera, CEM Gruber, B Bartolini, E Giombini, F Bondanini, GC Cocciolillo, MR Capobianchi, A Di Caro
EPI_ISL_1079382	Sant'Eugenio/CTO ASL Roma 2	INMI Lazzaro Spallanzani IRCCS	M Rueca, O Butera, CEM Gruber, B Bartolini, E Giombini, F Messina, F Bondanini, C Disegni, A Di Caro, MR Capobianchi
EPI_ISL_1079383	Azienda Ospedaliera San Camillo Forlanini	INMI Lazzaro Spallanzani IRCCS	CEM Gruber, B Bartolini, O Butera, E Giombini, F Messina, M Rueca, R.Sperning, D Gallone, MR Capobianchi, A Di Caro
EPI_ISL_1079386	Azienda Ospedaliera San Camillo Forlanini	INMI Lazzaro Spallanzani IRCCS	E Giombini, M Rueca, O Butera, CEM Gruber, B Bartolini,F Messina, D Gallone, M Irno Consalvo, A Di Caro, MR Capobianchi
EPI_ISL_1079388	University Hospital Sant'Andrea-Sapienza	INMI Lazzaro Spallanzani IRCCS	E Giombini, F Messina, M Rueca, O Butera, CEM Gruber, B Bartolini, I Santino, M Simmaco, A Di Caro, MR Capobianchi
EPI_ISL_1079390	Laboratorio Genzano - ASL RM 6	INMI Lazzaro Spallanzani IRCCS	F Messina, M Rueca, O Butera, CEM Gruber, B Bartolini, E Giombini, G Tramini, E Conti, MR Capobianchi, A Di Caro
EPI_ISL_1079391	Laboratorio Genzano - ASL RM 6	INMI Lazzaro Spallanzani IRCCS	M Rueca, O Butera, CEM Gruber, B Bartolini, E Giombini, F Messina, E Conti, G Tramini, A Di Caro, MR Capobianchi
EPI_ISL_1079392	Policlinico Universitario Campus Bio-Medico	INMI Lazzaro Spallanzani IRCCS	O Butera, CEM Gruber, B Bartolini, E Giombini, F Messina, M Rueca, E Riva, S Angeletti, MR Capobianchi, A Di Caro
EPI_ISL_1079394	Policlinico Universitario Campus Bio-Medico	INMI Lazzaro Spallanzani IRCCS	CEM Gruber, B Bartolini, E Giombini, F Messina, M Rueca, O Butera, M Fogolari, L De Florio, E Riva, A Di Caro, MR Capobianchi
EPI_ISL_1079395	IRCCS San Raffaele	INMI Lazzaro Spallanzani IRCCS	E Giombini, F Messina, M Rueca, O Butera, CEM Gruber, B Bartolini, D Limongi, D Russo, A Di Caro, MR Capobianchi
EPI_ISL_1079398	Ospedale "F. Spaziani" Frosinone	INMI Lazzaro Spallanzani IRCCS	F Messina, M Rueca, O Butera, CEM Gruber, B Bartolini, E Giombini, R Pulselli, G Brocco, MR Capobianchi, A Di Caro
EPI_ISL_1079400	Ospedale San Filippo Neri	INMI Lazzaro Spallanzani IRCCS	M Rueca, O Butera, F Messina, CEM Gruber, B Bartolini, E Giombini, M Melandri, ML Schiavone, A Tamburro, A Di Caro, MR Capobianchi
EPI_ISL_1079992	Ospedale S.M. Goretti di Latina - ASL Latina presidio ospedaliero Nord	INMI Lazzaro Spallanzani IRCCS	CEM Gruber, B Bartolini, E Giombini, F Messina, O Butera, M Rueca, S Pignalosa, L Corso, MR Capobianchi, A Di Caro
EPI_ISL_1079993	Ospedale S.M. Goretti di Latina - ASL Latina presidio ospedaliero Nord	INMI Lazzaro Spallanzani IRCCS	B Bartolini, E Giombini, F Messina, O Butera, M Rueca, CEM Gruber, L Di Biase, A Lucci, A Di Caro, MR Capobianchi
EPI_ISL_1079994	Azienda Ospedaliera San Camillo Forlanini	INMI Lazzaro Spallanzani IRCCS	O Butera, E Giombini, F Messina, M Rueca, CEM Gruber, B Bartolini, G Parisi, MI Consalvo, MR Capobianchi, A Di Caro
EPI_ISL_1079995	INMI Lazzaro Spallanzani IRCCS	INMI Lazzaro Spallanzani IRCCS	E Giombini, F Messina, M Rueca, O Butera, CEM Gruber, B Bartolini, A Di Caro, MR Capobianchi
EPI_ISL_1079996	Ospedale Sandro Pertini ASL Roma2	INMI Lazzaro Spallanzani IRCCS	F Messina, M Rueca, O Butera, CEM Gruber, B Bartolini, E Giombini, MC Cava, G Cappiello, MR Capobianchi, A Di Caro
EPI_ISL_1079998	Ospedale San Filippo Neri	INMI Lazzaro Spallanzani IRCCS	M Rueca, O Butera, F Messina, CEM Gruber, B Bartolini, E Giombini, M Meledandri, ML Schiavone, MR Capobianchi
EPI_ISL_1079999	Ospedale San Filippo Neri	INMI Lazzaro Spallanzani IRCCS	CEM Gruber, B Bartolini, E Giombini, F Messina, O Butera, M Rueca, ML Schiavone, A Tamburro, A Di Caro, MR Capobianchi
EPI_ISL_1080001	Ospedale Sandro Pertini ASL Roma2	INMI Lazzaro Spallanzani IRCCS	B Bartolini, E Giombini, F Messina, O Butera, M Rueca, CEM Gruber, R Longo, V Michela, MR Capobianchi, A Di Caro
EPI_ISL_1080464, EPI_ISL_1080465, EPI_ISL_1080466, EPI_ISL_1080467, EPI_ISL_1080468, EPI_ISL_1080469, EPI_ISL_1080470, EPI_ISL_1080471, EPI_ISL_1080472, EPI_ISL_1080473, EPI_ISL_1080474, EPI_ISL_1080475, EPI_ISL_1080476, EPI_ISL_1080477, EPI_ISL_1080478, EPI_ISL_1080479, EPI_ISL_1080481, EPI_ISL_1080482, EPI_ISL_1080483, EPI_ISL_1080484, EPI_ISL_1080485, EPI_ISL_1080486, EPI_ISL_1080487, EPI_ISL_1080488, EPI_ISL_1080489, EPI_ISL_1080490, EPI_ISL_1080491, EPI_ISL_1080492, EPI_ISL_1080493, EPI_ISL_1080494, EPI_ISL_1080495			
see above	Istituto Zooprofilattico Sperimentale del Mezzogiorno	Istituto Zooprofilattico Sperimentale del Mezzogiorno-U.O.C. Virologia	Maurizio Viscardi, Lorena Cardillo, Loredana Cozzolino, Pellegrino Cerino, Massimo Zollo,Giovanna Fusco, Esterina De Carlo, Antonio Limone
EPI_ISL_1080605, EPI_ISL_1080606, EPI_ISL_1080607, EPI_ISL_1080608, EPI_ISL_1080609, EPI_ISL_1080610, EPI_ISL_1080611, EPI_ISL_1080612, EPI_ISL_1080613, EPI_ISL_1080614, EPI_ISL_1080615, EPI_ISL_1080616, EPI_ISL_1080617, EPI_ISL_1080618, EPI_ISL_1080619, EPI_ISL_1080620, EPI_ISL_1080621, EPI_ISL_1080622, EPI_ISL_1080623, EPI_ISL_1080624, EPI_ISL_1080625, EPI_ISL_1080626, EPI_ISL_1080627, EPI_ISL_1080628, EPI_ISL_1080629, EPI_ISL_1080630, EPI_ISL_1080631, EPI_ISL_1080632, EPI_ISL_1080633, EPI_ISL_1080634, EPI_ISL_1080635, EPI_ISL_1080636, EPI_ISL_1080637, EPI_ISL_1080638, EPI_ISL_1080639, EPI_ISL_1080640, EPI_ISL_1080641, EPI_ISL_1080642, EPI_ISL_1080643, EPI_ISL_1080644, EPI_ISL_1080645, EPI_ISL_1080646, EPI_ISL_1080647, EPI_ISL_1080648, EPI_ISL_1080649, EPI_ISL_1080650, EPI_ISL_1080651, EPI_ISL_1080652, EPI_ISL_1080653, EPI_ISL_1080654, EPI_ISL_1080655, EPI_ISL_1080656, EPI_ISL_1080657, EPI_ISL_1080658, EPI_ISL_1080659, EPI_ISL_1080660, EPI_ISL_1080661, EPI_ISL_1080662, EPI_ISL_1080663, EPI_ISL_1080664, EPI_ISL_1080665, EPI_ISL_1080666, EPI_ISL_1080667, EPI_ISL_1080668, EPI_ISL_1080669, EPI_ISL_1080670, EPI_ISL_1080671, EPI_ISL_1080672, EPI_ISL_1080673, EPI_ISL_1080674, EPI_ISL_1080675, EPI_ISL_1080676, EPI_ISL_1080677, EPI_ISL_1080678, EPI_ISL_1080679, EPI_ISL_1080681, EPI_ISL_1080682, EPI_ISL_1080683, EPI_ISL_1080684, EPI_ISL_1080685, EPI_ISL_1080686, EPI_ISL_1080687, EPI_ISL_1080688, EPI_ISL_1080689, EPI_ISL_1080690, EPI_ISL_1080691, EPI_ISL_1080692, EPI_ISL_1080693, EPI_ISL_1080694, EPI_ISL_1080695, EPI_ISL_1080696, EPI_ISL_1080697, EPI_ISL_1080698, EPI_ISL_1080699, EPI_ISL_1080700, EPI_ISL_1080701, EPI_ISL_1080702, EPI_ISL_1080703, EPI_ISL_1080704, EPI_ISL_1080705, EPI_ISL_1080706, EPI_ISL_1080707, EPI_ISL_1080708, EPI_ISL_1080709, EPI_ISL_1080710, EPI_ISL_1080711, EPI_ISL_1080712, EPI_ISL_1080713, EPI_ISL_1080714, EPI_ISL_1080715, EPI_ISL_1080716, EPI_ISL_1080717, EPI_ISL_1080718, EPI_ISL_1080719, EPI_ISL_1080720, EPI_ISL_1080721, EPI_ISL_1080722, EPI_ISL_1080723, EPI_ISL_1080724, EPI_ISL_1080725, EPI_ISL_1080726, EPI_ISL_1080727, EPI_ISL_1080728, EPI_ISL_1080729, EPI_ISL_1080730, EPI_ISL_1080731, EPI_ISL_1080732, EPI_ISL_1080733, EPI_ISL_1080734, EPI_ISL_1080735, EPI_ISL_1080736, EPI_ISL_1080737, EPI_ISL_1080738, EPI_ISL_1080739, EPI_ISL_1080740, EPI_ISL_1080741, EPI_ISL_1080742, EPI_ISL_1080743, EPI_ISL_1080744, EPI_ISL_1080745, EPI_ISL_1080746, EPI_ISL_1080747, EPI_ISL_1080748			
see above	ASL Napoli 1 Centro	AMES Centro Polidiagnostico Strumentale S.r.l.	"Giovanni Savarese, Raffaella Ruggiero, Eloisa Evangelista, Antonella Di Carlo, Luisa Circelli, Luigi D'Amore, Roberto Sirica, Antonio Fico"
EPI_ISL_1081953	Laboratorio di Riferimento Regionale della Sicilia Occidentale	Laboratorio di Riferimento Regionale della Sicilia Occidentale	Fabio Tramuto, Carmelo Massimo Maida, Daniela Di Naro, Giulia Randazzo, Walter Mazzucco, Giorgio Graziano, Vincenzo Restivo, Claudio Costantino,

	per l'Emergenza COVID-19	per l'Emergenza COVID-19	Stefano Giaramida, Francesco Vitale
EPI_ISL_1082253	Laboratorio Biologia Molecolare Sars Cov2 - UOC Laboratorio Analisi - Servizio Medicina di Laboratorio, Ospedale "San Francesco" - ATS-ASSL Nuoro	Laboratorio specialistico UOC Ematologia - Ospedale "San Francesco" - ATS-ASSL Nuoro	Giovanna Piras, Rosanna Asproni, Paolo Malune, Maura Fiamma, Maria Itria Monne, Angelo Domenico Palmas, Iana Lo Maglio, Giuseppe Mameli
EPI_ISL_1082254	Virologia, Dipartimento di Scienze Biomediche, Università di Sassari, Viale San Pietro, 43/B - Sassari	Laboratorio specialistico UOC Ematologia - Ospedale "San Francesco" - ATS-ASSL Nuoro	Giovanna Piras, Asproni Rosanna, Paolo Malune, Maria Itria Monne, Angelo Domenico Palmas, Caterina Serra, Elena Rimini, Salvatore Rubino
EPI_ISL_1082292, EPI_ISL_1082293, EPI_ISL_1082295	INT Fondazione Pascale	INT Fondazione Pascale	INT Fondazione Pascale
EPI_ISL_1082454, EPI_ISL_1082455, EPI_ISL_1082456, EPI_ISL_1082457, EPI_ISL_1082458, EPI_ISL_1082459, EPI_ISL_1082460, EPI_ISL_1082461, EPI_ISL_1082462, EPI_ISL_1082463, EPI_ISL_1082464, EPI_ISL_1082465, EPI_ISL_1082466, EPI_ISL_1082467, EPI_ISL_1082468, EPI_ISL_1082469, EPI_ISL_1082470, EPI_ISL_1082471, EPI_ISL_1082472, EPI_ISL_1082473, EPI_ISL_1082474, EPI_ISL_1082475, EPI_ISL_1082476, EPI_ISL_1082477, EPI_ISL_1082478, EPI_ISL_1082479, EPI_ISL_1082480, EPI_ISL_1082481, EPI_ISL_1082482, EPI_ISL_1082483, EPI_ISL_1082484, EPI_ISL_1082485, EPI_ISL_1082486, EPI_ISL_1082487, EPI_ISL_1082488, EPI_ISL_1082489, EPI_ISL_1082490, EPI_ISL_1082491, EPI_ISL_1082492, EPI_ISL_1082493, EPI_ISL_1082494, EPI_ISL_1082495, EPI_ISL_1082496, EPI_ISL_1082497, EPI_ISL_1082498, EPI_ISL_1082499, EPI_ISL_1082500, EPI_ISL_1082501, EPI_ISL_1082502, EPI_ISL_1082503, EPI_ISL_1082504, EPI_ISL_1082505, EPI_ISL_1082506, EPI_ISL_1082507, EPI_ISL_1082508, EPI_ISL_1082509, EPI_ISL_1082510, EPI_ISL_1082511, EPI_ISL_1082512, EPI_ISL_1082513, EPI_ISL_1082514, EPI_ISL_1082515, EPI_ISL_1082516, EPI_ISL_1082517, EPI_ISL_1082518, EPI_ISL_1082519, EPI_ISL_1082520, EPI_ISL_1082521, EPI_ISL_1082522, EPI_ISL_1082523, EPI_ISL_1082524, EPI_ISL_1082525, EPI_ISL_1085025, EPI_ISL_1085026, EPI_ISL_1085027, EPI_ISL_1085028, EPI_ISL_1085029, EPI_ISL_1085030, EPI_ISL_1085032, EPI_ISL_1085033, EPI_ISL_1085034, EPI_ISL_1085035, EPI_ISL_1085036, EPI_ISL_1085037, EPI_ISL_1085038, EPI_ISL_1085039, EPI_ISL_1085040, EPI_ISL_1085041, EPI_ISL_1085042, EPI_ISL_1085043, EPI_ISL_1085044, EPI_ISL_1085045, EPI_ISL_1085046, EPI_ISL_1085047, EPI_ISL_1085048, EPI_ISL_1085049, EPI_ISL_1085050, EPI_ISL_1085051, EPI_ISL_1085052, EPI_ISL_1085053, EPI_ISL_1085054, EPI_ISL_1085055, EPI_ISL_1085056, EPI_ISL_1085057, EPI_ISL_1085058, EPI_ISL_1085059, EPI_ISL_1085060, EPI_ISL_1085061, EPI_ISL_1085062, EPI_ISL_1085063, EPI_ISL_1085064, EPI_ISL_1085065, EPI_ISL_1085066, EPI_ISL_1085067, EPI_ISL_1085068, EPI_ISL_1085069, EPI_ISL_1085070, EPI_ISL_1085071, EPI_ISL_1085072, EPI_ISL_1085073, EPI_ISL_1085074, EPI_ISL_1085075, EPI_ISL_1085076, EPI_ISL_1085077, EPI_ISL_1085078, EPI_ISL_1085079, EPI_ISL_1085080, EPI_ISL_1085081, EPI_ISL_1085082, EPI_ISL_1085083, EPI_ISL_1085084, EPI_ISL_1085085, EPI_ISL_1085086, EPI_ISL_1085087, EPI_ISL_1085088, EPI_ISL_1085089, EPI_ISL_1085090			
see above	ASL Napoli 1 Centro	AMES Centro Polidiagnostico Strumentale S.r.l.	"Giovanni Savarese, Raffaella Ruggiero, Eloisa Evangelista, Antonella Di Carlo, Luisa Circelli, Luigi D'Amore, Roberto Sirica, Nadia Petrillo, Monica Ianniello, Antonio Fico"
EPI_ISL_1085094	Laboratory of Infectious Diseases, Department of Biomedical and Clinical Sciences L. Sacco, University of Milan	Laboratory of Infectious Diseases, Department of Biomedical and Clinical Sciences L. Sacco, University of Milan	Alessia Lai, Annalisa Bergna, Carla Della Ventura, Claudia Balotta, Massimo Galli, Gianguglielmo Zehender on behalf of SARS-CoV-2 ITALIAN RESEARCH ENTERPRISE-(SCIRE) Collaborative Group
EPI_ISL_1085096, EPI_ISL_1085097, EPI_ISL_1085098, EPI_ISL_1085099, EPI_ISL_1085100, EPI_ISL_1085101, EPI_ISL_1085102, EPI_ISL_1085103, EPI_ISL_1085104, EPI_ISL_1085105, EPI_ISL_1085106, EPI_ISL_1085107, EPI_ISL_1085108, EPI_ISL_1085109, EPI_ISL_1085110, EPI_ISL_1085111, EPI_ISL_1085112, EPI_ISL_1085113, EPI_ISL_1085114, EPI_ISL_1085115, EPI_ISL_1085116, EPI_ISL_1085117, EPI_ISL_1085118, EPI_ISL_1085119, EPI_ISL_1085120, EPI_ISL_1085121, EPI_ISL_1085122, EPI_ISL_1085123, EPI_ISL_1085124, EPI_ISL_1085125, EPI_ISL_1085126, EPI_ISL_1085127, EPI_ISL_1085128, EPI_ISL_1085129, EPI_ISL_1085130, EPI_ISL_1085131, EPI_ISL_1085132, EPI_ISL_1085133, EPI_ISL_1085134, EPI_ISL_1085135, EPI_ISL_1085136, EPI_ISL_1085137, EPI_ISL_1085138, EPI_ISL_1085139, EPI_ISL_1085140, EPI_ISL_1085141, EPI_ISL_1085142, EPI_ISL_1085143, EPI_ISL_1085144, EPI_ISL_1085145, EPI_ISL_1085146, EPI_ISL_1085147, EPI_ISL_1085148, EPI_ISL_1085149, EPI_ISL_1085150, EPI_ISL_1085151, EPI_ISL_1085152, EPI_ISL_1085153, EPI_ISL_1085154, EPI_ISL_1085155, EPI_ISL_1085156, EPI_ISL_1085157, EPI_ISL_1085158, EPI_ISL_1085159, EPI_ISL_1085160			
see above	ASL Napoli 1 Centro	AMES Centro Polidiagnostico Strumentale S.r.l.	"Giovanni Savarese, Raffaella Ruggiero, Eloisa Evangelista, Antonella Di Carlo, Luisa Circelli, Luigi D'Amore, Roberto Sirica, Nadia Petrillo, Monica Ianniello, Antonio Fico"
EPI_ISL_1085162, EPI_ISL_1085164, EPI_ISL_1085165, EPI_ISL_1085167, EPI_ISL_1085169, EPI_ISL_1085170, EPI_ISL_1085173, EPI_ISL_1085174	Laboratory of Infectious Diseases, Department of Biomedical and Clinical Sciences L. Sacco, University of Milan	Laboratory of Infectious Diseases, Department of Biomedical and Clinical Sciences L. Sacco, University of Milan	Alessia Lai, Annalisa Bergna, Carla Della Ventura, Claudia Balotta, Massimo Galli, Gianguglielmo Zehender on behalf of SARS-CoV-2 ITALIAN RESEARCH ENTERPRISE-(SCIRE) Collaborative Group
EPI_ISL_1085176, EPI_ISL_1085177, EPI_ISL_1085178, EPI_ISL_1085179, EPI_ISL_1085180, EPI_ISL_1085181, EPI_ISL_1085182, EPI_ISL_1085183, EPI_ISL_1085184, EPI_ISL_1085185, EPI_ISL_1085186, EPI_ISL_1085187, EPI_ISL_1085188, EPI_ISL_1085189, EPI_ISL_1085190, EPI_ISL_1085191, EPI_ISL_1085192, EPI_ISL_1085193, EPI_ISL_1085194, EPI_ISL_1085195, EPI_ISL_1085196, EPI_ISL_1085197, EPI_ISL_1085198, EPI_ISL_1085199, EPI_ISL_1085200, EPI_ISL_1085201, EPI_ISL_1085202, EPI_ISL_1085203, EPI_ISL_1085204, EPI_ISL_1085205, EPI_ISL_1085206, EPI_ISL_1085207, EPI_ISL_1085208, EPI_ISL_1085209, EPI_ISL_1085210, EPI_ISL_1085211, EPI_ISL_1085212, EPI_ISL_1085213, EPI_ISL_1085214, EPI_ISL_1085215, EPI_ISL_1085216, EPI_ISL_1085217, EPI_ISL_1085218, EPI_ISL_1085219, EPI_ISL_1085220, EPI_ISL_1085221, EPI_ISL_1085222, EPI_ISL_1085223, EPI_ISL_1085224, EPI_ISL_1085225, EPI_ISL_1085226, EPI_ISL_1085227, EPI_ISL_1085228, EPI_ISL_1085229			
see above	ASL Napoli 1 Centro	AMES Centro Polidiagnostico Strumentale S.r.l.	"Giovanni Savarese, Raffaella Ruggiero, Eloisa Evangelista, Antonella Di Carlo, Luisa Circelli, Luigi D'Amore, Roberto Sirica, Nadia Petrillo, Monica Ianniello, Antonio Fico"
EPI_ISL_1085230	Laboratory of Infectious Diseases, Department of Biomedical and Clinical Sciences L. Sacco, University of Milan	Laboratory of Infectious Diseases, Department of Biomedical and Clinical Sciences L. Sacco, University of Milan	Alessia Lai, Annalisa Bergna, Carla Della Ventura, Claudia Balotta, Massimo Galli, Gianguglielmo Zehender on behalf of SARS-CoV-2 ITALIAN RESEARCH ENTERPRISE-(SCIRE) Collaborative Group
EPI_ISL_1087353, EPI_ISL_1087354, EPI_ISL_1087355, EPI_ISL_1087356, EPI_ISL_1087357, EPI_ISL_1087358, EPI_ISL_1087359, EPI_ISL_1087360, EPI_ISL_1087361, EPI_ISL_1087362, EPI_ISL_1087363, EPI_ISL_1087364, EPI_ISL_1087365, EPI_ISL_1087366, EPI_ISL_1087367, EPI_ISL_1087368, EPI_ISL_1087369, EPI_ISL_1087370, EPI_ISL_1087371, EPI_ISL_1087372, EPI_ISL_1087373, EPI_ISL_1087374, EPI_ISL_1087375, EPI_ISL_1087376, EPI_ISL_1087377, EPI_ISL_1087378, EPI_ISL_1087379, EPI_ISL_1087380, EPI_ISL_1087381, EPI_ISL_1087382, EPI_ISL_1087383, EPI_ISL_1087384, EPI_ISL_1087385, EPI_ISL_1087386, EPI_ISL_1087387, EPI_ISL_1087388, EPI_ISL_1087389, EPI_ISL_1087390, EPI_ISL_1087391, EPI_ISL_1087392, EPI_ISL_1087393, EPI_ISL_1087394, EPI_ISL_1087395, EPI_ISL_1087396, EPI_ISL_1087397, EPI_ISL_1087398, EPI_ISL_1087399, EPI_ISL_1087400, EPI_ISL_1087401, EPI_ISL_1087402, EPI_ISL_1087403, EPI_ISL_1087404, EPI_ISL_1087405, EPI_ISL_1087406, EPI_ISL_1087407, EPI_ISL_1087408, EPI_ISL_1087409, EPI_ISL_1087410, EPI_ISL_1087411, EPI_ISL_1087412, EPI_ISL_1087413, EPI_ISL_1088008, EPI_ISL_1088009, EPI_ISL_1088010, EPI_ISL_1088011, EPI_ISL_1088012, EPI_ISL_1088013, EPI_ISL_1088014, EPI_ISL_1088015, EPI_ISL_1088016, EPI_ISL_1088017, EPI_ISL_1088018, EPI_ISL_1088019, EPI_ISL_1088020, EPI_ISL_1088021, EPI_ISL_1088022, EPI_ISL_1088023, EPI_ISL_1088024, EPI_ISL_1088025, EPI_ISL_1088026, EPI_ISL_1088027, EPI_ISL_1088028, EPI_ISL_1088029, EPI_ISL_1088030, EPI_ISL_1088031, EPI_ISL_1088032, EPI_ISL_1088033, EPI_ISL_1088034, EPI_ISL_1088035, EPI_ISL_1088036, EPI_ISL_1088037, EPI_ISL_1088038, EPI_ISL_1088039, EPI_ISL_1088040, EPI_ISL_1088041, EPI_ISL_1088042, EPI_ISL_1088043, EPI_ISL_1088044, EPI_ISL_1088045, EPI_ISL_1088046, EPI_ISL_1088047, EPI_ISL_1088048, EPI_ISL_1088049, EPI_ISL_1088050, EPI_ISL_1088051, EPI_ISL_1088052, EPI_ISL_1088053, EPI_ISL_1088054, EPI_ISL_1088055, EPI_ISL_1088056, EPI_ISL_1088057, EPI_ISL_1088058, EPI_ISL_1088059, EPI_ISL_1088060, EPI_ISL_1088061, EPI_ISL_1088062, EPI_ISL_1088063, EPI_ISL_1088064, EPI_ISL_1088065, EPI_ISL_1088066, EPI_ISL_1088067, EPI_ISL_1088068, EPI_ISL_1088069, EPI_ISL_1088070, EPI_ISL_1088071, EPI_ISL_1088072, EPI_ISL_1088073, EPI_ISL_1088074, EPI_ISL_1088075, EPI_ISL_1088076			
see above	ASL Napoli 1 Centro	AMES Centro Polidiagnostico Strumentale S.r.l.	"Giovanni Savarese, Raffaella Ruggiero, Eloisa Evangelista, Antonella Di Carlo, Luisa Circelli, Luigi D'Amore, Roberto Sirica, Nadia Petrillo, Monica Ianniello, Antonio Fico"
EPI_ISL_1091275	Ospedale S.M. Goretti di Latina - ASL Latina presidio ospedaliero Nord	INMI Lazzaro Spallanzani IRCCS	CEM Gruber, B Bartolini, E Giombini, F Messina, O Butera, M Rueca, S Pignalosa, L Corso, MR Capobianchi, A Di Caro
EPI_ISL_1091276	INMI Lazzaro Spallanzani IRCCS	INMI Lazzaro Spallanzani IRCCS	O Butera, E Giombini, F Messina, M Rueca, CEM Gruber, B Bartolini, MR Capobianchi, A Di Caro
EPI_ISL_1091277	INMI Lazzaro Spallanzani IRCCS	INMI Lazzaro Spallanzani IRCCS	CEM Gruber, B Bartolini, O Butera, E Giombini, F Messina, M Rueca, A Di Caro, MR Capobianchi
EPI_ISL_1091278	INMI Lazzaro Spallanzani IRCCS	INMI Lazzaro Spallanzani IRCCS	B Bartolini, E Giombini, F Messina, O Butera, M Rueca, CEM Gruber, A Di Caro, MR Capobianchi
EPI_ISL_1091279	INMI Lazzaro Spallanzani IRCCS	INMI Lazzaro Spallanzani IRCCS	M Rueca, CEM Gruber, B Bartolini, O Butera, E Giombini, F Messina, A Di Caro, MR Capobianchi
EPI_ISL_1091280	Azienda Ospedaliera San Camillo Forlanini	INMI Lazzaro Spallanzani IRCCS	F Messina, M Rueca, O Butera, CEM Gruber, B Bartolini, E Giombini, G Parisi, D Gallone, MR Capobianchi, A Di Caro
EPI_ISL_1091281	Azienda Ospedaliera San Camillo Forlanini	INMI Lazzaro Spallanzani IRCCS	M Rueca, CEM Gruber, B Bartolini, O Butera, E Giombini, F Messina, G Parisi, A D'Agostino, A Di Caro, MR Capobianchi
EPI_ISL_1091282	Azienda Ospedaliera San Camillo Forlanini	INMI Lazzaro Spallanzani IRCCS	O Butera, E Giombini, F Messina, M Rueca, CEM Gruber, B Bartolini, G Parisi, ML Guarino, MR Capobianchi, A Di Caro
EPI_ISL_1091283	Azienda Ospedaliera San Camillo Forlanini	INMI Lazzaro Spallanzani IRCCS	E Giombini, F Messina, M Rueca, O Butera, CEM Gruber, B Bartolini, G Parisi, MI Consalvo, A Di Caro, MR Capobianchi
EPI_ISL_1091284	Azienda Ospedaliera San Camillo Forlanini	INMI Lazzaro Spallanzani IRCCS	B Bartolini, E Giombini, F Messina, O Butera, M Rueca, CEM Gruber, G Parisi, F Basile, MR Capobianchi, A Di Caro
EPI_ISL_1091285	Azienda Ospedaliera San Camillo Forlanini	INMI Lazzaro Spallanzani IRCCS	F Messina, M Rueca, O Butera, CEM Gruber, B Bartolini, E Giombini, G Parisi, G Meoni, A Di Caro, MR Capobianchi
EPI_ISL_1091286	Azienda Ospedaliera San Camillo Forlanini	INMI Lazzaro Spallanzani IRCCS	CEM Gruber, B Bartolini, E Giombini, F Messina, O Butera, M Rueca, G Parisi, ML Guarino, A Di Caro, MR Capobianchi
EPI_ISL_1091287	Ospedale Sandro Pertini ASL Roma2	INMI Lazzaro Spallanzani IRCCS	CEM Gruber, B Bartolini, O Butera, E Giombini, F Messina, M Rueca, MC Cava, R Longo, A Di Caro, MR Capobianchi
EPI_ISL_1091288	Ospedale Sandro Pertini ASL Roma3	INMI Lazzaro Spallanzani IRCCS	B Bartolini, E Giombini, F Messina, O Butera, M Rueca, CEM Gruber, G Cappiello, V Michela, MR Capobianchi, A Di Caro
EPI_ISL_1091289	Ospedale Sandro Pertini ASL Roma4	INMI Lazzaro Spallanzani IRCCS	O Butera, E Giombini, F Messina, M Rueca, CEM Gruber, B Bartolini, MC Cava, S Romano, MR Capobianchi, A Di Caro
EPI_ISL_1091290	Ospedale San Filippo Neri	INMI Lazzaro Spallanzani IRCCS	M Rueca, CEM Gruber, B Bartolini, O Butera, E Giombini, F Messina, ML Schiavone, A Tamburro, MR Capobianchi, A Di Caro
EPI_ISL_1091291	Ospedale San Filippo Neri	INMI Lazzaro Spallanzani IRCCS	F Messina, M Rueca, O Butera, CEM Gruber, B Bartolini, E Giombini, A Tamburro, M Meledandri, MR Capobianchi, A Di Caro
EPI_ISL_1091787	San Gallicano Dermatological Institute I.F.O.	INMI Lazzaro Spallanzani IRCCS	B Bartolini, E Giombini, F Messina, M Rueca, O Butera, CEM Gruber, F Pimpinelli, F Ensoli, G Prignano, A Di Caro, MR Capobianchi
EPI_ISL_1091788	INMI Lazzaro Spallanzani IRCCS	INMI Lazzaro Spallanzani IRCCS	E Giombini, F Messina, M Rueca, O Butera, CEM Gruber, B Bartolini, A Di Caro, MR Capobianchi
EPI_ISL_1091789	INMI Lazzaro Spallanzani IRCCS	INMI Lazzaro Spallanzani IRCCS	O Butera, CEM Gruber, B Bartolini, E Giombini, F Messina, M Rueca, MR Capobianchi, A Di Caro

EPI_ISL_1091790	San Gallicano Dermatological Institute I.F.O.	INMI Lazzaro Spallanzani IRCCS	M Rueca, O Butera, CEM Gruber, B Bartolini, E Giombini, F Messina, F Pimpinelli, F Ensoli, G D'Agosto, A Di Caro, MR Capobianchi
EPI_ISL_1091791	Laboratorio Genzano - ASL RM 6	INMI Lazzaro Spallanzani IRCCS	O Butera, CEM Gruber, B Bartolini, E Giombini, F Messina, M Rueca, G Tramini, E Conti, MR Capobianchi, A Di Caro
EPI_ISL_1091792	Laboratorio Genzano - ASL RM 6	INMI Lazzaro Spallanzani IRCCS	B Bartolini, E Giombini, F Messina, M Rueca, O Butera, CEM Gruber, G Tramini, E Conti, A Di Caro, MR Capobianchi
EPI_ISL_1091793	Laboratorio Genzano - ASL RM 6	INMI Lazzaro Spallanzani IRCCS	CEM Gruber, B Bartolini, E Giombini, F Messina, M Rueca, O Butera, G Tramini, E Conti, MR Capobianchi, A Di Caro
EPI_ISL_1091794	Ospedale "F. Spaziani" Frosinone	INMI Lazzaro Spallanzani IRCCS	F Messina, M Rueca, O Butera, CEM Gruber, B Bartolini, E Giombini, R Pulselli, C Sias, G Brocco, MR Capobianchi, A Di Caro
EPI_ISL_1091795	Laboratorio Genzano - ASL RM 6	INMI Lazzaro Spallanzani IRCCS	M Rueca, O Butera, CEM Gruber, B Bartolini, E Giombini, F Messina, G Tramini, E Conti, A Di Caro, MR Capobianchi
EPI_ISL_1091796	Ospedale S.M. Goretti di Latina - ASL Latina presidio ospedaliero Nord	INMI Lazzaro Spallanzani IRCCS	B Bartolini, E Giombini, F Messina, M Rueca, O Butera, CEM Gruber, A Lucci, S Pignalosa, L Corso, A Di Caro, MR Capobianchi
EPI_ISL_1091797	Azienda Ospedaliera San Camillo Forlanini	INMI Lazzaro Spallanzani IRCCS	M Rueca, O Butera, CEM Gruber, B Bartolini, E Giombini, F Messina, G Parisi, D Gallone, F Basile, A Di Caro, MR Capobianchi
EPI_ISL_1091798	Synlab Lazio S.r.l.	INMI Lazzaro Spallanzani IRCCS	E Giombini, F Messina, M Rueca, O Butera, CEM Gruber, B Bartolini, E Trappolini, SA Santini, A Di Caro, MR Capobianchi
EPI_ISL_1093477, EPI_ISL_1093478, EPI_ISL_1093479, EPI_ISL_1093480, EPI_ISL_1093481, EPI_ISL_1093482, EPI_ISL_1093483, EPI_ISL_1093484, EPI_ISL_1093485, EPI_ISL_1093486, EPI_ISL_1093487, EPI_ISL_1093488, EPI_ISL_1093489, EPI_ISL_1093490, EPI_ISL_1093491			
see above	Center of Advanced Studies and Technology, Molecular Genetics Laboratory	Center of Advanced Studies and Technology, Molecular Genetics Laboratory	Ferrante Rossella, Mandatori Domitilla, De Fabritiis Simone, Damiani Verena, Anaclerio Federico
EPI_ISL_1095366, EPI_ISL_1095367, EPI_ISL_1095368, EPI_ISL_1095369, EPI_ISL_1095370, EPI_ISL_1095371, EPI_ISL_1095372, EPI_ISL_1095373, EPI_ISL_1095374, EPI_ISL_1095375, EPI_ISL_1095376, EPI_ISL_1095377, EPI_ISL_1095378, EPI_ISL_1095379, EPI_ISL_1095380, EPI_ISL_1095381, EPI_ISL_1095382, EPI_ISL_1095383			
see above	Istituto Zooprofilattico Sperimentale del Mezzogiorno	Lab. Microbiologia e Virologia, Cotugno, A.O. dei Colli	Luigi Atripaldi, Claudia Tiberio, Anna Perfetti, Pellegrino Cerino, Biancamaria Pierri, Maria Concetta Cuomo
EPI_ISL_1095384	Laboratorio Microbiologia e Virologia P.O. Cotugno A.O. dei Colli	Laboratorio Microbiologia e Virologia P.O. Cotugno A.O. dei Colli	Luigi Atripaldi, Claudia Tiberio, Anna Perfetti, Pellegrino Cerino, Biancamaria Pierri, Maria Concetta Cuomo
EPI_ISL_1095385, EPI_ISL_1095386, EPI_ISL_1095387, EPI_ISL_1095388, EPI_ISL_1095389, EPI_ISL_1095390, EPI_ISL_1095391, EPI_ISL_1095392, EPI_ISL_1095393, EPI_ISL_1095394, EPI_ISL_1095395, EPI_ISL_1095396, EPI_ISL_1095397, EPI_ISL_1095398, EPI_ISL_1095399, EPI_ISL_1095400, EPI_ISL_1095401, EPI_ISL_1095402, EPI_ISL_1095403, EPI_ISL_1095404, EPI_ISL_1095405, EPI_ISL_1095406, EPI_ISL_1095407			
see above	Istituto Zooprofilattico Sperimentale del Mezzogiorno	Lab. Microbiologia e Virologia, Cotugno, A.O. dei Colli	Luigi Atripaldi, Claudia Tiberio, Anna Perfetti, Pellegrino Cerino, Biancamaria Pierri, Maria Concetta Cuomo
EPI_ISL_1095449, EPI_ISL_1096228, EPI_ISL_1096229, EPI_ISL_1096230, EPI_ISL_1096231, EPI_ISL_1096232, EPI_ISL_1096233, EPI_ISL_1096342	Laboratorio Microbiologia e Virologia P.O. Cotugno A.O. dei Colli	Laboratorio Microbiologia e Virologia P.O. Cotugno A.O. dei Colli	Luigi Atripaldi, Claudia Tiberio, Anna Perfetti, Pellegrino Cerino, Biancamaria Pierri, Maria Concetta Cuomo
EPI_ISL_1096349, EPI_ISL_1096350, EPI_ISL_1096351, EPI_ISL_1096352, EPI_ISL_1096353, EPI_ISL_1096354, EPI_ISL_1096355, EPI_ISL_1096356, EPI_ISL_1096357, EPI_ISL_1096358, EPI_ISL_1096359, EPI_ISL_1096360, EPI_ISL_1096361, EPI_ISL_1096362, EPI_ISL_1096363, EPI_ISL_1096364, EPI_ISL_1096365, EPI_ISL_1096366, EPI_ISL_1096367, EPI_ISL_1096368, EPI_ISL_1096369, EPI_ISL_1096370, EPI_ISL_1096371, EPI_ISL_1096372, EPI_ISL_1096373, EPI_ISL_1096374, EPI_ISL_1096375, EPI_ISL_1096376, EPI_ISL_1096377, EPI_ISL_1096378, EPI_ISL_1096379, EPI_ISL_1096380, EPI_ISL_1096381, EPI_ISL_1096382, EPI_ISL_1096383, EPI_ISL_1096384, EPI_ISL_1096385, EPI_ISL_1096386, EPI_ISL_1096387, EPI_ISL_1096388, EPI_ISL_1096389, EPI_ISL_1096390, EPI_ISL_1096391, EPI_ISL_1096392, EPI_ISL_1096393, EPI_ISL_1096394, EPI_ISL_1096395, EPI_ISL_1096396, EPI_ISL_1096397, EPI_ISL_1096398, EPI_ISL_1096399, EPI_ISL_1096400, EPI_ISL_1096401, EPI_ISL_1096402, EPI_ISL_1096403, EPI_ISL_1096404, EPI_ISL_1096405, EPI_ISL_1096406, EPI_ISL_1096407, EPI_ISL_1096408, EPI_ISL_1096409, EPI_ISL_1096410, EPI_ISL_1096411, EPI_ISL_1096412, EPI_ISL_1096413, EPI_ISL_1096414, EPI_ISL_1096415, EPI_ISL_1096416, EPI_ISL_1096417, EPI_ISL_1096418, EPI_ISL_1096419, EPI_ISL_1096420, EPI_ISL_1096421, EPI_ISL_1096422, EPI_ISL_1096423, EPI_ISL_1096424, EPI_ISL_1096425, EPI_ISL_1096426, EPI_ISL_1096427, EPI_ISL_1096428, EPI_ISL_1096429, EPI_ISL_1096430, EPI_ISL_1096431, EPI_ISL_1096432, EPI_ISL_1096433, EPI_ISL_1096434, EPI_ISL_1096435, EPI_ISL_1096436, EPI_ISL_1096437, EPI_ISL_1096438, EPI_ISL_1096439, EPI_ISL_1096440, EPI_ISL_1096441, EPI_ISL_1096442, EPI_ISL_1096443, EPI_ISL_1096444, EPI_ISL_1096445, EPI_ISL_1096446, EPI_ISL_1096447, EPI_ISL_1096448, EPI_ISL_1096449, EPI_ISL_1096450, EPI_ISL_1096451, EPI_ISL_1096452, EPI_ISL_1096453, EPI_ISL_1096454, EPI_ISL_1096455, EPI_ISL_1096456, EPI_ISL_1096457, EPI_ISL_1096458, EPI_ISL_1096459, EPI_ISL_1096460, EPI_ISL_1096461, EPI_ISL_1096462, EPI_ISL_1096463, EPI_ISL_1096464, EPI_ISL_1096465, EPI_ISL_1096466, EPI_ISL_1096467, EPI_ISL_1096468, EPI_ISL_1096469, EPI_ISL_1096470, EPI_ISL_1096471, EPI_ISL_1096472, EPI_ISL_1096473, EPI_ISL_1096474, EPI_ISL_1096475, EPI_ISL_1096476, EPI_ISL_1096477, EPI_ISL_1096478, EPI_ISL_1096479, EPI_ISL_1096480, EPI_ISL_1096481, EPI_ISL_1096482, EPI_ISL_1096483, EPI_ISL_1096484, EPI_ISL_1096485, EPI_ISL_1096486, EPI_ISL_1096487, EPI_ISL_1096488, EPI_ISL_1096489, EPI_ISL_1096490, EPI_ISL_1096491, EPI_ISL_1096492, EPI_ISL_1096493, EPI_ISL_1096494, EPI_ISL_1096495, EPI_ISL_1096496, EPI_ISL_1096497, EPI_ISL_1096498, EPI_ISL_1096499, EPI_ISL_1096500, EPI_ISL_1096501, EPI_ISL_1096502, EPI_ISL_1096503, EPI_ISL_1096504, EPI_ISL_1096505, EPI_ISL_1096506, EPI_ISL_1096507, EPI_ISL_1096508, EPI_ISL_1096509, EPI_ISL_1096510, EPI_ISL_1096511, EPI_ISL_1096512, EPI_ISL_1096513, EPI_ISL_1096514, EPI_ISL_1096515, EPI_ISL_1096516, EPI_ISL_1096517, EPI_ISL_1096518, EPI_ISL_1096519, EPI_ISL_1096520, EPI_ISL_1096521, EPI_ISL_1096522, EPI_ISL_1096523, EPI_ISL_1096524, EPI_ISL_1096525, EPI_ISL_1096526, EPI_ISL_1096527, EPI_ISL_1096528, EPI_ISL_1096529, EPI_ISL_1096530, EPI_ISL_1096531, EPI_ISL_1096532, EPI_ISL_1096533, EPI_ISL_1096534, EPI_ISL_1096535, EPI_ISL_1096536, EPI_ISL_1096537, EPI_ISL_1096538, EPI_ISL_1096539, EPI_ISL_1096540, EPI_ISL_1096541, EPI_ISL_1096542, EPI_ISL_1096543, EPI_ISL_1096544, EPI_ISL_1096545, EPI_ISL_1096546, EPI_ISL_1096547, EPI_ISL_1096548, EPI_ISL_1096549, EPI_ISL_1096550, EPI_ISL_1096551, EPI_ISL_1096552, EPI_ISL_1096553, EPI_ISL_1096554, EPI_ISL_1096555, EPI_ISL_1096556, EPI_ISL_1096557, EPI_ISL_1096558, EPI_ISL_1096559, EPI_ISL_1096560, EPI_ISL_1096561, EPI_ISL_1096562, EPI_ISL_1096563, EPI_ISL_1096564, EPI_ISL_1096565, EPI_ISL_1096566, EPI_ISL_1096567, EPI_ISL_1096568, EPI_ISL_1096569, EPI_ISL_1096570, EPI_ISL_1096571, EPI_ISL_1096572, EPI_ISL_1096573, EPI_ISL_1096574, EPI_ISL_1096575, EPI_ISL_1096576, EPI_ISL_1096577, EPI_ISL_1096578, EPI_ISL_1096579, EPI_ISL_1096580, EPI_ISL_1096581, EPI_ISL_1096582, EPI_ISL_1096583, EPI_ISL_1096584, EPI_ISL_1096585, EPI_ISL_1096586, EPI_ISL_1096587, EPI_ISL_1096588, EPI_ISL_1096589, EPI_ISL_1096590, EPI_ISL_1096591, EPI_ISL_1096592, EPI_ISL_1096593, EPI_ISL_1096594, EPI_ISL_1096595, EPI_ISL_1096596, EPI_ISL_1096597, EPI_ISL_1096598, EPI_ISL_1096599, EPI_ISL_1096600, EPI_ISL_1096601, EPI_ISL_1096602, EPI_ISL_1096603, EPI_ISL_1096604, EPI_ISL_1096605, EPI_ISL_1096606, EPI_ISL_1096607, EPI_ISL_1096608, EPI_ISL_1096609, EPI_ISL_1096610, EPI_ISL_1096611, EPI_ISL_1096612, EPI_ISL_1096613, EPI_ISL_1096614, EPI_ISL_1096615, EPI_ISL_1096616, EPI_ISL_1096617, EPI_ISL_1096618, EPI_ISL_1096619, EPI_ISL_1096620, EPI_ISL_1096621, EPI_ISL_1096622, EPI_ISL_1096623, EPI_ISL_1096624, EPI_ISL_1096625, EPI_ISL_1096626, EPI_ISL_1096627, EPI_ISL_1096628, EPI_ISL_1096629, EPI_ISL_1096630, EPI_ISL_1096631, EPI_ISL_1096632, EPI_ISL_1096633, EPI_ISL_1096634, EPI_ISL_1096635, EPI_ISL_1096636, EPI_ISL_1096637, EPI_ISL_1096638, EPI_ISL_1096639, EPI_ISL_1096640, EPI_ISL_1096641, EPI_ISL_1096642, EPI_ISL_1096643, EPI_ISL_1096644, EPI_ISL_1096645, EPI_ISL_1096646, EPI_ISL_1096647, EPI_ISL_1096648, EPI_ISL_1096649, EPI_ISL_1096650, EPI_ISL_1096651, EPI_ISL_1096652, EPI_ISL_1096653, EPI_ISL_1096654, EPI_ISL_1096655, EPI_ISL_1096656, EPI_ISL_1096657, EPI_ISL_1096658, EPI_ISL_1096659, EPI_ISL_1096660, EPI_ISL_1096661, EPI_ISL_1096662, EPI_ISL_1096663, EPI_ISL_1096664, EPI_ISL_1096665, EPI_ISL_1096666, EPI_ISL_1096667, EPI_ISL_1096668, EPI_ISL_1096669, EPI_ISL_1096670, EPI_ISL_1096671, EPI_ISL_1096672, EPI_ISL_1096673, EPI_ISL_1096674, EPI_ISL_1096675, EPI_ISL_1096676, EPI_ISL_1096677, EPI_ISL_1096678, EPI_ISL_1096679, EPI_ISL_1096680, EPI_ISL_1096681, EPI_ISL_1096682, EPI_ISL_1096683, EPI_ISL_1096684, EPI_ISL_1096685, EPI_ISL_1096686, EPI_ISL_1096687, EPI_ISL_1096688, EPI_ISL_1096689, EPI_ISL_1096690, EPI_ISL_1096691, EPI_ISL_1096692, EPI_ISL_1096693, EPI_ISL_1096694, EPI_ISL_1096695, EPI_ISL_1096696, EPI_ISL_1096697, EPI_ISL_1096698, EPI_ISL_1096699, EPI_ISL_1096700, EPI_ISL_1096701, EPI_ISL_1096702, EPI_ISL_1096703, EPI_ISL_1096704, EPI_ISL_1096705, EPI_ISL_1096706, EPI_ISL_1096707, EPI_ISL_1096708, EPI_ISL_1096709, EPI_ISL_1096710, EPI_ISL_1096711, EPI_ISL_1096712, EPI_ISL_1096713, EPI_ISL_1096714, EPI_ISL_1096715, EPI_ISL_1096716, EPI_ISL_1096717, EPI_ISL_1096718, EPI_ISL_1096719, EPI_ISL_1096720, EPI_ISL_1096721, EPI_ISL_1096722, EPI_ISL_1096723, EPI_ISL_1096724, EPI_ISL_1096725, EPI_ISL_1096726, EPI_ISL_1096727, EPI_ISL_1096728, EPI_ISL_1096729, EPI_ISL_1096730, EPI_ISL_1096731, EPI_ISL_1096732, EPI_ISL_1096733, EPI_ISL_1096734, EPI_ISL_1096735, EPI_ISL_1096736, EPI_ISL_1096737, EPI_ISL_1096738, EPI_ISL_1096739, EPI_ISL_1096740, EPI_ISL_1096741, EPI_ISL_1096742, EPI_ISL_1096743, EPI_ISL_1096744, EPI_ISL_1096745, EPI_ISL_1096746, EPI_ISL_1096747, EPI_ISL_1096748, EPI_ISL_1096749, EPI_ISL_1096750, EPI_ISL_1096751, EPI_ISL_1096752, EPI_ISL_1096753, EPI_ISL_1096754, EPI_ISL_1096755, EPI_ISL_1096756, EPI_ISL_1096757, EPI_ISL_1096758, EPI_ISL_1096759, EPI_ISL_1096760, EPI_ISL_1096761, EPI_ISL_1096762, EPI_ISL_1096763, EPI_ISL_1096764, EPI_ISL_1096765, EPI_ISL_1096766, EPI_ISL_1096767, EPI_ISL_1096768, EPI_ISL_1096769, EPI_ISL_1096770, EPI_ISL_1096771, EPI_ISL_1096772, EPI_ISL_1096773, EPI_ISL_1096774, EPI_ISL_1096775, EPI_ISL_1096776, EPI_ISL_1096777, EPI_ISL_1096778, EPI_ISL_1096779, EPI_ISL_1096780, EPI_ISL_1096781, EPI_ISL_1096782, EPI_ISL_1096783, EPI_ISL_1096784, EPI_ISL_1096785, EPI_ISL_1096786, EPI_ISL_1096787, EPI_ISL_1096788, EPI_ISL_1096789, EPI_ISL_1096790, EPI_ISL_1096791, EPI_ISL_1096792, EPI_ISL_1096793, EPI_ISL_1096794, EPI_ISL_1096795, EPI_ISL_1096796, EPI_ISL_1096797, EPI_ISL_1096798, EPI_ISL_1096799, EPI_ISL_1096800, EPI_ISL_1096801, EPI_ISL_1096802, EPI_ISL_1096803, EPI_ISL_1096804, EPI_ISL_1096805, EPI_ISL_1096806, EPI_ISL_1096807, EPI_ISL_1096808, EPI_ISL_1096809, EPI_ISL_1096810, EPI_ISL_1096811, EPI_ISL_1096812, EPI_ISL_1096813, EPI_ISL_1096814, EPI_ISL_1096815, EPI_ISL_1096816, EPI_ISL_1096817, EPI_ISL_1096818, EPI_ISL_1096819, EPI_ISL_1096820, EPI_ISL_1096821, EPI_ISL_1096822, EPI_ISL_1096823, EPI_ISL_1096824, EPI_ISL_1096825, EPI_ISL_1096826, EPI_ISL_1096827, EPI_ISL_1096828, EPI_ISL_1096829, EPI_ISL_1096830, EPI_ISL_1096831, EPI_ISL_1096832, EPI_ISL_1096833, EPI_ISL_1096834, EPI_ISL_1096835, EPI_ISL_1096836, EPI_ISL_1096837, EPI_ISL_1096838, EPI_ISL_1096839, EPI_ISL_1096840, EPI_ISL_1096841, EPI_ISL_1096842, EPI_ISL_1096843, EPI_ISL_1096844, EPI_ISL_1096845, EPI_ISL_1096846, EPI_ISL_1096847, EPI_ISL_1096848, EPI_ISL_1096849, EPI_ISL_1096850, EPI_ISL_1096851, EPI_ISL_1096852, EPI_ISL_1096853, EPI_ISL_1096854, EPI_ISL_1096855, EPI_ISL_1096856, EPI_ISL_1096857, EPI_ISL_1096858, EPI_ISL_1096859, EPI_ISL_1096860, EPI_ISL_1096861, EPI_ISL_1096862, EPI_ISL_1096863, EPI_ISL_1096864, EPI_ISL_1096865, EPI_ISL_1096866, EPI_ISL_1096867, EPI_ISL_1096868, EPI_ISL_1096869, EPI_ISL_1096870, EPI_ISL_1096871, EPI_ISL_1096872, EPI_ISL_1096873, EPI_ISL_1096874, EPI_ISL_1096875, EPI_ISL_1096876, EPI_ISL_1096877, EPI_ISL_1096878, EPI_ISL_1096879, EPI_ISL_1096880, EPI_ISL_1096881, EPI_ISL_1096882, EPI_ISL_1096883, EPI_ISL_1096884, EPI_ISL_1096885, EPI_ISL_1096886, EPI_ISL_1096887, EPI_ISL_1096888, EPI_ISL_1096889, EPI_ISL_1096890, EPI_ISL_1096891, EPI_ISL_1096892, EPI_ISL_1096893, EPI_ISL_1096894, EPI_ISL_1096895, EPI_ISL_1096896, EPI_ISL_1096897, EPI_ISL_1096898, EPI_ISL_1096899, EPI_ISL_1096900, EPI_ISL_1096901, EPI_ISL_1096902, EPI_ISL_1096903, EPI_ISL_1096904, EPI_ISL_1096905, EPI_ISL_1096906, EPI_ISL_1096907, EPI_ISL_1096908, EPI_ISL_1096909, EPI_ISL_1096910, EPI_ISL_1096911, EPI_ISL_1096912, EPI_ISL_1096913, EPI_ISL_1096914, EPI_ISL_1096915, EPI_ISL_1096916, EPI_ISL_1096917, EPI_ISL_1096918, EPI_ISL_1096919, EPI_ISL_1096920, EPI_ISL_1096921, EPI_ISL_1096922, EPI_ISL_1096923, EPI_ISL_1096924, EPI_ISL_1096925, EPI_ISL_1096926, EPI_ISL_1096927, EPI_ISL_1096928, EPI_ISL_1096929, EPI_ISL_1096930, EPI_ISL_1096931, EPI_ISL_1096932, EPI_ISL_1096933, EPI_ISL_1096934, EPI_ISL_1096935, EPI_ISL_1096936, EPI_ISL_1096937, EPI_ISL_1096938, EPI_ISL_1096939, EPI_ISL_1096940, EPI_ISL_1096941, EPI_ISL_1096942, EPI_ISL_1096943, EPI_ISL_1096944, EPI_ISL_1096945, EPI_ISL_1096946, EPI_ISL_1096947, EPI_ISL_1096948, EPI_ISL_1096949, EPI_ISL_1096950, EPI_ISL_1096951, EPI_ISL_1096952, EPI_ISL_1096953, EPI_ISL_1096954, EPI_ISL_1096955, EPI_ISL_1096956, EPI_ISL_1096957, EPI_ISL_1096958, EPI_ISL_1096959, EPI_ISL_1096960, EPI_ISL_1096961, EPI_ISL_1096962, EPI_ISL_1096963, EPI_ISL_1096964, EPI_ISL_1096965, EPI_ISL_1096966, EPI_ISL_1096967, EPI_ISL_1096968, EPI_ISL_1096969, EPI_ISL_1096970, EPI_ISL_1096971, EPI_ISL_1096972, EPI_ISL_1096973, EPI_ISL_1096974, EPI_ISL_1096975, EPI_ISL_1096976, EPI_ISL_1096977, EPI_ISL_1096978, EPI_ISL_1096979, EPI_ISL_1096980, EPI_ISL_1096981			
see above	ASL Napoli 1 Centro	AMES Centro Polidiagnostico Strumentale S.r.l.	"Giovanni Savarese, Raffaella Ruggiero, Eloisa Evangelista, Antonella Di Carlo, Luisa Circelli, Luigi D'Amore, Roberto Sirica, Antonio Fico"
EPI_ISL_1096982, EPI_ISL_1096984, EPI_ISL_1096986, EPI_ISL_1096989, EPI_ISL_1096991, EPI_ISL_1096993, EPI_ISL_1096994, EPI_ISL_1096996, EPI_ISL_1096998, EPI_ISL_1097000	Laboratorio Microbiologia e Virologia P.O. Cotugno A.O. dei Colli	Laboratorio Microbiologia e Virologia P.O. Cotugno A.O. dei Colli	Luigi Atripaldi, Claudia Tiberio, Anna Perfetti, Pellegrino Cerino, Biancamaria Pierri, Maria Concetta Cuomo
EPI_ISL_1097002	Center of Advanced Studies and Technology, Molecular Genetics Laboratory	Center of Advanced Studies and Technology, Molecular Genetics Laboratory	Ferrante Rossella, Mandatori Domitilla, De Fabritiis Simone, Damiani Verena, Anaclerio Federico
EPI_ISL_1097004, EPI_ISL_1097005, EPI_ISL_1097007, EPI_ISL_1097008, EPI_ISL_1097009, EPI_ISL_1097010, EPI_ISL_1097011, EPI_ISL_1097012, EPI_ISL_1097013, EPI_ISL_1097014, EPI_ISL_1097015			
see above	Laboratorio Microbiologia e Virologia P.O. Cotugno A.O. dei Colli	Laboratorio Microbiologia e Virologia P.O. Cotugno A.O. dei Colli	Luigi Atripaldi, Claudia Tiberio, Anna Perfetti, Pellegrino Cerino, Biancamaria Pierri, Maria Concetta Cuomo
EPI_ISL_1097016	Laboratorio Microbiologia e Virologia P.O. Cotugno A.O. dei Col	Laboratorio Microbiologia e Virologia P.O. Cotugno A.O. dei Colli	Luigi Atripaldi, Claudia Tiberio, Anna Perfetti, Pellegrino Cerino, Biancamaria Pierri, Maria Concetta Cuomo

EPI_ISL_1097017, EPI_ISL_1097019, EPI_ISL_1097020, EPI_ISL_1097021, EPI_ISL_1097022	Laboratorio Microbiologia e Virologia P.O. Cotugno A.O. dei Colli	Laboratorio Microbiologia e Virologia P.O. Cotugno A.O. dei Colli	Luigi Atripaldi, Claudia Tiberio, Anna Perfetti, Pellegrino Cerino, Biancamaria Pierri, Maria Concetta Cuomo
EPI_ISL_1104645, EPI_ISL_1104647, EPI_ISL_1104649	Virologia, Dipartimento di Scienze Biomediche, Università di Sassari, Viale San Pietro, 43/B - Sassari	Laboratorio specialistico UOC Ematologia - Ospedale "San Francesco" - ATS-ASSL Nuoro	Giovanna Piras, Asproni Rosanna, Paolo Malune, Maria Itria Monne, Angelo Domenico Palmas, Caterina Serra, Elena Rimini, Salvatore Rubino
EPI_ISL_1104650	Laboratorio Biologia Molecolare Sars Cov2 - UOC Laboratorio Analisi - Servizio Medicina di Laboratorio, Ospedale "San Francesco" - ATS-ASSL Nuoro	Laboratorio specialistico UOC Ematologia - Ospedale "San Francesco" - ATS-ASSL Nuoro	Giovanna Piras, Rosanna Asproni, Paolo Malune, Maura Fiamma, Maria Itria Monne, Angelo Domenico Palmas, Iana Lo Maglio, Giuseppe Mameli
EPI_ISL_1104652, EPI_ISL_1104654, EPI_ISL_1104656	Virologia, Dipartimento di Scienze Biomediche, Università di Sassari, Viale San Pietro, 43/B - Sassari	Laboratorio specialistico UOC Ematologia - Ospedale "San Francesco" - ATS-ASSL Nuoro	Giovanna Piras, Asproni Rosanna, Paolo Malune, Maria Itria Monne, Angelo Domenico Palmas, Caterina Serra, Elena Rimini, Salvatore Rubino
EPI_ISL_1109574	SC (UCO) Igiene e Sanità Pubblica (funzione integrata con SC Microbiologia e Virologia)	ARGO Laboratorio Genomica ed Epigenomica	Licastro D, Dal Monego S, Degasperi M, Marcello A, D'Agaro P, Lombardo F
EPI_ISL_1109575	SC (UCO) Igiene e Sanità Pubblica (funzione integrata con SC Microbiologia e Virologia) e Azienda Ospedaliera Pordenone	ARGO Laboratorio Genomica ed Epigenomica	Licastro D, Dal Monego S, Degasperi M, Marcello A, D'Agaro P, De Rosa R
EPI_ISL_1109576	SC (UCO) Igiene e Sanità Pubblica (funzione integrata con SC Microbiologia e Virologia)	ARGO Laboratorio Genomica ed Epigenomica	Licastro D, Dal Monego S, Degasperi M, Marcello A, Segat L, Piscianz E, D'Agaro P
EPI_ISL_1109577	SC (UCO) Igiene e Sanità Pubblica (funzione integrata con SC Microbiologia e Virologia)	ARGO Laboratorio Genomica ed Epigenomica	Licastro D, Dal Monego S, Degasperi M, Marcello A, D'Agaro P, Lombardo F
EPI_ISL_1109578, EPI_ISL_1109579, EPI_ISL_1109580, EPI_ISL_1109581	SC (UCO) Igiene e Sanità Pubblica (funzione integrata con SC Microbiologia e Virologia) e Az. Ospedaliero-Universitaria Udine	ARGO Laboratorio Genomica ed Epigenomica	Licastro D, Dal Monego S, Degasperi M, Marcello A, D'Agaro P, Pipan C
EPI_ISL_1109582	SC (UCO) Igiene e Sanità Pubblica (funzione integrata con SC Microbiologia e Virologia)	ARGO Laboratorio Genomica ed Epigenomica	Licastro D, Dal Monego S, Degasperi M, Marcello A, D'Agaro P, Lombardo F
EPI_ISL_1109583, EPI_ISL_1109584, EPI_ISL_1109585, EPI_ISL_1109586	SC (UCO) Igiene e Sanità Pubblica (funzione integrata con SC Microbiologia e Virologia) e Az. Ospedaliero-Universitaria Udine	ARGO Laboratorio Genomica ed Epigenomica	Licastro D, Dal Monego S, Degasperi M, Marcello A, D'Agaro P, Pipan C
EPI_ISL_1109587	SC (UCO) Igiene e Sanità Pubblica (funzione integrata con SC Microbiologia e Virologia) e Azienda Ospedaliera Pordenone	ARGO Laboratorio Genomica ed Epigenomica	Licastro D, Dal Monego S, Degasperi M, Marcello A, D'Agaro P, De Rosa R
EPI_ISL_1109588	SC (UCO) Igiene e Sanità Pubblica (funzione integrata con SC Microbiologia e Virologia)	ARGO Laboratorio Genomica ed Epigenomica	Licastro D, Dal Monego S, Degasperi M, Marcello A, D'Agaro P, Lombardo F
EPI_ISL_1109589, EPI_ISL_1109590	SC (UCO) Igiene e Sanità Pubblica (funzione integrata con SC Microbiologia e Virologia) e Az. Ospedaliero-Universitaria Udine	ARGO Laboratorio Genomica ed Epigenomica	Licastro D, Dal Monego S, Degasperi M, Marcello A, D'Agaro P, Pipan C
EPI_ISL_1109591	SC (UCO) Igiene e Sanità Pubblica (funzione integrata con SC Microbiologia e Virologia)	ARGO Laboratorio Genomica ed Epigenomica	Licastro D, Dal Monego S, Degasperi M, Marcello A, D'Agaro P, Lombardo F
EPI_ISL_1109592, EPI_ISL_1109593	SC (UCO) Igiene e Sanità Pubblica (funzione integrata con SC Microbiologia e Virologia)	ARGO Laboratorio Genomica ed Epigenomica	Licastro D, Dal Monego S, Degasperi M, Marcello A, Segat L, Piscianz E, D'Agaro P
EPI_ISL_1109594	SC (UCO) Igiene e Sanità Pubblica (funzione integrata con SC Microbiologia e Virologia) e Azienda Ospedaliera Pordenone	ARGO Laboratorio Genomica ed Epigenomica	Licastro D, Dal Monego S, Degasperi M, Marcello A, D'Agaro P, De Rosa R
EPI_ISL_1109595, EPI_ISL_1109596, EPI_ISL_1109597, EPI_ISL_1109598, EPI_ISL_1109599	SC (UCO) Igiene e Sanità Pubblica (funzione integrata con SC Microbiologia e Virologia) e Az. Ospedaliero-Universitaria Udine	ARGO Laboratorio Genomica ed Epigenomica	Licastro D, Dal Monego S, Degasperi M, Marcello A, D'Agaro P, Pipan C
EPI_ISL_1109600	SC (UCO) Igiene e Sanità Pubblica (funzione integrata con SC Microbiologia e Virologia)	ARGO Laboratorio Genomica ed Epigenomica	Licastro D, Dal Monego S, Degasperi M, Marcello A, D'Agaro P, Lombardo F
EPI_ISL_1109601, EPI_ISL_1109602, EPI_ISL_1109603, EPI_ISL_1109604, EPI_ISL_1109605, EPI_ISL_1109606, EPI_ISL_1109607	SC (UCO) Igiene e Sanità Pubblica (funzione integrata con SC Microbiologia e Virologia) e Az. Ospedaliero-Universitaria Udine	ARGO Laboratorio Genomica ed Epigenomica	Licastro D, Dal Monego S, Degasperi M, Marcello A, D'Agaro P, Pipan C
EPI_ISL_1109608, EPI_ISL_1109609	SC (UCO) Igiene e Sanità Pubblica (funzione integrata con SC Microbiologia e Virologia)	ARGO Laboratorio Genomica ed Epigenomica	Licastro D, Dal Monego S, Degasperi M, Marcello A, Segat L, Piscianz E, D'Agaro P
EPI_ISL_1109610, EPI_ISL_1109611, EPI_ISL_1109612	SC (UCO) Igiene e Sanità Pubblica (funzione integrata con SC Microbiologia e Virologia) e Azienda Ospedaliera Pordenone	ARGO Laboratorio Genomica ed Epigenomica	Licastro D, Dal Monego S, Degasperi M, Marcello A, D'Agaro P, De Rosa R
EPI_ISL_1109613	SC (UCO) Igiene e Sanità Pubblica (funzione integrata con SC Microbiologia e Virologia) e Az. Ospedaliero-Universitaria Udine	ARGO Laboratorio Genomica ed Epigenomica	Licastro D, Dal Monego S, Degasperi M, Marcello A, D'Agaro P, Pipan C
EPI_ISL_1109614, EPI_ISL_1109615	SC (UCO) Igiene e Sanità Pubblica (funzione integrata con SC Microbiologia e Virologia) e Azienda Ospedaliera Pordenone	ARGO Laboratorio Genomica ed Epigenomica	Licastro D, Dal Monego S, Degasperi M, Marcello A, D'Agaro P, De Rosa R
EPI_ISL_1109616, EPI_ISL_1109617, EPI_ISL_1109618, EPI_ISL_1109619	SC (UCO) Igiene e Sanità Pubblica (funzione integrata con SC Microbiologia e Virologia) e Az. Ospedaliero-Universitaria Udine	ARGO Laboratorio Genomica ed Epigenomica	Licastro D, Dal Monego S, Degasperi M, Marcello A, D'Agaro P, Pipan C
EPI_ISL_1109620	SC (UCO) Igiene e Sanità Pubblica (funzione integrata con SC Microbiologia e Virologia)	ARGO Laboratorio Genomica ed Epigenomica	Licastro D, Dal Monego S, Degasperi M, Marcello A, Segat L, Piscianz E, D'Agaro P
EPI_ISL_1109621	SC (UCO) Igiene e Sanità Pubblica (funzione integrata con SC Microbiologia e Virologia) e Az. Ospedaliero-Universitaria Udine	ARGO Laboratorio Genomica ed Epigenomica	Licastro D, Dal Monego S, Degasperi M, Marcello A, D'Agaro P, Pipan C
EPI_ISL_1110295, EPI_ISL_1110296, EPI_ISL_1110297, EPI_ISL_1110298, EPI_ISL_1110299, EPI_ISL_1110300, EPI_ISL_1110301, EPI_ISL_1110302, EPI_ISL_1110303, EPI_ISL_1110304, EPI_ISL_1110305, EPI_ISL_1110306, EPI_ISL_1110307, EPI_ISL_1110308, EPI_ISL_1110309, EPI_ISL_1110310, EPI_ISL_1110311, EPI_ISL_1110312, EPI_ISL_1110313, EPI_ISL_1110314, EPI_ISL_1110315, EPI_ISL_1110316, EPI_ISL_1110317, EPI_ISL_1110318, EPI_ISL_1110319, EPI_ISL_1110320, EPI_ISL_1110321, EPI_ISL_1110322, EPI_ISL_1110323, EPI_ISL_1110324, EPI_ISL_1110325, EPI_ISL_1110326, EPI_ISL_1110327, EPI_ISL_1110328, EPI_ISL_1110329, EPI_ISL_1110330, EPI_ISL_1110331, EPI_ISL_1110332, EPI_ISL_1110333, EPI_ISL_1110334, EPI_ISL_1110335, EPI_ISL_1110336, EPI_ISL_1110337, EPI_ISL_1110338, EPI_ISL_1110339, EPI_ISL_1110340, EPI_ISL_1110341, EPI_ISL_1110342, EPI_ISL_1110343, EPI_ISL_1110344, EPI_ISL_1110345, EPI_ISL_1110346, EPI_ISL_1110347, EPI_ISL_1110348, EPI_ISL_1110349, EPI_ISL_1110350, EPI_ISL_1110351, EPI_ISL_1110352, EPI_ISL_1110353, EPI_ISL_1110354, EPI_ISL_1110355, EPI_ISL_1110356, EPI_ISL_1110357, EPI_ISL_1110358, EPI_ISL_1110359, EPI_ISL_1110360, EPI_ISL_1110361, EPI_ISL_1110362, EPI_ISL_1110363, EPI_ISL_1110364, EPI_ISL_1110365, EPI_ISL_1110366, EPI_ISL_1110367, EPI_ISL_1110368, EPI_ISL_1110369, EPI_ISL_1110370, EPI_ISL_1110371, EPI_ISL_1110372, EPI_ISL_1110373, EPI_ISL_1110374, EPI_ISL_1110375, EPI_ISL_1110376, EPI_ISL_1110377, EPI_ISL_1110378, EPI_ISL_1110379, EPI_ISL_1110380, EPI_ISL_1110381, EPI_ISL_1110382, EPI_ISL_1110383, EPI_ISL_1110384, EPI_ISL_1110385, EPI_ISL_1110386, EPI_ISL_1110387, EPI_ISL_1110388			
see above	Azienda Sanitaria dell'Alto Adige Laboratorio Aziendale di Microbiologia e Virologia	Istituto di Genomica Applicata	Elisabetta Pagani, Irene Bianconi, Elisabetta Giacobazzi, Elisa Masi, Stefanie Wieser, Irena Jurman, Vera Vendramin, Gabriele Magris, Eleonora Paparelli, Davide Scaglione, Michele Morgante

EPI_ISL_1110581, EPI_ISL_1110582, EPI_ISL_1110583, EPI_ISL_1110584, EPI_ISL_1110585, EPI_ISL_1110586, EPI_ISL_1110587, EPI_ISL_1110588, EPI_ISL_1110589, EPI_ISL_1110590, EPI_ISL_1110591, EPI_ISL_1110592, EPI_ISL_1110593, EPI_ISL_1110594, EPI_ISL_1110595			
see above	LAB ANALISI PO CARDARELLI CB	Istituto Zooprofilattico Sperimentale dell'Abruzzo e Molise "G. Caporale"	Scutellà M, Niro G, Lorusso A, Marcacci M, Di Domenico M, Ancora M, Curini V, Mangone I, Rinaldi A, Scialabba S, Di Pasquale A, Cammà C, Puglia I, Calistri P, Savini G
EPI_ISL_1110596	SIESP L'Aquila	Istituto Zooprofilattico Sperimentale dell'Abruzzo e Molise "G. Caporale"	Lorusso A, Marcacci M, Di Domenico M, Ancora M, Curini V, Mangone I, Rinaldi A, Scialabba S, Di Pasquale A, Cammà C, Puglia I, Calistri P, Savini G
EPI_ISL_1110597	SIESP CHIETI-Drive in Chieti	Istituto Zooprofilattico Sperimentale dell'Abruzzo e Molise "G. Caporale"	Lorusso A, Marcacci M, Di Domenico M, Ancora M, Curini V, Mangone I, Rinaldi A, Scialabba S, Di Pasquale A, Cammà C, Puglia I, Calistri P, Savini G
EPI_ISL_1110598, EPI_ISL_1110599, EPI_ISL_1110600	SIESP CH-Drive in VASTO	Istituto Zooprofilattico Sperimentale dell'Abruzzo e Molise "G. Caporale"	Lorusso A, Marcacci M, Di Domenico M, Ancora M, Curini V, Mangone I, Rinaldi A, Scialabba S, Di Pasquale A, Cammà C, Puglia I, Calistri P, Savini G
EPI_ISL_1110601	Ospedale Civile Teramo	Istituto Zooprofilattico Sperimentale dell'Abruzzo e Molise "G. Caporale"	Lorusso A, Marcacci M, Di Domenico M, Ancora M, Curini V, Mangone I, Rinaldi A, Scialabba S, Di Pasquale A, Cammà C, Puglia I, Calistri P, Savini G
EPI_ISL_1110602	SIESP Dip. Prev Teramo	Istituto Zooprofilattico Sperimentale dell'Abruzzo e Molise "G. Caporale"	Lorusso A, Marcacci M, Di Domenico M, Ancora M, Curini V, Mangone I, Rinaldi A, Scialabba S, Di Pasquale A, Cammà C, Puglia I, Calistri P, Savini G
EPI_ISL_1112221, EPI_ISL_1112222	Ospedale Santissima Annunziata	Istituto Zooprofilattico Sperimentale della Puglia e della Basilicata	Parisi A., Bianco A., Capozzi L., Del Sambro L., Simone D., Giannico A., Ridolfi D.
EPI_ISL_1112223, EPI_ISL_1112224, EPI_ISL_1112225, EPI_ISL_1112226	Ospedale Di Venere - Carbonara	Istituto Zooprofilattico Sperimentale della Puglia e della Basilicata	Parisi A., Bianco A., Capozzi L., Del Sambro L., Simone D., Giannico A., Ridolfi D.
EPI_ISL_1112227, EPI_ISL_1112228, EPI_ISL_1112229, EPI_ISL_1112230, EPI_ISL_1112231, EPI_ISL_1112232, EPI_ISL_1112233, EPI_ISL_1112234	Istituto Zooprofilattico Sperimentale della Puglia e della Basilicata	Istituto Zooprofilattico Sperimentale della Puglia e della Basilicata	Parisi A., Bianco A., Capozzi L., Del Sambro L., Simone D., Giannico A., Ridolfi D.
EPI_ISL_1112235	Ospedali Riuniti Azienda Ospedaliera Universitaria - Foggia	Istituto Zooprofilattico Sperimentale della Puglia e della Basilicata	Parisi A., Bianco A., Capozzi L., Del Sambro L., Simone D., Giannico A., Ridolfi D.
EPI_ISL_1112236	Dipartimento di Scienze Biomediche e Oncologia Umana - Azienda Ospedaliero Universitaria Consorziale Policlinico	Istituto Zooprofilattico Sperimentale della Puglia e della Basilicata	Parisi A., Bianco A., Capozzi L., Del Sambro L., Simone D., Giannico A., Ridolfi D.
EPI_ISL_1112237, EPI_ISL_1112238, EPI_ISL_1112239, EPI_ISL_1112240, EPI_ISL_1112241, EPI_ISL_1112242, EPI_ISL_1112243, EPI_ISL_1112244, EPI_ISL_1112245, EPI_ISL_1112246, EPI_ISL_1112247, EPI_ISL_1112248, EPI_ISL_1112249, EPI_ISL_1112250, EPI_ISL_1112251, EPI_ISL_1112252, EPI_ISL_1112253, EPI_ISL_1112254, EPI_ISL_1112255, EPI_ISL_1112256, EPI_ISL_1112257, EPI_ISL_1112258, EPI_ISL_1112259, EPI_ISL_1112260, EPI_ISL_1112261, EPI_ISL_1112262, EPI_ISL_1112263, EPI_ISL_1112264, EPI_ISL_1112265, EPI_ISL_1112266, EPI_ISL_1112267, EPI_ISL_1112268, EPI_ISL_1112269, EPI_ISL_1112270, EPI_ISL_1112271, EPI_ISL_1112272, EPI_ISL_1112273, EPI_ISL_1112274, EPI_ISL_1112275, EPI_ISL_1112276, EPI_ISL_1112277, EPI_ISL_1112278, EPI_ISL_1112279, EPI_ISL_1112280, EPI_ISL_1112281, EPI_ISL_1112282, EPI_ISL_1112283, EPI_ISL_1112284, EPI_ISL_1112285, EPI_ISL_1112286, EPI_ISL_1112287, EPI_ISL_1112288, EPI_ISL_1112289, EPI_ISL_1112290, EPI_ISL_1112291			
see above	Dipartimento di Scienze Biomediche e Oncologia Umana - Azienda Ospedaliero Universitaria Consorziale Policlinico	Istituto Zooprofilattico Sperimentale della Puglia e della Basilicata	Parisi A., Bianco A., Capozzi L., Del Sambro L., Simone D., Chironna M., Loconsole D., Sallustio A.
EPI_ISL_1114766	SIESP L'Aquila	Istituto Zooprofilattico Sperimentale dell'Abruzzo e Molise "G. Caporale"	Lorusso A, Marcacci M, Di Domenico M, Ancora M, Curini V, Mangone I, Rinaldi A, Scialabba S, Di Pasquale A, Cammà C, Puglia I, Calistri P, Savini G
EPI_ISL_1114769	Virologia, Dipartimento di Scienze Biomediche, Università di Sassari, Viale San Pietro, 43/B - Sassari	Laboratorio specialistico UOC Ematologia - Ospedale "San Francesco" - ATS-ASSL Nuoro	Giovanna Piras, Rosanna Asproni, Paolo Malune, Maria Itria Monne, Angelo Domenico Palmas, Caterina Serra, Elena Rimini, Salvatore Rubino
EPI_ISL_1116471, EPI_ISL_1116472	Dipartimento di Scienze Biomediche e Oncologia Umana - Azienda Ospedaliero Universitaria Consorziale Policlinico	Istituto Zooprofilattico Sperimentale della Puglia e della Basilicata	Parisi A., Bianco A., Capozzi L., Del Sambro L., Simone D., Chironna M., Loconsole D., Sallustio A.
EPI_ISL_1116473	Istituto Zooprofilattico Sperimentale della Puglia e della Basilicata	Istituto Zooprofilattico Sperimentale della Puglia e della Basilicata	Parisi A., Bianco A., Capozzi L., Del Sambro L., Simone D., Chironna M., Loconsole D., Sallustio A., Ridolfi D.
EPI_ISL_1116474	Istituto Zooprofilattico Sperimentale della Puglia e della Basilicata	Istituto Zooprofilattico Sperimentale della Puglia e della Basilicata	Parisi A., Bianco A., Capozzi L., Del Sambro L., Simone D., Chironna M., Loconsole D., Sallustio A., Giannico A.
EPI_ISL_1116475	Dipartimento di Scienze Biomediche e Oncologia Umana - Azienda Ospedaliero Universitaria Consorziale Policlinico	Istituto Zooprofilattico Sperimentale della Puglia e della Basilicata	Parisi A., Bianco A., Capozzi L., Del Sambro L., Simone D., Chironna M., Loconsole D., Sallustio A.
EPI_ISL_1117458	OSP SAN SALVATORE, MEDICINA INTERNA	Istituto Zooprofilattico Sperimentale dell'Abruzzo e Molise "G. Caporale"	Lorusso A, Marcacci M, Di Domenico M, Ancora M, Curini V, Mangone I, Rinaldi A, Scialabba S, Di Pasquale A, Cammà C, Scialabba S, Puglia I, Calistri P, Savini G
EPI_ISL_1117459	SIESP L'AQUILA,	Istituto Zooprofilattico Sperimentale dell'Abruzzo e Molise "G. Caporale"	Lorusso A, Marcacci M, Di Domenico M, Ancora M, Curini V, Mangone I, Rinaldi A, Scialabba S, Di Pasquale A, Cammà C, Scialabba S, Puglia I, Calistri P, Savini G
EPI_ISL_1117460	SIESP DIP PREV CHIETI	Istituto Zooprofilattico Sperimentale dell'Abruzzo e Molise "G. Caporale"	Lorusso A, Marcacci M, Di Domenico M, Ancora M, Curini V, Mangone I, Rinaldi A, Scialabba S, Di Pasquale A, Cammà C, Scialabba S, Puglia I, Calistri P, Savini G
EPI_ISL_1117461	SIESP CHIETI,DRIVE IN CHIETI	Istituto Zooprofilattico Sperimentale dell'Abruzzo e Molise "G. Caporale"	Lorusso A, Marcacci M, Di Domenico M, Ancora M, Curini V, Mangone I, Rinaldi A, Scialabba S, Di Pasquale A, Cammà C, Scialabba S, Puglia I, Calistri P, Savini G
EPI_ISL_1117462	SIESP CHIETI, DRIVE IN CHIETI	Istituto Zooprofilattico Sperimentale dell'Abruzzo e Molise "G. Caporale"	Lorusso A, Marcacci M, Di Domenico M, Ancora M, Curini V, Mangone I, Rinaldi A, Scialabba S, Di Pasquale A, Cammà C, Scialabba S, Puglia I, Calistri P, Savini G
EPI_ISL_1117463	SIESP CHIETI, DRIVE IN LANCIANO	Istituto Zooprofilattico Sperimentale dell'Abruzzo e Molise "G. Caporale"	Lorusso A, Marcacci M, Di Domenico M, Ancora M, Curini V, Mangone I, Rinaldi A, Scialabba S, Di Pasquale A, Cammà C, Scialabba S, Puglia I, Calistri P, Savini G
EPI_ISL_1117464	SIESP CHIETI, DRIVE IN CHIETI	Istituto Zooprofilattico Sperimentale dell'Abruzzo e Molise "G. Caporale"	Lorusso A, Marcacci M, Di Domenico M, Ancora M, Curini V, Mangone I, Rinaldi A, Scialabba S, Di Pasquale A, Cammà C, Scialabba S, Puglia I, Calistri P, Savini G
EPI_ISL_1117465	SIESP DIP PREV CHIETI	Istituto Zooprofilattico Sperimentale dell'Abruzzo e Molise "G. Caporale"	Lorusso A, Marcacci M, Di Domenico M, Ancora M, Curini V, Mangone I, Rinaldi A, Scialabba S, Di Pasquale A, Cammà C, Scialabba S, Puglia I, Calistri P, Savini G
EPI_ISL_1117466, EPI_ISL_1117467	SIESP DIP PREV TERAMO	Istituto Zooprofilattico Sperimentale dell'Abruzzo e Molise "G. Caporale"	Lorusso A, Marcacci M, Di Domenico M, Ancora M, Curini V, Mangone I, Rinaldi A, Scialabba S, Di Pasquale A, Cammà C, Scialabba S, Puglia I, Calistri P, Savini G
EPI_ISL_1117468	OSP SAN SALVATORE, MEDICINA INTERNA	Istituto Zooprofilattico Sperimentale dell'Abruzzo e Molise "G. Caporale"	Lorusso A, Marcacci M, Di Domenico M, Ancora M, Curini V, Mangone I, Rinaldi A, Scialabba S, Di Pasquale A, Cammà C, Scialabba S, Puglia I, Calistri P, Savini G
EPI_ISL_1117469, EPI_ISL_1117470, EPI_ISL_1117471, EPI_ISL_1117472	SIESP DIP PREV TERAMO	Istituto Zooprofilattico Sperimentale dell'Abruzzo e Molise "G. Caporale"	Lorusso A, Marcacci M, Di Domenico M, Ancora M, Curini V, Mangone I, Rinaldi A, Scialabba S, Di Pasquale A, Cammà C, Scialabba S, Puglia I, Calistri P, Savini G
EPI_ISL_1117473	OSP SAN SALVATORE, MEDICINA INTERNA	Istituto Zooprofilattico Sperimentale dell'Abruzzo e Molise "G. Caporale"	Lorusso A, Marcacci M, Di Domenico M, Ancora M, Curini V, Mangone I, Rinaldi A, Scialabba S, Di Pasquale A, Cammà C, Scialabba S, Puglia I, Calistri P, Savini G
EPI_ISL_1117474	SIESP DIP PREV CHIETI	Istituto Zooprofilattico Sperimentale dell'Abruzzo e Molise "G. Caporale"	Lorusso A, Marcacci M, Di Domenico M, Ancora M, Curini V, Mangone I, Rinaldi A, Scialabba S, Di Pasquale A, Cammà C, Scialabba S, Puglia I, Calistri P, Savini G
EPI_ISL_1117475	SIESP SULMONA	Istituto Zooprofilattico Sperimentale dell'Abruzzo e Molise "G. Caporale"	Lorusso A, Marcacci M, Di Domenico M, Ancora M, Curini V, Mangone I, Rinaldi A, Scialabba S, Di Pasquale A, Cammà C, Scialabba S, Puglia I, Calistri P, Savini G

EPI_ISL_1117476, EPI_ISL_1117477	SIESP CHIETI, DRIVE IN CHIETI	Istituto Zooprofilattico Sperimentale dell'Abruzzo e Molise "G. Caporale"	Lorusso A, Marcacci M, Di Domenico M, Ancora M, Curini V, Mangone I, Rinaldi A, Scialabba S, Di Pasquale A, Cammà C, Scialabba S, Puglia I, Calistri P, Savini G
EPI_ISL_1117478	SIESP CHIETI, DRIVE IN ORTONA	Istituto Zooprofilattico Sperimentale dell'Abruzzo e Molise "G. Caporale"	Lorusso A, Marcacci M, Di Domenico M, Ancora M, Curini V, Mangone I, Rinaldi A, Scialabba S, Di Pasquale A, Cammà C, Scialabba S, Puglia I, Calistri P, Savini G
EPI_ISL_1117479	SIESP CHIETI, DRIVE IN LANCIANO	Istituto Zooprofilattico Sperimentale dell'Abruzzo e Molise "G. Caporale"	Lorusso A, Marcacci M, Di Domenico M, Ancora M, Curini V, Mangone I, Rinaldi A, Scialabba S, Di Pasquale A, Cammà C, Scialabba S, Puglia I, Calistri P, Savini G
EPI_ISL_1117480, EPI_ISL_1117481, EPI_ISL_1117482	SIESP DIP PREV TERAMO	Istituto Zooprofilattico Sperimentale dell'Abruzzo e Molise "G. Caporale"	Lorusso A, Marcacci M, Di Domenico M, Ancora M, Curini V, Mangone I, Rinaldi A, Scialabba S, Di Pasquale A, Cammà C, Scialabba S, Puglia I, Calistri P, Savini G
EPI_ISL_1117483, EPI_ISL_1117484	OSP SAN SALVATORE, MEDICINA INTERNA	Istituto Zooprofilattico Sperimentale dell'Abruzzo e Molise "G. Caporale"	Lorusso A, Marcacci M, Di Domenico M, Ancora M, Curini V, Mangone I, Rinaldi A, Scialabba S, Di Pasquale A, Cammà C, Scialabba S, Puglia I, Calistri P, Savini G
EPI_ISL_1117485	SIESP DIP PREV TERAMO	Istituto Zooprofilattico Sperimentale dell'Abruzzo e Molise "G. Caporale"	Lorusso A, Marcacci M, Di Domenico M, Ancora M, Curini V, Mangone I, Rinaldi A, Scialabba S, Di Pasquale A, Cammà C, Scialabba S, Puglia I, Calistri P, Savini G
EPI_ISL_1117486, EPI_ISL_1117487	SIESP DIP PREV CHIETI	Istituto Zooprofilattico Sperimentale dell'Abruzzo e Molise "G. Caporale"	Lorusso A, Marcacci M, Di Domenico M, Ancora M, Curini V, Mangone I, Rinaldi A, Scialabba S, Di Pasquale A, Cammà C, Scialabba S, Puglia I, Calistri P, Savini G
EPI_ISL_1117488	DIP PREV AVEZZANO, SERVIZIO DI IGIENE EPIDEMIOLOGIA E SANITÀ PUBBLICA	Istituto Zooprofilattico Sperimentale dell'Abruzzo e Molise "G. Caporale"	Lorusso A, Marcacci M, Di Domenico M, Ancora M, Curini V, Mangone I, Rinaldi A, Scialabba S, Di Pasquale A, Cammà C, Scialabba S, Puglia I, Calistri P, Savini G
EPI_ISL_1117489	SIESP DIP PREV TERAMO	Istituto Zooprofilattico Sperimentale dell'Abruzzo e Molise "G. Caporale"	Lorusso A, Marcacci M, Di Domenico M, Ancora M, Curini V, Mangone I, Rinaldi A, Scialabba S, Di Pasquale A, Cammà C, Scialabba S, Puglia I, Calistri P, Savini G
EPI_ISL_1117490	SIESP CHIETI, DRIVE IN VASTO	Istituto Zooprofilattico Sperimentale dell'Abruzzo e Molise "G. Caporale"	Lorusso A, Marcacci M, Di Domenico M, Ancora M, Curini V, Mangone I, Rinaldi A, Scialabba S, Di Pasquale A, Cammà C, Scialabba S, Puglia I, Calistri P, Savini G
EPI_ISL_1117491, EPI_ISL_1117492	SIESP DIP PREV CHIETI	Istituto Zooprofilattico Sperimentale dell'Abruzzo e Molise "G. Caporale"	Lorusso A, Marcacci M, Di Domenico M, Ancora M, Curini V, Mangone I, Rinaldi A, Scialabba S, Di Pasquale A, Cammà C, Scialabba S, Puglia I, Calistri P, Savini G
EPI_ISL_1117493	OSP SAN SALVATORE, MEDICINA INTERNA	Istituto Zooprofilattico Sperimentale dell'Abruzzo e Molise "G. Caporale"	Lorusso A, Marcacci M, Di Domenico M, Ancora M, Curini V, Mangone I, Rinaldi A, Scialabba S, Di Pasquale A, Cammà C, Scialabba S, Puglia I, Calistri P, Savini G
EPI_ISL_1117494	SIESP DIP PREV CHIETI	Istituto Zooprofilattico Sperimentale dell'Abruzzo e Molise "G. Caporale"	Lorusso A, Marcacci M, Di Domenico M, Ancora M, Curini V, Mangone I, Rinaldi A, Scialabba S, Di Pasquale A, Cammà C, Scialabba S, Puglia I, Calistri P, Savini G
EPI_ISL_1117495	SIESP DIP PREV TERAMO	Istituto Zooprofilattico Sperimentale dell'Abruzzo e Molise "G. Caporale"	Lorusso A, Marcacci M, Di Domenico M, Ancora M, Curini V, Mangone I, Rinaldi A, Scialabba S, Di Pasquale A, Cammà C, Scialabba S, Puglia I, Calistri P, Savini G
EPI_ISL_1117496	USCA PESCINA	Istituto Zooprofilattico Sperimentale dell'Abruzzo e Molise "G. Caporale"	Lorusso A, Marcacci M, Di Domenico M, Ancora M, Curini V, Mangone I, Rinaldi A, Scialabba S, Di Pasquale A, Cammà C, Scialabba S, Puglia I, Calistri P, Savini G
EPI_ISL_1117497	SIESP CHIETI, DRIVE IN ORTONA	Istituto Zooprofilattico Sperimentale dell'Abruzzo e Molise "G. Caporale"	Lorusso A, Marcacci M, Di Domenico M, Ancora M, Curini V, Mangone I, Rinaldi A, Scialabba S, Di Pasquale A, Cammà C, Scialabba S, Puglia I, Calistri P, Savini G
EPI_ISL_1117498	PRESIDIO TERRITORIALE CASOLI	Istituto Zooprofilattico Sperimentale dell'Abruzzo e Molise "G. Caporale"	Lorusso A, Marcacci M, Di Domenico M, Ancora M, Curini V, Mangone I, Rinaldi A, Scialabba S, Di Pasquale A, Cammà C, Scialabba S, Puglia I, Calistri P, Savini G
EPI_ISL_1117499	SIESP CHIETI, DRIVE IN VASTO	Istituto Zooprofilattico Sperimentale dell'Abruzzo e Molise "G. Caporale"	Lorusso A, Marcacci M, Di Domenico M, Ancora M, Curini V, Mangone I, Rinaldi A, Scialabba S, Di Pasquale A, Cammà C, Scialabba S, Puglia I, Calistri P, Savini G
EPI_ISL_1117500	SIESP CHIETI, DRIVE IN ORTONA	Istituto Zooprofilattico Sperimentale dell'Abruzzo e Molise "G. Caporale"	Lorusso A, Marcacci M, Di Domenico M, Ancora M, Curini V, Mangone I, Rinaldi A, Scialabba S, Di Pasquale A, Cammà C, Scialabba S, Puglia I, Calistri P, Savini G
EPI_ISL_1117501	OSP SAN SALVATORE, MEDICINA INTERNA	Istituto Zooprofilattico Sperimentale dell'Abruzzo e Molise "G. Caporale"	Lorusso A, Marcacci M, Di Domenico M, Ancora M, Curini V, Mangone I, Rinaldi A, Scialabba S, Di Pasquale A, Cammà C, Scialabba S, Puglia I, Calistri P, Savini G
EPI_ISL_1117502	SIESP DIP PREV CHIETI	Istituto Zooprofilattico Sperimentale dell'Abruzzo e Molise "G. Caporale"	Lorusso A, Marcacci M, Di Domenico M, Ancora M, Curini V, Mangone I, Rinaldi A, Scialabba S, Di Pasquale A, Cammà C, Scialabba S, Puglia I, Calistri P, Savini G
EPI_ISL_1117503	SIESP DIP PREV TERAMO	Istituto Zooprofilattico Sperimentale dell'Abruzzo e Molise "G. Caporale"	Lorusso A, Marcacci M, Di Domenico M, Ancora M, Curini V, Mangone I, Rinaldi A, Scialabba S, Di Pasquale A, Cammà C, Scialabba S, Puglia I, Calistri P, Savini G
EPI_ISL_1117504, EPI_ISL_1117505	OSP SAN SALVATORE, MEDICINA INTERNA	Istituto Zooprofilattico Sperimentale dell'Abruzzo e Molise "G. Caporale"	Lorusso A, Marcacci M, Di Domenico M, Ancora M, Curini V, Mangone I, Rinaldi A, Scialabba S, Di Pasquale A, Cammà C, Scialabba S, Puglia I, Calistri P, Savini G
EPI_ISL_1117506	SIESP DIP PREV CHIETI	Istituto Zooprofilattico Sperimentale dell'Abruzzo e Molise "G. Caporale"	Lorusso A, Marcacci M, Di Domenico M, Ancora M, Curini V, Mangone I, Rinaldi A, Scialabba S, Di Pasquale A, Cammà C, Scialabba S, Puglia I, Calistri P, Savini G
EPI_ISL_1117507, EPI_ISL_1117508	SIESP CHIETI, DRIVE IN LANCIANO	Istituto Zooprofilattico Sperimentale dell'Abruzzo e Molise "G. Caporale"	Lorusso A, Marcacci M, Di Domenico M, Ancora M, Curini V, Mangone I, Rinaldi A, Scialabba S, Di Pasquale A, Cammà C, Scialabba S, Puglia I, Calistri P, Savini G
EPI_ISL_1117509, EPI_ISL_1117510, EPI_ISL_1117511, EPI_ISL_1117512, EPI_ISL_1117513	SIESP DIP PREV CHIETI	Istituto Zooprofilattico Sperimentale dell'Abruzzo e Molise "G. Caporale"	Lorusso A, Marcacci M, Di Domenico M, Ancora M, Curini V, Mangone I, Rinaldi A, Scialabba S, Di Pasquale A, Cammà C, Scialabba S, Puglia I, Calistri P, Savini G
EPI_ISL_1117514	PRES OSP SAN LIBERATORE	Istituto Zooprofilattico Sperimentale dell'Abruzzo e Molise "G. Caporale"	Lorusso A, Marcacci M, Di Domenico M, Ancora M, Curini V, Mangone I, Rinaldi A, Scialabba S, Di Pasquale A, Cammà C, Scialabba S, Puglia I, Calistri P, Savini G
EPI_ISL_1117515	SIESP DIP PREV TERAMO	Istituto Zooprofilattico Sperimentale dell'Abruzzo e Molise "G. Caporale"	Lorusso A, Marcacci M, Di Domenico M, Ancora M, Curini V, Mangone I, Rinaldi A, Scialabba S, Di Pasquale A, Cammà C, Scialabba S, Puglia I, Calistri P, Savini G
EPI_ISL_1117516	SIESP CHIETI, DRIVE IN LANCIANO	Istituto Zooprofilattico Sperimentale dell'Abruzzo e Molise "G. Caporale"	Lorusso A, Marcacci M, Di Domenico M, Ancora M, Curini V, Mangone I, Rinaldi A, Scialabba S, Di Pasquale A, Cammà C, Scialabba S, Puglia I, Calistri P, Savini G
EPI_ISL_1117517	SIESP CHIETI, DRIVE IN ORTONA	Istituto Zooprofilattico Sperimentale dell'Abruzzo e Molise "G. Caporale"	Lorusso A, Marcacci M, Di Domenico M, Ancora M, Curini V, Mangone I, Rinaldi A, Scialabba S, Di Pasquale A, Cammà C, Scialabba S, Puglia I, Calistri P, Savini G
EPI_ISL_1117518	SIESP DIP PREV TERAMO	Istituto Zooprofilattico Sperimentale dell'Abruzzo e Molise "G. Caporale"	Lorusso A, Marcacci M, Di Domenico M, Ancora M, Curini V, Mangone I, Rinaldi A, Scialabba S, Di Pasquale A, Cammà C, Scialabba S, Puglia I, Calistri P, Savini G
EPI_ISL_1117519	SIESP CHIETI, DRIVE IN CHIETI	Istituto Zooprofilattico Sperimentale dell'Abruzzo e Molise "G. Caporale"	Lorusso A, Marcacci M, Di Domenico M, Ancora M, Curini V, Mangone I, Rinaldi A, Scialabba S, Di Pasquale A, Cammà C, Scialabba S, Puglia I, Calistri P, Savini G
EPI_ISL_1117520	USCA AVEZZANO	Istituto Zooprofilattico Sperimentale dell'Abruzzo e Molise "G. Caporale"	Lorusso A, Marcacci M, Di Domenico M, Ancora M, Curini V, Mangone I, Rinaldi A, Scialabba S, Di Pasquale A, Cammà C, Scialabba S, Puglia I, Calistri P, Savini G
EPI_ISL_1117521	DIP PREV AVEZZANO, SERVIZIO DI IGIENE EPIDEMIOLOGIA E SANITÀ PUBBLICA	Istituto Zooprofilattico Sperimentale dell'Abruzzo e Molise "G. Caporale"	Lorusso A, Marcacci M, Di Domenico M, Ancora M, Curini V, Mangone I, Rinaldi A, Scialabba S, Di Pasquale A, Cammà C, Scialabba S, Puglia I, Calistri P, Savini G
EPI_ISL_1117522, EPI_ISL_1117523, EPI_ISL_1117524	SIESP DIP PREV TERAMO	Istituto Zooprofilattico Sperimentale dell'Abruzzo e Molise "G. Caporale"	Lorusso A, Marcacci M, Di Domenico M, Ancora M, Curini V, Mangone I, Rinaldi A, Scialabba S, Di Pasquale A, Cammà C, Scialabba S, Puglia I, Calistri P, Savini G

EPI_ISL_1117525	OSP SAN SALVATORE, MEDICINA INTERNA	Istituto Zooprofilattico Sperimentale dell'Abruzzo e Molise "G. Caporale"	Lorusso A, Marcacci M, Di Domenico M, Ancora M, Curini V, Mangone I, Rinaldi A, Scialabba S, Di Pasquale A, Cammà C, Scialabba S, Puglia I, Calistri P, Savini G
EPI_ISL_1117526	IZS TERAMO	Istituto Zooprofilattico Sperimentale dell'Abruzzo e Molise "G. Caporale"	Lorusso A, Marcacci M, Di Domenico M, Ancora M, Curini V, Mangone I, Rinaldi A, Scialabba S, Di Pasquale A, Cammà C, Scialabba S, Puglia I, Calistri P, Savini G
EPI_ISL_1117661, EPI_ISL_1117662	SIESP DIP PREV TERAMO	Istituto Zooprofilattico Sperimentale dell'Abruzzo e Molise "G. Caporale"	Lorusso A, Marcacci M, Di Domenico M, Ancora M, Curini V, Mangone I, Rinaldi A, Scialabba S, Di Pasquale A, Cammà C, Puglia I, Calistri P, Savini G
EPI_ISL_1117663	SIESP CHIETI, DRIVE IN ORTONA	Istituto Zooprofilattico Sperimentale dell'Abruzzo e Molise "G. Caporale"	Lorusso A, Marcacci M, Di Domenico M, Ancora M, Curini V, Mangone I, Rinaldi A, Scialabba S, Di Pasquale A, Cammà C, Puglia I, Calistri P, Savini G
EPI_ISL_1117664	SIESP DIP PREV CHIETI	Istituto Zooprofilattico Sperimentale dell'Abruzzo e Molise "G. Caporale"	Lorusso A, Marcacci M, Di Domenico M, Ancora M, Curini V, Mangone I, Rinaldi A, Scialabba S, Di Pasquale A, Cammà C, Puglia I, Calistri P, Savini G
EPI_ISL_1117665, EPI_ISL_1117666	SIESP DIP PREV TERAMO	Istituto Zooprofilattico Sperimentale dell'Abruzzo e Molise "G. Caporale"	Lorusso A, Marcacci M, Di Domenico M, Ancora M, Curini V, Mangone I, Rinaldi A, Scialabba S, Di Pasquale A, Cammà C, Puglia I, Calistri P, Savini G
EPI_ISL_1117667	SIESP CHIETI, DRIVE IN ORTONA	Istituto Zooprofilattico Sperimentale dell'Abruzzo e Molise "G. Caporale"	Lorusso A, Marcacci M, Di Domenico M, Ancora M, Curini V, Mangone I, Rinaldi A, Scialabba S, Di Pasquale A, Cammà C, Puglia I, Calistri P, Savini G
EPI_ISL_1117668	SIESP DIP PREV TERAMO	Istituto Zooprofilattico Sperimentale dell'Abruzzo e Molise "G. Caporale"	Lorusso A, Marcacci M, Di Domenico M, Ancora M, Curini V, Mangone I, Rinaldi A, Scialabba S, Di Pasquale A, Cammà C, Puglia I, Calistri P, Savini G
EPI_ISL_1117669	SIESP CHIETI, DRIVE IN CHIETI	Istituto Zooprofilattico Sperimentale dell'Abruzzo e Molise "G. Caporale"	Lorusso A, Marcacci M, Di Domenico M, Ancora M, Curini V, Mangone I, Rinaldi A, Scialabba S, Di Pasquale A, Cammà C, Puglia I, Calistri P, Savini G
EPI_ISL_1117670, EPI_ISL_1117671	SIESP DIP PREV TERAMO	Istituto Zooprofilattico Sperimentale dell'Abruzzo e Molise "G. Caporale"	Lorusso A, Marcacci M, Di Domenico M, Ancora M, Curini V, Mangone I, Rinaldi A, Scialabba S, Di Pasquale A, Cammà C, Puglia I, Calistri P, Savini G
EPI_ISL_1117672	SIESP DIP PREV CHIETI	Istituto Zooprofilattico Sperimentale dell'Abruzzo e Molise "G. Caporale"	Lorusso A, Marcacci M, Di Domenico M, Ancora M, Curini V, Mangone I, Rinaldi A, Scialabba S, Di Pasquale A, Cammà C, Puglia I, Calistri P, Savini G
EPI_ISL_1117673	SIESP DIP PREV TERAMO	Istituto Zooprofilattico Sperimentale dell'Abruzzo e Molise "G. Caporale"	Lorusso A, Marcacci M, Di Domenico M, Ancora M, Curini V, Mangone I, Rinaldi A, Scialabba S, Di Pasquale A, Cammà C, Puglia I, Calistri P, Savini G
EPI_ISL_1117674	SIESP CHIETI, DRIVE IN CHIETI	Istituto Zooprofilattico Sperimentale dell'Abruzzo e Molise "G. Caporale"	Lorusso A, Marcacci M, Di Domenico M, Ancora M, Curini V, Mangone I, Rinaldi A, Scialabba S, Di Pasquale A, Cammà C, Puglia I, Calistri P, Savini G
EPI_ISL_1117675	DIP PREV AVEZZANO-SERVIZIO DI IGIENE EPIDEMIOLOGIA E SANITÀ PUBBLICA	Istituto Zooprofilattico Sperimentale dell'Abruzzo e Molise "G. Caporale"	Lorusso A, Marcacci M, Di Domenico M, Ancora M, Curini V, Mangone I, Rinaldi A, Scialabba S, Di Pasquale A, Cammà C, Puglia I, Calistri P, Savini G
EPI_ISL_1117676	SIESP CHIETI, DRIVE IN LANCIANO	Istituto Zooprofilattico Sperimentale dell'Abruzzo e Molise "G. Caporale"	Lorusso A, Marcacci M, Di Domenico M, Ancora M, Curini V, Mangone I, Rinaldi A, Scialabba S, Di Pasquale A, Cammà C, Puglia I, Calistri P, Savini G
EPI_ISL_1117677	USCA CASTEL DI SANGRO	Istituto Zooprofilattico Sperimentale dell'Abruzzo e Molise "G. Caporale"	Lorusso A, Marcacci M, Di Domenico M, Ancora M, Curini V, Mangone I, Rinaldi A, Scialabba S, Di Pasquale A, Cammà C, Puglia I, Calistri P, Savini G
EPI_ISL_1117678, EPI_ISL_1117679	SIESP CHIETI, DRIVE IN VASTO	Istituto Zooprofilattico Sperimentale dell'Abruzzo e Molise "G. Caporale"	Lorusso A, Marcacci M, Di Domenico M, Ancora M, Curini V, Mangone I, Rinaldi A, Scialabba S, Di Pasquale A, Cammà C, Puglia I, Calistri P, Savini G
EPI_ISL_1117680	SIESP DIP PREV TERAMO	Istituto Zooprofilattico Sperimentale dell'Abruzzo e Molise "G. Caporale"	Lorusso A, Marcacci M, Di Domenico M, Ancora M, Curini V, Mangone I, Rinaldi A, Scialabba S, Di Pasquale A, Cammà C, Puglia I, Calistri P, Savini G
EPI_ISL_1117681	SIESP CHIETI, DRIVE IN CHIETI	Istituto Zooprofilattico Sperimentale dell'Abruzzo e Molise "G. Caporale"	Lorusso A, Marcacci M, Di Domenico M, Ancora M, Curini V, Mangone I, Rinaldi A, Scialabba S, Di Pasquale A, Cammà C, Puglia I, Calistri P, Savini G
EPI_ISL_1117682	SIESP CHIETI, DRIVE IN LANCIANO	Istituto Zooprofilattico Sperimentale dell'Abruzzo e Molise "G. Caporale"	Lorusso A, Marcacci M, Di Domenico M, Ancora M, Curini V, Mangone I, Rinaldi A, Scialabba S, Di Pasquale A, Cammà C, Puglia I, Calistri P, Savini G
EPI_ISL_1117683, EPI_ISL_1117684	USCA CASTEL DI SANGRO	Istituto Zooprofilattico Sperimentale dell'Abruzzo e Molise "G. Caporale"	Lorusso A, Marcacci M, Di Domenico M, Ancora M, Curini V, Mangone I, Rinaldi A, Scialabba S, Di Pasquale A, Cammà C, Puglia I, Calistri P, Savini G
EPI_ISL_1117685	SIESP DIP PREV TERAMO	Istituto Zooprofilattico Sperimentale dell'Abruzzo e Molise "G. Caporale"	Lorusso A, Marcacci M, Di Domenico M, Ancora M, Curini V, Mangone I, Rinaldi A, Scialabba S, Di Pasquale A, Cammà C, Puglia I, Calistri P, Savini G
EPI_ISL_1117686	DIP PREV AVEZZANO-SERVIZIO DI IGIENE EPIDEMIOLOGIA E SANITÀ PUBBLICA	Istituto Zooprofilattico Sperimentale dell'Abruzzo e Molise "G. Caporale"	Lorusso A, Marcacci M, Di Domenico M, Ancora M, Curini V, Mangone I, Rinaldi A, Scialabba S, Di Pasquale A, Cammà C, Puglia I, Calistri P, Savini G
EPI_ISL_1117687	SIESP CHIETI, DRIVE IN ORTONA	Istituto Zooprofilattico Sperimentale dell'Abruzzo e Molise "G. Caporale"	Lorusso A, Marcacci M, Di Domenico M, Ancora M, Curini V, Mangone I, Rinaldi A, Scialabba S, Di Pasquale A, Cammà C, Puglia I, Calistri P, Savini G
EPI_ISL_1117688	SIESP DIP PREV CHIETI	Istituto Zooprofilattico Sperimentale dell'Abruzzo e Molise "G. Caporale"	Lorusso A, Marcacci M, Di Domenico M, Ancora M, Curini V, Mangone I, Rinaldi A, Scialabba S, Di Pasquale A, Cammà C, Puglia I, Calistri P, Savini G
EPI_ISL_1117689	SIESP CHIETI, DRIVE IN LANCIANO	Istituto Zooprofilattico Sperimentale dell'Abruzzo e Molise "G. Caporale"	Lorusso A, Marcacci M, Di Domenico M, Ancora M, Curini V, Mangone I, Rinaldi A, Scialabba S, Di Pasquale A, Cammà C, Puglia I, Calistri P, Savini G
EPI_ISL_1117690	DIP PREV AVEZZANO-SERVIZIO DI IGIENE EPIDEMIOLOGIA E SANITÀ PUBBLICA	Istituto Zooprofilattico Sperimentale dell'Abruzzo e Molise "G. Caporale"	Lorusso A, Marcacci M, Di Domenico M, Ancora M, Curini V, Mangone I, Rinaldi A, Scialabba S, Di Pasquale A, Cammà C, Puglia I, Calistri P, Savini G
EPI_ISL_1117691	SIESP DIP PREV TERAMO	Istituto Zooprofilattico Sperimentale dell'Abruzzo e Molise "G. Caporale"	Lorusso A, Marcacci M, Di Domenico M, Ancora M, Curini V, Mangone I, Rinaldi A, Scialabba S, Di Pasquale A, Cammà C, Puglia I, Calistri P, Savini G
EPI_ISL_1117692	PRESIDIO OSP SAN LIBERATORE	Istituto Zooprofilattico Sperimentale dell'Abruzzo e Molise "G. Caporale"	Lorusso A, Marcacci M, Di Domenico M, Ancora M, Curini V, Mangone I, Rinaldi A, Scialabba S, Di Pasquale A, Cammà C, Puglia I, Calistri P, Savini G
EPI_ISL_1117693	SIESP DIP PREV TERAMO	Istituto Zooprofilattico Sperimentale dell'Abruzzo e Molise "G. Caporale"	Lorusso A, Marcacci M, Di Domenico M, Ancora M, Curini V, Mangone I, Rinaldi A, Scialabba S, Di Pasquale A, Cammà C, Puglia I, Calistri P, Savini G
EPI_ISL_1117694, EPI_ISL_1117695	SIESP CHIETI, DRIVE IN LANCIANO	Istituto Zooprofilattico Sperimentale dell'Abruzzo e Molise "G. Caporale"	Lorusso A, Marcacci M, Di Domenico M, Ancora M, Curini V, Mangone I, Rinaldi A, Scialabba S, Di Pasquale A, Cammà C, Puglia I, Calistri P, Savini G
EPI_ISL_1117696	SIESP CHIETI, DRIVE IN VASTO	Istituto Zooprofilattico Sperimentale dell'Abruzzo e Molise "G. Caporale"	Lorusso A, Marcacci M, Di Domenico M, Ancora M, Curini V, Mangone I, Rinaldi A, Scialabba S, Di Pasquale A, Cammà C, Puglia I, Calistri P, Savini G
EPI_ISL_1117697	DIP PREV AVEZZANO-SERVIZIO DI IGIENE EPIDEMIOLOGIA E SANITÀ PUBBLICA	Istituto Zooprofilattico Sperimentale dell'Abruzzo e Molise "G. Caporale"	Lorusso A, Marcacci M, Di Domenico M, Ancora M, Curini V, Mangone I, Rinaldi A, Scialabba S, Di Pasquale A, Cammà C, Puglia I, Calistri P, Savini G
EPI_ISL_1117698	SIESP CHIETI, DRIVE IN LANCIANO	Istituto Zooprofilattico Sperimentale dell'Abruzzo e Molise "G. Caporale"	Lorusso A, Marcacci M, Di Domenico M, Ancora M, Curini V, Mangone I, Rinaldi A, Scialabba S, Di Pasquale A, Cammà C, Puglia I, Calistri P, Savini G
EPI_ISL_1117699	SIESP CHIETI, DRIVE IN VASTO	Istituto Zooprofilattico Sperimentale dell'Abruzzo e Molise "G. Caporale"	Lorusso A, Marcacci M, Di Domenico M, Ancora M, Curini V, Mangone I, Rinaldi A, Scialabba S, Di Pasquale A, Cammà C, Puglia I, Calistri P, Savini G

EPI_ISL_1118159, EPI_ISL_1118160, EPI_ISL_1118161, EPI_ISL_1118162, EPI_ISL_1118163, EPI_ISL_1118164, EPI_ISL_1118165, EPI_ISL_1118166, EPI_ISL_1118167, EPI_ISL_1118168, EPI_ISL_1118169, EPI_ISL_1118170, EPI_ISL_1118171, EPI_ISL_1118172, EPI_ISL_1118173, EPI_ISL_1118174, EPI_ISL_1118175, EPI_ISL_1118176, EPI_ISL_1118177, EPI_ISL_1118178, EPI_ISL_1118179, EPI_ISL_1118180, EPI_ISL_1118181, EPI_ISL_1118182, EPI_ISL_1118183, EPI_ISL_1118184, EPI_ISL_1118185, EPI_ISL_1118186, EPI_ISL_1118187, EPI_ISL_1118188, EPI_ISL_1118189, EPI_ISL_1118190, EPI_ISL_1118191, EPI_ISL_1118192, EPI_ISL_1118193, EPI_ISL_1118194, EPI_ISL_1118195, EPI_ISL_1118196, EPI_ISL_1118197, EPI_ISL_1118198, EPI_ISL_1118199, EPI_ISL_1118200, EPI_ISL_1118201, EPI_ISL_1118202, EPI_ISL_1118203, EPI_ISL_1118204			
see above	LAB ANALISI PO CARDARELLI CAMPOBASSO	Istituto Zooprofilattico Sperimentale dell'Abruzzo e Molise "G. Caporale"	Scutellà M, Niro G, Felice V. Lorusso A, Marcacci M, Di Domenico M, Ancora M, Curini V, Mangone I, Rinaldi A, Scialabba S, Di Pasquale A, Cammà C, Puglia I, Calistri P, Savini G
EPI_ISL_1118217, EPI_ISL_1118223, EPI_ISL_1118224, EPI_ISL_1118231, EPI_ISL_1118232, EPI_ISL_1118233, EPI_ISL_1118234, EPI_ISL_1118235, EPI_ISL_1118236, EPI_ISL_1118237, EPI_ISL_1118238, EPI_ISL_1118239, EPI_ISL_1118240, EPI_ISL_1118241, EPI_ISL_1118242, EPI_ISL_1118243, EPI_ISL_1118244, EPI_ISL_1118245, EPI_ISL_1118246, EPI_ISL_1118247, EPI_ISL_1118248, EPI_ISL_1118249, EPI_ISL_1118250, EPI_ISL_1118251, EPI_ISL_1118252, EPI_ISL_1118253, EPI_ISL_1118254, EPI_ISL_1118255, EPI_ISL_1118256, EPI_ISL_1118257, EPI_ISL_1118258, EPI_ISL_1118259, EPI_ISL_1118260, EPI_ISL_1118261, EPI_ISL_1118264			
see above	Virology Lab, Ospedali Riuniti, Ancona	Dipartimento di Scienze Biomediche e Sanità Pubblica, Università Politecnica delle Marche	Sara Caucci, Roberta Longo, Sofia Maria Luigia Tiano, Patrizia Bagnarelli, Stefano Menzo
EPI_ISL_1118930, EPI_ISL_1118932, EPI_ISL_1118934, EPI_ISL_1119567	Laboratorio Microbiologia e Virologia P.O. Cotugno A.O. dei Colli	Laboratorio Microbiologia e Virologia P.O. Cotugno A.O. dei Colli	Luigi Atripaldi, Claudia Tiberio, Anna Perfetti, Pellegrino Cerino, Biancamaria Pierri, Maria Concetta Cuomo
EPI_ISL_1120120	Area Biologia Molecolare - Istituto Zooprofilattico Sperimentale della Sicilia	Area Biologia Molecolare - Istituto Zooprofilattico Sperimentale della Sicilia	REALE Stefano, SCIBETTA Silvia, PIAZZA Angela, BRUNO Gabriella, CASTELLI Germano, BRUNO Federica, VITALE Francesco, TRAMUTO Fabio, MAIDA Carmelo Massimo, MAZZUCCO Walter, DI NARO Daniela, RANDAZZO Giulia, VITALE Fabrizio
EPI_ISL_1121032	SIESP CHIETI, DRIVE IN LANCIANO	Istituto Zooprofilattico Sperimentale dell'Abruzzo e Molise "G. Caporale"	Lorusso A, Marcacci M, Di Domenico M, Ancora M, Curini V, Mangone I, Rinaldi A, Scialabba S, Di Pasquale A, Cammà C, Puglia I, Calistri P, Savini G
EPI_ISL_1121033	USCA CASTEL DI SANGRO	Istituto Zooprofilattico Sperimentale dell'Abruzzo e Molise "G. Caporale"	Lorusso A, Marcacci M, Di Domenico M, Ancora M, Curini V, Mangone I, Rinaldi A, Scialabba S, Di Pasquale A, Cammà C, Puglia I, Calistri P, Savini G
EPI_ISL_1121034, EPI_ISL_1121035, EPI_ISL_1121036	LAB ANALISI PO CARDARELLI CAMPOBASSO	Istituto Zooprofilattico Sperimentale dell'Abruzzo e Molise "G. Caporale"	Scutellà M, Niro G, Felice V. Lorusso A, Marcacci M, Di Domenico M, Ancora M, Curini V, Mangone I, Rinaldi A, Scialabba S, Di Pasquale A, Cammà C, Puglia I, Calistri P, Savini G
EPI_ISL_1121037	SIESP DIP PREV TERAMO	Istituto Zooprofilattico Sperimentale dell'Abruzzo e Molise "G. Caporale"	Lorusso A, Marcacci M, Di Domenico M, Ancora M, Curini V, Mangone I, Rinaldi A, Scialabba S, Di Pasquale A, Cammà C, Scialabba S, Puglia I, Calistri P, Savini G
EPI_ISL_1121038	OSP SAN SALVATORE, MEDICINA INTERNA	Istituto Zooprofilattico Sperimentale dell'Abruzzo e Molise "G. Caporale"	Lorusso A, Marcacci M, Di Domenico M, Ancora M, Curini V, Mangone I, Rinaldi A, Scialabba S, Di Pasquale A, Cammà C, Scialabba S, Puglia I, Calistri P, Savini G
EPI_ISL_1123258	Virologia, Dipartimento di Scienze Biomediche, Università di Sassari, Viale San Pietro, 43/B - Sassari	Laboratorio specialistico UOC Ematologia - Ospedale "San Francesco" - ATS-ASSL Nuoro	Giovanna Piras, Rosanna Asproni, Paolo Malune, Maria Itria Monne, Angelo Domenico Palmas, Caterina Serra, Elena Rimini, Salvatore Rubino
EPI_ISL_1123297	Virology Unit, AOUP	Virology Unit, AOUP	Mamrialinda Vatteroni, Susi Frateschi, Mauro Pistello
EPI_ISL_1123298, EPI_ISL_1123299	Virology Unit, AOUP	Virology Unit	Marialinda Vatteroni, Susi Frateschi, Mauro Pistello
EPI_ISL_1123325, EPI_ISL_1123332	Virology Unit, AOUP	Virology Unit, AOUP	Marialinda Vatteroni, Susi Frateschi, Mauro Pistello
EPI_ISL_1123344	Virology Unit, AOUP	Virology Unit	Marialinda Vatteroni, Susi Frateschi, Mauro Pistello
EPI_ISL_1123345	Virology Unit, AOUP	Virology Unit, AOUP	Marialinda Vatteroni, Susi Frateschi, Mauro Pistello
EPI_ISL_1123346, EPI_ISL_1123347	Virology Unit, AOUP	Virology Unit	Marialinda Vatteroni, Susi Frateschi, Mauro Pistello
EPI_ISL_1123349	Virology Unit, AOUP	Virology Unit, AOUP	Marialinda Vatteroni, Susi Frateschi, Mauro Pistello
EPI_ISL_1129215	Istituto Zooprofilattico Sperimentale del Mezzogiorno	Lab. Microbiologia e Virologia, Cotugno, A.O. dei Colli	Luigi Atripaldi, Claudia Tiberio, Anna Perfetti, Pellegrino Cerino, Biancamaria Pierri, Maria Concetta Cuomo
EPI_ISL_1132668	Azienda Ospedaliero - Universitaria di Modena Policlinico - Virologia e Microbiologia Molecolare	Zooprofilattico Sperimentale dell'Emilia Romagna e della Lombardia (IZSLER), Risk Analysis and Genomic Epidemiology Unit	Monica Pecorari, William Gennari, Giulia Fregni Serpini, Marina Morganti, Ilaria Menozzi, Erika Scaltriti, Stefano Pongolini
EPI_ISL_1140102, EPI_ISL_1140103, EPI_ISL_1140104, EPI_ISL_1140105, EPI_ISL_1140106, EPI_ISL_1140107, EPI_ISL_1140108, EPI_ISL_1140109, EPI_ISL_1140110, EPI_ISL_1140111, EPI_ISL_1140112, EPI_ISL_1140113, EPI_ISL_1140114, EPI_ISL_1140115, EPI_ISL_1140116			
see above	Università di Parma, Laboratorio di Igiene e Sanità Pubblica	Istituto Zooprofilattico Sperimentale della Lombardia e dell'Emilia Romagna (IZSLER), Risk Analysis and Genomic Epidemiology Unit	Maria Eugenia Colucci, Licia Veronesi, Paola Affanni, Marina Morganti, Ilaria Menozzi, Erika Scaltriti, Stefano Pongolini
EPI_ISL_1154361, EPI_ISL_1154890	Area Biologia Molecolare - Istituto Zooprofilattico Sperimentale della Sicilia	Area Biologia Molecolare - Istituto Zooprofilattico Sperimentale della Sicilia	REALE Stefano, SCIBETTA Silvia, PIAZZA Angela, BRUNO Gabriella, CASTELLI Germano, BRUNO Federica, VITALE Francesco, TRAMUTO Fabio, MAIDA Carmelo Massimo, MAZZUCCO Walter, DI NARO Daniela, RANDAZZO Giulia, VITALE Fabrizio
EPI_ISL_1157894, EPI_ISL_1157895, EPI_ISL_1157896, EPI_ISL_1157897, EPI_ISL_1157898, EPI_ISL_1157899, EPI_ISL_1157900, EPI_ISL_1157901, EPI_ISL_1157902, EPI_ISL_1157903, EPI_ISL_1157904, EPI_ISL_1157905, EPI_ISL_1157906, EPI_ISL_1157907, EPI_ISL_1157908, EPI_ISL_1157909, EPI_ISL_1157910, EPI_ISL_1157911, EPI_ISL_1157912, EPI_ISL_1157913, EPI_ISL_1157914, EPI_ISL_1157915, EPI_ISL_1157916, EPI_ISL_1157917, EPI_ISL_1157918, EPI_ISL_1157919, EPI_ISL_1157920, EPI_ISL_1157921, EPI_ISL_1157922, EPI_ISL_1157923, EPI_ISL_1157924, EPI_ISL_1157925, EPI_ISL_1157926, EPI_ISL_1157927, EPI_ISL_1157928, EPI_ISL_1157929, EPI_ISL_1157930, EPI_ISL_1157931, EPI_ISL_1157932, EPI_ISL_1157933, EPI_ISL_1157934, EPI_ISL_1157935, EPI_ISL_1157936, EPI_ISL_1157937, EPI_ISL_1157938, EPI_ISL_1157939, EPI_ISL_1157940, EPI_ISL_1157941, EPI_ISL_1157942, EPI_ISL_1157943, EPI_ISL_1157944, EPI_ISL_1157945, EPI_ISL_1157946, EPI_ISL_1157947, EPI_ISL_1157948, EPI_ISL_1157949, EPI_ISL_1157950, EPI_ISL_1157951, EPI_ISL_1157952, EPI_ISL_1157953, EPI_ISL_1157954, EPI_ISL_1157955, EPI_ISL_1157956, EPI_ISL_1157957, EPI_ISL_1157958, EPI_ISL_1157959, EPI_ISL_1157960, EPI_ISL_1157961, EPI_ISL_1157962, EPI_ISL_1157963, EPI_ISL_1157964, EPI_ISL_1157965, EPI_ISL_1157966, EPI_ISL_1157967, EPI_ISL_1157968, EPI_ISL_1157969, EPI_ISL_1157970, EPI_ISL_1157971, EPI_ISL_1157972, EPI_ISL_1157973, EPI_ISL_1157974, EPI_ISL_1157975, EPI_ISL_1157976, EPI_ISL_1157977, EPI_ISL_1157978, EPI_ISL_1157979, EPI_ISL_1157980, EPI_ISL_1157981, EPI_ISL_1157982, EPI_ISL_1157983, EPI_ISL_1157984, EPI_ISL_1157985, EPI_ISL_1157986, EPI_ISL_1157987, EPI_ISL_1157988, EPI_ISL_1157989, EPI_ISL_1157990, EPI_ISL_1157991, EPI_ISL_1157992, EPI_ISL_1157993, EPI_ISL_1157994, EPI_ISL_1157995, EPI_ISL_1157996, EPI_ISL_1157997, EPI_ISL_1157998, EPI_ISL_1157999, EPI_ISL_1158000, EPI_ISL_1158001, EPI_ISL_1158002, EPI_ISL_1158003, EPI_ISL_1158004, EPI_ISL_1158005, EPI_ISL_1158006, EPI_ISL_1158007, EPI_ISL_1158008, EPI_ISL_1158009, EPI_ISL_1158010, EPI_ISL_1158011, EPI_ISL_1158012, EPI_ISL_1158013, EPI_ISL_1158014, EPI_ISL_1158015, EPI_ISL_1158016, EPI_ISL_1158017, EPI_ISL_1158018, EPI_ISL_1158019, EPI_ISL_1158020, EPI_ISL_1158021, EPI_ISL_1158022, EPI_ISL_1158023, EPI_ISL_1158024, EPI_ISL_1158025, EPI_ISL_1158026, EPI_ISL_1158027, EPI_ISL_1158028, EPI_ISL_1158029, EPI_ISL_1158030			
see above	ASL Napoli 1 Centro	AMES Centro Polidiagnostico Strumentale S.r.l.	"Giovanni Savarese, Raffaella Ruggiero, Eloisa Evangelista, Antonella Di Carlo, Luisa Circelli, Luigi D'Amore, Roberto Sirica, Nadia Petrillo, Monica Ianniello, Antonio Fico"
EPI_ISL_1158846	Unità Operativa di Microbiologia, IRCCS Policlinico di Sant'Orsola, Azienda Ospedaliero-Universitaria di Bologna	U.O. Microbiologia Laboratorio Unico Centro Servizi AUSL della Romagna	Giada Rossini, Giuliano Furlini, Tiziana Lazzarotto, Giorgio Dirani, Silvia Zannoli, Vittorio Sambri
EPI_ISL_1163688, EPI_ISL_1163689, EPI_ISL_1163690, EPI_ISL_1163691, EPI_ISL_1163692	UOC Microbiologia e Virologia, Azienda Ospedaliera Universitaria Senese, Siena, Italy	Dipartimento di Biotecnologie Mediche	Maria Grazia Cusi, David Pinzauti, Claudia Gandolfo, Gabriele Anichini, Gianni Pozzi, Gianni Gori Savellini, Francesco Santoro
EPI_ISL_1163795, EPI_ISL_1163796, EPI_ISL_1163797	Azienda USL Umbria 2	Istituto Zooprofilattico Sperimentale dell'Abruzzo e Molise "G. Caporale"	Proietti A., Pistoni E., Lorusso A, Marcacci M, Di Domenico M, Ancora M, Curini V, Mangone I, Rinaldi A, Scialabba S, Di Pasquale A, Cammà C, Puglia I, Calistri P, Savini G
EPI_ISL_1163798, EPI_ISL_1163799, EPI_ISL_1163800, EPI_ISL_1163801, EPI_ISL_1163802, EPI_ISL_1163803, EPI_ISL_1163804, EPI_ISL_1163805, EPI_ISL_1163806, EPI_ISL_1163807, EPI_ISL_1163808, EPI_ISL_1163809, EPI_ISL_1163810, EPI_ISL_1163811, EPI_ISL_1163812, EPI_ISL_1163813, EPI_ISL_1163814, EPI_ISL_1163815, EPI_ISL_1163816, EPI_ISL_1163817, EPI_ISL_1163818, EPI_ISL_1163819, EPI_ISL_1163820, EPI_ISL_1163821, EPI_ISL_1163822, EPI_ISL_1163823, EPI_ISL_1163824, EPI_ISL_1163825, EPI_ISL_1163826, EPI_ISL_1163827, EPI_ISL_1163828			
see above	Università degli Studi di Perugia	Istituto Zooprofilattico Sperimentale dell'Abruzzo e Molise "G. Caporale"	Mencacci A., Camilloni B., Lorusso A, Marcacci M, Di Domenico M, Ancora M, Curini V, Mangone I, Rinaldi A, Scialabba S, Di Pasquale A, Cammà C, Puglia I, Calistri P, Savini G
EPI_ISL_1163829, EPI_ISL_1163830	Istituto Zooprofilattico Sperimentale Umbria e Marche "Togo Rosati"	Istituto Zooprofilattico Sperimentale dell'Abruzzo e Molise "G. Caporale"	Biagetti M., Giammarioli M., Lorusso A, Marcacci M, Di Domenico M, Ancora M, Curini V, Mangone I, Rinaldi A, Scialabba S, Di Pasquale A, Cammà C, Puglia I, Calistri P, Savini G
EPI_ISL_1163889, EPI_ISL_1163890, EPI_ISL_1163891, EPI_ISL_1163892, EPI_ISL_1163893, EPI_ISL_1163895	IZSM	IZSM-U.O.C. Virologia	Maurizio Viscardi, Lorena Cardillo, Luigi Atripaldi,Giovanna Fusco, Esterina De Carlo, Antonio Limone
EPI_ISL_1163910	IZSM	IZSM-U.O.C. Virologia	Maurizio Viscardi, Lorena Cardillo, Giuseppe Prota, Vincenzo Petrella, Luigi Atripaldi, Giovanna Fusco, Esterina De Carlo, Antonio Limone

EPI_ISL_1163911, EPI_ISL_1163912, EPI_ISL_1163913	IZSM	IZSM-U.O.C. Virologia	Maurizio Viscardi, Lorena Cardillo, Luigi Atripaldi,Giovanna Fusco, Esterina De Carlo, Antonio Limone
EPI_ISL_1164754, EPI_ISL_1164755, EPI_ISL_1164756	Azienda USL Umbria 2	Istituto Zooprofilattico Sperimentale dell'Abruzzo e Molise "G. Caporale"	Proietti A, Pistoni E, Lorusso A, Marcacci M, Di Domenico M, Ancora M, Curini V, Mangone I, Rinaldi A, Scialabba S, Di Pasquale A, Cammà C, Puglia I, Calistri P, Savini G
EPI_ISL_1164816, EPI_ISL_1164853, EPI_ISL_1164854, EPI_ISL_1164930, EPI_ISL_1164969, EPI_ISL_1165000, EPI_ISL_1165066, EPI_ISL_1165077, EPI_ISL_1165138, EPI_ISL_1165143, EPI_ISL_1165899, EPI_ISL_1166083			
see above	Struttura Semplice Dipartimentale di Virologia e Microbiologia molecolare, Azienda Ospedaliero-Universitaria, Policlinico di Modena	U.O. Microbiologia, Laboratorio Unico Centro Servizi - AUSL della Romagna	Pecorari Monica, Gennari William, Fregni Serpini Giulia, Giorgio Dirani, Silvia Zannoli, Vittorio Sambri
EPI_ISL_1166147, EPI_ISL_1166148	Laboratorio Analisi Osp. Città di Castello - Azienda USL Umbria1	Istituto Zooprofilattico Sperimentale dell'Abruzzo e Molise "G. Caporale"	Malagigi V, Tacconi P, Lorusso A, Marcacci M, Di Domenico M, Ancora M, Curini V, Mangone I, Rinaldi A, Scialabba S, Di Pasquale A, Cammà C, Puglia I, Calistri P, Savini G
EPI_ISL_1166149, EPI_ISL_1166150, EPI_ISL_1166151, EPI_ISL_1166152, EPI_ISL_1166153, EPI_ISL_1166154, EPI_ISL_1166155, EPI_ISL_1166156, EPI_ISL_1166157, EPI_ISL_1166158, EPI_ISL_1166159, EPI_ISL_1166160, EPI_ISL_1166161, EPI_ISL_1166162, EPI_ISL_1166163, EPI_ISL_1166164, EPI_ISL_1166165, EPI_ISL_1166166, EPI_ISL_1166167, EPI_ISL_1166168, EPI_ISL_1166169, EPI_ISL_1166170, EPI_ISL_1166171			
see above	Università degli Studi di Perugia	Istituto Zooprofilattico Sperimentale dell'Abruzzo e Molise "G. Caporale"	Mencacci A, Camilloni B, Lorusso A, Marcacci M, Di Domenico M, Ancora M, Curini V, Mangone I, Rinaldi A, Scialabba S, Di Pasquale A, Cammà C, Puglia I, Calistri P, Savini G
EPI_ISL_1166172, EPI_ISL_1166173, EPI_ISL_1166174, EPI_ISL_1166175, EPI_ISL_1166176, EPI_ISL_1166177, EPI_ISL_1166178, EPI_ISL_1166179, EPI_ISL_1166180, EPI_ISL_1166181, EPI_ISL_1166182, EPI_ISL_1166183, EPI_ISL_1166184, EPI_ISL_1166185, EPI_ISL_1166186, EPI_ISL_1166187, EPI_ISL_1166188			
see above	Istituto Zooprofilattico Sperimentale Umbria e Marche "Togo Rosati"	Istituto Zooprofilattico Sperimentale dell'Abruzzo e Molise "G. Caporale"	Biagetti M, Giammarioli M, Lorusso A, Marcacci M, Di Domenico M, Ancora M, Curini V, Mangone I, Rinaldi A, Scialabba S, Di Pasquale A, Cammà C, Puglia I, Calistri P, Savini G
EPI_ISL_1166221, EPI_ISL_1166222, EPI_ISL_1166223, EPI_ISL_1166224, EPI_ISL_1166225, EPI_ISL_1166226, EPI_ISL_1166227, EPI_ISL_1166228, EPI_ISL_1166229, EPI_ISL_1166230, EPI_ISL_1166231, EPI_ISL_1166232, EPI_ISL_1166233, EPI_ISL_1166234, EPI_ISL_1166235, EPI_ISL_1166236, EPI_ISL_1166237, EPI_ISL_1166238, EPI_ISL_1166239, EPI_ISL_1166240, EPI_ISL_1166241, EPI_ISL_1166242, EPI_ISL_1166243, EPI_ISL_1166244, EPI_ISL_1166245, EPI_ISL_1166246, EPI_ISL_1166247, EPI_ISL_1166248, EPI_ISL_1166249, EPI_ISL_1166250, EPI_ISL_1166251, EPI_ISL_1166252, EPI_ISL_1166253, EPI_ISL_1166254, EPI_ISL_1166255, EPI_ISL_1166256, EPI_ISL_1166257, EPI_ISL_1166258, EPI_ISL_1166259, EPI_ISL_1166260, EPI_ISL_1166261, EPI_ISL_1166262, EPI_ISL_1166263, EPI_ISL_1166264, EPI_ISL_1166265, EPI_ISL_1166266, EPI_ISL_1166267, EPI_ISL_1166268, EPI_ISL_1166269, EPI_ISL_1166270, EPI_ISL_1166271, EPI_ISL_1166272, EPI_ISL_1166273, EPI_ISL_1166274, EPI_ISL_1166275, EPI_ISL_1166276, EPI_ISL_1166277, EPI_ISL_1166278, EPI_ISL_1166279, EPI_ISL_1166280, EPI_ISL_1166281, EPI_ISL_1166282, EPI_ISL_1166283, EPI_ISL_1166284, EPI_ISL_1166285, EPI_ISL_1166286, EPI_ISL_1166287, EPI_ISL_1166288, EPI_ISL_1166289, EPI_ISL_1166290, EPI_ISL_1166291, EPI_ISL_1166292, EPI_ISL_1166293, EPI_ISL_1166294, EPI_ISL_1166295, EPI_ISL_1166296, EPI_ISL_1166297, EPI_ISL_1166298, EPI_ISL_1166299, EPI_ISL_1166300, EPI_ISL_1166301, EPI_ISL_1166302, EPI_ISL_1166303, EPI_ISL_1166304, EPI_ISL_1166305, EPI_ISL_1166306, EPI_ISL_1166307, EPI_ISL_1166308, EPI_ISL_1166309, EPI_ISL_1166310, EPI_ISL_1166311, EPI_ISL_1166312, EPI_ISL_1166313, EPI_ISL_1166314, EPI_ISL_1166315, EPI_ISL_1166316, EPI_ISL_1166317, EPI_ISL_1166318, EPI_ISL_1166319, EPI_ISL_1166320, EPI_ISL_1166321, EPI_ISL_1166322, EPI_ISL_1166323, EPI_ISL_1166324, EPI_ISL_1166325, EPI_ISL_1166326, EPI_ISL_1166327, EPI_ISL_1166328, EPI_ISL_1166329, EPI_ISL_1166330, EPI_ISL_1166331, EPI_ISL_1166332, EPI_ISL_1166333, EPI_ISL_1166334, EPI_ISL_1166335, EPI_ISL_1166336, EPI_ISL_1166337, EPI_ISL_1166338, EPI_ISL_1166339, EPI_ISL_1166340, EPI_ISL_1166341, EPI_ISL_1166342, EPI_ISL_1166343, EPI_ISL_1166344, EPI_ISL_1166345, EPI_ISL_1166346, EPI_ISL_1166347, EPI_ISL_1166348, EPI_ISL_1166349, EPI_ISL_1166350, EPI_ISL_1166351, EPI_ISL_1166352, EPI_ISL_1166353, EPI_ISL_1166354, EPI_ISL_1166355, EPI_ISL_1166356, EPI_ISL_1166357, EPI_ISL_1166358, EPI_ISL_1166359, EPI_ISL_1166360, EPI_ISL_1166361, EPI_ISL_1166362, EPI_ISL_1166363, EPI_ISL_1166364, EPI_ISL_1166365, EPI_ISL_1166366, EPI_ISL_1166367, EPI_ISL_1166368, EPI_ISL_1166369, EPI_ISL_1166370, EPI_ISL_1166371, EPI_ISL_1166372, EPI_ISL_1166373, EPI_ISL_1166374, EPI_ISL_1166375, EPI_ISL_1166376, EPI_ISL_1166377, EPI_ISL_1166378, EPI_ISL_1166379, EPI_ISL_1166380, EPI_ISL_1166381, EPI_ISL_1166382, EPI_ISL_1166383, EPI_ISL_1166384, EPI_ISL_1166385, EPI_ISL_1166386, EPI_ISL_1166387, EPI_ISL_1166388, EPI_ISL_1166389, EPI_ISL_1166390, EPI_ISL_1166391, EPI_ISL_1166392, EPI_ISL_1166393, EPI_ISL_1166394, EPI_ISL_1166395, EPI_ISL_1166396, EPI_ISL_1166397, EPI_ISL_1166398, EPI_ISL_1166399, EPI_ISL_1166400, EPI_ISL_1166401, EPI_ISL_1166402, EPI_ISL_1166403, EPI_ISL_1166404, EPI_ISL_1166405, EPI_ISL_1166406, EPI_ISL_1166407, EPI_ISL_1166408, EPI_ISL_1166409, EPI_ISL_1166410, EPI_ISL_1166411, EPI_ISL_1166412, EPI_ISL_1166413, EPI_ISL_1166414, EPI_ISL_1166415, EPI_ISL_1166416, EPI_ISL_1166417, EPI_ISL_1166418, EPI_ISL_1166419, EPI_ISL_1166420, EPI_ISL_1166421, EPI_ISL_1166422, EPI_ISL_1166423, EPI_ISL_1166424, EPI_ISL_1166425, EPI_ISL_1166426, EPI_ISL_1166427, EPI_ISL_1166428, EPI_ISL_1166429, EPI_ISL_1166430, EPI_ISL_1166431, EPI_ISL_1166432, EPI_ISL_1166433, EPI_ISL_1166434, EPI_ISL_1166435, EPI_ISL_1166436, EPI_ISL_1166437, EPI_ISL_1166438, EPI_ISL_1166439, EPI_ISL_1166440, EPI_ISL_1166441, EPI_ISL_1166442, EPI_ISL_1166443, EPI_ISL_1166444, EPI_ISL_1166445, EPI_ISL_1166446, EPI_ISL_1166447, EPI_ISL_1166448, EPI_ISL_1166449, EPI_ISL_1166450, EPI_ISL_1166451, EPI_ISL_1166452, EPI_ISL_1166453, EPI_ISL_1166454, EPI_ISL_1166455, EPI_ISL_1166456, EPI_ISL_1166457, EPI_ISL_1166458, EPI_ISL_1166459, EPI_ISL_1166460, EPI_ISL_1166461, EPI_ISL_1166462, EPI_ISL_1166463, EPI_ISL_1166464, EPI_ISL_1166465, EPI_ISL_1166466, EPI_ISL_1166467, EPI_ISL_1166468, EPI_ISL_1166469, EPI_ISL_1166470, EPI_ISL_1166471, EPI_ISL_1166472, EPI_ISL_1166473, EPI_ISL_1166474, EPI_ISL_1166475, EPI_ISL_1166476, EPI_ISL_1166477, EPI_ISL_1166478, EPI_ISL_1166479, EPI_ISL_1166480, EPI_ISL_1166481, EPI_ISL_1166482, EPI_ISL_1166483, EPI_ISL_1166484, EPI_ISL_1166485, EPI_ISL_1166486, EPI_ISL_1166487, EPI_ISL_1166488, EPI_ISL_1166489, EPI_ISL_1166490, EPI_ISL_1166491, EPI_ISL_1166492, EPI_ISL_1166493, EPI_ISL_1166494, EPI_ISL_1166495, EPI_ISL_1166496, EPI_ISL_1166497, EPI_ISL_1166498, EPI_ISL_1166499, EPI_ISL_1166500, EPI_ISL_1166501, EPI_ISL_1166502, EPI_ISL_1166503, EPI_ISL_1166504, EPI_ISL_1166505, EPI_ISL_1166506, EPI_ISL_1166507, EPI_ISL_1166508, EPI_ISL_1166509, EPI_ISL_1166510, EPI_ISL_1166511, EPI_ISL_1166512, EPI_ISL_1166513, EPI_ISL_1166514, EPI_ISL_1166515, EPI_ISL_1166516, EPI_ISL_1166517, EPI_ISL_1166518, EPI_ISL_1166519, EPI_ISL_1166520, EPI_ISL_1166521, EPI_ISL_1166522, EPI_ISL_1166523, EPI_ISL_1166524, EPI_ISL_1166525, EPI_ISL_1166526, EPI_ISL_1166527, EPI_ISL_1166528, EPI_ISL_1166529, EPI_ISL_1166530, EPI_ISL_1166531, EPI_ISL_1166532, EPI_ISL_1166533			
see above	ASL Napoli 1 Centro	AMES Centro Polidiagnostico Strumentale S.r.l.	"Giovanni Savarese, Raffaella Ruggiero, Eloisa Evangelista, Antonella Di Carlo, Luisa Circelli, Luigi D'Amore, Nadia Petrillo, Monica Ianniello, Roberto Sirica,Maurizio D'Amora, Antonio Fico"
EPI_ISL_1166877, EPI_ISL_1166878, EPI_ISL_1166879, EPI_ISL_1166880, EPI_ISL_1166881, EPI_ISL_1166882	IZSM	IZSM-U.O.C. Virologia	Maurizio Viscardi, Lorena Cardillo, Luigi Atripaldi,Giovanna Fusco, Esterina De Carlo, Antonio Limone
EPI_ISL_1167308, EPI_ISL_1167309, EPI_ISL_1167310, EPI_ISL_1167311, EPI_ISL_1167312, EPI_ISL_1167313, EPI_ISL_1167314, EPI_ISL_1167315, EPI_ISL_1167316, EPI_ISL_1167317, EPI_ISL_1167318, EPI_ISL_1167319, EPI_ISL_1167320, EPI_ISL_1167321, EPI_ISL_1167322, EPI_ISL_1167323, EPI_ISL_1167324, EPI_ISL_1167325, EPI_ISL_1167326, EPI_ISL_1167327, EPI_ISL_1167328, EPI_ISL_1167329, EPI_ISL_1167330, EPI_ISL_1167331, EPI_ISL_1167332, EPI_ISL_1167333, EPI_ISL_1167334, EPI_ISL_1167335, EPI_ISL_1167336, EPI_ISL_1167337, EPI_ISL_1167338, EPI_ISL_1167339, EPI_ISL_1167340, EPI_ISL_1167341, EPI_ISL_1167342, EPI_ISL_1167343, EPI_ISL_1167344, EPI_ISL_1167345, EPI_ISL_1167346, EPI_ISL_1167347, EPI_ISL_1167348, EPI_ISL_1167349, EPI_ISL_1167350, EPI_ISL_1167351, EPI_ISL_1167352, EPI_ISL_1167353, EPI_ISL_1167354, EPI_ISL_1167355, EPI_ISL_1167356, EPI_ISL_1167357, EPI_ISL_1167358, EPI_ISL_1167359, EPI_ISL_1167360, EPI_ISL_1167361, EPI_ISL_1167362, EPI_ISL_1167363, EPI_ISL_1167364, EPI_ISL_1167365, EPI_ISL_1167366, EPI_ISL_1167367, EPI_ISL_1167368, EPI_ISL_1167369, EPI_ISL_1167370, EPI_ISL_1167371, EPI_ISL_1167372, EPI_ISL_1167373, EPI_ISL_1167374, EPI_ISL_1167375, EPI_ISL_1167376, EPI_ISL_1167377, EPI_ISL_1167378, EPI_ISL_1167379, EPI_ISL_1167380, EPI_ISL_1167381, EPI_ISL_1167382, EPI_ISL_1167383, EPI_ISL_1167384, EPI_ISL_1167385, EPI_ISL_1167386, EPI_ISL_1167387, EPI_ISL_1167388, EPI_ISL_1167389, EPI_ISL_1167390, EPI_ISL_1167391, EPI_ISL_1167392, EPI_ISL_1167393, EPI_ISL_1167394, EPI_ISL_1167395, EPI_ISL_1167396, EPI_ISL_1167397, EPI_ISL_1167398, EPI_ISL_1167399, EPI_ISL_1167400, EPI_ISL_1167401, EPI_ISL_1167402, EPI_ISL_1167403, EPI_ISL_1167404, EPI_ISL_1167405, EPI_ISL_1167406, EPI_ISL_1167407, EPI_ISL_1167408, EPI_ISL_1167409, EPI_ISL_1167410, EPI_ISL_1167411, EPI_ISL_1167412, EPI_ISL_1167413, EPI_ISL_1167414, EPI_ISL_1167415, EPI_ISL_1167416, EPI_ISL_1167417, EPI_ISL_1167418, EPI_ISL_1167419, EPI_ISL_1167420, EPI_ISL_1167421, EPI_ISL_1167422, EPI_ISL_1167423, EPI_ISL_1167424, EPI_ISL_1167425, EPI_ISL_1167426, EPI_ISL_1167427, EPI_ISL_1167428, EPI_ISL_1167429, EPI_ISL_1167430, EPI_ISL_1167431, EPI_ISL_1167432, EPI_ISL_1167433, EPI_ISL_1167434, EPI_ISL_1167435, EPI_ISL_1167436, EPI_ISL_1167437, EPI_ISL_1167438, EPI_ISL_1167439, EPI_ISL_1167440, EPI_ISL_1167441, EPI_ISL_1167442, EPI_ISL_1167443, EPI_ISL_1167444, EPI_ISL_1167445, EPI_ISL_1167446, EPI_ISL_1167447, EPI_ISL_1167448, EPI_ISL_1167449, EPI_ISL_1167450, EPI_ISL_1167451, EPI_ISL_1167452, EPI_ISL_1167453, EPI_ISL_1167454, EPI_ISL_1167455, EPI_ISL_1167456, EPI_ISL_1167457, EPI_ISL_1167458, EPI_ISL_1167459, EPI_ISL_1167460, EPI_ISL_1167461, EPI_ISL_1167462, EPI_ISL_1167463, EPI_ISL_1167464, EPI_ISL_1167465, EPI_ISL_1167466, EPI_ISL_1167467, EPI_ISL_1167468, EPI_ISL_1167469, EPI_ISL_1167470, EPI_ISL_1167471, EPI_ISL_1167472, EPI_ISL_1167473, EPI_ISL_1167474, EPI_ISL_1167475, EPI_ISL_1167476, EPI_ISL_1167477, EPI_ISL_1167478, EPI_ISL_1167479, EPI_ISL_1167480, EPI_ISL_1167481, EPI_ISL_1167482, EPI_ISL_1167483, EPI_ISL_1167484, EPI_ISL_1167485, EPI_ISL_1167486, EPI_ISL_1167487, EPI_ISL_1167488, EPI_ISL_1167489, EPI_ISL_1167490, EPI_ISL_1167491, EPI_ISL_1167492, EPI_ISL_1167493, EPI_ISL_1167494, EPI_ISL_1167495, EPI_ISL_1167496, EPI_ISL_1167497, EPI_ISL_1167498, EPI_ISL_1167499, EPI_ISL_1167500, EPI_ISL_1167501, EPI_ISL_1167502, EPI_ISL_1167503, EPI_ISL_1167504, EPI_ISL_1167505, EPI_ISL_1167506, EPI_ISL_1167507, EPI_ISL_1167508, EPI_ISL_1167509, EPI_ISL_1167510, EPI_ISL_1167511, EPI_ISL_1167512, EPI_ISL_1167513, EPI_ISL_1167514, EPI_ISL_1167515, EPI_ISL_1167516, EPI_ISL_1167517, EPI_ISL_1167518, EPI_ISL_1167519, EPI_ISL_1167520, EPI_ISL_1167521, EPI_ISL_1167522, EPI_ISL_1167523, EPI_ISL_1167524, EPI_ISL_1167525, EPI_ISL_1167526, EPI_ISL_1167527, EPI_ISL_1167528, EPI_ISL_1167529, EPI_ISL_1167530, EPI_ISL_1167531, EPI_ISL_1167532, EPI_ISL_1167533, EPI_ISL_1167534, EPI_ISL_1167535, EPI_ISL_1167536, EPI_ISL_1167537, EPI_ISL_1167538, EPI_ISL_1167539, EPI_ISL_1167540, EPI_ISL_1167541, EPI_ISL_1167542, EPI_ISL_1167543, EPI_ISL_1167544, EPI_ISL_1167545, EPI_ISL_1167546, EPI_ISL_1169061, EPI_ISL_1169062, EPI_ISL_1169063, EPI_ISL_1169064, EPI_ISL_1169065, EPI_ISL_1169066, EPI_ISL_1169067, EPI_ISL_1169068, EPI_ISL_1169069, EPI_ISL_1169070, EPI_ISL_1169071, EPI_ISL_1169072, EPI_ISL_1169073, EPI_ISL_1169074, EPI_ISL_1169075, EPI_ISL_1169076, EPI_ISL_1169077, EPI_ISL_1169078, EPI_ISL_1169079, EPI_ISL_1169080, EPI_ISL_1169081, EPI_ISL_1169082, EPI_ISL_1169083, EPI_ISL_1169084, EPI_ISL_1169085, EPI_ISL_1169086, EPI_ISL_1169087, EPI_ISL_1169088, EPI_ISL_1169089, EPI_ISL_1169090, EPI_ISL_1169091, EPI_ISL_1169092, EPI_ISL_1169093, EPI_ISL_1169094, EPI_ISL_1169095, EPI_ISL_1169096, EPI_ISL_1169097, EPI_ISL_1169098, EPI_ISL_1169099, EPI_ISL_1169100, EPI_ISL_1169101, EPI_ISL_1169102, EPI_ISL_1169103, EPI_ISL_1169104, EPI_ISL_1169105, EPI_ISL_1169106, EPI_ISL_1169107, EPI_ISL_1169108, EPI_ISL_1169109, EPI_ISL_1169110, EPI_ISL_1169111, EPI_ISL_1169112, EPI_ISL_1169113, EPI_ISL_1169114, EPI_ISL_1169115, EPI_ISL_1169116, EPI_ISL_1169117, EPI_ISL_1169118, EPI_ISL_1169119, EPI_ISL_1169120, EPI_ISL_1169121, EPI_ISL_1169122, EPI_ISL_1169123, EPI_ISL_1169124, EPI_ISL_1169125, EPI_ISL_1169126, EPI_ISL_1169127, EPI_ISL_1169128, EPI_ISL_1169129, EPI_ISL_1169130, EPI_ISL_1169131, EPI_ISL_1169132, EPI_ISL_1169133, EPI_ISL_1169134, EPI_ISL_1169135, EPI_ISL_1169136, EPI_ISL_1169137, EPI_ISL_1169138, EPI_ISL_1169139, EPI_ISL_1169140, EPI_ISL_1169141, EPI_ISL_1169142, EPI_ISL_1169143, EPI_ISL_1169144, EPI_ISL_1169145, EPI_ISL_1169146, EPI_ISL_1169147, EPI_ISL_1169148, EPI_ISL_1169149, EPI_ISL_1169150, EPI_ISL_1169151, EPI_ISL_1169152, EPI_ISL_1169153, EPI_ISL_1169154, EPI_ISL_1169155, EPI_ISL_1169156, EPI_ISL_1169157, EPI_ISL_1169158, EPI_ISL_1169159, EPI_ISL_1169160, EPI_ISL_1169161, EPI_ISL_1169162, EPI_ISL_1169163, EPI_ISL_1169164, EPI_ISL_1169165, EPI_ISL_1169166, EPI_ISL_1169167, EPI_ISL_1169168, EPI_ISL_1169169, EPI_ISL_1169170, EPI_ISL_1169171, EPI_ISL_1169172, EPI_ISL_1169173, EPI_ISL_1169174, EPI_ISL_1169175, EPI_ISL_1169176, EPI_ISL_1169177, EPI_ISL_1169178, EPI_ISL_1169179, EPI_ISL_1169180, EPI_ISL_1169181, EPI_ISL_1169182, EPI_ISL_1169183, EPI_ISL_1169184, EPI_ISL_1169185, EPI_ISL_1169186, EPI_ISL_1169187, EPI_ISL_1169188, EPI_ISL_1169189, EPI_ISL_1169190, EPI_ISL_1169191, EPI_ISL_1169192, EPI_ISL_1169193, EPI_ISL_1169194, EPI_ISL_1169195, EPI_ISL_1169196, EPI_ISL_1169197, EPI_ISL_1169198, EPI_ISL_1169199, EPI_ISL_1169200, EPI_ISL_1169201, EPI_ISL_1169202, EPI_ISL_1169203, EPI_ISL_1169204, EPI_ISL_1169205, EPI_ISL_1169206, EPI_ISL_1169207, EPI_ISL_1169208, EPI_ISL_1169209, EPI_ISL_1169210, EPI_ISL_1169211, EPI_ISL_1169212, EPI_ISL_1169213, EPI_ISL_1169214, EPI_ISL_1169215, EPI_ISL_1169216, EPI_ISL_1169217, EPI_ISL_1169218, EPI_ISL_1169219, EPI_ISL_1169220, EPI_ISL_1169221, EPI_ISL_1169222, EPI_ISL_1169223, EPI_ISL_1169224, EPI_ISL_1169225, EPI_ISL_1169226, EPI_ISL_1169227, EPI_ISL_1169228, EPI_ISL_1169229, EPI_ISL_1169230, EPI_ISL_1169231, EPI_ISL_1169232, EPI_ISL_1169233, EPI_ISL_1169234, EPI_ISL_1169235, EPI_ISL_1169236, EPI_ISL_1169237, EPI_ISL_1169238, EPI_ISL_1169239, EPI_ISL_1169240, EPI_ISL_1169241, EPI_ISL_1169242, EPI_ISL_1169243, EPI_ISL_1169244, EPI_ISL_1169245, EPI_ISL_1169246, EPI_ISL_1169247, EPI_ISL_1169248, EPI_ISL_1169249, EPI_ISL_1169250, EPI_ISL_1169251, EPI_ISL_1169252, EPI_ISL_1169253, EPI_ISL_1169254, EPI_ISL_1169255, EPI_ISL_1169256, EPI_ISL_1169257, EPI_ISL_1169258, EPI_ISL_1169259, EPI_ISL_1169260, EPI_ISL_1169261, EPI_ISL_1169262, EPI_ISL_1169263, EPI_ISL_1169264, EPI_ISL_1169265, EPI_ISL_1169266, EPI_ISL_1169267, EPI_ISL_1169268, EPI_ISL_1169269, EPI_ISL_1169270, EPI_ISL_1169271, EPI_ISL_1169272, EPI_ISL_1169273, EPI_ISL_1169274, EPI_ISL_1169275, EPI_ISL_1169276, EPI_ISL_1169277, EPI_ISL_1169278, EPI_ISL_1169279, EPI_ISL_1169280, EPI_ISL_1169281, EPI_ISL_1169282, EPI_ISL_1169283, EPI_ISL_1169284, EPI_ISL_1169285, EPI_ISL_1169286, EPI_ISL_1169287, EPI_ISL_1169288, EPI_ISL_1169289, EPI_ISL_1169290, EPI_ISL_1169291, EPI_ISL_1169292, EPI_ISL_1169293, EPI_ISL_1169294, EPI_ISL_1169295, EPI_ISL_1169296, EPI_ISL_1169297, EPI_ISL_1169298, EPI_ISL_1169299, EPI_ISL_1169300, EPI_ISL_1169301, EPI_ISL_1169302, EPI_ISL_1169303, EPI_ISL_1169304, EPI_ISL_1169305, EPI_ISL_1169306, EPI_ISL_1169307, EPI_ISL_1169308, EPI_ISL_1169309, EPI_ISL_1169310, EPI_ISL_1169311, EPI_ISL_1169312, EPI_ISL_1169313, EPI_ISL_1169314, EPI_ISL_1169315, EPI_ISL_1169316, EPI_ISL_1169317, EPI_ISL_1169318, EPI_ISL_1169319, EPI_ISL_1169320, EPI_ISL_1169321, EPI_ISL_1169322, EPI_ISL_1169323, EPI_ISL_1169324, EPI_ISL_1169325, EPI_ISL_1169326, EPI_ISL_1169327, EPI_ISL_1169328, EPI_ISL_1169329, EPI_ISL_1169330, EPI_ISL_1169331, EPI_ISL_1169332, EPI_ISL_1169333, EPI_ISL_1169334, EPI_ISL_1169335, EPI_ISL_1169336, EPI_ISL_1169337, EPI_ISL_1169338, EPI_ISL_1169339, EPI_ISL_1169340, EPI_ISL_1169341, EPI_ISL_1169342, EPI_ISL_1169343, EPI_ISL_1169344, EPI_ISL_1169345, EPI_ISL_1169346, EPI_ISL_1169347, EPI_ISL_1169348, EPI_ISL_1169349, EPI_ISL_1169350, EPI_ISL_1169351, EPI_ISL_1169352, EPI_ISL_1169353, EPI_ISL_1169354, EPI_ISL_1169355, EPI_ISL_1169356, EPI_ISL_1169357, EPI_ISL_1169358, EPI_ISL_1169359, EPI_ISL_1169360, EPI_ISL_1169361, EPI_ISL_1169362, EPI_ISL_1169363, EPI_ISL_1169364, EPI_ISL_1169365, EPI_ISL_1169366, EPI_ISL_1169367, EPI_ISL_1169368, EPI_ISL_1169369, EPI_ISL_1169370, EPI_ISL_1169371, EPI_ISL_1169372, EPI_ISL_1169373, EPI_ISL_1169374, EPI_ISL_1169375, EPI_ISL_1169376, EPI_ISL_1169377, EPI_ISL_1169378, EPI_ISL_1169379, EPI_ISL_1169380, EPI_ISL_1169381, EPI_ISL_1169382, EPI_ISL_1169383, EPI_ISL_1169384, EPI_ISL_1169385, EPI_ISL_1169386, EPI_ISL_1169387, EPI_ISL_1169388, EPI_ISL_1169389, EPI_ISL_1169390, EPI_ISL_1169391, EPI_ISL_1169392, EPI_ISL_1169393, EPI_ISL_1169394, EPI_ISL_1169395, EPI_ISL_1169396, EPI_ISL_1169397, EPI_ISL_1169398, EPI_ISL_1169399, EPI_ISL_1169400, EPI_ISL_1169401, EPI_ISL_1169402, EPI_ISL_1169403, EPI_ISL_1169404, EPI_ISL_1169405, EPI_ISL_1169406, EPI_ISL_1169407, EPI_ISL_1169408, EPI_ISL_1169409, EPI_ISL_1169410, EPI_ISL_1169411, EPI_ISL_1169412, EPI_ISL_1169413, EPI_ISL_1169414, EPI_ISL_1169415, EPI_ISL_1169416, EPI_ISL_1169417, EPI_ISL_1169418, EPI_ISL_1169419, EPI_ISL_1169420, EPI_ISL_1169421, EPI_ISL_1169422, EPI_ISL_1169423, EPI_ISL_1169424, EPI_ISL_1169425, EPI_ISL_1169426, EPI_ISL_1169427, EPI_ISL_1169428, EPI_ISL_1169429, EPI_ISL_1169430, EPI_ISL_1169431, EPI_ISL_1169432, EPI_ISL_1169433, EPI_ISL_1169434, EPI_ISL_1169435, EPI_ISL_1169436, EPI_ISL_1169437, EPI_ISL_1169438, EPI_ISL_1169439, EPI_ISL_1169440, EPI_ISL_1169441, EPI_ISL_1169442, EPI_ISL_1169443, EPI_ISL_1169444, EPI_ISL_1169445, EPI_ISL_1169446, EPI_ISL_1169447, EPI_ISL_1169448, EPI_ISL_1169449, EPI_ISL_1169450, EPI_ISL_1169451, EPI_ISL_1169452, EPI_ISL_1169453, EPI_ISL_1169454, EPI_ISL_1169455, EPI_ISL_1169456, EPI_ISL_1169457, EPI_ISL_1169458, EPI_ISL_1169459, EPI_ISL_1169460, EPI_ISL_1169461, EPI_ISL_1169462, EPI_ISL_1169463, EPI_ISL_1169464, EPI_ISL_1169465, EPI_ISL_1169466, EPI_ISL_1169467, EPI_ISL_1169468, EPI_ISL_1169469, EPI_ISL_1169470, EPI_ISL_1169471, EPI_ISL_1169472, EPI_ISL_1169473, EPI_ISL_1169474, EPI_ISL_1169475, EPI_ISL_1169476, EPI_ISL_1169477, EPI_ISL_1169478, EPI_ISL_1169479, EPI_ISL_1169480, EPI_ISL_1169481, EPI_ISL_1169482			
see above	ASL Napoli 1 Centro	AMES Centro Polidiagnostico Strumentale S.r.l.	"Giovanni Savarese, Raffaella Ruggiero, Eloisa Evangelista, Antonella Di Carlo, Luisa Circelli, Luigi D'Amore, Nadia Petrillo, Monica Ianniello, Roberto

			Sirica,Maurizio D'Amora, Antonio Fico"
EPI_ISL_1169883	INMI Lazzaro Spallanzani IRCCS	INMI Lazzaro Spallanzani IRCCS	CEM Gruber, B Bartolini, E Giombini, F Messina, M Rueca, O Butera, MR Capobianchi, A Di Caro
EPI_ISL_1169884	INMI Lazzaro Spallanzani IRCCS	INMI Lazzaro Spallanzani IRCCS	F Messina, M Rueca, O Butera, CEM Gruber, B Bartolini, E Giombini, MR Capobianchi, A Di Caro
EPI_ISL_1169885	INMI Lazzaro Spallanzani IRCCS	INMI Lazzaro Spallanzani IRCCS	M Rueca, O Butera, CEM Gruber, B Bartolini, E Giombini, F Messina, A Di Caro, MR Capobianchi
EPI_ISL_1169886	INMI Lazzaro Spallanzani IRCCS	INMI Lazzaro Spallanzani IRCCS	B Bartolini, E Giombini, F Messina, M Rueca, O Butera, CEM Gruber, A Di Caro, MR Capobianchi
EPI_ISL_1169887	San Gallicano Dermatological Institute I.F.O.	INMI Lazzaro Spallanzani IRCCS	E Giombini, F Messina, M Rueca, O Butera, CEM Gruber, B Bartolini, F Pimpinelli, F Ensoli, EG Di Domenico, A Di Caro, MR Capobianchi
EPI_ISL_1169888	Ospedale "F. Spaziani" Frosinone	INMI Lazzaro Spallanzani IRCCS	E Giombini, F Messina, M Rueca, O Butera, CEM Gruber, B Bartolini, R Pulselli, C Gargiulo, C Sias, A Di Caro, MR Capobianchi
EPI_ISL_1169889	San Gallicano Dermatological Institute I.F.O.	INMI Lazzaro Spallanzani IRCCS	CEM Gruber, B Bartolini, E Giombini, F Messina, M Rueca, O Butera, F Pimpinelli, F Ensoli, G D'Agosto, MR Capobianchi, A Di Caro
EPI_ISL_1169890	Laboratorio Genzano - ASL RM 6	INMI Lazzaro Spallanzani IRCCS	O Butera, CEM Gruber, B Bartolini, E Giombini, F Messina, M Rueca, G Tramini, E Conti, MR Capobianchi, A Di Caro
EPI_ISL_1169891	Synlab Lazio S.r.l.	INMI Lazzaro Spallanzani IRCCS	F Messina, M Rueca, O Butera, CEM Gruber, B Bartolini, E Giombini, E Trappolini, SA Santini, MR Capobianchi, A Di Caro
EPI_ISL_1169892, EPI_ISL_1169893	Università degli Studi di Perugia	Istituto Zooprofilattico Sperimentale dell'Abruzzo e Molise "G. Caporale"	Mencacci A., Camilloni B., Lorusso A, Marcacci M, Di Domenico M, Ancora M, Curini V, Mangone I, Rinaldi A, Scialabba S, Di Pasquale A, Cammà C, Puglia I, Calistri P, Savini G
EPI_ISL_1169894, EPI_ISL_1169895	Istituto Zooprofilattico Sperimentale Umbria e Marche "Togo Rosati"	Istituto Zooprofilattico Sperimentale dell'Abruzzo e Molise "G. Caporale"	Biagetti M., Giammarioli M., Lorusso A, Marcacci M, Di Domenico M, Ancora M, Curini V, Mangone I, Rinaldi A, Scialabba S, Di Pasquale A, Cammà C, Puglia I, Calistri P, Savini G
EPI_ISL_1169905, EPI_ISL_1169906, EPI_ISL_1169907, EPI_ISL_1169908, EPI_ISL_1169909, EPI_ISL_1169910, EPI_ISL_1169911	UOC Microbiologia e Virologia, Azienda Ospedaliera Universitaria Senese, Siena, Italy	Dipartimento di Biotecnologie Mediche	Maria Grazia Cusi, David Pinzauti, Claudia Gandolfo, Gabriele Anichini, Gianni Pozzi, Gianni Gori Savellini, Francesco Santoro
EPI_ISL_1171675, EPI_ISL_1171676, EPI_ISL_1171677, EPI_ISL_1171678, EPI_ISL_1171679, EPI_ISL_1171680, EPI_ISL_1171681, EPI_ISL_1171682, EPI_ISL_1171683, EPI_ISL_1171684, EPI_ISL_1171685, EPI_ISL_1171686, EPI_ISL_1171687, EPI_ISL_1171688, EPI_ISL_1171689, EPI_ISL_1171690, EPI_ISL_1171691, EPI_ISL_1171692, EPI_ISL_1171693, EPI_ISL_1171694, EPI_ISL_1171695, EPI_ISL_1171696, EPI_ISL_1171697, EPI_ISL_1171698, EPI_ISL_1171699, EPI_ISL_1171700, EPI_ISL_1171701, EPI_ISL_1171702, EPI_ISL_1171703, EPI_ISL_1171704, EPI_ISL_1171705			
see above	UOC LAB ANALISI PO CARDARELLI CAMPOBASSO	Istituto Zooprofilattico Sperimentale dell'Abruzzo e Molise "G. Caporale"	Scutellà M, Niro G, Felice V. Lorusso A, Marcacci M, Di Domenico M, Ancora M, Curini V, Mangone I, Rinaldi A, Scialabba S, Di Pasquale A, Cammà C, Puglia I, Calistri P, Savini G
EPI_ISL_1173204, EPI_ISL_1173205	Istituto Zooprofilattico Sperimentale del Mezzogiorno	Istituto Zooprofilattico Sperimentale del Mezzogiorno-U.O.C. Virologia	Maurizio Viscardi, Lorena Cardillo, Luigi Atripaldi,Giovanna Fusco, Esterina De Carlo, Antonio Limone
EPI_ISL_1173769, EPI_ISL_1173770, EPI_ISL_1173771, EPI_ISL_1173772, EPI_ISL_1173773, EPI_ISL_1173774, EPI_ISL_1173775, EPI_ISL_1173776, EPI_ISL_1173777, EPI_ISL_1173778, EPI_ISL_1173779, EPI_ISL_1173780, EPI_ISL_1173781, EPI_ISL_1173784, EPI_ISL_1173787, EPI_ISL_1173789, EPI_ISL_1173792, EPI_ISL_1173795, EPI_ISL_1173798, EPI_ISL_1173801, EPI_ISL_1173804, EPI_ISL_1173808, EPI_ISL_1173811, EPI_ISL_1173814, EPI_ISL_1173817, EPI_ISL_1173820, EPI_ISL_1173822, EPI_ISL_1173825, EPI_ISL_1173828, EPI_ISL_1173832, EPI_ISL_1173835, EPI_ISL_1173838, EPI_ISL_1173840, EPI_ISL_1173843, EPI_ISL_1173847, EPI_ISL_1173850, EPI_ISL_1173853, EPI_ISL_1173856, EPI_ISL_1173859, EPI_ISL_1173862, EPI_ISL_1173865, EPI_ISL_1173868, EPI_ISL_1173871, EPI_ISL_1173874, EPI_ISL_1173876, EPI_ISL_1173879, EPI_ISL_1173882, EPI_ISL_1173886, EPI_ISL_1173889, EPI_ISL_1173891, EPI_ISL_1173895, EPI_ISL_1173897, EPI_ISL_1173901, EPI_ISL_1173904, EPI_ISL_1173907, EPI_ISL_1173910, EPI_ISL_1173914, EPI_ISL_1173916, EPI_ISL_1173919, EPI_ISL_1173922, EPI_ISL_1173925, EPI_ISL_1173928, EPI_ISL_1173931, EPI_ISL_1173935, EPI_ISL_1173938, EPI_ISL_1173940, EPI_ISL_1173946, EPI_ISL_1173950, EPI_ISL_1173953, EPI_ISL_1173956, EPI_ISL_1173959, EPI_ISL_1173962			
see above	Università Federico II - Dipartimento di scienze mediche traslazionali - Napoli	TIGEM	Antonio Grimaldi Patrizia Annunziata Francesco Panariello Michele Cennamo Valentina Bouche Chiara Colantuono Lucio Di Filippo Mariano Fiorenza Anna Manfredi Marcello Salvi Giuseppe Portella Andrea Ballabio Davide Cacchiarelli
EPI_ISL_1176355	Laboratorio di Riferimento Regionale della Sicilia Occidentale per l'Emergenza COVID-19	Laboratorio di Riferimento Regionale della Sicilia Occidentale per l'Emergenza COVID-19	Fabio Tramuto, Carmelo Massimo Maida, Daniela Di Naro, Giulia Randazzo, Walter Mazzucco, Giorgio Graziano, Vincenzo Restivo, Claudio Costantino, Francesco Vitale
EPI_ISL_1178582	Department of Infectious Diseases, Istituto Superiore di Sanità, Rome, Italy; Università degli Studi di Perugia, Perugia, Italy	Istituto Superiore di Sanità (ISS)	Paola Stefanelli, Alessandra Lo Presti, Angela Di Martino, Stefano Fiore, Antonella Mencacci, Barbara Camilloni, Manuela Marra, Maria Carollo, Marco Crescenzi
EPI_ISL_1180141, EPI_ISL_1180142, EPI_ISL_1180143, EPI_ISL_1180144	Laboratorio Biologia Molecolare Sars Cov2 - UOC Laboratorio Analisi - Servizio Medicina di Laboratorio, Ospedale "San Francesco" - ATS-ASSL Nuoro	Laboratorio specialistico UOC Ematologia - Ospedale "San Francesco" - ATS-ASSL Nuoro	Giovanna Piras, Rosanna Asproni, Paolo Malune, Maria Itria Monne, Maura Fiamma, Angelo Domenico Palmas, Iana Lo Maglio, Giuseppe Mameli
EPI_ISL_1181344	Department of Infectious Diseases, Istituto Superiore di Sanità, Rome, Italy; AOR San Carlo, Potenza, Italy	Istituto Superiore di Sanità (ISS)	Paola Stefanelli, Angela Di Martino, Alessandra Lo Presti, Stefano Fiore, Antonio Picerno, Lopizzo Teresa, Anna Curci, Angela Menchise, Manuela Marra, Maria Carollo, Marco Crescenzi
EPI_ISL_1181347	Department of Infectious Diseases, Istituto Superiore di Sanità, Rome, Italy; U.O.S.D. Laboratorio Analisi Distretto 4 Ovest AULSS 9 Scaligera, Italy	Istituto Superiore di Sanità (ISS)	Paola Stefanelli, Angela Di Martino, Alessandra Lo Presti, Stefano Fiore, Maria Pompilia Visconti, Rosa Sforza, Manuela Marra, Maria Carollo, Marco Crescenzi
EPI_ISL_1181627	Department of Infectious Diseases, Istituto Superiore di Sanità, Rome, Italy; Azienda Usl Toscana sud est, Arezzo, Italy	Istituto Superiore di Sanità (ISS)	Paola Stefanelli, Angela Di Martino, Alessandra Lo Presti, Stefano Fiore, Agostino Ognibene, Manuela Marra, Maria Carollo, Marco Crescenzi
EPI_ISL_1181933, EPI_ISL_1181934, EPI_ISL_1181935, EPI_ISL_1181936, EPI_ISL_1181937, EPI_ISL_1181938, EPI_ISL_1181939, EPI_ISL_1181940, EPI_ISL_1181941, EPI_ISL_1181942, EPI_ISL_1181943, EPI_ISL_1181944, EPI_ISL_1181945			
see above	Azienda Ospedaliero - Universitaria di Modena Policlinico - Virologia e Microbiologia Molecolare	Istituto Zooprofilattico Sperimentale della Lombardia e dell'Emilia Romagna (IZSLER), Risk Analysis and Genomic Epidemiology Unit	Monica Pecorari, William Gennari, Giulia Fregni Serpini, Marina Morganti, Ilaria Menozzi, Erika Scaltriti, Stefano Pongolini
EPI_ISL_410545	INMI Lazzaro Spallanzani IRCCS	Laboratory of Virology, INMI Lazzaro Spallanzani IRCCS	Maria R. Capobianchi, Cesare E. M. Gruber, Martina Rueca, Barbara Bartolini, Francesco Messina, Emanuela Giombini, Francesca Colavita, Concetta Castilletti, Eleonora Lalle, Fabrizio Carletti, Emanuele Nicastri, Giuseppe Ippolito.
EPI_ISL_410546	INMI Lazzaro Spallanzani IRCCS	Laboratory of Virology, INMI Lazzaro Spallanzani IRCCS	Maria R. Capobianchi, Cesare E. M. Gruber, Martina Rueca, Fabrizio Carletti, Barbara Bartolini, Francesco Messina, Emanuela Giombini, Francesca Colavita, Concetta Castilletti, Eleonora Lalle, Emanuele Nicastri, Giuseppe Ippolito.
EPI_ISL_412973	Department of Infectious Diseases, Istituto Superiore di Sanità, Roma , Italy	Virology Laboratory, Scientific Department, Army Medical Center	Paola Stefanelli, Stefano Fiore, Antonella Marchi, Eleonora Benedetti, Concetta Fabiani, Giovanni Faggioni, Antonella Fortunato, Riccardo De Santis, Silvia Fillo, Anna Anselmo, Andrea Ciammaruconi, Stefano Palomba, Florigio Lista
EPI_ISL_412974	Department of Infectious Diseases, Istituto Superiore di Sanità, Rome, Italy	Virology Laboratory, Scientific Department, Army Medical Center	Paola Stefanelli, Stefano Fiore, Antonella Marchi, Eleonora Benedetti, Concetta Fabiani, Giovanni Faggioni, Antonella Fortunato, Silvia Fillo, Riccardo De Santis, Andrea Ciammaruconi, Giancarlo Petralito, Filippo Molinari, Florigio Lista
EPI_ISL_413489	Laboratorio di Microbiologia e Virologia, Università Vita-Salute San Raffaele, Milano	Laboratorio di Microbiologia e Virologia, Università Vita-Salute San Raffaele, Milano	R.A Diotti, E. Criscuolo, M. Castelli, V. Caputo, R. Ferrarese, M. Sampaolo, E. Boeri, I. Negri, V. Amato, G. Lo Raso, C. Di Resta, R. Burioni, M. Clementi, N. Mancini & N. Clementi
EPI_ISL_417418	Laboratory of Molecular Virology International Center fro Genetic Engineering and Biotechnology (ICGEB)	ARGO Open Lab Platform for Genome sequencing	Licastro D, Rajasekharan S, Dal Monego S, Segat L, D'Agaro P, Marcello A
EPI_ISL_417419, EPI_ISL_417421	Laboratory of Molecular Virology International Center for Genetic Engineering and Biotechnology (ICGEB)	ARGO Open Lab Platform for Genome sequencing	Licastro D, Rajasekharan S, Dal Monego S, Segat L, D'Agaro P, Marcello A
EPI_ISL_417423	Laboratory of Molecular Virology International Center for Genetic Engineering and Biotechnology (ICGEB)	ARGO Open Lab Platform for Genome sequencing	Licastro D, Rajasekharan, Dal Monego S, Segat L, D'Agaro P, Marcello A
EPI_ISL_417445, EPI_ISL_417446, EPI_ISL_417447	Laboratory of Infectious Diseases, Department of Biomedical and Clinical Sciences L. Sacco, University of Milan	Laboratory of Infectious Diseases, Department of Biomedical and Clinical Sciences L. Sacco, University of Milan	Gianguglielmo Zehender, Alessia Lai, Annalisa Bergna, Luca Meroni, Agostino Riva, Claudia Balotta, Maciej Tarkowski, Arianna Gabrieli, Dario Bernacchia, Stefano Rusconi, Giuliano Rizzardini, Spinello Antinori, Massimo Galli
EPI_ISL_417491	Virology Laboratory, Department of Biomedical Sciences and Public Health, University Politecnica delle Marche	Virology and Legal Medicine Laboratories, Department of Biomedical Sciences and Public Health, University Politecnica delle Marche	Bagnarelli,P., Caucci,S., Di Sante,L., Menzo,S., Alessandrini,F., Onofri,V., Turchi,C., Tagliabracci,A.

EPI_ISL_417921	INMI Lazzaro Spallanzani IRCCS	Laboratory of Virology, INMI Lazzaro Spallanzani IRCCS	Martina Rueca, Barbara Bartolini, Francesco Messina, Cesare E. M. Gruber, Emanuela Giombini, Maria R. Capobianchi, Fabrizio Carletti, Francesca Colavita, Concetta Castilletti, Eleonora Lalle, Daniele Lapa, Giuseppe Ippolito.
EPI_ISL_417922	INMI Lazzaro Spallanzani IRCCS	Laboratory of Virology, INMI Lazzaro Spallanzani IRCCS	Cesare E. M. Gruber, Martina Rueca, Barbara Bartolini, Francesco Messina, Emanuela Giombini, Maria R. Capobianchi, Fabrizio Carletti, Francesca Colavita, Concetta Castilletti, Eleonora Lalle, Daniele Lapa, Giuseppe Ippolito.
EPI_ISL_417923	INMI Lazzaro Spallanzani IRCCS	Laboratory of Virology, INMI Lazzaro Spallanzani IRCCS	Francesco Messina, Barbara Bartolini, Martina Rueca, Cesare E. M. Gruber, Emanuela Giombini, Maria R. Capobianchi, Fabrizio Carletti, Francesca Colavita, Concetta Castilletti, Eleonora Lalle, Daniele Lapa, Giuseppe Ippolito.
EPI_ISL_418255	Presidio Ospedaliero "S. Spirito" - PESCARA	Istituto Zooprofilattico Sperimentale dell'Abruzzo e Molise "G. Caporale"	Lorusso A, Marcacci M, Cammà C, Monaco F, Puglia I, Di Pasquale A, Rinaldi A, Mangone I, Savini G
EPI_ISL_418256	Ospedale "San Liberatore" di Atri	Istituto Zooprofilattico Sperimentale dell'Abruzzo e Molise "G. Caporale"	Lorusso A, Marcacci M, Di Domenico M, Puglia I, Curini V, Ancora M, Di Pasquale A, Rinaldi A, Mangone I, Cammà C, Savini G.
EPI_ISL_418257	Ospedale Civile Giuseppe Mazzini, Teramo	Istituto Zooprofilattico Sperimentale dell'Abruzzo e Molise "G.Caporale"	Lorusso A, Marcacci M, Di Domenico M, Puglia I, Curini V, Ancora M, Di Pasquale A, Rinaldi A, Mangone I, Cammà C, Savini G.
EPI_ISL_418258, EPI_ISL_418259	Presidio ospedaliero "Santo Spirito"	Istituto Zooprofilattico Sperimentale dell'Abruzzo e Molise "G. Caporale"	Lorusso A, Marcacci M, Di Domenico M, Puglia I, Curini V, Ancora M, Di Pasquale A, Rinaldi A, Mangone I, Cammà C, Savini G.
EPI_ISL_418260, EPI_ISL_418261	Ospedale Civile Giuseppe Mazzini	Istituto Zooprofilattico Sperimentale dell'Abruzzo e Molise "G. Caporale"	Lorusso A, Marcacci M, Di Domenico M, Puglia I, Curini V, Ancora M, Di Pasquale A, Rinaldi A, Mangone I, Cammà C, Savini G.
EPI_ISL_419254	INMI Lazzaro Spallanzani IRCCS	Laboratory of Virology, INMI Lazzaro Spallanzani IRCCS	Barbara Bartolini, Martina Rueca, Francesco Messina, Cesare E. M. Gruber, Emanuela Giombini, Maria R. Capobianchi, Fabrizio Carletti, Francesca Colavita, Concetta Castilletti, Eleonora Lalle, Daniele Lapa, Giuseppe Ippolito.
EPI_ISL_419255	INMI Lazzaro Spallanzani IRCCS	INMI Lazzaro Spallanzani IRCCS	Antonino Di Caro, Cesare E. M. Gruber, Martina Rueca, Barbara Bartolini, Francesco Messina, Emanuela Giombini, Maria R. Capobianchi, Fabrizio Carletti, Francesca Colavita, Concetta Castilletti, Eleonora Lalle, Daniele Lapa, Giuseppe Ippolito.
EPI_ISL_420563	Ospedale Civile Giuseppe Mazzini	Istituto Zooprofilattico Sperimentale dell'Abruzzo e Molise "G. Caporale"	Lorusso A, Marcacci M, Di Domenico M, Ancora M, Curini V, Mangone I, Rinaldi A, Di Pasquale A, Cammà C, Puglia I, Savini G
EPI_ISL_420564	Ospedale Civile Castel Di Sangro	Istituto Zooprofilattico Sperimentale dell'Abruzzo e Molise "G.Caporale"	Lorusso A, Marcacci M, Di Domenico M, Ancora M, Curini V, Mangone I, Rinaldi A, Di Pasquale A, Cammà C, Puglia I, Savini G
EPI_ISL_420565	Ospedale Civile Giuseppe Mazzini	Istituto Zooprofilattico Sperimentale dell'Abruzzo e Molise "G.Caporale"	Lorusso A, Marcacci M, Di Domenico M, Ancora M, Curini V, Mangone I, Rinaldi A, Di Pasquale A, Cammà C, Puglia I, Savini G
EPI_ISL_420566, EPI_ISL_420567	Ospedale Regionale San Salvatore	Istituto Zooprofilattico Sperimentale dell'Abruzzo e Molise "G.Caporale"	Lorusso A, Marcacci M, Di Domenico M, Ancora M, Curini V, Mangone I, Rinaldi A, Di Pasquale A, Cammà C, Puglia I, Savini G
EPI_ISL_420568, EPI_ISL_420569, EPI_ISL_420583, EPI_ISL_420592	Ospedale Civile Giuseppe Mazzini	Istituto Zooprofilattico Sperimentale dell'Abruzzo e Molise "G.Caporale"	Lorusso A, Marcacci M, Di Domenico M, Ancora M, Curini V, Mangone I, Rinaldi A, Di Pasquale A, Cammà C, Puglia I, Savini G
EPI_ISL_422437, EPI_ISL_422438	ULSS9 Distretto di Bussolengo	Istituto Zooprofilattico Sperimentale delle Venezie	Adelaide Milani, Alessia Schivo, Annalisa Salviato, Erika Giorgia Quaranta, Ambra Pastori, Bianca Zecchin, Alice Fusaro, Isabella Monne, Calogero Terregino, Antonia Ricci
EPI_ISL_424342	INMI Lazzaro Spallanzani IRCCS	Laboratory of Virology, INMI Lazzaro Spallanzani IRCCS	Concetta Castilletti, Barbara Bartolini, Martina Rueca, Cesare Ernesto Maria Gruber, Francesco Messina, Fabrizio Carletti, Eleonora Lalle, Licia Bordi, Giulia Matusali, Francesca Colavita, Maria Rosaria Capobianchi, Francesco Vairo, Giuseppe Ippolito, Antonino Di Caro
EPI_ISL_424343	INMI Lazzaro Spallanzani IRCCS	Laboratory of Virology, INMI Lazzaro Spallanzani IRCCS	Fabrizio Carletti, Barbara Bartolini, Martina Rueca, Cesare Ernesto Maria Gruber, Francesco Messina, Eleonora Lalle, Licia Bordi, Giulia Matusali, Francesca Colavita, Maria Rosaria Capobianchi, Concetta Castilletti, Francesco Vairo, Giuseppe Ippolito, Antonino Di Caro
EPI_ISL_424344	INMI Lazzaro Spallanzani IRCCS	Laboratory of Virology, INMI Lazzaro Spallanzani IRCCS	Eleonora Lalle, Barbara Bartolini, Martina Rueca, Cesare Ernesto Maria Gruber, Francesco Messina, Fabrizio Carletti, Licia Bordi, Giulia Matusali, Francesca Colavita, Maria Rosaria Capobianchi, Concetta Castilletti, Francesco Vairo, Giuseppe Ippolito, Antonino Di Caro
EPI_ISL_428853	Laboratory of Molecular Virology International Center for Genetic Engineering and Biotechnology (ICGEB)	ARGO Open Lab Platform for Genome Sequencing	Licastro D, Rajasekharan S, Dal Monego S, Segat L, D'Agaro P, Marcello A
EPI_ISL_428854	Laboratory of Molecular Virology International Center for Genetic Engineering and Biotechnology (ICGEB)	ARGO Open Lab Platform for Genome sequencing	Licastro D, Rajasekharan S, Dal Monego S, Segat L, D'Agaro P, Marcello A
EPI_ISL_429226, EPI_ISL_429227	Presidio Ospedaliero Santo Spirito	Istituto Zooprofilattico Sperimentale dell'Abruzzo e Molise "G. Caporale"	Lorusso A, Marcacci M, Di Domenico M, Ancora M, Curini V, Mangone I, Rinaldi A, Di Pasquale A, Camma C, Puglia I, Savini G
EPI_ISL_429228	Ospedale Civile Giuseppe Mazzini	Istituto Zooprofilattico Sperimentale dell'Abruzzo e Molise "G. Caporale"	Lorusso A, Marcacci M, Di Domenico M, Ancora M, Curini V, Mangone I, Rinaldi A, Di Pasquale A, Camma C, Puglia I, Savini G
EPI_ISL_429229	Ospedale Regionale San Salvatore	Istituto Zooprofilattico Sperimentale dell'Abruzzo e Molise "G. Caporale"	Lorusso A, Marcacci M, Di Domenico M, Ancora M, Curini V, Mangone I, Rinaldi A, Di Pasquale A, Camma C, Puglia I, Savini G
EPI_ISL_429230, EPI_ISL_429231, EPI_ISL_429232, EPI_ISL_429233, EPI_ISL_429234, EPI_ISL_429235	Ospedale Civile Giuseppe Mazzini	Istituto Zooprofilattico Sperimentale dell'Abruzzo e Molise "G. Caporale"	Lorusso A, Marcacci M, Di Domenico M, Ancora M, Curini V, Mangone I, Rinaldi A, Di Pasquale A, Camma C, Puglia I, Savini G
EPI_ISL_429236	Ospedale Civile S. Liberatore di Atri	Istituto Zooprofilattico Sperimentale dell'Abruzzo e Molise "G. Caporale"	Lorusso A, Marcacci M, Di Domenico M, Ancora M, Curini V, Mangone I, Rinaldi A, Di Pasquale A, Camma C, Puglia I, Savini G
EPI_ISL_435145	Ospedale Civile Giuseppe Mazzini	Istituto Zooprofilattico Sperimentale dell'Abruzzo e Molise "G.Caporale"	Lorusso A, Marcacci M, Di Domenico M, Ancora M, Curini V, Mangone I, Rinaldi A, Di Pasquale A, Cammà C, Puglia I, Savini G
EPI_ISL_435146, EPI_ISL_435147	Villa Serena del Dr. Leonardo Petruzzi	Istituto Zooprofilattico Sperimentale dell'Abruzzo e Molise "G.Caporale"	Lorusso A, Marcacci M, Di Domenico M, Ancora M, Curini V, Mangone I, Rinaldi A, Di Pasquale A, Cammà C, Puglia I, Savini G
EPI_ISL_435148	Ospedale SS Annunziata	Istituto Zooprofilattico Sperimentale dell'Abruzzo e Molise "G.Caporale"	Lorusso A, Marcacci M, Di Domenico M, Ancora M, Curini V, Mangone I, Rinaldi A, Di Pasquale A, Cammà C, Puglia I, Savini G
EPI_ISL_435149	SERVIZIO DI IGIENE E SANITÀ PUBBLICA ASL Teramo	Istituto Zooprofilattico Sperimentale dell'Abruzzo e Molise "G.Caporale"	Lorusso A, Marcacci M, Di Domenico M, Ancora M, Curini V, Mangone I, Rinaldi A, Di Pasquale A, Cammà C, Puglia I, Savini G
EPI_ISL_435150, EPI_ISL_435151	Ospedale SS Annunziata	Istituto Zooprofilattico Sperimentale dell'Abruzzo e Molise "G.Caporale"	Lorusso A, Marcacci M, Di Domenico M, Ancora M, Curini V, Mangone I, Rinaldi A, Di Pasquale A, Cammà C, Puglia I, Savini G
EPI_ISL_435152	Servizio di Igiene, Epidemiologia e Sanità Pubblica (SIESP) Avezzano	Istituto Zooprofilattico Sperimentale dell'Abruzzo e Molise "G.Caporale"	Lorusso A, Marcacci M, Di Domenico M, Ancora M, Curini V, Mangone I, Rinaldi A, Di Pasquale A, Cammà C, Puglia I, Savini G
EPI_ISL_435153, EPI_ISL_435154, EPI_ISL_435155	SERVIZIO DI IGIENE E SANITÀ PUBBLICA ASL Teramo	Istituto Zooprofilattico Sperimentale dell'Abruzzo e Molise "G.Caporale"	Lorusso A, Marcacci M, Di Domenico M, Ancora M, Curini V, Mangone I, Rinaldi A, Di Pasquale A, Cammà C, Puglia I, Savini G
EPI_ISL_436718	Ospedale Regionale San Salvatore	Istituto Zooprofilattico Sperimentale dell'Abruzzo e Molise "G.Caporale"	Lorusso A, Marcacci M, Di Domenico M, Ancora M, Curini V, Mangone I, Rinaldi A, Di Pasquale A, Cammà C, Puglia I, Savini G
EPI_ISL_436719, EPI_ISL_436720, EPI_ISL_436721, EPI_ISL_436722	Ospedale Civile S. Liberatore di Atri	Istituto Zooprofilattico Sperimentale dell'Abruzzo e Molise "G.Caporale"	Lorusso A, Marcacci M, Di Domenico M, Ancora M, Curini V, Mangone I, Rinaldi A, Di Pasquale A, Cammà C, Puglia I, Savini G

EPI_ISL_436723	Ospedale Civile Giuseppe Mazzini	Istituto Zooprofilattico Sperimentale dell'Abruzzo e Molise "G.Caporale"	Lorusso A, Marcacci M, Di Domenico M, Ancora M, Curini V, Mangone I, Rinaldi A, Di Pasquale A, Cammà C, Puglia I, Savini G
EPI_ISL_436724	Ospedale Civile S. Liberatore di Atri	Istituto Zooprofilattico Sperimentale dell'Abruzzo e Molise "G.Caporale"	Lorusso A, Marcacci M, Di Domenico M, Ancora M, Curini V, Mangone I, Rinaldi A, Di Pasquale A, Cammà C, Puglia I, Savini G
EPI_ISL_436725	RSA/RP Villa San Giovanni - Gruppo Edos	Istituto Zooprofilattico Sperimentale dell'Abruzzo e Molise "G.Caporale"	Lorusso A, Marcacci M, Di Domenico M, Ancora M, Curini V, Mangone I, Rinaldi A, Di Pasquale A, Cammà C, Puglia I, Savini G
EPI_ISL_436726, EPI_ISL_436727, EPI_ISL_436728, EPI_ISL_436729	SERVIZIO DI IGIENE E SANITÀ PUBBLICA ASL Teramo	Istituto Zooprofilattico Sperimentale dell'Abruzzo e Molise "G.Caporale"	Lorusso A, Marcacci M, Di Domenico M, Ancora M, Curini V, Mangone I, Rinaldi A, Di Pasquale A, Cammà C, Puglia I, Savini G
EPI_ISL_436730	Servizio di igiene epidemiologia e sanità pubblica (Siesp) Chieti	Istituto Zooprofilattico Sperimentale dell'Abruzzo e Molise "G.Caporale"	Lorusso A, Marcacci M, Di Domenico M, Ancora M, Curini V, Mangone I, Rinaldi A, Di Pasquale A, Cammà C, Puglia I, Savini G
EPI_ISL_436731, EPI_ISL_436732	Ospedale Civile S. Liberatore di Atri	Istituto Zooprofilattico Sperimentale dell'Abruzzo e Molise "G.Caporale"	Lorusso A, Marcacci M, Di Domenico M, Ancora M, Curini V, Mangone I, Rinaldi A, Di Pasquale A, Cammà C, Puglia I, Savini G
EPI_ISL_451298	Laboratory of Virology, INMI Lazzaro Spallanzani IRCCS	Laboratory of Virology, INMI Lazzaro Spallanzani IRCCS	Cesare E.M. Gruber, Martina Rueca, Barbara Bartolini, Francesco Messina, Antonino Di Caro, Maria R. Capobianchi, Giuseppe Ippolito
EPI_ISL_451299	Laboratory of Virology, INMI Lazzaro Spallanzani IRCCS	Laboratory of Virology, INMI Lazzaro Spallanzani IRCCS	Martina Rueca, Cesare E.M. Gruber, Barbara Bartolini, Francesco Messina, Antonino Di Caro, Maria R. Capobianchi, Giuseppe Ippolito
EPI_ISL_451300	Laboratory of Virology, INMI Lazzaro Spallanzani IRCCS	Laboratory of Virology, INMI Lazzaro Spallanzani IRCCS	Cesare E.M. Gruber, Martina Rueca, Barbara Bartolini, Francesco Messina, Antonino Di Caro, Maria R. Capobianchi, Giuseppe Ippolito
EPI_ISL_451301	Laboratory of Virology, INMI Lazzaro Spallanzani IRCCS	Laboratory of Virology, INMI Lazzaro Spallanzani IRCCS	Martina Rueca, Cesare E.M. Gruber, Barbara Bartolini, Francesco Messina, Antonino Di Caro, Maria R. Capobianchi, Giuseppe Ippolito
EPI_ISL_451302	Laboratory of Virology, INMI Lazzaro Spallanzani IRCCS	Laboratory of Virology, INMI Lazzaro Spallanzani IRCCS	Cesare E.M. Gruber, Martina Rueca, Barbara Bartolini, Francesco Messina, Antonino Di Caro, Maria R. Capobianchi, Giuseppe Ippolito
EPI_ISL_451303	Laboratory of Virology, INMI Lazzaro Spallanzani IRCCS	Laboratory of Virology, INMI Lazzaro Spallanzani IRCCS	Martina Rueca, Cesare E.M. Gruber, Barbara Bartolini, Francesco Messina, Antonino Di Caro, Maria R. Capobianchi, Giuseppe Ippolito
EPI_ISL_451304	Laboratory of Virology, INMI Lazzaro Spallanzani IRCCS	Laboratory of Virology, INMI Lazzaro Spallanzani IRCCS	Cesare E.M. Gruber, Martina Rueca, Barbara Bartolini, Francesco Messina, Antonino Di Caro, Maria R. Capobianchi, Giuseppe Ippolito
EPI_ISL_451305	Laboratory of Virology, INMI Lazzaro Spallanzani IRCCS	Laboratory of Virology, INMI Lazzaro Spallanzani IRCCS	Martina Rueca, Cesare E.M. Gruber, Barbara Bartolini, Francesco Messina, Antonino Di Caro, Maria R. Capobianchi, Giuseppe Ippolito
EPI_ISL_451306	Molecular Virology Unit, Fondazione IRCCS Policlinico San Matteo , Pavia	Laboratory of Virology, INMI Lazzaro Spallanzani IRCCS	Antonio Piralla, Fausto Baldanti, Martina Rueca, Antonino Di Caro, Maria R. Capobianchi, Cesare E.M. Gruber, Barbara Bartolini
EPI_ISL_451307	Molecular Virology Unit, Fondazione IRCCS Policlinico San Matteo , Pavia	Laboratory of Virology, INMI Lazzaro Spallanzani IRCCS	Fausto Baldanti, Antonio Piralla, Antonino Di Caro, Cesare E.M. Gruber, Martina Rueca, Barbara Bartolini, Maria R. Capobianchi
EPI_ISL_451308	Molecular Virology Unit, Fondazione IRCCS Policlinico San Matteo , Pavia	Laboratory of Virology, INMI Lazzaro Spallanzani IRCCS	Antonio Piralla, Fausto Baldanti, Maria R. Capobianchi, Cesare E.M. Gruber, Martina Rueca, Barbara Bartolini, Antonino Di Caro
EPI_ISL_451309	Molecular Virology Unit, Fondazione IRCCS Policlinico San Matteo , Pavia	Laboratory of Virology, INMI Lazzaro Spallanzani IRCCS	Fausto Baldanti, Antonio Piralla, Cesare E.M. Gruber, Maria R. Capobianchi, Antonino Di Caro, Martina Rueca, Barbara Bartolini
EPI_ISL_451961	Istituto Zooprofilattico Sperimentale Puglia e Basilicata; Dipartimento di Bioscienze, Biotecnologie e Biofarmaceutica dell'Università degli Studi di Bari "A.Moro"; Istituto di Biomembrane. Bioenergetica e Biotecnologie Molecolari del Consiglio Nazionale delle Ricerche di Bari	Beaconlab (Bioinformatics Evolution and Comparative Genomics lab), Dept of Biosciences, University of Milan	Parisi A.,Pesole G., Manzari C., Chiara M.
EPI_ISL_451962	Istituto Zooprofilattico Sperimentale Puglia e Basilicata; Dipartimento di Bioscienze, Biotecnologie e Biofarmaceutica dell'Università degli Studi di Bari "A.Moro"; Istituto di Biomembrane. Bioenergetica e Biotecnologie Molecolari del Consiglio Nazionale delle Ricerche di Bari	Beaconlab (Bioinformatics, Evolution and Comparative Genomics lab), Dept of Biosciences, University on Milan	Parisi A.,Pesole G., Manzari C., Chiara M.
EPI_ISL_452181, EPI_ISL_452182, EPI_ISL_452183, EPI_ISL_452184, EPI_ISL_452185, EPI_ISL_452186, EPI_ISL_452187, EPI_ISL_452188, EPI_ISL_452189	ULSS9 Distretto di Bussolengo	Istituto Zooprofilattico Sperimentale delle Venezie	Adelaide Milani, Alessia Schivo, Annalisa Salviato, Erika Giorgia Quaranta, Ambra Pastori, Bianca Zecchin, Alice Fusaro, Isabella Monne, Calogero Terregino, Antonia Ricci
EPI_ISL_452190, EPI_ISL_452191	ULSS9 Distretto di San Bonifacio	Istituto Zooprofilattico Sperimentale delle Venezie	Adelaide Milani, Alessia Schivo, Annalisa Salviato, Erika Giorgia Quaranta, Ambra Pastori, Bianca Zecchin, Alice Fusaro, Isabella Monne, Calogero Terregino, Antonia Ricci
EPI_ISL_454733	Department of Medical, Biotechnologies University of Siena	Department of Medical, Biotechnologies University of Siena	Cusi,M.G., Pinzauti,D., Gandolfo,C., Anichini,G., Pozzi,G. and Santoro,F.
EPI_ISL_457699, EPI_ISL_457700	Department of Infectious Diseases, Istituto Superiore di Sanità, Roma , Italy	Army Medical and Veterinary Research Center	Paola Stefanelli, Alessandra Lo Presti, Stefano Fiore, Antonella Marchi, Eleonora Benedetti, Concetta Fabiani Silvia Fillo, Giovanni Faggioni, Riccardo De Sanctis, Antonella Fortunato, Anna Anselmo, Francesco Giordani, Vanessa Vera Fain, Nino D'Amore, Florigio Lista
EPI_ISL_457721, EPI_ISL_457724, EPI_ISL_457728, EPI_ISL_457732, EPI_ISL_457736, EPI_ISL_457749	Department of Infectious Diseases, Istituto Superiore di Sanità, Roma , Italy	Army Medical and Veterinary Research Center	Paola Stefanelli, Alessandra Lo Presti, Stefano Fiore, Antonella Marchi, Eleonora Benedetti, Concetta Fabiani Silvia Fillo, Giovanni Faggioni, Riccardo De Sanctis, Antonella Fortunato, Anna Anselmo, Francesco Giordani, Vanessa Vera Fain, Nino D'Amore, Florigio Lista
EPI_ISL_457825	Army Medical Research Center - Scientific Department	Army Medical and Veterinary Research Center	Silvia Fillo, Giovanni Faggioni, Riccardo De Sanctis, Antonella Fortunato, Anna Anselmo, Francesco Giordani, Vanessa Vera Fain, Nino D'Amore, Florigio Lista
EPI_ISL_457826	Army Medical Center - Scientific Department	Army Medical and Veterinary Research Center	Silvia Fillo, Giovanni Faggioni, Riccardo De Sanctis, Antonella Fortunato, Anna Anselmo, Francesco Giordani, Vanessa Vera Fain, Nino D'Amore, Florigio Lista
EPI_ISL_458084	Laboratorio Biologia Molecolare Sars Cov2 - UOC Laboratorio Analisi - Servizio Medicina di Laboratorio, Ospedale "San Francesco" - ATS-ASSL Nuoro	Laboratorio specialistico UOC Ematologia - Ospedale "San Francesco" - ATS-ASSL Nuoro	Piras Giovanna, Fancello Tatiana, Asproni Rosanna, Fiamma Maura, Monne Maria Itria, Toja Alessandro, Sanna Filomena, Floris Anna Rita, Sulis Vincenzo, Palmas Angelo Domenico, Casu Gavino, Lo Maglio Iana, Mameli Giuseppe.
EPI_ISL_458085	Laboratorio Biologia Molecolare Sars Cov2 - UOC Laboratorio Analisi - Servizio Medicina di Laboratorio , Ospedale "San Francesco" - ATS- ASSL Nuoro	Laboratorio specialistico UOC Ematologia - Ospedale "San Francesco" - ATS-ASSL Nuoro	Piras Giovanna, Fancello Tatiana, Asproni Rosanna, Fiamma Maura, Monne Maria Itria, Toja Alessandro, Sanna Filomena, Floris Anna Rita, Sulis Vincenzo, Palmas Angelo Domenico, Casu Gavino, Lo Maglio Iana, Mameli Giuseppe.
EPI_ISL_460079	Molecular Virology Unit, Fondazione IRCCS Policlinico San Matteo , Pavia	Laboratory of Virology, INMI Lazzaro Spallanzani IRCCS	Barbara Bartolini, Cesare E.M. Gruber, Maria R. Capobianchi, Martina Rueca, Antonio Piralla, Fausto Baldanti, Antonino Di Caro
EPI_ISL_460080	Molecular Virology Unit, Fondazione IRCCS Policlinico San Matteo , Pavia	Laboratory of Virology, INMI Lazzaro Spallanzani IRCCS	Antonio Piralla, Barbara Bartolini, Fausto Baldanti, Martina Rueca, Antonino Di Caro, Cesare E.M. Gruber, Maria R. Capobianchi
EPI_ISL_460081	Molecular Virology Unit, Fondazione IRCCS Policlinico San Matteo , Pavia	Laboratory of Virology, INMI Lazzaro Spallanzani IRCCS	Fausto Baldanti, Martina Rueca, Antonio Piralla, Antonino Di Caro, Maria R. Capobianchi, Cesare E.M. Gruber, Barbara Bartolini
EPI_ISL_460082	Molecular Virology Unit, Fondazione IRCCS Policlinico San Matteo , Pavia	Laboratory of Virology, INMI Lazzaro Spallanzani IRCCS	Martina Rueca, Cesare E.M. Gruber, Antonio Piralla, Antonino Di Caro, Barbara Bartolini, Maria R. Capobianchi, Fausto Baldanti
EPI_ISL_460083	Molecular Virology Unit, Fondazione IRCCS Policlinico San Matteo , Pavia	Laboratory of Virology, INMI Lazzaro Spallanzani IRCCS	Martina Rueca, Antonino Di Caro, Cesare E.M. Gruber, Barbara Bartolini, Fausto Baldanti, Antonio Piralla, Maria R. Capobianchi

EPI_ISL_460084	Molecular Virology Unit, Fondazione IRCCS Policlinico San Matteo , Pavia	Laboratory of Virology, INMI Lazzaro Spallanzani IRCCS	Fausto Baldanti, Antonio Piralla, Martina Rueca, Barbara Bartolini, Maria R. Capobianchi, Cesare E.M. Gruber, Antonino Di Caro
EPI_ISL_460085	Molecular Virology Unit, Fondazione IRCCS Policlinico San Matteo , Pavia	Laboratory of Virology, INMI Lazzaro Spallanzani IRCCS	Cesare E.M. Gruber, Maria R. Capobianchi, Barbara Bartolini, Fausto Baldanti, Martina Rueca, Antonio Piralla, Antonino Di Caro
EPI_ISL_460086	Molecular Virology Unit, Fondazione IRCCS Policlinico San Matteo , Pavia	Laboratory of Virology, INMI Lazzaro Spallanzani IRCCS	Maria R. Capobianchi, Fausto Baldanti, Antonio Piralla, Antonino Di Caro, Barbara Bartolini, Cesare E.M. Gruber, Martina Rueca
EPI_ISL_460087	Molecular Virology Unit, Fondazione IRCCS Policlinico San Matteo , Pavia	Laboratory of Virology, INMI Lazzaro Spallanzani IRCCS	Cesare E.M. Gruber, Maria R. Capobianchi, Martina Rueca, Barbara Bartolini, Antonino Di Caro, Antonio Piralla, Fausto Baldanti
EPI_ISL_460088	Molecular Virology Unit, Fondazione IRCCS Policlinico San Matteo , Pavia	Laboratory of Virology, INMI Lazzaro Spallanzani IRCCS	Martina Rueca, Barbara Bartolini, Fausto Baldanti, Maria R. Capobianchi, Cesare E.M. Gruber, Antonino Di Caro, Antonio Piralla
EPI_ISL_460089	Molecular Virology Unit, Fondazione IRCCS Policlinico San Matteo , Pavia	Laboratory of Virology, INMI Lazzaro Spallanzani IRCCS	Antonino Di Caro, Barbara Bartolini, Martina Rueca, Cesare E.M. Gruber, Antonio Piralla, Fausto Baldanti, Maria R. Capobianchi
EPI_ISL_460090	Molecular Virology Unit, Fondazione IRCCS Policlinico San Matteo , Pavia	Laboratory of Virology, INMI Lazzaro Spallanzani IRCCS	Antonio Piralla, Cesare E.M. Gruber, Antonino Di Caro, Maria R. Capobianchi, Martina Rueca, Barbara Bartolini, Fausto Baldanti
EPI_ISL_460091	Molecular Virology Unit, Fondazione IRCCS Policlinico San Matteo , Pavia	Laboratory of Virology, INMI Lazzaro Spallanzani IRCCS	Antonino Di Caro, Antonio Piralla, Martina Rueca, Fausto Baldanti, Barbara Bartolini, Maria R. Capobianchi, Cesare E.M. Gruber
EPI_ISL_460092	Molecular Virology Unit, Fondazione IRCCS Policlinico San Matteo , Pavia	Laboratory of Virology, INMI Lazzaro Spallanzani IRCCS	Cesare E.M. Gruber, Martina Rueca, Maria R. Capobianchi, Antonino Di Caro, Antonio Piralla, Barbara Bartolini, Fausto Baldanti
EPI_ISL_460093	Molecular Virology Unit, Fondazione IRCCS Policlinico San Matteo , Pavia	Laboratory of Virology, INMI Lazzaro Spallanzani IRCCS	Maria R. Capobianchi, Antonio Piralla, Antonino Di Caro, Fausto Baldanti, Martina Rueca, Cesare E.M. Gruber, Barbara Bartolini
EPI_ISL_460094	Molecular Virology Unit, Fondazione IRCCS Policlinico San Matteo , Pavia	Laboratory of Virology, INMI Lazzaro Spallanzani IRCCS	Barbara Bartolini, Maria R. Capobianchi, Antonino Di Caro, Antonio Piralla, Cesare E.M. Gruber, Martina Rueca, Fausto Baldanti
EPI_ISL_460095	Molecular Virology Unit, Fondazione IRCCS Policlinico San Matteo , Pavia	Laboratory of Virology, INMI Lazzaro Spallanzani IRCCS	Barbara Bartolini, Antonino Di Caro, Fausto Baldanti, Cesare E.M. Gruber, Maria R. Capobianchi, Martina Rueca, Antonio Piralla
EPI_ISL_468914, EPI_ISL_469016, EPI_ISL_469018, EPI_ISL_469019, EPI_ISL_469020, EPI_ISL_469021, EPI_ISL_469022	Istituto Zooprofilattico Sperimentale Puglia e Basilicata; Dipartimento di Bioscienze, Biotecnologie e Biofarmaceutica dell'Università degli Studi di Bari "A.Moro"; Istituto di Biomembrane. Bioenergetica e Biotecnologie Molecolari del Consiglio Nazionale delle Ricerche di Bari	Beaconlab (Bioinformatics, Evolution and Comparative Genomics lab), Dept of Biosciences, University on Milan	Parisi A.,Pesole G., Manzari C., Chiara M.
EPI_ISL_469023	Istituto Zooprofilattico Sperimentale Puglia e Basilicata; Dipartimento di Bioscienze, Biotecnologie e Biofarmaceutica dell'Università degli Studi di Bari "A.Moro"; Istituto di Biomembrane. Bioenergetica e Biotecnologie Molecolari del Consiglio Nazionale delle Ricerche di Bari	Beaconlab (Bioinformatics, Evolution and Comparative Genomics lab), Dept of Biosciences, University on Milan	Parisi A.,Pesole G., Manzari C., Chiara M
EPI_ISL_477193, EPI_ISL_477194	Istituto Zooprofilattico Sperimentale Puglia e Basilicata;	Beaconlab (Bioinformatics, Evolution and Comparative Genomics lab), Dept of Biosciences, University on Mila	Parisi A.,Pesole G., Manzari C., Chiara M.
EPI_ISL_477195, EPI_ISL_477196, EPI_ISL_477197, EPI_ISL_477198, EPI_ISL_477199, EPI_ISL_477200, EPI_ISL_477201	Istituto Zooprofilattico Sperimentale Puglia e Basilicata;	Beaconlab (Bioinformatics, Evolution and Comparative Genomics lab), Dept of Biosciences, University on Milan	Parisi A.,Pesole G., Manzari C., Chiara M.
EPI_ISL_477202, EPI_ISL_477203	Istituto Zooprofilattico Sperimentale Puglia e Basilicata;	Beaconlab (Bioinformatics, Evolution and Comparative Genomics lab), Dept of Biosciences, University on Mila	Parisi A.,Pesole G., Manzari C., Chiara M.
EPI_ISL_477204	Prof. Massimo Zollo CEINGE TASK-FORCE COVID19 - Regione Campania	Prof. Massimo Zollo CEINGE TASK-FORCE COVID19 - Regione Campania	Veronica Ferrucci1,2, Dae young Kong8, Fatemeh asadzadeh1,2, Laura Marrone1,2, Roberto Siciliano1,2, Rino Cerino3, Giovanna Fusco3, Marika Comegna1,2, Angelo Boccia2, Maurizio Viscardi3, Giorgia Borriello3, Sergio Brandi3, Claudia Tiberio4, Luigi Atripaldi4, Giovanni Paolella1,2, Giuseppe Castaldo1,2, Stefano Pascarella4, Martina Bianchi4, Lorenzo Chiariotti1,2, Jae Myun Lee5, Jae Ho Jung6, Kyong Seop Yun7, Hong Yeoul Kim 7,8* and Massimo Zollo1,2* 1 CEINGE Biotecnologie Avanzate, Naples, Italia 2 Dipartimento di Medicina Molecolare e Biotecnologie Mediche DMMBM University of Naples Federico II, Italia 3 Istituto Zooprofilattico Sperimentale del Mezzogiorno, Naples, Italia 4 -U.O.C. di Patologia Clinica Ospedale D. Cotugno, Azienda Sanitaria Ospedali dei Colli, Naples, Italy. 5 Università La Sapienza di Roma, Italia 6 Department of Microbiology, Yonsei University College of Medicine, Seoul, Korea 7 Department of Surgery, Yonsei University College of Medicine, Seoul, Korea 8 Haim bio co., Ltd, , Indust
EPI_ISL_479616, EPI_ISL_479617	Laboratory of Molecular Virology of the International Centre for Genetic Engineering and Biotechnology (ICGEB)	ARGO Open Lab Platform for Genome Sequencing	Licastro, D, Rajasekharan S, Dal Monego S, Segat L, D'Agaro P, Salton F, Confalonieri P, Confalonieri M Marcello A
EPI_ISL_479618, EPI_ISL_479619, EPI_ISL_479790, EPI_ISL_479791	Laboratory of Molecular Virology of the International Centre for Genetic Engineering and Biotechnology (ICGEB)	ARGO Open Lab Platform for Genome Sequencing	Licastro, D, Rajasekharan S, Dal Monego S, Segat L, D'Agaro P, Salton F, Confalonieri P, Confalonieri M, Marcello A
EPI_ISL_486646, EPI_ISL_486647	Microbiology, Virology and Biemergency Laboratory-ASST FBF Sacco	Microbiology, Virology and Biemergency Laboratory-ASST FBF Sacco	Mancon A, Comandatore F, Romeri F, Micheli V, Rimoldi SG
EPI_ISL_486648	Microbiology, Virology and Biemergency Laboratory-ASST FBF Sacco	Microbiology, Virology and Biemergency Laboratory-ASST FBF Sacco	Micheli V, Comandatore F, Romeri F, Mancon A, Rimoldi SG
EPI_ISL_486649	Microbiology, Virology and Biemergency Laboratory-ASST FBF Sacco	Microbiology, Virology and Biemergency Laboratory-ASST FBF Sacco	Rimoldi SG, Comandatore F, Romeri F, Mancon A, Micheli V
EPI_ISL_486650	Microbiology, Virology and Biemergency Laboratory-ASST FBF Sacco	Microbiology, Virology and Biemergency Laboratory-ASST FBF Sacco	Romeri F, Comandatore F, Mancon A, Micheli V, Rimoldi SG
EPI_ISL_486651	Microbiology, Virology and Biemergency Laboratory-ASST FBF Sacco	Microbiology, Virology and Biemergency Laboratory-ASST FBF Sacco	Mancon A, Comandatore F, Romeri F, Micheli V, Rimoldi SG
EPI_ISL_486652	Microbiology, Virology and Biemergency Laboratory-ASST FBF Sacco	Microbiology, Virology and Biemergency Laboratory-ASST FBF Sacco	Micheli V, Comandatore F, Romeri F, Mancon A, Rimoldi SG
EPI_ISL_486653	Microbiology, Virology and Biemergency Laboratory-ASST FBF Sacco	Microbiology, Virology and Biemergency Laboratory-ASST FBF Sacco	Rimoldi SG, Comandatore F, Romeri F, Mancon A, Micheli V
EPI_ISL_486654	Microbiology, Virology and Biemergency Laboratory-ASST FBF Sacco	Microbiology, Virology and Biemergency Laboratory-ASST FBF Sacco	Romeri F, Comandatore F, Mancon A, Micheli V, Rimoldi SG
EPI_ISL_486655	Microbiology, Virology and Biemergency Laboratory-ASST FBF Sacco	Microbiology, Virology and Biemergency Laboratory-ASST FBF Sacco	Mancon A, Comandatore F, Romeri F, Micheli V, Rimoldi SG
EPI_ISL_486656	Microbiology, Virology and Biemergency Laboratory-ASST FBF Sacco	Microbiology, Virology and Biemergency Laboratory-ASST FBF Sacco	Micheli V, Comandatore F, Romeri F, Mancon A, Rimoldi SG

EPI_ISL_486657	Microbiology, Virology and Biemergency Laboratory-ASST FBF Sacco	Microbiology, Virology and Biemergency Laboratory-ASST FBF Sacco	Rimoldi SG, Comandatore F, Romeri F, Mancon A, Micheli V
EPI_ISL_486658	Microbiology, Virology and Biemergency Laboratory-ASST FBF Sacco	Microbiology, Virology and Biemergency Laboratory-ASST FBF Sacco	Romeri F, Comandatore F, Mancon A, Micheli V, Rimoldi SG
EPI_ISL_486659	Microbiology, Virology and Biemergency Laboratory-ASST FBF Sacco	Microbiology, Virology and Biemergency Laboratory-ASST FBF Sacco	Micheli V, Comandatore F, Romeri F, Mancon A, Rimoldi SG
EPI_ISL_486660	Microbiology, Virology and Biemergency Laboratory-ASST FBF Sacco	Microbiology, Virology and Biemergency Laboratory-ASST FBF Sacco	Rimoldi SG, Comandatore F, Romeri F, Mancon A, Micheli V
EPI_ISL_486661	Microbiology, Virology and Biemergency Laboratory-ASST FBF Sacco	Microbiology, Virology and Biemergency Laboratory-ASST FBF Sacco	Romeri F, Comandatore F, Mancon A, Micheli V, Rimoldi SG
EPI_ISL_486662	Microbiology, Virology and Biemergency Laboratory-ASST FBF Sacco	Microbiology, Virology and Biemergency Laboratory-ASST FBF Sacco	Mancon A, Comandatore F, Romeri F, Micheli V, Rimoldi SG
EPI_ISL_486663	Microbiology, Virology and Biemergency Laboratory-ASST FBF Sacco	Microbiology, Virology and Biemergency Laboratory-ASST FBF Sacco	Micheli V, Comandatore F, Romeri F, Mancon A, Rimoldi SG
EPI_ISL_486664	Microbiology, Virology and Biemergency Laboratory-ASST FBF Sacco	Microbiology, Virology and Biemergency Laboratory-ASST FBF Sacco	Rimoldi SG, Comandatore F, Romeri F, Mancon A, Micheli V
EPI_ISL_486665	Microbiology, Virology and Biemergency Laboratory-ASST FBF Sacco	Microbiology, Virology and Biemergency Laboratory-ASST FBF Sacco	Micheli V, Rimoldi SG, Comandatore F, Mancon A, Romeri F
EPI_ISL_487276	Department of Food Safety, Nutrition and Veterinary public health, Istituto Superiore di Sanita'	Department of Biomedical, Surgical and Dental Sciences and Department of Biomedical Sciences for Health	Delbue,S., Ferrante,P., Basilico,N., Parapini,S., Binda,S., D'Alessandro,S., Galli,C., Signorini,L., Primache,V., Anselmi,G., Pariani,E.
EPI_ISL_492980, EPI_ISL_492981, EPI_ISL_492982, EPI_ISL_492983, EPI_ISL_492984, EPI_ISL_492985, EPI_ISL_492986, EPI_ISL_492987	IRCCS Sacro Cuore Don Calabria Hospital, Department of Infectious, Tropical Diseases & Microbiology	University of Verona, Department of Biotechnology	Antonio Mori, Michela Deiana, Elena Pomari, Chiara Piubelli; Giulia Lopatriello, Luca Marcolungo, Cristina Beltrami, Chiara Degli Esposti, Emanuela Cosentino, Massimo Delledonne
EPI_ISL_493328	INMI Lazzaro Spallanzani IRCCS	INMI Lazzaro Spallanzani IRCCS	Martina Rueca, Cesare E.M. Gruber, Barbara Bartolini, Francesco Messina, Maria R. Capobianchi, Antonino Di Caro
EPI_ISL_493329	INMI Lazzaro Spallanzani IRCCS	INMI Lazzaro Spallanzani IRCCS	Barbara Bartolini, Martina Rueca, Cesare E.M. Gruber, Francesco Messina, Antonino Di Caro, Maria R. Capobianchi
EPI_ISL_493330	INMI Lazzaro Spallanzani IRCCS	INMI Lazzaro Spallanzani IRCCS	Cesare E.M. Gruber, Martina Rueca, Barbara Bartolini, Francesco Messina, Maria R. Capobianchi, Antonino Di Caro
EPI_ISL_493331	INMI Lazzaro Spallanzani IRCCS	INMI Lazzaro Spallanzani IRCCS	Martina Rueca, Cesare E.M. Gruber, Barbara Bartolini, Francesco Messina, Maria R. Capobianchi, Antonino Di Caro
EPI_ISL_493332	Istituto Zooprofilattico Sperimentale del Mezzogiorno	INMI Lazzaro Spallanzani IRCCS	Cesare E.M. Gruber, Martina Rueca, Barbara Bartolini, Francesco Messina, Antonino Di Caro, Giovanna Fusco, Maurizio Viscardi, Giorgia Borriello, Maria R. Capobianchi
EPI_ISL_493333	Istituto Zooprofilattico Sperimentale del Mezzogiorno	INMI Lazzaro Spallanzani IRCCS	Barbara Bartolini, Martina Rueca, Cesare E.M. Gruber, Francesco Messina, Antonino Di Caro, Giovanna Fusco, Maurizio Viscardi, Giorgia Borriello, Maria R. Capobianchi
EPI_ISL_496482	Dept. Infectious, Tropical Diseases & Microbiology, IRCCS Sacro Cuore Don Calabria Hospital	1) Dept. Infectious, Tropical Diseases & Microbiology, IRCCS Sacro Cuore Don Calabria Hospital; 2) Centro Piattaforme Tecnologiche, University of Verona; 3) Dept. Neurosciences, Biomedicine and Movement Sciences, University of Verona.	1) Antonio Mori, Michela Deiana, Elena Pomari, Chiara Piubelli; 2) Monica Castellucci and Francesca Griggio; 3) Giovanni Malerba
EPI_ISL_498559, EPI_ISL_498560, EPI_ISL_498561, EPI_ISL_498562, EPI_ISL_498563	Laboratory of Molecular Virology International Center for Genetic Engineering and Biotechnology (ICGEB)	ARGO Open Lab Platform for Genome Sequencing	Licastro D, Rajasekharan S, Dal Monego S, Segat L, D'Agaro P, Marcello A
EPI_ISL_514432	Prof. Massimo Zollo CEINGE TASK-FORCE COVID19 - Regione Campania	Prof. Massimo Zollo CEINGE TASK-FORCE COVID19 - Regione Campania	Veronica Ferrucci, Dae young Kong, Fatemeh asadzadeh, Laura Marrone, Roberto Siciliano, Rino Cerino, Giovanna Fusco, Marika Comegna, Angelo Boccia, Maurizio Viscardi, Giorgia Borriello, Sergio Brandi, Claudia Tiberio, Luigi Atripaldi, Giovanni Paolella, Giuseppe Castaldo, Stefano Pascarella, Martina Bianchi, Lorenzo Chiariotti, Jae Myun Lee, Jae Ho Jung, Kyong Seop Yun, Hong Yeoul Kim and Massimo Zollo
EPI_ISL_514751	CoronaNet Lab- TaskForce Regione Campania, CEINGE Biotecnologie Avanzate, Via G. Salvatore	CoronaNet Lab- TaskForce Regione Campania, CEINGE Biotecnologie Avanzate, Via G. Salvatore	Zollo,M., Ferrucci,V., Kong,Dy., Asadzadeh,F., Marrone,L.,Siciliano,R., Cerino,R., Fusco,G., Comegna,M., Boccia,A.,Viscardi,M., Borriello,G., Brandi,S., Tiberio,C., Atripaldi,L.,Paolella,G., Castaldo,G., Pascarella,S., Bianchi,M., Chiariotti,L.,Lee,J.M., Jung,J.H., Yun,K.S. and Kim,H.Y.
EPI_ISL_516079, EPI_ISL_516080, EPI_ISL_516081, EPI_ISL_516082, EPI_ISL_516083, EPI_ISL_516084, EPI_ISL_516085, EPI_ISL_516086, EPI_ISL_516087, EPI_ISL_516088	Biomedical Sciences and Public Health, Polytechnic University of Marche	Biomedical Sciences and Public Health, Polytechnic University of Marche	Bagnarelli,P., Caucci,S., Di Sante,L., Menzo,S., Alessandrini,F., Onofri,V., Turchi,C., Melchionda,F., Tagliabracci,A.
EPI_ISL_522855	ULSS9 Distretto di Bussolengo	Istituto Zooprofilattico Sperimentale delle Venezie	Adelaide Milani, Alessia Schivo, Annalisa Salviato, Erika Giorgia Quaranta, Ambra Pastori, Bianca Zecchin, Alice Fusaro, Isabella Monne, Calogero Terregino, Antonia Ricci
EPI_ISL_522856	ULSS9 Distretto di San Bonifacio	Istituto Zooprofilattico Sperimentale delle Venezie	Adelaide Milani, Alessia Schivo, Annalisa Salviato, Erika Giorgia Quaranta, Ambra Pastori, Bianca Zecchin, Alice Fusaro, Isabella Monne, Calogero Terregino, Antonia Ricci
EPI_ISL_522857	ULSS9 Scaligera	Istituto Zooprofilattico Sperimentale delle Venezie	Adelaide Milani, Alessia Schivo, Annalisa Salviato, Erika Giorgia Quaranta, Ambra Pastori, Bianca Zecchin, Alice Fusaro, Isabella Monne, Calogero Terregino, Antonia Ricci
EPI_ISL_522858	ULSS9 Distretto di San Bonifacio	Istituto Zooprofilattico Sperimentale delle Venezie	Adelaide Milani, Alessia Schivo, Annalisa Salviato, Erika Giorgia Quaranta, Ambra Pastori, Bianca Zecchin, Alice Fusaro, Isabella Monne, Calogero Terregino, Antonia Ricci
EPI_ISL_522859	ULSS9 Scaligera	Istituto Zooprofilattico Sperimentale delle Venezie	Adelaide Milani, Alessia Schivo, Annalisa Salviato, Erika Giorgia Quaranta, Ambra Pastori, Bianca Zecchin, Alice Fusaro, Isabella Monne, Calogero Terregino, Antonia Ricci
EPI_ISL_522860, EPI_ISL_522861, EPI_ISL_522862, EPI_ISL_522863, EPI_ISL_522864, EPI_ISL_522865, EPI_ISL_522866, EPI_ISL_522867, EPI_ISL_522868	ULSS9 Distretto di Bussolengo	Istituto Zooprofilattico Sperimentale delle Venezie	Adelaide Milani, Alessia Schivo, Annalisa Salviato, Erika Giorgia Quaranta, Ambra Pastori, Bianca Zecchin, Alice Fusaro, Isabella Monne, Calogero Terregino, Antonia Ricci
EPI_ISL_525495, EPI_ISL_525496	Laboratory of Molecular Virology of the International Centre for Genetic Engineering and Biotechnology (ICGEB)	ARGO Open Lab Platform for Genome Sequencing	Licastro D, Rajasekharan S, Dal Monego S, Segat L, D'Agaro P, Marcello A
EPI_ISL_525553, EPI_ISL_525554, EPI_ISL_525555, EPI_ISL_525556, EPI_ISL_525557, EPI_ISL_525558, EPI_ISL_525559, EPI_ISL_525560, EPI_ISL_525561, EPI_ISL_525562, EPI_ISL_525563, EPI_ISL_525564, EPI_ISL_525565, EPI_ISL_525566, EPI_ISL_525567, EPI_ISL_525568, EPI_ISL_525569, EPI_ISL_525570, EPI_ISL_525571, EPI_ISL_525572, EPI_ISL_525573, EPI_ISL_525574, EPI_ISL_527380			
see above	Istituto Zooprofilattico Sperimentale Puglia e Basilicata; Dipartimento di Bioscienze, Biotecnologie e Biofarmaceutica	Beaconlab (Bioinformatics, Evolution and Comparative Genomics lab), Dept of Biosciences, University on Milan	Parisi A.,Pesole G., Manzari C., Chiara M

	dell'Università degli Studi di Bari "A.Moro"; Istituto di Biomembrane. Bioenergetica e Biotecnologie Molecolari del Consiglio Nazionale delle Ricerche di Bari		
EPI_ISL_528919	Ospedale Civile S. Liberatore-Atri	Istitituto Zooprofilattico Sperimentale dell'Abruzzo e Molise "G.Caporale"	Lorusso A, Marcacci M, Di Domenico M, Curini V, Ancora M, Cammà C, Rinaldi A, Mangone I, Di Pasquale A, Puglia I, Savini G.
EPI_ISL_528920, EPI_ISL_528921	Presidio Ospedaliero "Santo Spirito"-Pescara	Istitituto Zooprofilattico Sperimentale dell'Abruzzo e Molise "G.Caporale"	Lorusso A, Marcacci M, Di Domenico M, Curini V, Ancora M, Cammà C, Rinaldi A, Mangone I, Di Pasquale A, Puglia I, Savini G.
EPI_ISL_528922	Ospedale "Giuseppe Mazzini"-Teramo	Istitituto Zooprofilattico Sperimentale dell'Abruzzo e Molise "G.Caporale"	Lorusso A, Marcacci M, Di Domenico M, Curini V, Ancora M, Cammà C, Rinaldi A, Mangone I, Di Pasquale A, Puglia I, Savini G.
EPI_ISL_528923	Ospedale Civile S. Liberatore-Atri	Istitituto Zooprofilattico Sperimentale dell'Abruzzo e Molise "G.Caporale"	Lorusso A, Marcacci M, Di Domenico M, Curini V, Ancora M, Cammà C, Rinaldi A, Mangone I, Di Pasquale A, Puglia I, Savini G.
EPI_ISL_528924	Ospedale "Giuseppe Mazzini"-Teramo	Istitituto Zooprofilattico Sperimentale dell'Abruzzo e Molise "G.Caporale"	Lorusso A, Marcacci M, Di Domenico M, Curini V, Ancora M, Cammà C, Rinaldi A, Mangone I, Di Pasquale A, Puglia I, Savini G.
EPI_ISL_528925	Ospedale Regionale San Salvatore-L'Aquila	Istitituto Zooprofilattico Sperimentale dell'Abruzzo e Molise "G.Caporale"	Lorusso A, Marcacci M, Di Domenico M, Curini V, Ancora M, Cammà C, Rinaldi A, Mangone I, Di Pasquale A, Puglia I, Savini G.
EPI_ISL_528926, EPI_ISL_528927, EPI_ISL_528928	Ospedale "Giuseppe Mazzini"-Teramo	Istitituto Zooprofilattico Sperimentale dell'Abruzzo e Molise "G.Caporale"	Lorusso A, Marcacci M, Di Domenico M, Curini V, Ancora M, Cammà C, Rinaldi A, Mangone I, Di Pasquale A, Puglia I, Savini G.
EPI_ISL_528929	Ospedale Civile S. Liberatore-Atri	Istitituto Zooprofilattico Sperimentale dell'Abruzzo e Molise "G.Caporale"	Lorusso A, Marcacci M, Di Domenico M, Curini V, Ancora M, Cammà C, Rinaldi A, Mangone I, Di Pasquale A, Puglia I, Savini G.
EPI_ISL_528934, EPI_ISL_528935, EPI_ISL_528936, EPI_ISL_528937, EPI_ISL_528938, EPI_ISL_528939, EPI_ISL_528940, EPI_ISL_528941, EPI_ISL_528942, EPI_ISL_528943, EPI_ISL_528944, EPI_ISL_528945, EPI_ISL_528946, EPI_ISL_528947, EPI_ISL_528948, EPI_ISL_528949			
see above	Agenzia di Tutela della Salute di Bergamo	Istitituto Zooprofilattico Sperimentale dell'Abruzzo e Molise "G.Caporale"	Lorusso A, Marcacci M, Di Domenico M, Curini V, Ancora M, Cammà C, Rinaldi A, Mangone I, Di Pasquale A, Puglia I, Savini G.
EPI_ISL_528990	Ospedale Civile Maria SS. dello Splendore	Istitituto Zooprofilattico Sperimentale dell'Abruzzo e Molise "G.Caporale"	Lorusso A, Marcacci M, Di Domenico M, Curini V, Ancora M, Cammà C, Rinaldi A, Mangone I, Di Pasquale A, Puglia I, Savini G.
EPI_ISL_528991, EPI_ISL_528992	Ospedale SS Annunziata-Sulmona	Istitituto Zooprofilattico Sperimentale dell'Abruzzo e Molise "G.Caporale"	Lorusso A, Marcacci M, Di Domenico M, Curini V, Ancora M, Cammà C, Rinaldi A, Mangone I, Di Pasquale A, Puglia I, Savini G.
EPI_ISL_528993	Ospedale Civile S. Liberatore-Atri	Istitituto Zooprofilattico Sperimentale dell'Abruzzo e Molise "G.Caporale"	Lorusso A, Marcacci M, Di Domenico M, Curini V, Ancora M, Cammà C, Rinaldi A, Mangone I, Di Pasquale A, Puglia I, Savini G.
EPI_ISL_528994, EPI_ISL_528995, EPI_ISL_528996, EPI_ISL_528997, EPI_ISL_528998, EPI_ISL_528999, EPI_ISL_529000, EPI_ISL_529001, EPI_ISL_529002, EPI_ISL_529003, EPI_ISL_529004, EPI_ISL_529005			
see above	Servizio di igiene epidemiologia e sanità pubblica (SIESP)-Chieti	Istitituto Zooprofilattico Sperimentale dell'Abruzzo e Molise "G.Caporale"	Lorusso A, Marcacci M, Di Domenico M, Curini V, Ancora M, Cammà C, Rinaldi A, Mangone I, Di Pasquale A, Puglia I, Savini G.
EPI_ISL_529006	Servizio di igiene e sanità pubblica (SIESP)-Teramo	Istitituto Zooprofilattico Sperimentale dell'Abruzzo e Molise "G.Caporale"	Lorusso A, Marcacci M, Di Domenico M, Curini V, Ancora M, Cammà C, Rinaldi A, Mangone I, Di Pasquale A, Puglia I, Savini G.
EPI_ISL_529007	Ospedale Civile S. Liberatore-Atri	Istitituto Zooprofilattico Sperimentale dell'Abruzzo e Molise "G.Caporale"	Lorusso A, Marcacci M, Di Domenico M, Curini V, Ancora M, Cammà C, Rinaldi A, Mangone I, Di Pasquale A, Puglia I, Savini G.
EPI_ISL_529008	Servizio di igiene e sanità pubblica (SIESP)-Teramo	Istitituto Zooprofilattico Sperimentale dell'Abruzzo e Molise "G.Caporale"	Lorusso A, Marcacci M, Di Domenico M, Curini V, Ancora M, Cammà C, Rinaldi A, Mangone I, Di Pasquale A, Puglia I, Savini G.
EPI_ISL_529009	Ospedale Civile S. Liberatore-Atri	Istitituto Zooprofilattico Sperimentale dell'Abruzzo e Molise "G.Caporale"	Lorusso A, Marcacci M, Di Domenico M, Curini V, Ancora M, Cammà C, Rinaldi A, Mangone I, Di Pasquale A, Puglia I, Savini G.
EPI_ISL_529010, EPI_ISL_529011, EPI_ISL_529012	Servizio Igiene Epidemiologia e Sanità Pubblica (SIESP)-L'Aquila	Istitituto Zooprofilattico Sperimentale dell'Abruzzo e Molise "G.Caporale"	Lorusso A, Marcacci M, Di Domenico M, Curini V, Ancora M, Cammà C, Rinaldi A, Mangone I, Di Pasquale A, Puglia I, Savini G.
EPI_ISL_529013	Presidio Ospedaliero "S.Filippo e Nicola"-Avezzano	Istitituto Zooprofilattico Sperimentale dell'Abruzzo e Molise "G.Caporale"	Lorusso A, Marcacci M, Di Domenico M, Curini V, Ancora M, Cammà C, Rinaldi A, Mangone I, Di Pasquale A, Puglia I, Savini G.
EPI_ISL_529014, EPI_ISL_529015	Ospedale "Ss. Annunziata"	Istitituto Zooprofilattico Sperimentale dell'Abruzzo e Molise "G.Caporale"	Lorusso A, Marcacci M, Di Domenico M, Curini V, Ancora M, Cammà C, Rinaldi A, Mangone I, Di Pasquale A, Puglia I, Savini G.
EPI_ISL_529016	Ospedale SS Annunziata-Sulmona	Istitituto Zooprofilattico Sperimentale dell'Abruzzo e Molise "G.Caporale"	Lorusso A, Marcacci M, Di Domenico M, Curini V, Ancora M, Cammà C, Rinaldi A, Mangone I, Di Pasquale A, Puglia I, Savini G.
EPI_ISL_529017	RSA San Raffaele Sulmona	Istitituto Zooprofilattico Sperimentale dell'Abruzzo e Molise "G.Caporale"	Lorusso A, Marcacci M, Di Domenico M, Curini V, Ancora M, Cammà C, Rinaldi A, Mangone I, Di Pasquale A, Puglia I, Savini G.
EPI_ISL_529018	Ospedale "Giuseppe Mazzini"-Teramo	Istitituto Zooprofilattico Sperimentale dell'Abruzzo e Molise "G.Caporale"	Lorusso A, Marcacci M, Di Domenico M, Curini V, Ancora M, Cammà C, Rinaldi A, Mangone I, Di Pasquale A, Puglia I, Savini G.
EPI_ISL_529019	RSA/RP Villa San Giovanni - Gruppo Edos	Istitituto Zooprofilattico Sperimentale dell'Abruzzo e Molise "G.Caporale"	Lorusso A, Marcacci M, Di Domenico M, Curini V, Ancora M, Cammà C, Rinaldi A, Mangone I, Di Pasquale A, Puglia I, Savini G.
EPI_ISL_529020, EPI_ISL_529021	Ospedale Civile S. Liberatore-Atri	Istitituto Zooprofilattico Sperimentale dell'Abruzzo e Molise "G.Caporale"	Lorusso A, Marcacci M, Di Domenico M, Curini V, Ancora M, Cammà C, Rinaldi A, Mangone I, Di Pasquale A, Puglia I, Savini G.
EPI_ISL_529022	Ospedale "Ss. Annunziata"	Istitituto Zooprofilattico Sperimentale dell'Abruzzo e Molise "G.Caporale"	Lorusso A, Marcacci M, Di Domenico M, Curini V, Ancora M, Cammà C, Rinaldi A, Mangone I, Di Pasquale A, Puglia I, Savini G.
EPI_ISL_529023, EPI_ISL_529024, EPI_ISL_529025	Servizio di igiene epidemiologia e sanità pubblica (SIESP)-Chieti	Istitituto Zooprofilattico Sperimentale dell'Abruzzo e Molise "G.Caporale"	Lorusso A, Marcacci M, Di Domenico M, Curini V, Ancora M, Cammà C, Rinaldi A, Mangone I, Di Pasquale A, Puglia I, Savini G.
EPI_ISL_529026	Ospedale "Giuseppe Mazzini"-Teramo	Istitituto Zooprofilattico Sperimentale dell'Abruzzo e Molise "G.Caporale"	Lorusso A, Marcacci M, Di Domenico M, Curini V, Ancora M, Cammà C, Rinaldi A, Mangone I, Di Pasquale A, Puglia I, Savini G.
EPI_ISL_542098, EPI_ISL_542099, EPI_ISL_542100, EPI_ISL_542101, EPI_ISL_542102, EPI_ISL_542103, EPI_ISL_542104, EPI_ISL_542105, EPI_ISL_542106, EPI_ISL_542107, EPI_ISL_542108, EPI_ISL_542109, EPI_ISL_542110, EPI_ISL_542111, EPI_ISL_542112, EPI_ISL_542113, EPI_ISL_542114, EPI_ISL_542115, EPI_ISL_542116, EPI_ISL_542117, EPI_ISL_542118, EPI_ISL_542119, EPI_ISL_542120, EPI_ISL_542121, EPI_ISL_542122, EPI_ISL_542123, EPI_ISL_542124, EPI_ISL_542125, EPI_ISL_542126, EPI_ISL_542127, EPI_ISL_542128, EPI_ISL_542129, EPI_ISL_542130, EPI_ISL_542131, EPI_ISL_542132, EPI_ISL_542133, EPI_ISL_542134, EPI_ISL_542135, EPI_ISL_542136, EPI_ISL_542137, EPI_ISL_542138, EPI_ISL_542139, EPI_ISL_542140, EPI_ISL_542141, EPI_ISL_542142, EPI_ISL_542143, EPI_ISL_542144, EPI_ISL_542145, EPI_ISL_542146, EPI_ISL_542147, EPI_ISL_542148, EPI_ISL_542149, EPI_ISL_542150, EPI_ISL_542151, EPI_ISL_542152, EPI_ISL_542153, EPI_ISL_542154, EPI_ISL_542155, EPI_ISL_542156, EPI_ISL_542157, EPI_ISL_542158, EPI_ISL_542159, EPI_ISL_542160, EPI_ISL_542161, EPI_ISL_542162, EPI_ISL_542163, EPI_ISL_542164, EPI_ISL_542165, EPI_ISL_542166, EPI_ISL_542167, EPI_ISL_542168, EPI_ISL_542169, EPI_ISL_542170, EPI_ISL_542171, EPI_ISL_542172, EPI_ISL_542173, EPI_ISL_542174, EPI_ISL_542175, EPI_ISL_542176, EPI_ISL_542177, EPI_ISL_542178, EPI_ISL_542179, EPI_ISL_542180, EPI_ISL_542181, EPI_ISL_542182, EPI_ISL_542183, EPI_ISL_542184, EPI_ISL_542185, EPI_ISL_542186, EPI_ISL_542187, EPI_ISL_542188, EPI_ISL_542189, EPI_ISL_542190, EPI_ISL_542191, EPI_ISL_542192, EPI_ISL_542193, EPI_ISL_542194, EPI_ISL_542195, EPI_ISL_542196, EPI_ISL_542197, EPI_ISL_542198, EPI_ISL_542199, EPI_ISL_542200, EPI_ISL_542201, EPI_ISL_542202, EPI_ISL_542203, EPI_ISL_542204, EPI_ISL_542205, EPI_ISL_542206, EPI_ISL_542207, EPI_ISL_542208, EPI_ISL_542209, EPI_ISL_542210, EPI_ISL_542211, EPI_ISL_542212, EPI_ISL_542213, EPI_ISL_542214, EPI_ISL_542215, EPI_ISL_542216, EPI_ISL_542217, EPI_ISL_542218, EPI_ISL_542219, EPI_ISL_542220, EPI_ISL_542221, EPI_ISL_542222, EPI_ISL_542223, EPI_ISL_542224, EPI_ISL_542225, EPI_ISL_542226, EPI_ISL_542227, EPI_ISL_542228, EPI_ISL_542229, EPI_ISL_542230, EPI_ISL_542231, EPI_ISL_542232, EPI_ISL_542233, EPI_ISL_542234, EPI_ISL_542235, EPI_ISL_542236, EPI_ISL_542237, EPI_ISL_542238, EPI_ISL_542239, EPI_ISL_542240, EPI_ISL_542241, EPI_ISL_542242, EPI_ISL_542243, EPI_ISL_542244, EPI_ISL_542245, EPI_ISL_542246, EPI_ISL_542247, EPI_ISL_542248, EPI_ISL_542249, EPI_ISL_542250, EPI_ISL_542251, EPI_ISL_542252, EPI_ISL_542253, EPI_ISL_542254, EPI_ISL_542255, EPI_ISL_542256, EPI_ISL_542257, EPI_ISL_542258, EPI_ISL_542259, EPI_ISL_542260, EPI_ISL_542261, EPI_ISL_542262, EPI_ISL_542263, EPI_ISL_542264, EPI_ISL_542265, EPI_ISL_542266, EPI_ISL_542267, EPI_ISL_542268, EPI_ISL_542269, EPI_ISL_542270, EPI_ISL_542271, EPI_ISL_542272, EPI_ISL_542273, EPI_ISL_542274, EPI_ISL_542275, EPI_ISL_542276, EPI_ISL_542277			
see above	ASST GOM Niguarda	Dep. Of Oncology and Hemato-Oncology University of Milan	Claudia Alteri, Valeria Cento, Antonio Piralla, Valentino Costabile, Monica Tallarita, Luna Colagrossi, Silvia Renica, Federica Giardina, Federica Novazzi,

			Stefano Gaiarsa, Elisa Matarazzo, Maria Antonello, Chiara Vismara, Roberto Fumagalli, Oscar Massimiliano Epis, Massimo Puoti, Carlo Federico Perno, Fausto Baldanti
EPI_ISL_542278, EPI_ISL_542279, EPI_ISL_542280, EPI_ISL_542281, EPI_ISL_542282, EPI_ISL_542283, EPI_ISL_542284, EPI_ISL_542285, EPI_ISL_542286, EPI_ISL_542287, EPI_ISL_542288, EPI_ISL_542289, EPI_ISL_542290, EPI_ISL_542291, EPI_ISL_542292, EPI_ISL_542293, EPI_ISL_542294, EPI_ISL_542295, EPI_ISL_542296, EPI_ISL_542297, EPI_ISL_542298, EPI_ISL_542299, EPI_ISL_542300, EPI_ISL_542301, EPI_ISL_542302, EPI_ISL_542303, EPI_ISL_542304, EPI_ISL_542305, EPI_ISL_542306, EPI_ISL_542307, EPI_ISL_542308, EPI_ISL_542309, EPI_ISL_542310, EPI_ISL_542311, EPI_ISL_542312, EPI_ISL_542313, EPI_ISL_542314, EPI_ISL_542315, EPI_ISL_542316, EPI_ISL_542317, EPI_ISL_542318, EPI_ISL_542319, EPI_ISL_542320, EPI_ISL_542321, EPI_ISL_542322, EPI_ISL_542323, EPI_ISL_542324, EPI_ISL_542325, EPI_ISL_542326, EPI_ISL_542327, EPI_ISL_542328, EPI_ISL_542329, EPI_ISL_542330, EPI_ISL_542331, EPI_ISL_542332, EPI_ISL_542333, EPI_ISL_542334, EPI_ISL_542335, EPI_ISL_542336, EPI_ISL_542337, EPI_ISL_542338, EPI_ISL_542339, EPI_ISL_542340, EPI_ISL_542341, EPI_ISL_542342, EPI_ISL_542343, EPI_ISL_542344, EPI_ISL_542345, EPI_ISL_542346, EPI_ISL_542347, EPI_ISL_542348, EPI_ISL_542349, EPI_ISL_542350, EPI_ISL_542351, EPI_ISL_542352, EPI_ISL_542353, EPI_ISL_542354, EPI_ISL_542355, EPI_ISL_542356, EPI_ISL_542357, EPI_ISL_542358, EPI_ISL_542359, EPI_ISL_542360, EPI_ISL_542361, EPI_ISL_542362, EPI_ISL_542363, EPI_ISL_542364, EPI_ISL_542365, EPI_ISL_542366, EPI_ISL_542367, EPI_ISL_542368, EPI_ISL_542369, EPI_ISL_542370, EPI_ISL_542371, EPI_ISL_542372, EPI_ISL_542373, EPI_ISL_542374, EPI_ISL_542375, EPI_ISL_542376, EPI_ISL_542377, EPI_ISL_542378, EPI_ISL_542379, EPI_ISL_542380, EPI_ISL_542381, EPI_ISL_542382, EPI_ISL_542383, EPI_ISL_542384, EPI_ISL_542385, EPI_ISL_542386, EPI_ISL_542387, EPI_ISL_542388, EPI_ISL_542389, EPI_ISL_542390, EPI_ISL_542391, EPI_ISL_542392, EPI_ISL_542393, EPI_ISL_542394, EPI_ISL_542395, EPI_ISL_542396, EPI_ISL_542397, EPI_ISL_542398, EPI_ISL_542399			
see above	San Matteo Hospital Pavia	Dep. Of Oncology and Hemato-Oncology University of Milan	Claudia Alteri, Valeria Cento, Antonio Piralla, Valentino Costabile, Monica Tallarita, Luna Colagrossi, Silvia Renica, Federica Giardina, Federica Novazzi, Stefano Gaiarsa, Elisa Matarazzo, Maria Antonello, Chiara Vismara, Roberto Fumagalli, Oscar Massimiliano Epis, Massimo Puoti, Carlo Federico Perno, Fausto Baldanti
EPI_ISL_542400, EPI_ISL_542401, EPI_ISL_542402, EPI_ISL_542403, EPI_ISL_542404, EPI_ISL_542405, EPI_ISL_542406, EPI_ISL_542407, EPI_ISL_542408, EPI_ISL_542409, EPI_ISL_542410, EPI_ISL_542411, EPI_ISL_542412, EPI_ISL_542413, EPI_ISL_542414, EPI_ISL_542415, EPI_ISL_542416, EPI_ISL_542417, EPI_ISL_542418, EPI_ISL_542419, EPI_ISL_542420, EPI_ISL_542421, EPI_ISL_542422, EPI_ISL_542423, EPI_ISL_542424, EPI_ISL_542425, EPI_ISL_542426, EPI_ISL_542427, EPI_ISL_542428, EPI_ISL_542429, EPI_ISL_542430, EPI_ISL_542431, EPI_ISL_542432, EPI_ISL_542433, EPI_ISL_542434, EPI_ISL_542435, EPI_ISL_542436, EPI_ISL_542437, EPI_ISL_542438, EPI_ISL_542439, EPI_ISL_542440, EPI_ISL_542441, EPI_ISL_542442, EPI_ISL_542443			
see above	ASST GOM Niguarda	Dep. Of Oncology and Hemato-Oncology University of Milan	Claudia Alteri, Valeria Cento, Antonio Piralla, Valentino Costabile, Monica Tallarita, Luna Colagrossi, Silvia Renica, Federica Giardina, Federica Novazzi, Stefano Gaiarsa, Elisa Matarazzo, Maria Antonello, Chiara Vismara, Roberto Fumagalli, Oscar Massimiliano Epis, Massimo Puoti, Carlo Federico Perno, Fausto Baldanti
EPI_ISL_547965	Laboratorio Biologia Molecolare SarsCov2 UOC LAboratorio Analisi SErvizio Medicina di Laboratorio Ospedale San Francesco ATS-ASSL Nuoro	Laboratorio Specialistico UOC Ematologia Ospedale San Francesco - ATS ASSL NUORO	Piras Giovanna, Aspironi Rosanna, Monne Maria Itria, Fancello Tatiana,Fiamma Maura,Toja Alessandro, Sanna Filomena, Floris Anna Rita, Sulis Vincenzo, Palmas Angelo Domenico, Casu Gavino, Lo Maglio Iana, Mameli Giuseppe.
EPI_ISL_560407	Istituto Zooprofilattico Sperimentale del Mezzogiorno	INMI Lazzaro Spallanzani IRCCS	Barbara Bartolini, Cesare E.M. Gruber, Martina Rueca, Francesco Messina, Antonino Di Caro, Giovanna Fusco, Maurizio Viscardi, Giorgia Borriello, Sergio Brandi, Maria R. Capobianchi
EPI_ISL_568579	Virus Molecular Laboratory of the Microbiology and Virology Department	INMI Lazzaro Spallanzani IRCCS	Cesare E.M. Gruber, Martina Rueca, Barbara Bartolini, Francesco Messina, Silvia Meschi, Francesca Colavita, Concetta Castilletti, Elena Percivalle, Irene Cassaniti, Edoardo Vecchio Nepita, Fausto Baldanti, Maria R. Capobianchi, Antonino Di Caro
EPI_ISL_569865, EPI_ISL_569866, EPI_ISL_569867, EPI_ISL_569868, EPI_ISL_569869, EPI_ISL_569870, EPI_ISL_569871, EPI_ISL_569872, EPI_ISL_569873, EPI_ISL_569874, EPI_ISL_569875, EPI_ISL_569876, EPI_ISL_569877, EPI_ISL_569878, EPI_ISL_569879, EPI_ISL_569880, EPI_ISL_569881, EPI_ISL_569882, EPI_ISL_569883, EPI_ISL_569884, EPI_ISL_569885, EPI_ISL_569886			
see above	Amedeo di savoia	Crosetto lab, Karolinska Institutet, SciLifeLab	Michele Simonetti, Maria Grazia Milia, Luuk Harbers, Ning Zhang, Anna Sapino, Valeria Ghisetti, Nicola Crosetto
EPI_ISL_572320, EPI_ISL_572321, EPI_ISL_572322, EPI_ISL_572323, EPI_ISL_572324	IZSM	IZSM	Maurizio Viscardi, Lorena Cardillo, Giovanna Fusco
EPI_ISL_582123, EPI_ISL_583954, EPI_ISL_583955, EPI_ISL_583956, EPI_ISL_583957, EPI_ISL_583958, EPI_ISL_583959, EPI_ISL_583960, EPI_ISL_583961, EPI_ISL_583962, EPI_ISL_583963, EPI_ISL_583964, EPI_ISL_583965, EPI_ISL_583966, EPI_ISL_583967			
see above	UOC Microbiologia e Virologia, Azienda Ospedaliera Universitaria Senese, Siena, Italy	Dipartimento di Biotecnologie Mediche	Maria Grazia Cusi, David Pinzauti, Claudia Gandolfo, Gabriele Anichini, Gianni Pozzi, Francesco Santoro
EPI_ISL_584048	Laboratory of Molecular Virology, Department of Biomedical, Surgical and Dental Sciences University of Milano	Laboratory of Molecular Virology, Department of Biomedical, Surgical and Dental Sciences University of Milano	Delbue,S., Modenese,A., Bianchi,M., Fattori,M., D'Alessandro,S., Pariani,E., Basilico,N., Galli,C. and Ferrante,P.
EPI_ISL_584049	Laboratory of Molecular Virology, Department of Biomedical, Surgical and Dental Sciences University of Milano	Laboratory of Molecular Virology, Department of Biomedical, Surgical and Dental Sciences University of Milano	Delbue,S., Modenese,A., Bianchi,M., Fattori,M., D'Alessandro,S.,Pariani,E., Basilico,N., Galli,C. and Ferrante,P.
EPI_ISL_584051	Laboratory of Molecular Virology, Department of Biomedical, Surgical and Dental Sciences University of Milano	Laboratory of Molecular Virology, Department of Biomedical, Surgical and Dental Sciences University of Milano	Delbue,S., Ferrante,P., Basilico,N., Parapini,S., Binda,S., D'Alessandro,S., Galli,C., Signorini,L., Primache,V., Anselmi,G. and Pariani,E
EPI_ISL_584052	Laboratory of Molecular Virology, Department of Biomedical, Surgical and Dental Sciences University of Milano	Laboratory of Molecular Virology, Department of Biomedical, Surgical and Dental Sciences University of Milano	Delbue,S., D'Alessandro,S., Modenese,A., Signorini,L., Parapini,S.,Dolci,M., Binda,S., Primache,V., Taramelli,D., Incorvaia,B. and Ferrante,P.
EPI_ISL_584069, EPI_ISL_584071, EPI_ISL_584072	IZSM	IZSM	Maurizio Viscardi, Lorena Cardillo, Giovanna Fusco
EPI_ISL_590693	INMI Lazzaro Spallanzani IRCCS	INMI Lazzaro Spallanzani IRCCS	Martina Rueca, Barbara Bartolini, Cesare E.M. Gruber, Francesco Messina, Emanuela Giombini, Beatrice Valli, Eleonora Lalle, Simone Lanini, Francesco Vairo, Maria R. Capobianchi, Antonino Di Caro
EPI_ISL_590694	INMI Lazzaro Spallanzani IRCCS	INMI Lazzaro Spallanzani IRCCS	Barbara Bartolini, Martina Rueca, Francesco Messina, Cesare E.M. Gruber, Emanuela Giombini, Beatrice Valli, Eleonora Lalle, Simone Lanini, Francesco Vairo, Maria R. Capobianchi, Antonino Di Caro
EPI_ISL_590695	INMI Lazzaro Spallanzani IRCCS	INMI Lazzaro Spallanzani IRCCS	Cesare E.M. Gruber, Francesco Messina, Barbara Bartolini, Martina Rueca, Emanuela Giombini, Beatrice Valli, Eleonora Lalle, Simone Lanini, Francesco Vairo, Antonino Di Caro, Maria R. Capobianchi
EPI_ISL_590696	INMI Lazzaro Spallanzani IRCCS	INMI Lazzaro Spallanzani IRCCS	Cesare E.M. Gruber, Barbara Bartolini, Francesco Messina, Martina Rueca, Emanuela Giombini, Beatrice Valli, Eleonora Lalle, Simone Lanini, Francesco Vairo, Antonino Di Caro, Maria R. Capobianchi
EPI_ISL_590697	INMI Lazzaro Spallanzani IRCCS	INMI Lazzaro Spallanzani IRCCS	Martina Rueca, Cesare E.M. Gruber, Barbara Bartolini, Francesco Messina, Emanuela Giombini, Beatrice Valli, Eleonora Lalle, Simone Lanini, Francesco Vairo, Antonino Di Caro, Maria R. Capobianchi
EPI_ISL_590698	INMI Lazzaro Spallanzani IRCCS	INMI Lazzaro Spallanzani IRCCS	Barbara Bartolini, Francesco Messina, Cesare E.M. Gruber, Martina Rueca, Emanuela Giombini, Beatrice Valli, Eleonora Lalle, Simone Lanini, Francesco Vairo, Maria R. Capobianchi, Antonino Di Caro
EPI_ISL_591327, EPI_ISL_591328, EPI_ISL_591329, EPI_ISL_591330, EPI_ISL_591331, EPI_ISL_591332, EPI_ISL_591333, EPI_ISL_591334, EPI_ISL_591335	Dipartimento di Biotecnologie Mediche, University of Siena	Dipartimento di Biotecnologie Mediche, University of Siena	Cusi,M.G., Pinzauti,D., Gandolfo,C., Anichini,G., Pozzi,G., Santoro,F.
EPI_ISL_591336	Dipartimento di Biotecnologie Mediche, University of Siena	Dipartimento di Biotecnologie Mediche, University of Siena	COVID
EPI_ISL_591337, EPI_ISL_591338, EPI_ISL_591339, EPI_ISL_591340	Dipartimento di Biotecnologie Mediche, University of Siena	Dipartimento di Biotecnologie Mediche, University of Siena	Cusi,M.G., Pinzauti,D., Gandolfo,C., Anichini,G., Pozzi,G., Santoro,F.
EPI_ISL_602304	Istituto Zooprofilattico Sperimentale del Mezzogiorno	U.O. Diagnostica Virologica Dip. Sanità Animale IZSM	Maurizio Viscardi, Lorena Cardillo , e Giovanna Fusco
EPI_ISL_603137	INMI Lazzaro Spallanzani IRCCS	INMI Lazzaro Spallanzani IRCCS	Cesare E.M. Gruber, Martina Rueca, Barbara Bartolini, Francesco Messina, Emanuela Giombini, Simone Lanini, Antonino Di Caro, Maria R. Capobianchi
EPI_ISL_603138	INMI Lazzaro Spallanzani IRCCS	INMI Lazzaro Spallanzani IRCCS	Martina Rueca, Francesco Messina, Barbara Bartolini, Cesare E.M. Gruber, Emanuela Giombini, Simone Lanini, Antonino Di Caro, Maria R. Capobianchi
EPI_ISL_603139	INMI Lazzaro Spallanzani IRCCS	INMI Lazzaro Spallanzani IRCCS	Martina Rueca, Cesare E.M. Gruber, Barbara Bartolini, Francesco Messina, Emanuela Giombini, Simone Lanini, Antonino Di Caro, Maria R. Capobianchi
EPI_ISL_603140	INMI Lazzaro Spallanzani IRCCS	INMI Lazzaro Spallanzani IRCCS	Martina Rueca, Cesare E.M. Gruber, Francesco Messina, Barbara Bartolini, Emanuela Giombini, Simone Lanini, Antonino Di Caro, Maria R. Capobianchi
EPI_ISL_603141	INMI Lazzaro Spallanzani IRCCS	INMI Lazzaro Spallanzani IRCCS	Francesco Messina, Cesare E.M. Gruber, Martina Rueca, Barbara Bartolini, Emanuela Giombini, Simone Lanini, Maria R. Capobianchi, Antonino Di Caro
EPI_ISL_603142	INMI Lazzaro Spallanzani IRCCS	INMI Lazzaro Spallanzani IRCCS	Barbara Bartolini, Cesare E.M. Gruber, Francesco Messina, Martina Rueca, Simone Lanini, Emanuela Giombini, Maria R. Capobianchi, Antonino Di Caro

EPI_ISL_603143	INMI Lazzaro Spallanzani IRCCS	INMI Lazzaro Spallanzani IRCCS	Martina Rueca, Francesco Messina, Cesare E.M. Gruber, Barbara Bartolini, Emanuela Giombini, Simone Lanini, Antonino Di Caro, Maria R. Capobianchi
EPI_ISL_603144	INMI Lazzaro Spallanzani IRCCS	INMI Lazzaro Spallanzani IRCCS	Cesare E.M. Gruber, Martina Rueca, Francesco Messina, Barbara Bartolini, Emanuela Giombini, Simone Lanini, Maria R. Capobianchi, Antonino Di Caro
EPI_ISL_603145	INMI Lazzaro Spallanzani IRCCS	INMI Lazzaro Spallanzani IRCCS	Francesco Messina, Cesare E.M. Gruber, Martina Rueca, Barbara Bartolini, Emanuela Giombini, Simone Lanini, Antonino Di Caro, Maria R. Capobianchi
EPI_ISL_603146	INMI Lazzaro Spallanzani IRCCS	INMI Lazzaro Spallanzani IRCCS	Cesare E.M. Gruber, Martina Rueca, Barbara Bartolini, Emanuela Giombini, Francesco Messina, Simone Lanini, Maria R. Capobianchi, Antonino Di Caro
EPI_ISL_603147	INMI Lazzaro Spallanzani IRCCS	INMI Lazzaro Spallanzani IRCCS	Cesare E.M. Gruber, Barbara Bartolini, Francesco Messina, Martina Rueca, Simone Lanini, Emanuela Giombini, Antonino Di Caro, Maria R. Capobianchi
EPI_ISL_603148	INMI Lazzaro Spallanzani IRCCS	INMI Lazzaro Spallanzani IRCCS	Cesare E.M. Gruber, Francesco Messina, Martina Rueca, Simone Lanini, Emanuela Giombini, Barbara Bartolini, Maria R. Capobianchi, Antonino Di Caro
EPI_ISL_603149	INMI Lazzaro Spallanzani IRCCS	INMI Lazzaro Spallanzani IRCCS	Francesco Messina, Barbara Bartolini, Cesare E.M. Gruber, Martina Rueca, Simone Lanini, Emanuela Giombini, Patrizia Massarelli, Antonino Di Caro, Maria R. Capobianchi
EPI_ISL_603150	INMI Lazzaro Spallanzani IRCCS	INMI Lazzaro Spallanzani IRCCS	Barbara Bartolini, Francesco Messina, Martina Rueca, Cesare E.M. Gruber, Simone Lanini, Emanuela Giombini, Patrizia Massarelli, Maria R. Capobianchi, Antonino Di Caro
EPI_ISL_603151	INMI Lazzaro Spallanzani IRCCS	INMI Lazzaro Spallanzani IRCCS	Francesco Messina, Barbara Bartolini, Cesare E.M. Gruber, Martina Rueca, Emanuela Giombini, Simone Lanini, Patrizia Massarelli, Maria R. Capobianchi, Antonino Di Caro
EPI_ISL_603152	INMI Lazzaro Spallanzani IRCCS	INMI Lazzaro Spallanzani IRCCS	Barbara Bartolini, Cesare E.M. Gruber, Francesco Messina, Martina Rueca, Emanuela Giombini, Simone Lanini, Patrizia Massarelli, Antonino Di Caro, Maria R. Capobianchi
EPI_ISL_603153	INMI Lazzaro Spallanzani IRCCS	INMI Lazzaro Spallanzani IRCCS	Francesco Messina, Cesare E.M. Gruber, Barbara Bartolini, Simone Lanini, Emanuela Giombini, Martina Rueca, Marcello Meledandri, Maria Letizia Schiavone, Annunziata Tamburro, Antonino Di Caro, Maria R. Capobianchi
EPI_ISL_603154	INMI Lazzaro Spallanzani IRCCS	INMI Lazzaro Spallanzani IRCCS	Martina Rueca, Francesco Messina, Cesare E.M. Gruber, Barbara Bartolini, Emanuela Giombini, Simone Lanini, Antonino Di Caro, Maria R. Capobianchi
EPI_ISL_603155	INMI Lazzaro Spallanzani IRCCS	INMI Lazzaro Spallanzani IRCCS	Martina Rueca, Francesco Messina, Barbara Bartolini, Cesare E.M. Gruber, Simone Lanini, Emanuela Giombini, Maria R. Capobianchi, Antonino Di Caro
EPI_ISL_603156	INMI Lazzaro Spallanzani IRCCS	INMI Lazzaro Spallanzani IRCCS	Barbara Bartolini, Francesco Messina, Cesare E.M. Gruber, Martina Rueca, Emanuela Giombini, Simone Lanini, Maria R. Capobianchi, Antonino Di Caro
EPI_ISL_603157	INMI Lazzaro Spallanzani IRCCS	INMI Lazzaro Spallanzani IRCCS	Francesco Messina, Martina Rueca, Barbara Bartolini, Cesare E.M. Gruber, Simone Lanini, Emanuela Giombini, Fulvia Pimpinelli, Antonino Di Caro, Maria R. Capobianchi
EPI_ISL_603158	INMI Lazzaro Spallanzani IRCCS	INMI Lazzaro Spallanzani IRCCS	Barbara Bartolini, Cesare E.M. Gruber, Francesco Messina, Martina Rueca, Emanuela Giombini, Simone Lanini, Fulvia Pimpinelli, Maria R. Capobianchi, Antonino Di Caro
EPI_ISL_603159	INMI Lazzaro Spallanzani IRCCS	INMI Lazzaro Spallanzani IRCCS	Barbara Bartolini, Francesco Messina, Cesare E.M. Gruber, Martina Rueca, Simone Lanini, Emanuela Giombini, Fulvia Pimpinelli, Maria R. Capobianchi, Antonino Di Caro
EPI_ISL_603160	INMI Lazzaro Spallanzani IRCCS	INMI Lazzaro Spallanzani IRCCS	Barbara Bartolini, Simone Lanini, Emanuela Giombini, Francesco Messina, Cesare E.M. Gruber, Martina Rueca, Fulvia Pimpinelli, Maria R. Capobianchi, Antonino Di Caro
EPI_ISL_603161	INMI Lazzaro Spallanzani IRCCS	INMI Lazzaro Spallanzani IRCCS	Cesare E.M. Gruber, Barbara Bartolini, Francesco Messina, Martina Rueca, Emanuela Giombini, Simone Lanini, Fulvia Pimpinelli, Antonino Di Caro, Maria R. Capobianchi
EPI_ISL_603162	INMI Lazzaro Spallanzani IRCCS	INMI Lazzaro Spallanzani IRCCS	Cesare E.M. Gruber, Francesco Messina, Martina Rueca, Barbara Bartolini, Emanuela Giombini, Simone Lanini, Fulvia Pimpinelli, Antonino Di Caro, Maria R. Capobianchi
EPI_ISL_603163	INMI Lazzaro Spallanzani IRCCS	INMI Lazzaro Spallanzani IRCCS	Cesare E.M. Gruber, Martina Rueca, Emanuela Giombini, Simone Lanini, Francesco Messina, Barbara Bartolini, Fulvia Pimpinelli, Maria R. Capobianchi, Antonino Di Caro
EPI_ISL_603164	INMI Lazzaro Spallanzani IRCCS	INMI Lazzaro Spallanzani IRCCS	Cesare E.M. Gruber, Francesco Messina, Martina Rueca, Barbara Bartolini, Emanuela Giombini, Simone Lanini, Fulvia Pimpinelli, Antonino Di Caro, Maria R. Capobianchi
EPI_ISL_603165	INMI Lazzaro Spallanzani IRCCS	INMI Lazzaro Spallanzani IRCCS	Martina Rueca, Francesco Messina, Barbara Bartolini, Cesare E.M. Gruber, Emanuela Giombini, Simone Lanini, Fulvia Pimpinelli, Maria R. Capobianchi, Antonino Di Caro
EPI_ISL_603166	INMI Lazzaro Spallanzani IRCCS	INMI Lazzaro Spallanzani IRCCS	Martina Rueca, Cesare E.M. Gruber, Barbara Bartolini, Francesco Messina, Simone Lanini, Emanuela Giombini, Fulvia Pimpinelli, Maria R. Capobianchi, Antonino Di Caro
EPI_ISL_603167	INMI Lazzaro Spallanzani IRCCS	INMI Lazzaro Spallanzani IRCCS	Martina Rueca, Cesare E.M. Gruber, Francesco Messina, Barbara Bartolini, Simone Lanini, Emanuela Giombini, Fulvia Pimpinelli, Maria R. Capobianchi, Antonino Di Caro
EPI_ISL_603168	INMI Lazzaro Spallanzani IRCCS	INMI Lazzaro Spallanzani IRCCS	Martina Rueca, Barbara Bartolini, Cesare E.M. Gruber, Simone Lanini, Emanuela Giombini, Francesco Messina, Fulvia Pimpinelli, Antonino Di Caro, Maria R. Capobianchi
EPI_ISL_603169	INMI Lazzaro Spallanzani IRCCS	INMI Lazzaro Spallanzani IRCCS	Francesco Messina, Barbara Bartolini, Cesare E.M. Gruber, Martina Rueca, Simone Lanini, Emanuela Giombini, Fulvia Pimpinelli, Maria R. Capobianchi, Antonino Di Caro
EPI_ISL_603170	INMI Lazzaro Spallanzani IRCCS	INMI Lazzaro Spallanzani IRCCS	Martina Rueca, Barbara Bartolini, Cesare E.M. Gruber, Francesco Messina, Simone Lanini, Emanuela Giombini, Maria R. Capobianchi, Antonino Di Caro
EPI_ISL_603171	INMI Lazzaro Spallanzani IRCCS	INMI Lazzaro Spallanzani IRCCS	Barbara Bartolini, Cesare E.M. Gruber, Francesco Messina, Martina Rueca, Emanuela Giombini, Simone Lanini, Mariarosa Gaudio, Antonino Di Caro, Maria R. Capobianchi
EPI_ISL_603172	INMI Lazzaro Spallanzani IRCCS	INMI Lazzaro Spallanzani IRCCS	Cesare E.M. Gruber, Martina Rueca, Barbara Bartolini, Simone Lanini, Francesco Messina, Emanuela Giombini, Mariarosa Gaudio, Antonino Di Caro, Maria R. Capobianchi
EPI_ISL_603173	INMI Lazzaro Spallanzani IRCCS	INMI Lazzaro Spallanzani IRCCS	Barbara Bartolini, Cesare E.M. Gruber, Martina Rueca, Francesco Messina, Simone Lanini, Emanuela Giombini, Giuseppina Cappiello, Maria R. Capobianchi, Antonino Di Caro
EPI_ISL_603174	INMI Lazzaro Spallanzani IRCCS	INMI Lazzaro Spallanzani IRCCS	Francesco Messina, Barbara Bartolini, Martina Rueca, Emanuela Giombini, Simone Lanini, Cesare E.M. Gruber, Mariarosa Gaudio, Maria R. Capobianchi, Antonino Di Caro
EPI_ISL_603175	INMI Lazzaro Spallanzani IRCCS	INMI Lazzaro Spallanzani IRCCS	Barbara Bartolini, Cesare E.M. Gruber, Simone Lanini, Emanuela Giombini, Francesco Messina, Martina Rueca, Mariarosa Gaudio, Antonino Di Caro, Maria R. Capobianchi
EPI_ISL_603176	INMI Lazzaro Spallanzani IRCCS	INMI Lazzaro Spallanzani IRCCS	Cesare E.M. Gruber, Barbara Bartolini, Francesco Messina, Martina Rueca, Simone Lanini, Emanuela Giombini, Antonino Di Caro, Maria R. Capobianchi
EPI_ISL_603177	INMI Lazzaro Spallanzani IRCCS	INMI Lazzaro Spallanzani IRCCS	Barbara Bartolini, Francesco Messina, Emanuela Giombini, Martina Rueca, Cesare E.M. Gruber, Simone Lanini, Maria R. Capobianchi, Antonino Di Caro
EPI_ISL_603178	INMI Lazzaro Spallanzani IRCCS	INMI Lazzaro Spallanzani IRCCS	Martina Rueca, Francesco Messina, Cesare E.M. Gruber, Barbara Bartolini, Simone Lanini, Emanuela Giombini, Antonino Di Caro, Maria R. Capobianchi
EPI_ISL_603179	INMI Lazzaro Spallanzani IRCCS	INMI Lazzaro Spallanzani IRCCS	Cesare E.M. Gruber, Barbara Bartolini, Francesco Messina, Martina Rueca, Emanuela Giombini, Simone Lanini, Antonino Di Caro, Maria R. Capobianchi
EPI_ISL_603180	INMI Lazzaro Spallanzani IRCCS	INMI Lazzaro Spallanzani IRCCS	Barbara Bartolini, Cesare E.M. Gruber, Francesco Messina, Simone Lanini, Martina Rueca, Emanuela Giombini, Maria R. Capobianchi, Antonino Di Caro
EPI_ISL_603181	INMI Lazzaro Spallanzani IRCCS	INMI Lazzaro Spallanzani IRCCS	Francesco Messina, Cesare E.M. Gruber, Barbara Bartolini, Martina Rueca, Simone Lanini, Emanuela Giombini, Maria R. Capobianchi, Antonino Di Caro
EPI_ISL_603182	INMI Lazzaro Spallanzani IRCCS	INMI Lazzaro Spallanzani IRCCS	Martina Rueca, Barbara Bartolini, Cesare E.M. Gruber, Francesco Messina, Emanuela Giombini, Simone Lanini, Maria R. Capobianchi, Antonino Di Caro
EPI_ISL_603183	INMI Lazzaro Spallanzani IRCCS	INMI Lazzaro Spallanzani IRCCS	Barbara Bartolini, Francesco Messina, Martina Rueca, Cesare E.M. Gruber, Emanuela Giombini, Simone Lanini, Maria R. Capobianchi, Antonino Di Caro
EPI_ISL_603184	INMI Lazzaro Spallanzani IRCCS	INMI Lazzaro Spallanzani IRCCS	Martina Rueca, Barbara Bartolini, Francesco Messina, Cesare E.M. Gruber, Emanuela Giombini, Simone Lanini, Maria R. Capobianchi, Antonino Di Caro
EPI_ISL_603185	INMI Lazzaro Spallanzani IRCCS	INMI Lazzaro Spallanzani IRCCS	Cesare E.M. Gruber, Martina Rueca, Barbara Bartolini, Francesco Messina, Simone Lanini, Emanuela Giombini, Maria R. Capobianchi, Antonino Di Caro

EPI_ISL_603186	INMI Lazzaro Spallanzani IRCCS	INMI Lazzaro Spallanzani IRCCS	Francesco Messina, Martina Rueca, Barbara Bartolini, Cesare E.M. Gruber, Emanuela Giombini, Simone Lanini, Maria R. Capobianchi, Antonino Di Caro
EPI_ISL_603187	INMI Lazzaro Spallanzani IRCCS	INMI Lazzaro Spallanzani IRCCS	Cesare E.M. Gruber, Martina Rueca, Francesco Messina, Barbara Bartolini, Simone Lanini, Emanuela Giombini, Antonino Di Caro, Maria R. Capobianchi
EPI_ISL_609989	INMI Lazzaro Spallanzani IRCCS	INMI Lazzaro Spallanzani IRCCS	C.E.M Gruber, B Bartolini, M Rueca, F Messina, E Giombini, A Di Caro, MR Capobianchi
EPI_ISL_609990	INMI Lazzaro Spallanzani IRCCS	INMI Lazzaro Spallanzani IRCCS	B Bartolini, C.E.M Gruber, M Rueca, F Messina, E Giombini, MR Capobianchi, A Di Caro
EPI_ISL_609991	INMI Lazzaro Spallanzani IRCCS	INMI Lazzaro Spallanzani IRCCS	M Rueca, B Bartolini, C.E.M Gruber, F Messina, E Giombini, A Di Caro, MR Capobianchi
EPI_ISL_609992	INMI Lazzaro Spallanzani IRCCS	INMI Lazzaro Spallanzani IRCCS	F Messina, E Giombini, M Rueca, B Bartolini, C.E.M Gruber, MR Capobianchi, A Di Caro
EPI_ISL_609993	INMI Lazzaro Spallanzani IRCCS	INMI Lazzaro Spallanzani IRCCS	E Giombini, M Rueca, B Bartolini, C.E.M Gruber, F Messina, A Di Caro, MR Capobianchi
EPI_ISL_609994	INMI Lazzaro Spallanzani IRCCS	INMI Lazzaro Spallanzani IRCCS	C.E.M Gruber, F Messina, M Rueca, B Bartolini, E Giombini, MR Capobianchi, A Di Caro
EPI_ISL_609995	INMI Lazzaro Spallanzani IRCCS	INMI Lazzaro Spallanzani IRCCS	E Giombini, C.E.M Gruber, M Rueca, B Bartolini, F Messina, A Di Caro, MR Capobianchi
EPI_ISL_609996	INMI Lazzaro Spallanzani IRCCS	INMI Lazzaro Spallanzani IRCCS	F Messina, M Rueca, B Bartolini, C.E.M Gruber, E Giombini, MR Capobianchi, A Di Caro
EPI_ISL_609997	INMI Lazzaro Spallanzani IRCCS	INMI Lazzaro Spallanzani IRCCS	M Rueca, B Bartolini, C.E.M Gruber, F Messina, E Giombini, A Di Caro, MR Capobianchi
EPI_ISL_609998	INMI Lazzaro Spallanzani IRCCS	INMI Lazzaro Spallanzani IRCCS	F Messina, B Bartolini, M Rueca, C.E.M Gruber, E Giombini, A Di Caro, MR Capobianchi
EPI_ISL_609999	INMI Lazzaro Spallanzani IRCCS	INMI Lazzaro Spallanzani IRCCS	B Bartolini, M Rueca, C.E.M Gruber, F Messina, E Giombini, MR Capobianchi, A Di Caro
EPI_ISL_613560	Laboratorio Biologia Molecolare Sars Cov2 - UOC Laboratorio Analisi - Servizio Medicina di Laboratorio, Ospedale "San Francesco" - ATS-ASSL Nuoro Via Mannironi 1, 08100 Nuoro	Laboratorio specialistico UOC Ematologia - Ospedale "San Francesco" - ATS-ASSL Nuoro Nuoro	Piras Giovanna, Fancello Tatiana, Asproni Rosanna, Fiamma Maura, Monne Maria Itria, Toja Alessandro, Sanna Filomena, Floris Anna Rita, Sulis Vincenzo, Palmas Angelo Domenico, Casu Gavino, Lo Maglio Iana, Mameli Giuseppe
EPI_ISL_613706	Laboratorio Biologia Molecolare Sars Cov2 - UOC Laboratorio Analisi - Servizio Medicina di Laboratorio, Ospedale "San Francesco" - ATS-ASSL Nuoro	Laboratorio specialistico UOC Ematologia - Ospedale "San Francesco" - ATS-ASSL Nuoro	Piras Giovanna, Fancello Tatiana, Asproni Rosanna, Fiamma Maura, Monne Maria Itria, Toja Alessandro, Sanna Filomena, Floris Anna Rita, Sulis Vincenzo, Palmas Angelo Domenico, Casu Gavino, Lo Maglio Iana, Mameli Giuseppe
EPI_ISL_613710	Laboratorio Biologia Molecolare Sars Cov2 - UOC Laboratorio Analisi - Servizio Medicina di Laboratorio, Ospedale "San Francesco" - ATS-ASSL Nuoro	Laboratorio specialistico UOC Ematologia - Ospedale "San Francesco" - ATS-ASSL Nuoro	Piras Giovanna, Fancello Tatiana, Asproni Rosanna, Fiamma Maura, Monne Maria Itria, Toja Alessandro, Sanna Filomena, Floris Anna Rita, Sulis Vincenzo, Palmas Angelo Domenico, Casu Gavino, Lo Maglio Iana, Mameli Giuseppe.
EPI_ISL_613953, EPI_ISL_613955, EPI_ISL_614396, EPI_ISL_614397, EPI_ISL_614398, EPI_ISL_614889	Laboratorio Biologia Molecolare Sars Cov2 - UOC Laboratorio Analisi - Servizio Medicina di Laboratorio, Ospedale "San Francesco" - ATS-ASSL Nuoro	Laboratorio specialistico UOC Ematologia - Ospedale "San Francesco" - ATS-ASSL Nuoro	Piras Giovanna, Fancello Tatiana, Asproni Rosanna, Fiamma Maura, Monne Maria Itria, Toja Alessandro, Sanna Filomena, Floris Anna Rita, Sulis Vincenzo, Palmas Angelo Domenico, Casu Gavino, Lo Maglio Iana, Mameli Giuseppe
EPI_ISL_636462, EPI_ISL_636463	ULSS6 Euganea	Istituto Zooprofilattico Sperimentale delle Venezie	Adelaide Milani, Alessia Schivo, Annalisa Salviato, Erika Giorgia Quaranta, Ambra Pastori, Bianca Zecchin, Alice Fusaro, Isabella Monne, Calogero Terregino, Antonia Ricci
EPI_ISL_636464	ULSS6 Piove di Sacco	Istituto Zooprofilattico Sperimentale delle Venezie	Adelaide Milani, Alessia Schivo, Annalisa Salviato, Erika Giorgia Quaranta, Ambra Pastori, Bianca Zecchin, Alice Fusaro, Isabella Monne, Calogero Terregino, Antonia Ricci
EPI_ISL_636465, EPI_ISL_636466	ULSS6 Euganea	Istituto Zooprofilattico Sperimentale delle Venezie	Adelaide Milani, Alessia Schivo, Annalisa Salviato, Erika Giorgia Quaranta, Ambra Pastori, Bianca Zecchin, Alice Fusaro, Isabella Monne, Calogero Terregino, Antonia Ricci
EPI_ISL_636467, EPI_ISL_636468, EPI_ISL_636469, EPI_ISL_636470	ULSS6 Distretto Padova Terme Colli	Istituto Zooprofilattico Sperimentale delle Venezie	Adelaide Milani, Alessia Schivo, Annalisa Salviato, Erika Giorgia Quaranta, Ambra Pastori, Bianca Zecchin, Alice Fusaro, Isabella Monne, Calogero Terregino, Antonia Ricci
EPI_ISL_636471, EPI_ISL_636472, EPI_ISL_636473	ULSS6 Piove di Sacco	Istituto Zooprofilattico Sperimentale delle Venezie	Adelaide Milani, Alessia Schivo, Annalisa Salviato, Erika Giorgia Quaranta, Ambra Pastori, Bianca Zecchin, Alice Fusaro, Isabella Monne, Calogero Terregino, Antonia Ricci
EPI_ISL_636474, EPI_ISL_636475	ULSS6 Distretto Padova Terme Colli	Istituto Zooprofilattico Sperimentale delle Venezie	Adelaide Milani, Alessia Schivo, Annalisa Salviato, Erika Giorgia Quaranta, Ambra Pastori, Bianca Zecchin, Alice Fusaro, Isabella Monne, Calogero Terregino, Antonia Ricci
EPI_ISL_636488	ULSS9 Scaligera	Istituto Zooprofilattico Sperimentale delle Venezie	Adelaide Milani, Alessia Schivo, Annalisa Salviato, Erika Giorgia Quaranta, Ambra Pastori, Bianca Zecchin, Alice Fusaro, Isabella Monne, Calogero Terregino, Antonia Ricci
EPI_ISL_637107, EPI_ISL_637108, EPI_ISL_637109	Laboratorio Biologia Molecolare Sars Cov2 - UOC Laboratorio Analisi - Servizio Medicina di Laboratorio, Ospedale "San Francesco" - ATS-ASSL Nuoro	Laboratorio specialistico UOC Ematologia - Ospedale "San Francesco" - ATS-ASSL Nuoro	Piras Giovanna, Fancello Tatiana, Asproni Rosanna, Fiamma Maura, Monne Maria Itria, Toja Alessandro, Sanna Filomena, Floris Anna Rita, Sulis Vincenzo, Palmas Angelo Domenico, Casu Gavino, Lo Maglio Iana, Mameli Giuseppe
EPI_ISL_649189, EPI_ISL_649190	Istituto Zooprofilattico Sperimentale della Puglia e della Basilicata	Istituto Zooprofilattico Sperimentale della Puglia e della Basilicata	Parisi A., Bianco A., Capozzi L., Del Sambro L., Manzulli V, Rondinone V., Pace L., Galante D., Cipolletta D.
EPI_ISL_649191	Istituto Zooprofilattico Sperimentale della Puglia e della Basilicata	Istituto Zooprofilattico Sperimentale della Puglia e della Basilicata	Parisi A., Bianco A., Capozzi L., Del Sambro L., Manzulli V, Rondinone V., Pace L., Cipolletta D., Galante D.
EPI_ISL_649785	I.R.C.C.S. "S. De Bellis" - Ente Ospedaliero	Istituto Zooprofilattico Sperimentale della Puglia e della Basilicata	Parisi A., Bianco A., Capozzi L., Del Sambro L., Lippolis A., Notarnicola M., Manzulli V, Rondinone V., Pace L.
EPI_ISL_649938	I.R.C.C.S. "S. De Bellis" - Ente Ospedaliero	Istituto Zooprofilattico Sperimentale della Puglia e della Basilicata	Parisi A., Bianco A., Capozzi L., Del Sambro L., Lippolis A., Notarnicola M., Cipolletta D., Galante D.
EPI_ISL_649939	I.R.C.C.S. "S. De Bellis" - Ente Ospedaliero	Istituto Zooprofilattico Sperimentale della Puglia e della Basilicata	Parisi A., Bianco A., Capozzi L., Del Sambro L., Lippolis A., Notarnicola M., Manzulli V, Rondinone V., Pace L.
EPI_ISL_649940	Istituto Zooprofilattico Sperimentale della Puglia e della Basilicata	Istituto Zooprofilattico Sperimentale della Puglia e della Basilicata	Parisi A., Bianco A., Capozzi L., Del Sambro L., Manzulli V, Rondinone V., Pace L., Cipolletta D., Galante D.
EPI_ISL_653763, EPI_ISL_653764, EPI_ISL_653765, EPI_ISL_653766, EPI_ISL_653767, EPI_ISL_653768, EPI_ISL_653769, EPI_ISL_653770, EPI_ISL_653771, EPI_ISL_653772, EPI_ISL_653773, EPI_ISL_653774, EPI_ISL_653775, EPI_ISL_653776, EPI_ISL_653777, EPI_ISL_653778, EPI_ISL_653779, EPI_ISL_653780, EPI_ISL_653781, EPI_ISL_653782, EPI_ISL_653783			
see above	I.R.C.C.S. "S. De Bellis" - Ente Ospedaliero	Istituto Zooprofilattico Sperimentale della Puglia e della Basilicata	Parisi A., Bianco A., Capozzi L., Del Sambro L., Lippolis A., Notarnicola M., Manzulli V, Rondinone V., Pace L.
EPI_ISL_653784, EPI_ISL_653785, EPI_ISL_653786	Istituto Zooprofilattico Sperimentale della Puglia e della Basilicata	Istituto Zooprofilattico Sperimentale della Puglia e della Basilicata	Parisi A., Bianco A., Capozzi L., Del Sambro L., Manzulli V, Rondinone V., Pace L., Cipolletta D., Galante D.
EPI_ISL_653787, EPI_ISL_653788, EPI_ISL_653789, EPI_ISL_653790, EPI_ISL_653791, EPI_ISL_653792, EPI_ISL_653793	I.R.C.C.S. "S. De Bellis" - Ente Ospedaliero	Istituto Zooprofilattico Sperimentale della Puglia e della Basilicata	Parisi A., Bianco A., Capozzi L., Del Sambro L., Lippolis A., Notarnicola M., Manzulli V, Rondinone V., Pace L.
EPI_ISL_653794, EPI_ISL_653795, EPI_ISL_653796, EPI_ISL_653797, EPI_ISL_653798, EPI_ISL_653799, EPI_ISL_653800, EPI_ISL_653801, EPI_ISL_653802, EPI_ISL_653803, EPI_ISL_653804, EPI_ISL_653805, EPI_ISL_653806, EPI_ISL_653807, EPI_ISL_653808, EPI_ISL_653809, EPI_ISL_653810, EPI_ISL_653811, EPI_ISL_653812			
see above	I.R.C.C.S. "S. De Bellis" - Ente Ospedaliero	Istituto Zooprofilattico Sperimentale della Puglia e della Basilicata	Parisi A., Bianco A., Capozzi L., Del Sambro L., Lippolis A., Notarnicola M., Cipolletta D., Galante D.
EPI_ISL_653813	Istituto Zooprofilattico Sperimentale della Puglia e della	Istituto Zooprofilattico Sperimentale della Puglia e della	Parisi A., Bianco A., Capozzi L., Del Sambro L., Manzulli V, Rondinone V., Pace L., Cipolletta D., Galante D.

	Basilicata	Basilicata	
EPI_ISL_653814, EPI_ISL_653815, EPI_ISL_653816, EPI_ISL_653817, EPI_ISL_653818, EPI_ISL_653819, EPI_ISL_653820, EPI_ISL_653821, EPI_ISL_653822, EPI_ISL_653823	I.R.C.C.S. "S. De Bellis" - Ente Ospedaliero	Istituto Zooprofilattico Sperimentale della Puglia e della Basilicata	Parisi A., Bianco A., Capozzi L., Del Sambro L., Lippolis A., Notarnicola M., Cipolletta D., Galante D.
EPI_ISL_710503	Laboratorio specialistico UOC Ematologia - Ospedale "San Francesco" - ATS-ASSL Nuoro	Laboratorio specialistico UOC Ematologia - Ospedale "San Francesco" - ATS-ASSL Nuoro	Giovanna Piras
EPI_ISL_710542	National Institute for Infectious Diseases, INMI, "L. Spallanzani" IRCCS	National Institute for Infectious Diseases, INMI, "L. Spallanzani" IRCCS	C.E.M Gruber, B Bartolini, M Rueca, F Messina, E Giombini, A Di Caro, MR Capobianchi
EPI_ISL_710543	National Institute for Infectious Diseases, INMI, "L. Spallanzani" IRCCS	National Institute for Infectious Diseases, INMI, "L. Spallanzani" IRCCS	B Bartolini, C.E.M Gruber, M Rueca, F Messina, E Giombini, MR Capobianchi, A Di Caro
EPI_ISL_710544	National Institute for Infectious Diseases, INMI, "L. Spallanzani" IRCCS	National Institute for Infectious Diseases, INMI, "L. Spallanzani" IRCCS	M Rueca, B Bartolini, C.E.M Gruber, F Messina, E Giombini, A Di Caro, MR Capobianchi
EPI_ISL_710545	National Institute for Infectious Diseases, INMI, "L. Spallanzani" IRCCS	National Institute for Infectious Diseases, INMI, "L. Spallanzani" IRCCS	F Messina, E Giombini, M Rueca, B Bartolini, C.E.M Gruber, MR Capobianchi, A Di Caro
EPI_ISL_710546	National Institute for Infectious Diseases, INMI, "L. Spallanzani" IRCCS	National Institute for Infectious Diseases, INMI, "L. Spallanzani" IRCCS	E Giombini, M Rueca, B Bartolini, C.E.M Gruber, F Messina, A Di Caro, MR Capobianchi
EPI_ISL_717978	Army Medical Center, Scientific Department, Virology Laboratory	Army Medical Center, Scientific Department, Virology Laboratory	Silvia Fillo, Giovanni Faggioni, Riccardo De Santis, Antonella Fortunato, Anna Anselmo, Vanessa Vera Fain, Francesco Giordani, Nino D'Amore, Anella Monte, Marzia Cavalli, Alessandra Amoroso, Stella Lia, Roberta Sorrentino, Rossella Tirelli, Federica Galeano, Annalisa Pelo, Margherita De Santis, Giulia Campoli, Andrea Ciammaruconi, Florigio Lista.
EPI_ISL_718262	National Institute for Infectious Diseases, INMI, "L. Spallanzani" IRCCS	National Institute for Infectious Diseases, INMI, "L. Spallanzani" IRCCS	C.E.M Gruber, F Messina, M Rueca, B Bartolini, E Giombini, MR Capobianchi, A Di Caro
EPI_ISL_718263	National Institute for Infectious Diseases, INMI, "L. Spallanzani" IRCCS	National Institute for Infectious Diseases, INMI, "L. Spallanzani" IRCCS	E Giombini, C.E.M Gruber, M Rueca, B Bartolini, F Messina, A Di Caro, MR Capobianchi
EPI_ISL_718264	National Institute for Infectious Diseases, INMI, "L. Spallanzani" IRCCS	National Institute for Infectious Diseases, INMI, "L. Spallanzani" IRCCS	F Messina, M Rueca, B Bartolini, C.E.M Gruber, E Giombini, MR Capobianchi, A Di Caro
EPI_ISL_718265	National Institute for Infectious Diseases, INMI, "L. Spallanzani" IRCCS	National Institute for Infectious Diseases, INMI, "L. Spallanzani" IRCCS	B Bartolini, M Rueca, C.E.M Gruber, F Messina, E Giombini, A Di Caro, MR Capobianchi
EPI_ISL_721624	National Institute for Infectious Diseases, INMI, "L. Spallanzani" IRCCS	National Institute for Infectious Diseases, INMI, "L. Spallanzani" IRCCS	C.E.M Gruber, B Bartolini, M Rueca, F Messina, E Giombini, A Di Caro, MR Capobianchi
EPI_ISL_721625	National Institute for Infectious Diseases, INMI, "L. Spallanzani" IRCCS	National Institute for Infectious Diseases, INMI, "L. Spallanzani" IRCCS	B Bartolini, C.E.M Gruber, M Rueca, F Messina, E Giombini, MR Capobianchi, A Di Caro
EPI_ISL_721626	National Institute for Infectious Diseases, INMI, "L. Spallanzani" IRCCS	National Institute for Infectious Diseases, INMI, "L. Spallanzani" IRCCS	M Rueca, B Bartolini, C.E.M Gruber, F Messina, E Giombini, A Di Caro, MR Capobianchi
EPI_ISL_721627	National Institute for Infectious Diseases, INMI, "L. Spallanzani" IRCCS	National Institute for Infectious Diseases, INMI, "L. Spallanzani" IRCCS	F Messina, E Giombini, M Rueca, B Bartolini, C.E.M Gruber, MR Capobianchi, A Di Caro
EPI_ISL_721628	National Institute for Infectious Diseases, INMI, "L. Spallanzani" IRCCS	National Institute for Infectious Diseases, INMI, "L. Spallanzani" IRCCS	E Giombini, M Rueca, B Bartolini, C.E.M Gruber, F Messina, A Di Caro, MR Capobianchi
EPI_ISL_722851	I.R.C.C.S. "S. De Bellis" - Ente Ospedaliero	Istituto Zooprofilattico Sperimentale della Puglia e della Basilicata	Parisi A., Bianco A., Capozzi L., Del Sambro L., Lippolis A., Notarnicola M., Manzulli V, Rondinone V., Pace L.
EPI_ISL_722852	I.R.C.C.S. "S. De Bellis" - Ente Ospedaliero	Istituto Zooprofilattico Sperimentale della Puglia e della Basilicata	Parisi A., Bianco A., Capozzi L., Del Sambro L., Lippolis A., Notarnicola M., Cipolletta D., Galante D.
EPI_ISL_722853	I.R.C.C.S. "S. De Bellis" - Ente Ospedaliero	Istituto Zooprofilattico Sperimentale della Puglia e della Basilicata	Parisi A., Bianco A., Capozzi L., Del Sambro L., Lippolis A., Notarnicola M., Manzulli V, Rondinone V., Pace L.
EPI_ISL_722854	I.R.C.C.S. "S. De Bellis" - Ente Ospedaliero	Istituto Zooprofilattico Sperimentale della Puglia e della Basilicata	Parisi A., Bianco A., Capozzi L., Del Sambro L., Lippolis A., Notarnicola M., Cipolletta D., Galante D.
EPI_ISL_722855, EPI_ISL_722856, EPI_ISL_722857, EPI_ISL_722858	Dipartimento di Scienze Biomediche e Oncologia Umana - Azienda Ospedaliero Universitaria Consorziale Policlinico	Istituto Zooprofilattico Sperimentale della Puglia e della Basilicata	Parisi A., Bianco A., Capozzi L., Del Sambro L., Chironna M., Loconsole D.
EPI_ISL_722859, EPI_ISL_722860	I.R.C.C.S. "S. De Bellis" - Ente Ospedaliero	Istituto Zooprofilattico Sperimentale della Puglia e della Basilicata	Parisi A., Bianco A., Capozzi L., Del Sambro L., Lippolis A., Notarnicola M., Manzulli V, Rondinone V., Pace L.
EPI_ISL_722861	I.R.C.C.S. "S. De Bellis" - Ente Ospedaliero	Istituto Zooprofilattico Sperimentale della Puglia e della Basilicata	Parisi A., Bianco A., Capozzi L., Del Sambro L., Lippolis A., Notarnicola M., Cipolletta D., Galante D.
EPI_ISL_722862	I.R.C.C.S. "S. De Bellis" - Ente Ospedaliero	Istituto Zooprofilattico Sperimentale della Puglia e della Basilicata	Parisi A., Bianco A., Capozzi L., Del Sambro L., Lippolis A., Notarnicola M., Manzulli V, Rondinone V., Pace L.
EPI_ISL_722863	I.R.C.C.S. "S. De Bellis" - Ente Ospedaliero	Istituto Zooprofilattico Sperimentale della Puglia e della Basilicata	Parisi A., Bianco A., Capozzi L., Del Sambro L., Lippolis A., Notarnicola M., Cipolletta D., Galante D.
EPI_ISL_722864, EPI_ISL_722865	I.R.C.C.S. "S. De Bellis" - Ente Ospedaliero	Istituto Zooprofilattico Sperimentale della Puglia e della Basilicata	Parisi A., Bianco A., Capozzi L., Del Sambro L., Lippolis A., Notarnicola M., Manzulli V, Rondinone V., Pace L.
EPI_ISL_722866	I.R.C.C.S. "S. De Bellis" - Ente Ospedaliero	Istituto Zooprofilattico Sperimentale della Puglia e della Basilicata	Parisi A., Bianco A., Capozzi L., Del Sambro L., Lippolis A., Notarnicola M., Cipolletta D., Galante D.
EPI_ISL_722867, EPI_ISL_722868	I.R.C.C.S. "S. De Bellis" - Ente Ospedaliero	Istituto Zooprofilattico Sperimentale della Puglia e della Basilicata	Parisi A., Bianco A., Capozzi L., Del Sambro L., Lippolis A., Notarnicola M., Manzulli V, Rondinone V., Pace L.
EPI_ISL_722869	I.R.C.C.S. "S. De Bellis" - Ente Ospedaliero	Istituto Zooprofilattico Sperimentale della Puglia e della Basilicata	Parisi A., Bianco A., Capozzi L., Del Sambro L., Lippolis A., Notarnicola M., Cipolletta D., Galante D.
EPI_ISL_722870	I.R.C.C.S. "S. De Bellis" - Ente Ospedaliero	Istituto Zooprofilattico Sperimentale della Puglia e della Basilicata	Parisi A., Bianco A., Capozzi L., Del Sambro L., Lippolis A., Notarnicola M., Manzulli V, Rondinone V., Pace L.
EPI_ISL_722871	I.R.C.C.S. "S. De Bellis" - Ente Ospedaliero	Istituto Zooprofilattico Sperimentale della Puglia e della Basilicata	Parisi A., Bianco A., Capozzi L., Del Sambro L., Lippolis A., Notarnicola M., Cipolletta D., Galante D.
EPI_ISL_722872	Dipartimento di Scienze Biomediche e Oncologia Umana - Azienda Ospedaliero Universitaria Consorziale Policlinico	Istituto Zooprofilattico Sperimentale della Puglia e della Basilicata	Parisi A., Bianco A., Capozzi L., Del Sambro L., Chironna M., Loconsole D.

EPI_ISL_722873, EPI_ISL_722874, EPI_ISL_722875, EPI_ISL_722876, EPI_ISL_722877, EPI_ISL_722878, EPI_ISL_722879, EPI_ISL_722880, EPI_ISL_722881, EPI_ISL_722882, EPI_ISL_722883, EPI_ISL_722884, EPI_ISL_722885, EPI_ISL_722886, EPI_ISL_722887, EPI_ISL_722888, EPI_ISL_722889, EPI_ISL_722890, EPI_ISL_722891, EPI_ISL_722892, EPI_ISL_722893, EPI_ISL_722894			
see above	Istituto Zooprofilattico Sperimentale della Puglia e della Basilicata	Istituto Zooprofilattico Sperimentale della Puglia e della Basilicata	Parisi A., Bianco A., Capozzi L., Del Sambro L., Manzulli V, Rondinone V., Pace L., Cipolletta D., Galante D.
EPI_ISL_722895	I.R.C.C.S. "S. De Bellis" - Ente Ospedaliero	Istituto Zooprofilattico Sperimentale della Puglia e della Basilicata	Parisi A., Bianco A., Capozzi L., Del Sambro L., Lippolis A., Notarnicola M., Cipolletta D., Galante D.
EPI_ISL_722896, EPI_ISL_722897	Dipartimento di Scienze Biomediche e Oncologia Umana - Azienda Ospedaliero Universitaria Consorziale Policlinico	Istituto Zooprofilattico Sperimentale della Puglia e della Basilicata	Parisi A., Bianco A., Capozzi L., Del Sambro L., Chironna M., Loconsole D.
EPI_ISL_722898, EPI_ISL_722900	I.R.C.C.S. "S. De Bellis" - Ente Ospedaliero	Istituto Zooprofilattico Sperimentale della Puglia e della Basilicata	Parisi A., Bianco A., Capozzi L., Del Sambro L., Lippolis A., Notarnicola M., Cipolletta D., Galante D.
EPI_ISL_722901, EPI_ISL_722902, EPI_ISL_722903, EPI_ISL_722904, EPI_ISL_722905, EPI_ISL_722906, EPI_ISL_722907, EPI_ISL_722908, EPI_ISL_722909, EPI_ISL_722910, EPI_ISL_722911, EPI_ISL_722913, EPI_ISL_722914, EPI_ISL_722915, EPI_ISL_722916, EPI_ISL_722917, EPI_ISL_722918, EPI_ISL_722919, EPI_ISL_722920, EPI_ISL_722921, EPI_ISL_722922, EPI_ISL_722923, EPI_ISL_722924, EPI_ISL_722925			
see above	Istituto Zooprofilattico Sperimentale della Puglia e della Basilicata	Istituto Zooprofilattico Sperimentale della Puglia e della Basilicata	Parisi A., Bianco A., Capozzi L., Del Sambro L., Manzulli V, Rondinone V., Pace L., Cipolletta D., Galante D.
EPI_ISL_728279	National Institute for Infectious Diseases, INMI, "L. Spallanzani" IRCCS	National Institute for Infectious Diseases, INMI, "L. Spallanzani" IRCCS	E Giombini, C.E.M Gruber, M Rueca, B Bartolini, F Messina, A Di Caro, MR Capobianchi
EPI_ISL_728280	National Institute for Infectious Diseases, INMI, "L. Spallanzani" IRCCS	National Institute for Infectious Diseases, INMI, "L. Spallanzani" IRCCS	F Messina, M Rueca, B Bartolini, C.E.M Gruber, E Giombini, MR Capobianchi, A Di Caro
EPI_ISL_728281	National Institute for Infectious Diseases, INMI, "L. Spallanzani" IRCCS	National Institute for Infectious Diseases, INMI, "L. Spallanzani" IRCCS	B Bartolini, M Rueca, C.E.M Gruber, F Messina, E Giombini, A Di Caro, MR Capobianchi
EPI_ISL_728282	National Institute for Infectious Diseases, INMI, "L. Spallanzani" IRCCS	National Institute for Infectious Diseases, INMI, "L. Spallanzani" IRCCS	M. Rueca, C.E.M Gruber, B Bartolini, F Messina, E Giombini, A Di Caro, MR Capobianchi
EPI_ISL_728283	National Institute for Infectious Diseases, INMI, "L. Spallanzani" IRCCS	National Institute for Infectious Diseases, INMI, "L. Spallanzani" IRCCS	B Bartolini, C.E.M Gruber, M Rueca, F Messina, E Giombini, MR Capobianchi, A Di Caro
EPI_ISL_728284	National Institute for Infectious Diseases, INMI, "L. Spallanzani" IRCCS	National Institute for Infectious Diseases, INMI, "L. Spallanzani" IRCCS	M Rueca, B Bartolini, C.E.M Gruber, F Messina, E Giombini, A Di Caro, MR Capobianchi
EPI_ISL_728285	National Institute for Infectious Diseases, INMI, "L. Spallanzani" IRCCS	National Institute for Infectious Diseases, INMI, "L. Spallanzani" IRCCS	F Messina, E Giombini, M Rueca, B Bartolini, C.E.M Gruber, MR Capobianchi, A Di Caro
EPI_ISL_728286	National Institute for Infectious Diseases, INMI, "L. Spallanzani" IRCCS	National Institute for Infectious Diseases, INMI, "L. Spallanzani" IRCCS	E Giombini, M Rueca, B Bartolini, C.E.M Gruber, F Messina, A Di Caro, MR Capobianchi
EPI_ISL_728287	National Institute for Infectious Diseases, INMI, "L. Spallanzani" IRCCS	National Institute for Infectious Diseases, INMI, "L. Spallanzani" IRCCS	C.E.M Gruber, F Messina, M Rueca, B Bartolini, E Giombini, MR Capobianchi, A Di Caro
EPI_ISL_730653	University of Bari, Valenzano, Italy	Istituto Zooprofilattico Sperimentale dell'Abruzzo e del Molise "G. Caporale".	N. Decaro, E. Lorusso, G. Elia, C. Desario, D. Buonavoglia, V., Martella, C. Buonavoglia, A. Lorusso, C. Cammà, V. Curini
EPI_ISL_735504	University of Bari Biomedical Sciences and Human Oncology	University of Bari Biomedical Sciences and Human Oncology	Maria Chironna, Anna Sallustio, Daniela Loconsole
EPI_ISL_735505, EPI_ISL_735506	University of Bari Biomedical Sciences and Human Oncology	University of Bari Biomedical Sciences and Human Oncology	Maria Chironna, Anna Sallustio, Daniela Loconsole, Marisa Accogli
EPI_ISL_735510	National Institute for Infectious Diseases, INMI, "L. Spallanzani" IRCCS	National Institute for Infectious Diseases, INMI, "L. Spallanzani" IRCCS	M. Rueca, C.E.M Gruber, E. Giombini, F. Messina, B. Bartolini, F. Carletti, A. Di Caro, M.R Capobianchi
EPI_ISL_735511	Clinical Pathology and Microbiology, San Gallicano Dermatologic Institute IRCCS	National Institute for Infectious Diseases, INMI, "L. Spallanzani" IRCCS	B. Bartolini, C.E.M Gruber, F. Messina, E. Giombini, M. Rueca, F. Carletti, F. Pimpinelli, F. Ensoli, M.R. Capobianchi, A. Di Caro
EPI_ISL_736777, EPI_ISL_736778, EPI_ISL_736779, EPI_ISL_736780, EPI_ISL_736781, EPI_ISL_736782, EPI_ISL_736783, EPI_ISL_736784, EPI_ISL_736785, EPI_ISL_736786, EPI_ISL_736787, EPI_ISL_736788, EPI_ISL_736789, EPI_ISL_736790, EPI_ISL_736791, EPI_ISL_736792, EPI_ISL_736793, EPI_ISL_736794, EPI_ISL_736795, EPI_ISL_736796, EPI_ISL_736797, EPI_ISL_736798, EPI_ISL_736799, EPI_ISL_736800, EPI_ISL_736801, EPI_ISL_736802, EPI_ISL_736803, EPI_ISL_736804, EPI_ISL_736805, EPI_ISL_736806, EPI_ISL_736807, EPI_ISL_736808, EPI_ISL_736809, EPI_ISL_736810, EPI_ISL_736811, EPI_ISL_736812, EPI_ISL_736813, EPI_ISL_736814, EPI_ISL_736815, EPI_ISL_736816, EPI_ISL_736817, EPI_ISL_736818, EPI_ISL_736819, EPI_ISL_736820, EPI_ISL_736821, EPI_ISL_736822, EPI_ISL_736823, EPI_ISL_736824, EPI_ISL_736825, EPI_ISL_736826, EPI_ISL_736827, EPI_ISL_736828, EPI_ISL_736829, EPI_ISL_736830, EPI_ISL_736831, EPI_ISL_736832, EPI_ISL_736833, EPI_ISL_736834, EPI_ISL_736835, EPI_ISL_736836, EPI_ISL_736837, EPI_ISL_736838, EPI_ISL_736839, EPI_ISL_736840, EPI_ISL_736841, EPI_ISL_736842, EPI_ISL_736843, EPI_ISL_736844, EPI_ISL_736845, EPI_ISL_736846, EPI_ISL_736847, EPI_ISL_736848, EPI_ISL_736849, EPI_ISL_736850, EPI_ISL_736851, EPI_ISL_736852, EPI_ISL_736853, EPI_ISL_736854, EPI_ISL_736855, EPI_ISL_736856, EPI_ISL_736857, EPI_ISL_736858, EPI_ISL_736859, EPI_ISL_736860, EPI_ISL_736861, EPI_ISL_736862, EPI_ISL_736863, EPI_ISL_736864, EPI_ISL_736865, EPI_ISL_736866, EPI_ISL_736867, EPI_ISL_736868, EPI_ISL_736869, EPI_ISL_736870, EPI_ISL_736871, EPI_ISL_736872, EPI_ISL_736873, EPI_ISL_736874, EPI_ISL_736875, EPI_ISL_736876, EPI_ISL_736877, EPI_ISL_736878, EPI_ISL_736879, EPI_ISL_736880, EPI_ISL_736881, EPI_ISL_736882, EPI_ISL_736883, EPI_ISL_736884, EPI_ISL_736885, EPI_ISL_736886, EPI_ISL_736887, EPI_ISL_736888, EPI_ISL_736889, EPI_ISL_736890			
see above	Istituto Zooprofilattico Sperimentale del Mezzogiorno	TIGEM	Antonio Grimaldi, Patrizia Annunziata, Francesco Panariello, Biancamaria Pierri, Valentina Bouche, Chiara Colantuono, Maria Concetta Cuomo, Denise Di Concilio, Lucio Di Filippo, Anna Manfredi, Marcello Salvi, Antonio Limone, Pellegrino Cerino, Andrea Ballabio, Davide Cacchiarelli.
EPI_ISL_736996	Istituto Zooprofilattico Sperimentale del Mezzogiorno	National Institute for Infectious Diseases, INMI, "L. Spallanzani" IRCCS	E. Giombini, C.E.M Gruber, F. Messina, M. Rueca, B. Bartolini, F. Carletti, P. Cerino, B. Pierri, C. Buonerba, D. Di Concilio, M.C. Cuomo, A. Di Caro, M.R Capobianchi
EPI_ISL_736997	Istituto Zooprofilattico Sperimentale del Mezzogiorno	National Institute for Infectious Diseases, INMI, "L. Spallanzani" IRCCS	F. Messina, C.E.M Gruber, M. Rueca, B. Bartolini, E. Giombini, F. Carletti, P. Cerino, B. Pierri, C. Buonerba, D. Di Concilio, M.C. Cuomo, M.R. Capobianchi, A. Di Caro
EPI_ISL_738044	SIESP CHIETI - DRIVE IN LANCIANO	Istituto Zooprofilattico Sperimentale dell'Abruzzo e Molise "G. Caporale"	Lorusso A, Marcacci M, Di Domenico M, Ancora M, Curini V, Mangone I, Rinaldi A, Di Pasquale A, Cammà C, Puglia I, Savini G
EPI_ISL_738045	SIESP DIPARTIMENTO DI PREVENZIONE CHIETI	Istituto Zooprofilattico Sperimentale dell'Abruzzo e Molise "G. Caporale"	Lorusso A, Marcacci M, Di Domenico M, Ancora M, Curini V, Mangone I, Rinaldi A, Di Pasquale A, Cammà C, Puglia I, Savini G
EPI_ISL_738046, EPI_ISL_738047, EPI_ISL_738048	SIESP CHIETI - DRIVE IN ORTONA	Istituto Zooprofilattico Sperimentale dell'Abruzzo e Molise "G. Caporale"	Lorusso A, Marcacci M, Di Domenico M, Ancora M, Curini V, Mangone I, Rinaldi A, Di Pasquale A, Cammà C, Puglia I, Savini G
EPI_ISL_738121, EPI_ISL_738122, EPI_ISL_738123, EPI_ISL_738124, EPI_ISL_738125, EPI_ISL_738126, EPI_ISL_738127, EPI_ISL_738128, EPI_ISL_738129, EPI_ISL_738130, EPI_ISL_738131, EPI_ISL_738132			
see above	IZSM-U.O.C. Virologia	Istituto Zooprofilattico Sperimentale del Mezzogiorno	Maurizio Viscardi, Lorena Cardillo, Giovanna Fusco
EPI_ISL_738144	University of Bari Biomedical Sciences and Human Oncology	University of Bari Biomedical Sciences and Human Oncology	Maria Chironna, Anna Sallustio, Daniela Loconsole, Marisa Accogli
EPI_ISL_738147, EPI_ISL_738194, EPI_ISL_738243	Microbiology and Virology Unit, Florence Careggi University Hospital	Microbiology and Virology Unit, Florence Careggi University Hospital	Vincenzo Di Pilato, Marco Coppi, Alberto Antonelli, Simona Pollini, Gian Maria Rossolini
EPI_ISL_745192	Ospedale Di Venere	Istituto Zooprofilattico Sperimentale della Puglia e della Basilicata	Parisi A., Capozzi L., Del Sambro L., Bianco A., Chiara M., Pesole G., De Sabato L., Iacobellis M.
EPI_ISL_745193	Ospedale Di Venere	Istituto Zooprofilattico Sperimentale della Puglia e della Basilicata	Parisi A., Bianco A., Capozzi L., Del Sambro L., Chiara M., De Sabato L., Pesole G., Iacobellis M
EPI_ISL_746826	National Institute for Infectious Diseases, INMI, "L. Spallanzani" IRCCS	National Institute for Infectious Diseases, INMI, "L. Spallanzani" IRCCS	C.E.M Gruber, B Bartolini, M Rueca, F Messina, E Giombini, A Di Caro, MR Capobianchi

EPI_ISL_746827	National Institute for Infectious Diseases, INMI, "L. Spallanzani" IRCCS	National Institute for Infectious Diseases, INMI, "L. Spallanzani" IRCCS	B Bartolini, C.E.M Gruber, M Rueca, F Messina, E Giombini, MR Capobianchi, A Di Caro
EPI_ISL_746828	National Institute for Infectious Diseases, INMI, "L. Spallanzani" IRCCS	National Institute for Infectious Diseases, INMI, "L. Spallanzani" IRCCS	M Rueca, B Bartolini, C.E.M Gruber, F Messina, E Giombini, A Di Caro, MR Capobianchi
EPI_ISL_746829	National Institute for Infectious Diseases, INMI, "L. Spallanzani" IRCCS	National Institute for Infectious Diseases, INMI, "L. Spallanzani" IRCCS	F Messina, E Giombini, M Rueca, B Bartolini, C.E.M Gruber, MR Capobianchi, A Di Caro
EPI_ISL_746830	National Institute for Infectious Diseases, INMI, "L. Spallanzani" IRCCS	National Institute for Infectious Diseases, INMI, "L. Spallanzani" IRCCS	E Giombini, M Rueca, B Bartolini, C.E.M Gruber, F Messina, A Di Caro, MR Capobianchi
EPI_ISL_746831	National Institute for Infectious Diseases, INMI, "L. Spallanzani" IRCCS	National Institute for Infectious Diseases, INMI, "L. Spallanzani" IRCCS	C.E.M Gruber, F Messina, M Rueca, B Bartolini, E Giombini, MR Capobianchi, A Di Caro
EPI_ISL_746832	National Institute for Infectious Diseases, INMI, "L. Spallanzani" IRCCS	National Institute for Infectious Diseases, INMI, "L. Spallanzani" IRCCS	E Giombini, C.E.M Gruber, M Rueca, B Bartolini, F Messina, A Di Caro, MR Capobianchi
EPI_ISL_746833	National Institute for Infectious Diseases, INMI, "L. Spallanzani" IRCCS	National Institute for Infectious Diseases, INMI, "L. Spallanzani" IRCCS	F Messina, M Rueca, B Bartolini, C.E.M Gruber, E Giombini, MR Capobianchi, A Di Caro
EPI_ISL_746834	National Institute for Infectious Diseases, INMI, "L. Spallanzani" IRCCS	National Institute for Infectious Diseases, INMI, "L. Spallanzani" IRCCS	B Bartolini, M Rueca, C.E.M Gruber, F Messina, E Giombini, A Di Caro, MR Capobianchi
EPI_ISL_746835	National Institute for Infectious Diseases, INMI, "L. Spallanzani" IRCCS	National Institute for Infectious Diseases, INMI, "L. Spallanzani" IRCCS	M Rueca, E Giombini, B Bartolini, C.E.M Gruber, F Messina, A Di Caro, MR Capobianchi
EPI_ISL_747459, EPI_ISL_747460, EPI_ISL_747461, EPI_ISL_747462, EPI_ISL_747463, EPI_ISL_747464	Ospedale San Bonifacio	Istituto Zooprofilattico Sperimentale delle Venezie	Adelaide Milani, Alessia Schivo, Annalisa Salviato, Erika Giorgia Quaranta, Ambra Pastori, Bianca Zecchin, Alice Fusaro, Isabella Monne, Calogero Terregino, Antonia Ricci
EPI_ISL_747465, EPI_ISL_747466, EPI_ISL_747467, EPI_ISL_747468, EPI_ISL_747469, EPI_ISL_747470	Ospedale Mater Salutis	Istituto Zooprofilattico Sperimentale delle Venezie	Adelaide Milani, Alessia Schivo, Annalisa Salviato, Erika Giorgia Quaranta, Ambra Pastori, Bianca Zecchin, Alice Fusaro, Isabella Monne, Calogero Terregino, Antonia Ricci
EPI_ISL_747471, EPI_ISL_747472, EPI_ISL_747473, EPI_ISL_747474, EPI_ISL_747475, EPI_ISL_747476	ULSS9 Distretto di Bussolengo	Istituto Zooprofilattico Sperimentale delle Venezie	Adelaide Milani, Alessia Schivo, Annalisa Salviato, Erika Giorgia Quaranta, Ambra Pastori, Bianca Zecchin, Alice Fusaro, Isabella Monne, Calogero Terregino, Antonia Ricci
EPI_ISL_747477, EPI_ISL_747478, EPI_ISL_747479, EPI_ISL_747480, EPI_ISL_747481	ULSS 5 Polesana	Istituto Zooprofilattico Sperimentale delle Venezie	Adelaide Milani, Alessia Schivo, Annalisa Salviato, Erika Giorgia Quaranta, Ambra Pastori, Bianca Zecchin, Alice Fusaro, Isabella Monne, Calogero Terregino, Antonia Ricci
EPI_ISL_747482, EPI_ISL_747483	Ospedale San Bonifacio	Istituto Zooprofilattico Sperimentale delle Venezie	Adelaide Milani, Alessia Schivo, Annalisa Salviato, Erika Giorgia Quaranta, Ambra Pastori, Bianca Zecchin, Alice Fusaro, Isabella Monne, Calogero Terregino, Antonia Ricci
EPI_ISL_747484, EPI_ISL_747485	Ospedale Mater Salutis	Istituto Zooprofilattico Sperimentale delle Venezie	Adelaide Milani, Alessia Schivo, Annalisa Salviato, Erika Giorgia Quaranta, Ambra Pastori, Bianca Zecchin, Alice Fusaro, Isabella Monne, Calogero Terregino, Antonia Ricci
EPI_ISL_747486, EPI_ISL_747487, EPI_ISL_747488, EPI_ISL_747489	ULSS 5 Polesana	Istituto Zooprofilattico Sperimentale delle Venezie	Adelaide Milani, Alessia Schivo, Annalisa Salviato, Erika Giorgia Quaranta, Ambra Pastori, Bianca Zecchin, Alice Fusaro, Isabella Monne, Calogero Terregino, Antonia Ricci
EPI_ISL_751318, EPI_ISL_751319, EPI_ISL_751320, EPI_ISL_751321, EPI_ISL_751322, EPI_ISL_751323, EPI_ISL_751324, EPI_ISL_751325, EPI_ISL_751326, EPI_ISL_751327, EPI_ISL_751328, EPI_ISL_751329, EPI_ISL_751330, EPI_ISL_751331, EPI_ISL_751332, EPI_ISL_751333, EPI_ISL_751334, EPI_ISL_751335, EPI_ISL_751336, EPI_ISL_751337, EPI_ISL_751338, EPI_ISL_751339, EPI_ISL_751340, EPI_ISL_751341, EPI_ISL_751342, EPI_ISL_751343, EPI_ISL_751344, EPI_ISL_751345, EPI_ISL_751346, EPI_ISL_751347, EPI_ISL_751348, EPI_ISL_751349, EPI_ISL_751350, EPI_ISL_751351, EPI_ISL_751352, EPI_ISL_751353, EPI_ISL_751354, EPI_ISL_751355, EPI_ISL_751356, EPI_ISL_751357, EPI_ISL_751358, EPI_ISL_751359, EPI_ISL_751360, EPI_ISL_751361, EPI_ISL_751362, EPI_ISL_751363, EPI_ISL_751364, EPI_ISL_751365, EPI_ISL_751366, EPI_ISL_751367, EPI_ISL_751368, EPI_ISL_751369, EPI_ISL_751370, EPI_ISL_751371, EPI_ISL_751372, EPI_ISL_751373, EPI_ISL_751374, EPI_ISL_751375, EPI_ISL_751376, EPI_ISL_751377, EPI_ISL_751378, EPI_ISL_751379, EPI_ISL_751380, EPI_ISL_751381, EPI_ISL_751382, EPI_ISL_751383, EPI_ISL_751384, EPI_ISL_751385, EPI_ISL_751386, EPI_ISL_751387, EPI_ISL_751388, EPI_ISL_751389, EPI_ISL_751390, EPI_ISL_751391, EPI_ISL_751392, EPI_ISL_751393, EPI_ISL_751394, EPI_ISL_751395, EPI_ISL_751396, EPI_ISL_751397, EPI_ISL_751398, EPI_ISL_751399, EPI_ISL_751400, EPI_ISL_751401, EPI_ISL_751402, EPI_ISL_751403, EPI_ISL_751404, EPI_ISL_751405, EPI_ISL_751406, EPI_ISL_751407, EPI_ISL_751408, EPI_ISL_751409, EPI_ISL_751410, EPI_ISL_751411, EPI_ISL_751412, EPI_ISL_751413, EPI_ISL_751414, EPI_ISL_751415, EPI_ISL_751416, EPI_ISL_751417, EPI_ISL_751418, EPI_ISL_751419, EPI_ISL_751420, EPI_ISL_751421, EPI_ISL_751422, EPI_ISL_751423, EPI_ISL_751424, EPI_ISL_751425, EPI_ISL_751426, EPI_ISL_751427, EPI_ISL_751428, EPI_ISL_751429, EPI_ISL_751430, EPI_ISL_751431, EPI_ISL_751432, EPI_ISL_751433, EPI_ISL_751434, EPI_ISL_751435, EPI_ISL_751436, EPI_ISL_751437, EPI_ISL_751438, EPI_ISL_751439, EPI_ISL_751440, EPI_ISL_751441, EPI_ISL_751442, EPI_ISL_751443, EPI_ISL_751444, EPI_ISL_751445, EPI_ISL_751446, EPI_ISL_751447			
see above	IRCCS Sacro Cuore Don Calabria Hospital, Department of Infectious, Tropical Diseases & Microbiology	University of Verona, Department of Biotechnology	Antonio Mori, Michela Deiana, Elena Pomari, Chiara Piubelli; Giulia Lopatriello, Luca Marcolungo, Cristina Beltrami, Chiara Degli Esposti, Emanuela Cosentino, Massimo Delledonne
EPI_ISL_755572, EPI_ISL_755573, EPI_ISL_755574	Center of Advanced Studies and Technology, CAST	Center of Advanced Studies and Technology, CAST	Ferrante,R., Mandatori,D., De Fabritiis,S.
EPI_ISL_763069, EPI_ISL_763071, EPI_ISL_763072, EPI_ISL_763073	Istituto Zooprofilattico Sperimentale dell' Umbria e delle Marche -Togo Rosati	Istituto Superiore di Sanità	Massimo Biagetti , Monica Giammarioli, Luca De Sabato, Gabriele Vaccari, Ilaria Di Bartolo, Giovanni Ianiro
EPI_ISL_763078, EPI_ISL_763079, EPI_ISL_763080, EPI_ISL_763081, EPI_ISL_763082, EPI_ISL_763083, EPI_ISL_763084, EPI_ISL_763085	Microbiologia e Virologia	Istituto Zooprofilattico Sperimentale delle Venezie	Adelaide Milani, Alessia Schivo, Annalisa Salviato, Erika Giorgia Quaranta, Ambra Pastori, Bianca Zecchin, Alice Fusaro, Isabella Monne, Calogero Terregino, Antonia Ricci
EPI_ISL_763094, EPI_ISL_763095, EPI_ISL_763096, EPI_ISL_763097, EPI_ISL_763098, EPI_ISL_763138, EPI_ISL_763318, EPI_ISL_763320, EPI_ISL_763322, EPI_ISL_763323, EPI_ISL_763324, EPI_ISL_763325, EPI_ISL_763326, EPI_ISL_763327, EPI_ISL_763328, EPI_ISL_763329, EPI_ISL_763330			
see above	Istituto Zooprofilattico Sperimentale dell' Umbria e delle Marche -Togo Rosati	Istituto Superiore di Sanità	Massimo Biagetti , Monica Giammarioli, Luca De Sabato, Gabriele Vaccari, Ilaria Di Bartolo, Giovanni Ianiro
EPI_ISL_765567	National Institute for Infectious Diseases, INMI, "L. Spallanzani" IRCCS	National Institute for Infectious Diseases, INMI, "L. Spallanzani" IRCCS	E Giombini, C.E.M Gruber, M Rueca, B Bartolini, O Butera, F Messina, A Di Caro, G Parisi, MR Capobianchi
EPI_ISL_765568	National Institute for Infectious Diseases, INMI, "L. Spallanzani" IRCCS	National Institute for Infectious Diseases, INMI, "L. Spallanzani" IRCCS	O Butera, C.E.M Gruber, F Messina, M Rueca, B Bartolini, E Giombini, MR Capobianchi, A Di Caro
EPI_ISL_765569	National Institute for Infectious Diseases, INMI, "L. Spallanzani" IRCCS	National Institute for Infectious Diseases, INMI, "L. Spallanzani" IRCCS	B Bartolini, M Rueca, O Butera, C.E.M Gruber, F Messina, E Giombini, A Di Caro, MR Capobianchi
EPI_ISL_765570	National Institute for Infectious Diseases, INMI, "L. Spallanzani" IRCCS	National Institute for Infectious Diseases, INMI, "L. Spallanzani" IRCCS	M. Rueca, C.E.M Gruber, B Bartolini, O Butera, F Messina, E Giombini, A Di Caro, MR Capobianchi
EPI_ISL_765571	National Institute for Infectious Diseases, INMI, "L. Spallanzani" IRCCS	National Institute for Infectious Diseases, INMI, "L. Spallanzani" IRCCS	E Giombini, B Bartolini, O Butera, C.E.M Gruber, M Rueca, F Messina, MR Capobianchi, A Di Caro
EPI_ISL_765572	National Institute for Infectious Diseases, INMI, "L. Spallanzani" IRCCS	National Institute for Infectious Diseases, INMI, "L. Spallanzani" IRCCS	C.E.M Gruber, M Rueca, B Bartolini, F Messina, E Giombini,O Butera, A Di Caro, MR Capobianchi
EPI_ISL_765573	National Institute for Infectious Diseases, INMI, "L. Spallanzani" IRCCS	National Institute for Infectious Diseases, INMI, "L. Spallanzani" IRCCS	F Messina, E Giombini, M Rueca, B Bartolini, C.E.M Gruber, MR Capobianchi, O Butera, A Di Caro
EPI_ISL_766571, EPI_ISL_766572, EPI_ISL_766573	ULSS 7 Pedemontana - Distretto 1	Istituto Zooprofilattico Sperimentale delle Venezie	Adelaide Milani, Alessia Schivo, Annalisa Salviato, Erika Giorgia Quaranta, Ambra Pastori, Bianca Zecchin, Alice Fusaro, Isabella Monne, Calogero Terregino, Antonia Ricci

EPI_ISL_766574, EPI_ISL_766575, EPI_ISL_766576, EPI_ISL_766577, EPI_ISL_766578, EPI_ISL_766579	ULSS 8 Berica	Istituto Zooprofilattico Sperimentale delle Venezie	Adelaide Milani, Alessia Schivo, Annalisa Salviato, Erika Giorgia Quaranta, Ambra Pastori, Bianca Zecchin, Alice Fusaro, Isabella Monne, Calogero Terregino, Antonia Ricci
EPI_ISL_766580, EPI_ISL_766581	ULSS 2 Treviso	Istituto Zooprofilattico Sperimentale delle Venezie	Adelaide Milani, Alessia Schivo, Annalisa Salviato, Erika Giorgia Quaranta, Ambra Pastori, Bianca Zecchin, Alice Fusaro, Isabella Monne, Calogero Terregino, Antonia Ricci
EPI_ISL_767013, EPI_ISL_767043	University of Bari Biomedical Sciences and Human Oncology	University of Bari Biomedical Sciences and Human Oncology	Maria Chironna, Anna Sallustio, Daniela Loconsole, Marisa Accogli
EPI_ISL_775239, EPI_ISL_775240, EPI_ISL_775243, EPI_ISL_775244, EPI_ISL_775246, EPI_ISL_775257, EPI_ISL_775259, EPI_ISL_775261	INT Fondazione Pascale	INT Fondazione Pascale	INT Fondazione Pascale
EPI_ISL_776879, EPI_ISL_776880, EPI_ISL_776881, EPI_ISL_776882, EPI_ISL_776883, EPI_ISL_776884, EPI_ISL_776885, EPI_ISL_776886, EPI_ISL_776887, EPI_ISL_776888, EPI_ISL_776889, EPI_ISL_776890, EPI_ISL_776891, EPI_ISL_776892, EPI_ISL_776893, EPI_ISL_776894, EPI_ISL_776895, EPI_ISL_776896, EPI_ISL_776897, EPI_ISL_776898, EPI_ISL_776899, EPI_ISL_776900, EPI_ISL_776901, EPI_ISL_776902, EPI_ISL_776903, EPI_ISL_776904, EPI_ISL_776905, EPI_ISL_776906, EPI_ISL_776907, EPI_ISL_776908, EPI_ISL_776909, EPI_ISL_776910, EPI_ISL_776911, EPI_ISL_776912, EPI_ISL_776913, EPI_ISL_776914, EPI_ISL_776915, EPI_ISL_776916, EPI_ISL_776917, EPI_ISL_776918, EPI_ISL_776919, EPI_ISL_776920, EPI_ISL_776921, EPI_ISL_776922, EPI_ISL_776923, EPI_ISL_776924, EPI_ISL_776925, EPI_ISL_776926, EPI_ISL_776927, EPI_ISL_776928, EPI_ISL_776929, EPI_ISL_776930, EPI_ISL_776931, EPI_ISL_776932, EPI_ISL_776933, EPI_ISL_776934, EPI_ISL_776935, EPI_ISL_776936, EPI_ISL_776937, EPI_ISL_776938, EPI_ISL_776939, EPI_ISL_776940, EPI_ISL_776941, EPI_ISL_776942, EPI_ISL_776943, EPI_ISL_776944, EPI_ISL_776945, EPI_ISL_776946, EPI_ISL_776947, EPI_ISL_776948, EPI_ISL_776949, EPI_ISL_776950, EPI_ISL_776951, EPI_ISL_776952, EPI_ISL_776953, EPI_ISL_776954, EPI_ISL_776955, EPI_ISL_776956, EPI_ISL_776957, EPI_ISL_776958, EPI_ISL_776959, EPI_ISL_776960, EPI_ISL_776961, EPI_ISL_776962, EPI_ISL_776963, EPI_ISL_776964, EPI_ISL_776965, EPI_ISL_776966, EPI_ISL_776967, EPI_ISL_776968, EPI_ISL_776969, EPI_ISL_776971, EPI_ISL_776972, EPI_ISL_776973, EPI_ISL_776974, EPI_ISL_776975, EPI_ISL_776976, EPI_ISL_776977, EPI_ISL_776978, EPI_ISL_776979, EPI_ISL_776980, EPI_ISL_776981, EPI_ISL_776982, EPI_ISL_776983, EPI_ISL_776984, EPI_ISL_776985, EPI_ISL_776986, EPI_ISL_776987, EPI_ISL_776988, EPI_ISL_776989, EPI_ISL_776990, EPI_ISL_776991, EPI_ISL_776992, EPI_ISL_776993, EPI_ISL_776995, EPI_ISL_776996, EPI_ISL_776997, EPI_ISL_776998, EPI_ISL_776999, EPI_ISL_777000, EPI_ISL_777001, EPI_ISL_777002, EPI_ISL_777003, EPI_ISL_777004, EPI_ISL_777005, EPI_ISL_777006, EPI_ISL_777007, EPI_ISL_777008, EPI_ISL_777009, EPI_ISL_778646, EPI_ISL_778647, EPI_ISL_778648, EPI_ISL_778649, EPI_ISL_778650, EPI_ISL_778651, EPI_ISL_778652, EPI_ISL_778653, EPI_ISL_778654, EPI_ISL_778655, EPI_ISL_778656, EPI_ISL_778657, EPI_ISL_778658, EPI_ISL_778659, EPI_ISL_778660, EPI_ISL_778661, EPI_ISL_778662, EPI_ISL_778663, EPI_ISL_778664, EPI_ISL_778665, EPI_ISL_778666, EPI_ISL_778667, EPI_ISL_778668, EPI_ISL_778669, EPI_ISL_778670, EPI_ISL_778671, EPI_ISL_778672, EPI_ISL_778673, EPI_ISL_778674, EPI_ISL_778675, EPI_ISL_778676, EPI_ISL_778677, EPI_ISL_778678, EPI_ISL_778679, EPI_ISL_778680, EPI_ISL_778681, EPI_ISL_778682, EPI_ISL_778683, EPI_ISL_778684, EPI_ISL_778685, EPI_ISL_778686, EPI_ISL_778687, EPI_ISL_778688, EPI_ISL_778689, EPI_ISL_778690, EPI_ISL_778691, EPI_ISL_778692, EPI_ISL_778693, EPI_ISL_778694, EPI_ISL_778695, EPI_ISL_778696, EPI_ISL_778697, EPI_ISL_778698, EPI_ISL_778699, EPI_ISL_778700, EPI_ISL_778701, EPI_ISL_778702, EPI_ISL_778703, EPI_ISL_778704, EPI_ISL_778705, EPI_ISL_778706, EPI_ISL_778707, EPI_ISL_778708, EPI_ISL_778709, EPI_ISL_778710, EPI_ISL_778711, EPI_ISL_778712, EPI_ISL_778713, EPI_ISL_778714, EPI_ISL_778715, EPI_ISL_778716, EPI_ISL_778717, EPI_ISL_778718, EPI_ISL_778719, EPI_ISL_778720, EPI_ISL_778721, EPI_ISL_778722, EPI_ISL_778723, EPI_ISL_778724, EPI_ISL_778725, EPI_ISL_778726, EPI_ISL_778727, EPI_ISL_778728, EPI_ISL_778729, EPI_ISL_778730, EPI_ISL_778731, EPI_ISL_778732, EPI_ISL_778733, EPI_ISL_778734, EPI_ISL_778735, EPI_ISL_778736, EPI_ISL_778737, EPI_ISL_778738, EPI_ISL_778739, EPI_ISL_778740, EPI_ISL_778741, EPI_ISL_778742, EPI_ISL_778743, EPI_ISL_778744, EPI_ISL_778745, EPI_ISL_778746, EPI_ISL_778747, EPI_ISL_778748, EPI_ISL_778749, EPI_ISL_778750, EPI_ISL_778751, EPI_ISL_778752, EPI_ISL_778753, EPI_ISL_778754, EPI_ISL_778755, EPI_ISL_778756, EPI_ISL_778757, EPI_ISL_778758, EPI_ISL_778759, EPI_ISL_778760, EPI_ISL_778761, EPI_ISL_778762, EPI_ISL_778763, EPI_ISL_778764, EPI_ISL_778765, EPI_ISL_778766, EPI_ISL_778767, EPI_ISL_778768, EPI_ISL_778769, EPI_ISL_778770, EPI_ISL_778771, EPI_ISL_778772, EPI_ISL_778773, EPI_ISL_778774, EPI_ISL_778775, EPI_ISL_778776, EPI_ISL_778777, EPI_ISL_778778, EPI_ISL_778779, EPI_ISL_778780, EPI_ISL_778781, EPI_ISL_778782, EPI_ISL_778783, EPI_ISL_778784, EPI_ISL_778785, EPI_ISL_778786, EPI_ISL_778787, EPI_ISL_778788, EPI_ISL_778789, EPI_ISL_778790, EPI_ISL_778791, EPI_ISL_778792, EPI_ISL_778793, EPI_ISL_778794, EPI_ISL_778795, EPI_ISL_778796, EPI_ISL_778797, EPI_ISL_778798, EPI_ISL_778799, EPI_ISL_778800, EPI_ISL_778801, EPI_ISL_778802, EPI_ISL_778803, EPI_ISL_778804, EPI_ISL_778805, EPI_ISL_778806, EPI_ISL_778807, EPI_ISL_778808, EPI_ISL_778809, EPI_ISL_778810, EPI_ISL_778811, EPI_ISL_778812, EPI_ISL_778813, EPI_ISL_778814, EPI_ISL_778815, EPI_ISL_778816, EPI_ISL_778817			
see above	Istituto Zooprofilattico Sperimentale del Mezzogiorno	TIGEM	Antonio Grimaldi, Patrizia Annunziata, Francesco Panariello, Biancamaria Pierri, Valentina Bouche, Chiara Colantuono, Maria Concetta Cuomo, Denise Di Concilio, Lucio Di Filippo, Anna Manfredi, Marcello Salvi, Antonio Limone, Pellegrino Cerino, Andrea Ballabio, Davide Cacchiarelli.
EPI_ISL_778860	Laboratory of Infectious Diseases, Department of Biomedical and Clinical Sciences L. Sacco, University of Milan	Laboratory of Infectious Diseases, Department of Biomedical and Clinical Sciences L. Sacco, University of Milan	Alessia Lai, Annalisa Bergna, Carla Della Ventura, Claudia Balotta, Massimo Galli, Gianguglielmo Zehender on behalf of SARS-CoV-2 ITALIAN RESEARCH ENTERPRISE-(SCIRE) Collaborative Group
EPI_ISL_778868	Virology Lab, Ospedali Riuniti di Ancona	Dipartimento di Scienze Biomediche e Cliniche, L.Sacco, Università di Milano	Alessia Lai, Annalisa Bergna, Gianni Zehender, Claudia Balotta, Sara Caucci, Laura Di Sante, Roberta Longo, Sofia Maria Luigia Tiano, Massimo Galli, Patrizia Bagnarelli, Stefano Menzo
EPI_ISL_778869	Virology Lab, Ospedali Riuniti, Ancona	Dipartimento di Scenze Biomediche e Cliniche, L. Sacco, Università di Milano	Alessia Lai, Annalisa Bergna, Gianni Zehender, Claudia Balotta, Sara Caucci, Laura Di Sante, Roberta Longo, Sofia Maria Luigia Tiano, Massimo Galli, Patrizia Bagnarelli, Stefano Menzo
EPI_ISL_779704, EPI_ISL_779707, EPI_ISL_779709, EPI_ISL_779712, EPI_ISL_779713, EPI_ISL_779714, EPI_ISL_780375, EPI_ISL_780376, EPI_ISL_780378, EPI_ISL_780381, EPI_ISL_780411, EPI_ISL_780412, EPI_ISL_780413			
see above	Laboratory of Infectious Diseases, Department of Biomedical and Clinical Sciences L. Sacco, University of Milan	Laboratory of Infectious Diseases, Department of Biomedical and Clinical Sciences L. Sacco, University of Milan	Alessia Lai, Annalisa Bergna, Carla Della Ventura, Claudia Balotta, Massimo Galli, Gianguglielmo Zehender on behalf of SARS-CoV-2 ITALIAN RESEARCH ENTERPRISE-(SCIRE) Collaborative Group
EPI_ISL_788943, EPI_ISL_788944, EPI_ISL_788945, EPI_ISL_788946, EPI_ISL_788947, EPI_ISL_788948, EPI_ISL_788949, EPI_ISL_788950, EPI_ISL_788951, EPI_ISL_788952, EPI_ISL_788953, EPI_ISL_788954, EPI_ISL_788955, EPI_ISL_788956, EPI_ISL_788957, EPI_ISL_788958, EPI_ISL_788959, EPI_ISL_788960, EPI_ISL_788961, EPI_ISL_788962, EPI_ISL_788963, EPI_ISL_788964, EPI_ISL_788965, EPI_ISL_788966, EPI_ISL_788967, EPI_ISL_788968, EPI_ISL_788969, EPI_ISL_788970, EPI_ISL_788971, EPI_ISL_788972, EPI_ISL_788973, EPI_ISL_788974, EPI_ISL_788975, EPI_ISL_788976, EPI_ISL_788977, EPI_ISL_788978			
see above	Ospedale "Di Venere"	Beaconlab (Bioinformatics, Evolution and Comparative Genomics lab), Dept of Biosciences, University on Milan	Iacobellis M, d'Avenia M, Piluscio R, Parisi A, Chiara M, Manzari C, Pesole G
EPI_ISL_794745	Istituto Zooprofilattico Sperimentale della Puglia e della Basilicata	Istituto Zooprofilattico Sperimentale della Puglia e della Basilicata	Parisi A., Bianco A., Capozzi L., Del Sambro L., Manzulli V, Rondinone V., Pace L., Cipolletta D., Galante D.
EPI_ISL_794746	SISP ASL BA AREA SUD	Istituto Zooprofilattico Sperimentale della Puglia e della Basilicata	Parisi A., Bianco A., Capozzi L., Del Sambro L., Manzulli V, Rondinone V., Pace L., Cipolletta D., Galante D.
EPI_ISL_794747	Ospedale Vito Fazzi	Istituto Zooprofilattico Sperimentale della Puglia e della Basilicata	Parisi A., Bianco A., Capozzi L., Del Sambro L., Manzulli V, Rondinone V., Pace L., Cipolletta D., Galante D.
EPI_ISL_794748	SISP ASL BA AREA SUD	Istituto Zooprofilattico Sperimentale della Puglia e della Basilicata	Parisi A., Bianco A., Capozzi L., Del Sambro L., Manzulli V, Rondinone V., Pace L., Cipolletta D., Galante D.
EPI_ISL_794749	Istituto Zooprofilattico Sperimentale della Puglia e della Basilicata	Istituto Zooprofilattico Sperimentale della Puglia e della Basilicata	Parisi A., Bianco A., Capozzi L., Del Sambro L., Manzulli V, Rondinone V., Pace L., Cipolletta D., Galante D.
EPI_ISL_794750	Ospedale Santa Caterina Novella	Istituto Zooprofilattico Sperimentale della Puglia e della Basilicata	Parisi A., Bianco A., Capozzi L., Del Sambro L., Manzulli V, Rondinone V., Pace L., Cipolletta D., Galante D.
EPI_ISL_794751	Ospedale Vito Fazzi	Istituto Zooprofilattico Sperimentale della Puglia e della Basilicata	Parisi A., Bianco A., Capozzi L., Del Sambro L., Manzulli V, Rondinone V., Pace L., Cipolletta D., Galante D.
EPI_ISL_794752	Istituto Zooprofilattico Sperimentale della Puglia e della Basilicata	Istituto Zooprofilattico Sperimentale della Puglia e della Basilicata	Parisi A., Bianco A., Capozzi L., Del Sambro L., Manzulli V, Rondinone V., Pace L., Cipolletta D., Galante D.
EPI_ISL_794753	Ospedale Cardinale G. Panico	Istituto Zooprofilattico Sperimentale della Puglia e della Basilicata	Parisi A., Bianco A., Capozzi L., Del Sambro L., Manzulli V, Rondinone V., Pace L., Cipolletta D., Galante D.
EPI_ISL_794754, EPI_ISL_794755	Istituto Zooprofilattico Sperimentale della Puglia e della Basilicata	Istituto Zooprofilattico Sperimentale della Puglia e della Basilicata	Parisi A., Bianco A., Capozzi L., Del Sambro L., Manzulli V, Rondinone V., Pace L., Cipolletta D., Galante D.
EPI_ISL_794756, EPI_ISL_794757, EPI_ISL_794758	Presidio di Brindisi Di Summa - Perrino	Istituto Zooprofilattico Sperimentale della Puglia e della Basilicata	Parisi A., Bianco A., Capozzi L., Del Sambro L., Manzulli V, Rondinone V., Pace L., Cipolletta D., Galante D.
EPI_ISL_794759, EPI_ISL_794760, EPI_ISL_794761, EPI_ISL_794762, EPI_ISL_794763, EPI_ISL_794764, EPI_ISL_794765, EPI_ISL_794766, EPI_ISL_794767, EPI_ISL_794768, EPI_ISL_794769, EPI_ISL_794770, EPI_ISL_794771, EPI_ISL_794772, EPI_ISL_794773, EPI_ISL_794774, EPI_ISL_794775, EPI_ISL_794776, EPI_ISL_794777, EPI_ISL_794778, EPI_ISL_794779, EPI_ISL_794780, EPI_ISL_794781, EPI_ISL_794782, EPI_ISL_794783, EPI_ISL_794784, EPI_ISL_794785, EPI_ISL_794786, EPI_ISL_794787, EPI_ISL_794788, EPI_ISL_794789, EPI_ISL_794790, EPI_ISL_794791, EPI_ISL_794792, EPI_ISL_794793, EPI_ISL_794794, EPI_ISL_794795, EPI_ISL_794796, EPI_ISL_794797, EPI_ISL_794798, EPI_ISL_794799, EPI_ISL_794800, EPI_ISL_794801, EPI_ISL_794802, EPI_ISL_794803, EPI_ISL_794804, EPI_ISL_794805, EPI_ISL_794806, EPI_ISL_794807, EPI_ISL_794808, EPI_ISL_794809, EPI_ISL_794810, EPI_ISL_794811, EPI_ISL_794812, EPI_ISL_794813, EPI_ISL_794814, EPI_ISL_794815, EPI_ISL_794816, EPI_ISL_794817			
see above	Istituto Zooprofilattico Sperimentale della Puglia e della Basilicata	Istituto Zooprofilattico Sperimentale della Puglia e della Basilicata	Parisi A., Bianco A., Capozzi L., Del Sambro L., Manzulli V, Rondinone V., Pace L., Cipolletta D., Galante D.
EPI_ISL_794826, EPI_ISL_794827, EPI_ISL_794828	ULSS 7 Pedemontana - Distretto 2	Istituto Zooprofilattico Sperimentale delle Venezie	Adelaide Milani, Alessia Schivo, Annalisa Salviato, Erika Giorgia Quaranta, Ambra Pastori, Bianca Zecchin, Alice Fusaro, Isabella Monne, Calogero Terregino, Antonia Ricci
EPI_ISL_803880, EPI_ISL_803881, EPI_ISL_803882, EPI_ISL_803883, EPI_ISL_803884, EPI_ISL_803885, EPI_ISL_803886, EPI_ISL_803887, EPI_ISL_803888, EPI_ISL_803891, EPI_ISL_803892, EPI_ISL_803893, EPI_ISL_803895			

see above	Department of Medical Biotechnologies, University of Siena	Laboratory of Infectious Diseases, Department of Biomedical and Clinical Sciences L. Sacco, University of Milan	Ilaria Vicenti, Filippo Dragoni, Maurizio Zazzi, Maria Grazia Cusi, Alessia Lai, Annalisa Bergna, Carla Della Ventura, Claudia Balotta, Massimo Galli, Gianguglielmo Zehender on behalf of SARS-CoV-2 ITALIAN RESEARCH ENTERPRISE-(SCIRE) Collaborative Group
EPI_ISL_803896	Virology Unit, Pisa University Hospital and Retrovirus Center, University of Pisa	National Institute for Infectious Diseases, INMI, "L. Spallanzani" IRCCS	M Rueca, E Giombini, C.E.M Gruber, B Bartolini, O Butera, F Messina, A Rosellini, P Mazzetti, M Pistello, A Di Caro, MR Capobianchi
EPI_ISL_803897	National Institute for Infectious Diseases, INMI, "L. Spallanzani" IRCCS	National Institute for Infectious Diseases, INMI, "L. Spallanzani" IRCCS	F Messina, O Butera, E Giombini, M Rueca, B Bartolini, C.E.M Gruber, MR Capobianchi, A Di Caro
EPI_ISL_803898	National Institute for Infectious Diseases, INMI, "L. Spallanzani" IRCCS	National Institute for Infectious Diseases, INMI, "L. Spallanzani" IRCCS	C.E.M Gruber, B Bartolini, E Giombini, M Rueca, O Butera, F Messina, A Di Caro, MR Capobianchi
EPI_ISL_803899	Genomic Medicine Laboratory, IRCCS Santa Lucia Foundation	National Institute for Infectious Diseases, INMI, "L. Spallanzani" IRCCS	E Giombini, M. Rueca, B Bartolini, O Butera, C.E.M Gruber, F Messina, E Giardina, MR Capobianchi, A Di Caro
EPI_ISL_803900	National Institute for Infectious Diseases, INMI, "L. Spallanzani" IRCCS	National Institute for Infectious Diseases, INMI, "L. Spallanzani" IRCCS	B Bartolini, O Butera, C.E.M Gruber, M Rueca, F Messina, E Giombini, MR Capobianchi, A Di Caro
EPI_ISL_804038, EPI_ISL_804040, EPI_ISL_804041, EPI_ISL_804042, EPI_ISL_804044, EPI_ISL_804048, EPI_ISL_804051, EPI_ISL_804052, EPI_ISL_804053, EPI_ISL_804054	SC (UCO) Igiene e Sanità Pubblica (funzione integrata con SC Microbiologia e Virologia) e Laboratory of Molecular Virology of the International Centre for Genetic Engineering and Biotechnology (ICGEB)	ARGO Laboratorio Genomica ed Epigenomica	Licastro D, Dal Monego S, Degasperi M, Marcello A, D'Agaro P
EPI_ISL_806730	Presidio Ospedaliero S.Liberatore Atri	Istituto Zooprofilattico Sperimentale dell'Abruzzo e Molise "G. Caporale"	Lorusso A, Marcacci M, Di Domenico M, Ancora M, Curini V, Mangone I, Rinaldi A, Di Pasquale A, Cammà C, Puglia I, Calistri P, Savini G
EPI_ISL_806731, EPI_ISL_806732	SIESP DIPARTIMENTO DI PREVENZIONE CHIETI	Istituto Zooprofilattico Sperimentale dell'Abruzzo e Molise "G. Caporale"	Lorusso A, Marcacci M, Di Domenico M, Ancora M, Curini V, Mangone I, Rinaldi A, Di Pasquale A, Cammà C, Puglia I, Calistri P, Savini G
EPI_ISL_806733	SIESP CHIETI - DRIVE IN CHIETI	Istituto Zooprofilattico Sperimentale dell'Abruzzo e Molise "G. Caporale"	Lorusso A, Marcacci M, Di Domenico M, Ancora M, Curini V, Mangone I, Rinaldi A, Di Pasquale A, Cammà C, Puglia I, Calistri P, Savini G
EPI_ISL_806734	SIESP CHIETI - DRIVE IN LANCIANO	Istituto Zooprofilattico Sperimentale dell'Abruzzo e Molise "G. Caporale"	Lorusso A, Marcacci M, Di Domenico M, Ancora M, Curini V, Mangone I, Rinaldi A, Di Pasquale A, Cammà C, Puglia I, Calistri P, Savini G
EPI_ISL_806735, EPI_ISL_806736, EPI_ISL_806737, EPI_ISL_806738, EPI_ISL_806739, EPI_ISL_806740, EPI_ISL_806741, EPI_ISL_806742, EPI_ISL_806743, EPI_ISL_806744	SIESP DIPARTIMENTO DI PREVENZIONE TERAMO	Istituto Zooprofilattico Sperimentale dell'Abruzzo e Molise "G. Caporale"	Lorusso A, Marcacci M, Di Domenico M, Ancora M, Curini V, Mangone I, Rinaldi A, Di Pasquale A, Cammà C, Puglia I, Calistri P, Savini G
EPI_ISL_806745	RSA Giulianova	Istituto Zooprofilattico Sperimentale dell'Abruzzo e Molise "G. Caporale"	Lorusso A, Marcacci M, Di Domenico M, Ancora M, Curini V, Mangone I, Rinaldi A, Di Pasquale A, Cammà C, Puglia I, Calistri P, Savini G
EPI_ISL_806746, EPI_ISL_806747, EPI_ISL_806748	SIESP DIPARTIMENTO DI PREVENZIONE TERAMO	Istituto Zooprofilattico Sperimentale dell'Abruzzo e Molise "G. Caporale"	Lorusso A, Marcacci M, Di Domenico M, Ancora M, Curini V, Mangone I, Rinaldi A, Di Pasquale A, Cammà C, Puglia I, Calistri P, Savini G
EPI_ISL_806749, EPI_ISL_806750, EPI_ISL_806751, EPI_ISL_806752, EPI_ISL_806753, EPI_ISL_806754, EPI_ISL_806755, EPI_ISL_806756, EPI_ISL_806757	SIESP DIPARTIMENTO DI PREVENZIONE SULMONA	Istituto Zooprofilattico Sperimentale dell'Abruzzo e Molise "G. Caporale"	Lorusso A, Marcacci M, Di Domenico M, Ancora M, Curini V, Mangone I, Rinaldi A, Di Pasquale A, Cammà C, Puglia I, Calistri P, Savini G
EPI_ISL_806758, EPI_ISL_806759, EPI_ISL_806760, EPI_ISL_806761	DIPARTIMENTO PREVENZIONE AVEZZANO-SERVIZIO DI IGIENE EPIDEMIOLOGIA E SANITA' PUBBLICA	Istituto Zooprofilattico Sperimentale dell'Abruzzo e Molise "G. Caporale"	Lorusso A, Marcacci M, Di Domenico M, Ancora M, Curini V, Mangone I, Rinaldi A, Di Pasquale A, Cammà C, Puglia I, Calistri P, Savini G
EPI_ISL_806762	SIESP CHIETI - DRIVE IN ORTONA	Istituto Zooprofilattico Sperimentale dell'Abruzzo e Molise "G. Caporale"	Lorusso A, Marcacci M, Di Domenico M, Ancora M, Curini V, Mangone I, Rinaldi A, Di Pasquale A, Cammà C, Puglia I, Calistri P, Savini G
EPI_ISL_806763, EPI_ISL_806764, EPI_ISL_806765	DIPARTIMENTO PREVENZIONE AVEZZANO-SERVIZIO DI IGIENE EPIDEMIOLOGIA E SANITA' PUBBLICA	Istituto Zooprofilattico Sperimentale dell'Abruzzo e Molise "G. Caporale"	Lorusso A, Marcacci M, Di Domenico M, Ancora M, Curini V, Mangone I, Rinaldi A, Di Pasquale A, Cammà C, Puglia I, Calistri P, Savini G
EPI_ISL_806766, EPI_ISL_806767, EPI_ISL_806768, EPI_ISL_806769, EPI_ISL_806770, EPI_ISL_806771, EPI_ISL_806772	SIESP DIPARTIMENTO DI PREVENZIONE TERAMO	Istituto Zooprofilattico Sperimentale dell'Abruzzo e Molise "G. Caporale"	Lorusso A, Marcacci M, Di Domenico M, Ancora M, Curini V, Mangone I, Rinaldi A, Di Pasquale A, Cammà C, Puglia I, Calistri P, Savini G
EPI_ISL_806773, EPI_ISL_806774	Ospedale S.Salvatore-Medicina Interna L'Aquila	Istituto Zooprofilattico Sperimentale dell'Abruzzo e Molise "G. Caporale"	Lorusso A, Marcacci M, Di Domenico M, Ancora M, Curini V, Mangone I, Rinaldi A, Di Pasquale A, Cammà C, Puglia I, Calistri P, Savini G
EPI_ISL_806775, EPI_ISL_806776	SIESP CHIETI - DRIVE IN LANCIANO	Istituto Zooprofilattico Sperimentale dell'Abruzzo e Molise "G. Caporale"	Lorusso A, Marcacci M, Di Domenico M, Ancora M, Curini V, Mangone I, Rinaldi A, Di Pasquale A, Cammà C, Puglia I, Calistri P, Savini G
EPI_ISL_806777, EPI_ISL_806778, EPI_ISL_806779, EPI_ISL_806780	SIESP DIPARTIMENTO DI PREVENZIONE TERAMO	Istituto Zooprofilattico Sperimentale dell'Abruzzo e Molise "G. Caporale"	Lorusso A, Marcacci M, Di Domenico M, Ancora M, Curini V, Mangone I, Rinaldi A, Di Pasquale A, Cammà C, Puglia I, Calistri P, Savini G
EPI_ISL_806781, EPI_ISL_806782	DIPARTIMENTO PREVENZIONE AVEZZANO-SERVIZIO DI IGIENE EPIDEMIOLOGIA E SANITA' PUBBLICA	Istituto Zooprofilattico Sperimentale dell'Abruzzo e Molise "G. Caporale"	Lorusso A, Marcacci M, Di Domenico M, Ancora M, Curini V, Mangone I, Rinaldi A, Di Pasquale A, Cammà C, Puglia I, Calistri P, Savini G
EPI_ISL_806783	Casa di cura Di Lorenzo- Avezzano	Istituto Zooprofilattico Sperimentale dell'Abruzzo e Molise "G. Caporale"	Lorusso A, Marcacci M, Di Domenico M, Ancora M, Curini V, Mangone I, Rinaldi A, Di Pasquale A, Cammà C, Puglia I, Calistri P, Savini G
EPI_ISL_806784, EPI_ISL_806785	Ospedale Civile G.Mazzini-Teramo	Istituto Zooprofilattico Sperimentale dell'Abruzzo e Molise "G. Caporale"	Lorusso A, Marcacci M, Di Domenico M, Ancora M, Curini V, Mangone I, Rinaldi A, Di Pasquale A, Cammà C, Puglia I, Calistri P, Savini G
EPI_ISL_806786, EPI_ISL_806787, EPI_ISL_806788	SIESP DIPARTIMENTO DI PREVENZIONE TERAMO	Istituto Zooprofilattico Sperimentale dell'Abruzzo e Molise "G. Caporale"	Lorusso A, Marcacci M, Di Domenico M, Ancora M, Curini V, Mangone I, Rinaldi A, Di Pasquale A, Cammà C, Puglia I, Calistri P, Savini G
EPI_ISL_806789	Ospedale Civile Giulianova	Istituto Zooprofilattico Sperimentale dell'Abruzzo e Molise "G. Caporale"	Lorusso A, Marcacci M, Di Domenico M, Ancora M, Curini V, Mangone I, Rinaldi A, Di Pasquale A, Cammà C, Puglia I, Calistri P, Savini G
EPI_ISL_806790	SIESP DIPARTIMENTO DI PREVENZIONE DELL'AQUILA	Istituto Zooprofilattico Sperimentale dell'Abruzzo e Molise "G. Caporale"	Lorusso A, Marcacci M, Di Domenico M, Ancora M, Curini V, Mangone I, Rinaldi A, Di Pasquale A, Cammà C, Puglia I, Calistri P, Savini G
EPI_ISL_806791, EPI_ISL_806792, EPI_ISL_806793, EPI_ISL_806794, EPI_ISL_806795, EPI_ISL_806796, EPI_ISL_806797, EPI_ISL_806798	SIESP CHIETI - DRIVE IN ORTONA	Istituto Zooprofilattico Sperimentale dell'Abruzzo e Molise "G. Caporale"	Lorusso A, Marcacci M, Di Domenico M, Ancora M, Curini V, Mangone I, Rinaldi A, Di Pasquale A, Cammà C, Puglia I, Calistri P, Savini G
EPI_ISL_806799	SIESP CHIETI - DRIVE IN LANCIANO	Istituto Zooprofilattico Sperimentale dell'Abruzzo e Molise "G. Caporale"	Lorusso A, Marcacci M, Di Domenico M, Ancora M, Curini V, Mangone I, Rinaldi A, Di Pasquale A, Cammà C, Puglia I, Calistri P, Savini G

EPI_ISL_806800, EPI_ISL_806801, EPI_ISL_806802, EPI_ISL_806803	SIESP CHIETI - DRIVE IN GISSI	Istituto Zooprofilattico Sperimentale dell'Abruzzo e Molise "G. Caporale"	Lorusso A, Marcacci M, Di Domenico M, Ancora M, Curini V, Mangone I, Rinaldi A, Di Pasquale A, Cammà C, Puglia I, Calistri P, Savini G
EPI_ISL_806804, EPI_ISL_806805	SIESP CHIETI - DISTRETTO SANITARIO CHIETI	Istituto Zooprofilattico Sperimentale dell'Abruzzo e Molise "G. Caporale"	Lorusso A, Marcacci M, Di Domenico M, Ancora M, Curini V, Mangone I, Rinaldi A, Di Pasquale A, Cammà C, Puglia I, Calistri P, Savini G
EPI_ISL_806806	SIESP DIPARTIMENTO DI PREVENZIONE CHIETI	Istituto Zooprofilattico Sperimentale dell'Abruzzo e Molise "G. Caporale"	Lorusso A, Marcacci M, Di Domenico M, Ancora M, Curini V, Mangone I, Rinaldi A, Di Pasquale A, Cammà C, Puglia I, Calistri P, Savini G
EPI_ISL_812968	Department of Molecular Medicine, University of Padova	Department of Molecular Medicine, University of Padova	Lavezzo,E., Franchin,E., Barzon,L., Del Vecchio,C., Rossi,L.,Manganelli,R., Loregian,A., Abate,D., Sciro,M., De Canale,E.,Vanuzzo,M.C., Besutti,V., Saluzzo,F., Onelia,F., Pacenti,M.,Manuto,L., Parisi,S.G., Masi,G., Trevisan,M., Toppo,S. and Crisanti,A.
EPI_ISL_824407	Istituto Nazionale Malattie Infettive Lazzaro Spallanzani IRCCS	Istituto Nazionale Malattie Infettive Lazzaro Spallanzani IRCCS	Emanuela Giombini, Ornella Butera, Cesare E.M. Gruber, Martina Rueca, Francesco Messina, Barbara Bartolini, Silvia Meschi, Francesca Colavita, Concetta Castilletti, Antonino Di Caro, Maria R. Capobianchi
EPI_ISL_824408	Istituto Nazionale Malattie Infettive Lazzaro Spallanzani IRCCS	Istituto Nazionale Malattie Infettive Lazzaro Spallanzani IRCCS	Barbara Bartolini, Ornella Butera, Cesare E.M. Gruber, Martina Rueca, Francesco Messina, Emanuela Giombini, Silvia Meschi, Francesca Colavita, Concetta Castilletti, Maria R. Capobianchi, Antonino Di Caro
EPI_ISL_825155, EPI_ISL_825156	Microbiology and Virology Unit, Florence Careggi University Hospital	Microbiology and Virology Unit, Florence Careggi University Hospital	Vincenzo Di Pilato, Marco Coppi, Fabio Morecchiato, Alberto Antonelli, Emanuele Gori, Gian Maria Rossolini
EPI_ISL_826284, EPI_ISL_826458	University of Bari Biomedical Sciences and Human Oncology	University of Bari Biomedical Sciences and Human Oncology	Chironna Maria, Sallustio Anna, Loconsole Daniela, Accogli Marisa
EPI_ISL_826520	University of Bari Biomedical Sciences and Human Oncology	University of Bari Biomedical Sciences and Human Oncology	Chironna M, Sallustio A., Loconsole D., Accogli M.
EPI_ISL_832815, EPI_ISL_832816	SIESP CHIETI - DRIVE IN LANCIANO	Istituto Zooprofilattico Sperimentale dell'Abruzzo e Molise "G.Caporale"	Lorusso A, Marcacci M, Di Domenico M, Curini V, Ancora M, Cammà C, Rinaldi A, Mangone I, Di Pasquale A, Puglia I, Calistri P, Savini G.
EPI_ISL_832817	SIESP DIPARTIMENTO DI PREVENZIONE TERAMO	Istituto Zooprofilattico Sperimentale dell'Abruzzo e Molise "G.Caporale"	Lorusso A, Marcacci M, Di Domenico M, Curini V, Ancora M, Cammà C, Rinaldi A, Mangone I, Di Pasquale A, Puglia I, Calistri P, Savini G.
EPI_ISL_832818	Ospedale Civile Atri	Istituto Zooprofilattico Sperimentale dell'Abruzzo e Molise "G.Caporale"	Lorusso A, Marcacci M, Di Domenico M, Curini V, Ancora M, Cammà C, Rinaldi A, Mangone I, Di Pasquale A, Puglia I, Calistri P, Savini G.
EPI_ISL_832819	SIESP DIPARTIMENTO DI PREVENZIONE SULMONA	Istituto Zooprofilattico Sperimentale dell'Abruzzo e Molise "G.Caporale"	Lorusso A, Marcacci M, Di Domenico M, Curini V, Ancora M, Cammà C, Rinaldi A, Mangone I, Di Pasquale A, Puglia I, Calistri P, Savini G.
EPI_ISL_832820	Medico Competente P.O. L'Aquila	Istituto Zooprofilattico Sperimentale dell'Abruzzo e Molise "G.Caporale"	Lorusso A, Marcacci M, Di Domenico M, Curini V, Ancora M, Cammà C, Rinaldi A, Mangone I, Di Pasquale A, Puglia I, Calistri P, Savini G.
EPI_ISL_832821	SIESP CHIETI - DRIVE IN CHIETI	Istituto Zooprofilattico Sperimentale dell'Abruzzo e Molise "G.Caporale"	Lorusso A, Marcacci M, Di Domenico M, Curini V, Ancora M, Cammà C, Rinaldi A, Mangone I, Di Pasquale A, Puglia I, Calistri P, Savini G.
EPI_ISL_832822	SIESP CHIETI - DRIVE IN LANCIANO	Istituto Zooprofilattico Sperimentale dell'Abruzzo e Molise "G.Caporale"	Lorusso A, Marcacci M, Di Domenico M, Curini V, Ancora M, Cammà C, Rinaldi A, Mangone I, Di Pasquale A, Puglia I, Calistri P, Savini G.
EPI_ISL_833042	OSPEDALE SAN SALVATORE L'AQUILA UOC PNEUMOLOGIAE UTSIR	Istituto Zooprofilattico Sperimentale dell'Abruzzo e Molise "G. Caporale"	Lorusso A, Marcacci M, Di Domenico M, Ancora M, Curini V, Mangone I, Rinaldi A, Delli Compagni E, Di Pasquale A, Cammà C, Puglia I, Calistri P, Savini G
EPI_ISL_833043	SIESP DIPARTIMENTO DI PREVENZIONE CHIETI	Istituto Zooprofilattico Sperimentale dell'Abruzzo e Molise "G. Caporale"	Lorusso A, Marcacci M, Di Domenico M, Ancora M, Curini V, Mangone I, Rinaldi A, Delli Compagni E, Di Pasquale A, Cammà C, Puglia I, Calistri P, Savini G
EPI_ISL_833044	SIESP CHIETI - DRIVE IN CHIETI	Istituto Zooprofilattico Sperimentale dell'Abruzzo e Molise "G. Caporale"	Lorusso A, Marcacci M, Di Domenico M, Ancora M, Curini V, Mangone I, Rinaldi A, Delli Compagni E, Di Pasquale A, Cammà C, Puglia I, Calistri P, Savini G
EPI_ISL_833045	SIESP CHIETI - DRIVE IN ORTONA	Istituto Zooprofilattico Sperimentale dell'Abruzzo e Molise "G. Caporale"	Lorusso A, Marcacci M, Di Domenico M, Ancora M, Curini V, Mangone I, Rinaldi A, Delli Compagni E, Di Pasquale A, Cammà C, Puglia I, Calistri P, Savini G
EPI_ISL_833046	FPAM	Istituto Zooprofilattico Sperimentale dell'Abruzzo e Molise "G. Caporale"	Lorusso A, Marcacci M, Di Domenico M, Ancora M, Curini V, Mangone I, Rinaldi A, Delli Compagni E, Di Pasquale A, Cammà C, Puglia I, Calistri P, Savini G
EPI_ISL_833047, EPI_ISL_833048	OSPEDALE CIVILE ATRI	Istituto Zooprofilattico Sperimentale dell'Abruzzo e Molise "G. Caporale"	Lorusso A, Marcacci M, Di Domenico M, Ancora M, Curini V, Mangone I, Rinaldi A, Delli Compagni E, Di Pasquale A, Cammà C, Puglia I, Calistri P, Savini G
EPI_ISL_833049	SIESP CHIETI - DRIVE IN LANCIANO	Istituto Zooprofilattico Sperimentale dell'Abruzzo e Molise "G. Caporale"	Lorusso A, Marcacci M, Di Domenico M, Ancora M, Curini V, Mangone I, Rinaldi A, Delli Compagni E, Di Pasquale A, Cammà C, Puglia I, Calistri P, Savini G
EPI_ISL_833050, EPI_ISL_833051	DIP. PREV. AVEZZANO SERVIZIO DI IGIENE EPIDEMIOLOGIAE SANITA' PUBBLICA	Istituto Zooprofilattico Sperimentale dell'Abruzzo e Molise "G. Caporale"	Lorusso A, Marcacci M, Di Domenico M, Ancora M, Curini V, Mangone I, Rinaldi A, Delli Compagni E, Di Pasquale A, Cammà C, Puglia I, Calistri P, Savini G
EPI_ISL_833052, EPI_ISL_833053, EPI_ISL_833054	"Presidio Ospedaliero "San Liberatore" Atri	Istituto Zooprofilattico Sperimentale dell'Abruzzo e Molise "G. Caporale"	Lorusso A, Marcacci M, Di Domenico M, Ancora M, Curini V, Mangone I, Rinaldi A, Delli Compagni E, Di Pasquale A, Cammà C, Puglia I, Calistri P, Savini G
EPI_ISL_833055, EPI_ISL_833056	"SIESP DIPARTIMENTO DI PREVENZIONE CHIETi	Istituto Zooprofilattico Sperimentale dell'Abruzzo e Molise "G. Caporale"	Lorusso A, Marcacci M, Di Domenico M, Ancora M, Curini V, Mangone I, Rinaldi A, Delli Compagni E, Di Pasquale A, Cammà C, Puglia I, Calistri P, Savini G
EPI_ISL_833057, EPI_ISL_833058, EPI_ISL_833059, EPI_ISL_833060, EPI_ISL_833061, EPI_ISL_833062, EPI_ISL_833063, EPI_ISL_833064, EPI_ISL_833065, EPI_ISL_833066, EPI_ISL_833067			
see above	DIP. PREV. AVEZZANO SERVIZIO DI IGIENE EPIDEMIOLOGIAE SANITA' PUBBLICA	Istituto Zooprofilattico Sperimentale dell'Abruzzo e Molise "G. Caporale"	Lorusso A, Marcacci M, Di Domenico M, Ancora M, Curini V, Mangone I, Rinaldi A, Delli Compagni E, Di Pasquale A, Cammà C, Puglia I, Calistri P, Savini G
EPI_ISL_833068, EPI_ISL_833069, EPI_ISL_833070, EPI_ISL_833071, EPI_ISL_833072, EPI_ISL_833073, EPI_ISL_833074, EPI_ISL_833075	FPAM	Istituto Zooprofilattico Sperimentale dell'Abruzzo e Molise "G. Caporale"	Lorusso A, Marcacci M, Di Domenico M, Ancora M, Curini V, Mangone I, Rinaldi A, Delli Compagni E, Di Pasquale A, Cammà C, Puglia I, Calistri P, Savini G
EPI_ISL_833076	IZSAM	Istituto Zooprofilattico Sperimentale dell'Abruzzo e Molise "G. Caporale"	Lorusso A, Marcacci M, Di Domenico M, Ancora M, Curini V, Mangone I, Rinaldi A, Delli Compagni E, Di Pasquale A, Cammà C, Puglia I, Calistri P, Savini G
EPI_ISL_833077, EPI_ISL_833078	OSPEDALE CIVILE ATRI	Istituto Zooprofilattico Sperimentale dell'Abruzzo e Molise "G. Caporale"	Lorusso A, Marcacci M, Di Domenico M, Ancora M, Curini V, Mangone I, Rinaldi A, Delli Compagni E, Di Pasquale A, Cammà C, Puglia I, Calistri P, Savini G
EPI_ISL_833079, EPI_ISL_833080	OSPEDALE CIVILE TERAMO	Istituto Zooprofilattico Sperimentale dell'Abruzzo e Molise "G. Caporale"	Lorusso A, Marcacci M, Di Domenico M, Ancora M, Curini V, Mangone I, Rinaldi A, Delli Compagni E, Di Pasquale A, Cammà C, Puglia I, Calistri P, Savini G
EPI_ISL_833081	OSPEDALE S.S. ANNUNZIATA CHIETI	Istituto Zooprofilattico Sperimentale dell'Abruzzo e Molise "G. Caporale"	Lorusso A, Marcacci M, Di Domenico M, Ancora M, Curini V, Mangone I, Rinaldi A, Delli Compagni E, Di Pasquale A, Cammà C, Puglia I, Calistri P, Savini G
EPI_ISL_833082	OSPEDALE SAN SALVATORE	Istituto Zooprofilattico Sperimentale dell'Abruzzo e Molise "G. Caporale"	Lorusso A, Marcacci M, Di Domenico M, Ancora M, Curini V, Mangone I, Rinaldi A, Delli Compagni E, Di Pasquale A, Cammà C, Puglia I, Calistri P, Savini G
EPI_ISL_833083, EPI_ISL_833084	RPS	Istituto Zooprofilattico Sperimentale dell'Abruzzo e Molise "G. Caporale"	Lorusso A, Marcacci M, Di Domenico M, Ancora M, Curini V, Mangone I, Rinaldi A, Delli Compagni E, Di Pasquale A, Cammà C, Puglia I, Calistri P, Savini G
EPI_ISL_833085, EPI_ISL_833086	Servizio di Igiene Epidemiologia e Sanità Pubblica (SIESP)	Istituto Zooprofilattico Sperimentale dell'Abruzzo e Molise "G.	Lorusso A, Marcacci M, Di Domenico M, Ancora M, Curini V, Mangone I, Rinaldi A, Delli Compagni E, Di Pasquale A, Cammà C, Puglia I, Calistri P, Savini

	CHIETI - DRIVE IN GISSI	Caporale"	G
EPI_ISL_833087	Servizio di Igiene Epidemiologia e Sanità Pubblica (SIESP) L'AQUILA	Istituto Zooprofilattico Sperimentale dell'Abruzzo e Molise "G. Caporale"	Lorusso A, Marcacci M, Di Domenico M, Ancora M, Curini V, Mangone I, Rinaldi A, Delli Compagni E, Di Pasquale A, Cammà C, Puglia I, Calistri P, Savini G
EPI_ISL_833088, EPI_ISL_833089	Servizio di Igiene Epidemiologia e Sanità Pubblica (SIESP) SULMONA	Istituto Zooprofilattico Sperimentale dell'Abruzzo e Molise "G. Caporale"	Lorusso A, Marcacci M, Di Domenico M, Ancora M, Curini V, Mangone I, Rinaldi A, Delli Compagni E, Di Pasquale A, Cammà C, Puglia I, Calistri P, Savini G
EPI_ISL_833090, EPI_ISL_833091, EPI_ISL_833092, EPI_ISL_833093, EPI_ISL_833094, EPI_ISL_833095, EPI_ISL_833096	SIESP CHIETI - DRIVE IN CHIETI	Istituto Zooprofilattico Sperimentale dell'Abruzzo e Molise "G. Caporale"	Lorusso A, Marcacci M, Di Domenico M, Ancora M, Curini V, Mangone I, Rinaldi A, Delli Compagni E, Di Pasquale A, Cammà C, Puglia I, Calistri P, Savini G
EPI_ISL_833097, EPI_ISL_833098	SIESP CHIETI - DRIVE IN LANCIANO	Istituto Zooprofilattico Sperimentale dell'Abruzzo e Molise "G. Caporale"	Lorusso A, Marcacci M, Di Domenico M, Ancora M, Curini V, Mangone I, Rinaldi A, Delli Compagni E, Di Pasquale A, Cammà C, Puglia I, Calistri P, Savini G
EPI_ISL_833099, EPI_ISL_833100	SIESP CHIETI - DRIVE IN ORTONA	Istituto Zooprofilattico Sperimentale dell'Abruzzo e Molise "G. Caporale"	Lorusso A, Marcacci M, Di Domenico M, Ancora M, Curini V, Mangone I, Rinaldi A, Delli Compagni E, Di Pasquale A, Cammà C, Puglia I, Calistri P, Savini G
EPI_ISL_833101, EPI_ISL_833102	SIESP DIPARTIMENTO DI PREVENZIONE CHIETI	Istituto Zooprofilattico Sperimentale dell'Abruzzo e Molise "G. Caporale"	Lorusso A, Marcacci M, Di Domenico M, Ancora M, Curini V, Mangone I, Rinaldi A, Delli Compagni E, Di Pasquale A, Cammà C, Puglia I, Calistri P, Savini G
EPI_ISL_833103, EPI_ISL_833104, EPI_ISL_833105, EPI_ISL_833106, EPI_ISL_833107, EPI_ISL_833108, EPI_ISL_833109, EPI_ISL_833110, EPI_ISL_833111, EPI_ISL_833112, EPI_ISL_833113, EPI_ISL_833114, EPI_ISL_833115, EPI_ISL_833116, EPI_ISL_833117, EPI_ISL_833118			
see above	SIESP DIPARTIMENTO DI PREVENZIONE TERAMO C.DA CASALENA	Istituto Zooprofilattico Sperimentale dell'Abruzzo e Molise "G. Caporale"	Lorusso A, Marcacci M, Di Domenico M, Ancora M, Curini V, Mangone I, Rinaldi A, Delli Compagni E, Di Pasquale A, Cammà C, Puglia I, Calistri P, Savini G
EPI_ISL_833119	SIESP L'AQUILA	Istituto Zooprofilattico Sperimentale dell'Abruzzo e Molise "G. Caporale"	Lorusso A, Marcacci M, Di Domenico M, Ancora M, Curini V, Mangone I, Rinaldi A, Delli Compagni E, Di Pasquale A, Cammà C, Puglia I, Calistri P, Savini G
EPI_ISL_833120, EPI_ISL_833121, EPI_ISL_833122	SIESP SULMONA	Istituto Zooprofilattico Sperimentale dell'Abruzzo e Molise "G. Caporale"	Lorusso A, Marcacci M, Di Domenico M, Ancora M, Curini V, Mangone I, Rinaldi A, Delli Compagni E, Di Pasquale A, Cammà C, Puglia I, Calistri P, Savini G
EPI_ISL_833123	Usca Pizzoli	Istituto Zooprofilattico Sperimentale dell'Abruzzo e Molise "G. Caporale"	Lorusso A, Marcacci M, Di Domenico M, Ancora M, Curini V, Mangone I, Rinaldi A, Delli Compagni E, Di Pasquale A, Cammà C, Puglia I, Calistri P, Savini G
EPI_ISL_833124, EPI_ISL_833125, EPI_ISL_833126, EPI_ISL_833127, EPI_ISL_833128	USCA Sulmona	Istituto Zooprofilattico Sperimentale dell'Abruzzo e Molise "G. Caporale"	Lorusso A, Marcacci M, Di Domenico M, Ancora M, Curini V, Mangone I, Rinaldi A, Delli Compagni E, Di Pasquale A, Cammà C, Puglia I, Calistri P, Savini G
EPI_ISL_833129	USCA Tagliacozzo	Istituto Zooprofilattico Sperimentale dell'Abruzzo e Molise "G. Caporale"	Lorusso A, Marcacci M, Di Domenico M, Ancora M, Curini V, Mangone I, Rinaldi A, Delli Compagni E, Di Pasquale A, Cammà C, Puglia I, Calistri P, Savini G
EPI_ISL_833228, EPI_ISL_833229	R.P. GUARDIAGRELE Ospedale di Comunità	Istitituto Zooprofilattico Sperimentale dell'Abruzzo e Molise "G.Caporale"	Lorusso A, Marcacci M, Di Domenico M, Curini V, Ancora M, Cammà C, Rinaldi A, Mangone I, Di Pasquale A, Puglia I, Calistri P, Savini G.
EPI_ISL_833230	OSPEDALE S.S. ANNUNZIATA CHIETI - CLINICA MEDICA (MEDICINA GENERALE 1)	Istitituto Zooprofilattico Sperimentale dell'Abruzzo e Molise "G.Caporale"	Lorusso A, Marcacci M, Di Domenico M, Curini V, Ancora M, Cammà C, Rinaldi A, Mangone I, Di Pasquale A, Puglia I, Calistri P, Savini G.
EPI_ISL_833231	SIESP CHIETI - DRIVE IN LANCIANO	Istitituto Zooprofilattico Sperimentale dell'Abruzzo e Molise "G.Caporale"	Lorusso A, Marcacci M, Di Domenico M, Curini V, Ancora M, Cammà C, Rinaldi A, Mangone I, Di Pasquale A, Puglia I, Calistri P, Savini G.
EPI_ISL_833232	SIESP CHIETI - DRIVE IN CHIETI	Istitituto Zooprofilattico Sperimentale dell'Abruzzo e Molise "G.Caporale"	Lorusso A, Marcacci M, Di Domenico M, Curini V, Ancora M, Cammà C, Rinaldi A, Mangone I, Di Pasquale A, Puglia I, Calistri P, Savini G.
EPI_ISL_833233, EPI_ISL_833234, EPI_ISL_833235, EPI_ISL_833236, EPI_ISL_833237, EPI_ISL_833238	SIESP DIPARTIMENTO DI PREVENZIONE CHIETI	Istitituto Zooprofilattico Sperimentale dell'Abruzzo e Molise "G.Caporale"	Lorusso A, Marcacci M, Di Domenico M, Curini V, Ancora M, Cammà C, Rinaldi A, Mangone I, Di Pasquale A, Puglia I, Calistri P, Savini G.
EPI_ISL_833239, EPI_ISL_833240	SIESP DIPARTIMENTO DI PREVENZIONE TERAMO	Istitituto Zooprofilattico Sperimentale dell'Abruzzo e Molise "G.Caporale"	Lorusso A, Marcacci M, Di Domenico M, Curini V, Ancora M, Cammà C, Rinaldi A, Mangone I, Di Pasquale A, Puglia I, Calistri P, Savini G.
EPI_ISL_833241	SIESP DIPARTIMENTO DI PREVENZIONE CHIETI	Istitituto Zooprofilattico Sperimentale dell'Abruzzo e Molise "G.Caporale"	Lorusso A, Marcacci M, Di Domenico M, Curini V, Ancora M, Cammà C, Rinaldi A, Mangone I, Di Pasquale A, Puglia I, Calistri P, Savini G.
EPI_ISL_833242	SIESP CHIETI - DRIVE IN LANCIANO	Istitituto Zooprofilattico Sperimentale dell'Abruzzo e Molise "G.Caporale"	Lorusso A, Marcacci M, Di Domenico M, Curini V, Ancora M, Cammà C, Rinaldi A, Mangone I, Di Pasquale A, Puglia I, Calistri P, Savini G.
EPI_ISL_833243, EPI_ISL_833244, EPI_ISL_833245, EPI_ISL_833246, EPI_ISL_833247	SIESP DIPARTIMENTO DI PREVENZIONE CHIETI	Istitituto Zooprofilattico Sperimentale dell'Abruzzo e Molise "G.Caporale"	Lorusso A, Marcacci M, Di Domenico M, Curini V, Ancora M, Cammà C, Rinaldi A, Mangone I, Di Pasquale A, Puglia I, Calistri P, Savini G.
EPI_ISL_833250	SIESP CHIETI - DRIVE IN ORTONA	Istituto Zooprofilattico Sperimentale dell'Abruzzo e Molise "G. Caporale"	Lorusso A, Marcacci M, Di Domenico M, Ancora M, Curini V, Mangone I, Rinaldi A, Di Pasquale A, Cammà C, Puglia I, Calistri P, Savini G
EPI_ISL_833251	Ospedale Civile Atri-Medicina Interna	Istituto Zooprofilattico Sperimentale dell'Abruzzo e Molise "G. Caporale"	Lorusso A, Marcacci M, Di Domenico M, Ancora M, Curini V, Mangone I, Rinaldi A, Di Pasquale A, Cammà C, Puglia I, Calistri P, Savini G
EPI_ISL_833252	Ospedale Civile Giulianova-Pronto Soccorso	Istituto Zooprofilattico Sperimentale dell'Abruzzo e Molise "G. Caporale"	Lorusso A, Marcacci M, Di Domenico M, Ancora M, Curini V, Mangone I, Rinaldi A, Di Pasquale A, Cammà C, Puglia I, Calistri P, Savini G
EPI_ISL_833253	USCA-Avezzano	Istituto Zooprofilattico Sperimentale dell'Abruzzo e Molise "G. Caporale"	Lorusso A, Marcacci M, Di Domenico M, Ancora M, Curini V, Mangone I, Rinaldi A, Di Pasquale A, Cammà C, Puglia I, Calistri P, Savini G
EPI_ISL_833254	USCA-Pescina	Istituto Zooprofilattico Sperimentale dell'Abruzzo e Molise "G. Caporale"	Lorusso A, Marcacci M, Di Domenico M, Ancora M, Curini V, Mangone I, Rinaldi A, Di Pasquale A, Cammà C, Puglia I, Calistri P, Savini G
EPI_ISL_833255, EPI_ISL_833256, EPI_ISL_833257	Ospedale Civile Atri	Istituto Zooprofilattico Sperimentale dell'Abruzzo e Molise "G. Caporale"	Lorusso A, Marcacci M, Di Domenico M, Ancora M, Curini V, Mangone I, Rinaldi A, Di Pasquale A, Cammà C, Puglia I, Calistri P, Savini G
EPI_ISL_833258, EPI_ISL_833259	SIESP DIPARTIMENTO DI PREVENZIONE TERAMO	Istituto Zooprofilattico Sperimentale dell'Abruzzo e Molise "G. Caporale"	Lorusso A, Marcacci M, Di Domenico M, Ancora M, Curini V, Mangone I, Rinaldi A, Di Pasquale A, Cammà C, Puglia I, Calistri P, Savini G
EPI_ISL_833260, EPI_ISL_833261	Giulianova	Istituto Zooprofilattico Sperimentale dell'Abruzzo e Molise "G. Caporale"	Lorusso A, Marcacci M, Di Domenico M, Ancora M, Curini V, Mangone I, Rinaldi A, Di Pasquale A, Cammà C, Puglia I, Calistri P, Savini G
EPI_ISL_833262, EPI_ISL_833263, EPI_ISL_833264	SIESP DIPARTIMENTO DI PREVENZIONE CHIETI	Istituto Zooprofilattico Sperimentale dell'Abruzzo e Molise "G. Caporale"	Lorusso A, Marcacci M, Di Domenico M, Ancora M, Curini V, Mangone I, Rinaldi A, Di Pasquale A, Cammà C, Puglia I, Calistri P, Savini G
EPI_ISL_833265, EPI_ISL_833266, EPI_ISL_833267, EPI_ISL_833268, EPI_ISL_833269	Ospedale SS Annunziata Chieti	Istituto Zooprofilattico Sperimentale dell'Abruzzo e Molise "G. Caporale"	Lorusso A, Marcacci M, Di Domenico M, Ancora M, Curini V, Mangone I, Rinaldi A, Di Pasquale A, Cammà C, Puglia I, Calistri P, Savini G
EPI_ISL_833270	SIESP CHIETI - DRIVE IN ORTONA	Istituto Zooprofilattico Sperimentale dell'Abruzzo e Molise "G.	Lorusso A, Marcacci M, Di Domenico M, Ancora M, Curini V, Mangone I, Rinaldi A, Di Pasquale A, Cammà C, Puglia I, Calistri P, Savini G

		Caporale"	
EPI_ISL_833271	SIESP CHIETI - DRIVE IN GISSI	Istituto Zooprofilattico Sperimentale dell'Abruzzo e Molise "G. Caporale"	Lorusso A, Marcacci M, Di Domenico M, Ancora M, Curini V, Mangone I, Rinaldi A, Di Pasquale A, Cammà C, Puglia I, Calistri P, Savini G
EPI_ISL_833272, EPI_ISL_833273, EPI_ISL_833274, EPI_ISL_833275	SIESP CHIETI - DRIVE IN ORTONA	Istituto Zooprofilattico Sperimentale dell'Abruzzo e Molise "G. Caporale"	Lorusso A, Marcacci M, Di Domenico M, Ancora M, Curini V, Mangone I, Rinaldi A, Di Pasquale A, Cammà C, Puglia I, Calistri P, Savini G
EPI_ISL_833276	Ospedale Civile G.Mazzini-Teramo	Istituto Zooprofilattico Sperimentale dell'Abruzzo e Molise "G. Caporale"	Lorusso A, Marcacci M, Di Domenico M, Ancora M, Curini V, Mangone I, Rinaldi A, Di Pasquale A, Cammà C, Puglia I, Calistri P, Savini G
EPI_ISL_833277	USCA-Sulmona	Istituto Zooprofilattico Sperimentale dell'Abruzzo e Molise "G. Caporale"	Lorusso A, Marcacci M, Di Domenico M, Ancora M, Curini V, Mangone I, Rinaldi A, Di Pasquale A, Cammà C, Puglia I, Calistri P, Savini G
EPI_ISL_833278, EPI_ISL_833279, EPI_ISL_833280	SIESP CHIETI- DRIVE IN CHIETI	Istituto Zooprofilattico Sperimentale dell'Abruzzo e Molise "G. Caporale"	Lorusso A, Marcacci M, Di Domenico M, Ancora M, Curini V, Mangone I, Rinaldi A, Di Pasquale A, Cammà C, Puglia I, Calistri P, Savini G
EPI_ISL_833281, EPI_ISL_833282	SIESP DIPARTIMENTO DI PREVENZIONE TERAMO	Istituto Zooprofilattico Sperimentale dell'Abruzzo e Molise "G. Caporale"	Lorusso A, Marcacci M, Di Domenico M, Ancora M, Curini V, Mangone I, Rinaldi A, Di Pasquale A, Cammà C, Puglia I, Calistri P, Savini G
EPI_ISL_833283	DIPARTIMENTO PREVENZIONE AVEZZANO-SERVIZIO DI IGIENE EPIDEMIOLOGIA E SANITA' PUBBLICA	Istituto Zooprofilattico Sperimentale dell'Abruzzo e Molise "G. Caporale"	Lorusso A, Marcacci M, Di Domenico M, Ancora M, Curini V, Mangone I, Rinaldi A, Di Pasquale A, Cammà C, Puglia I, Calistri P, Savini G
EPI_ISL_833284, EPI_ISL_833285, EPI_ISL_833286, EPI_ISL_833287	SIESP CHIETI - DRIVE IN ORTONA	Istituto Zooprofilattico Sperimentale dell'Abruzzo e Molise "G. Caporale"	Lorusso A, Marcacci M, Di Domenico M, Ancora M, Curini V, Mangone I, Rinaldi A, Di Pasquale A, Cammà C, Puglia I, Calistri P, Savini G
EPI_ISL_833288	PRESIDIO TERRITORIALE CASOLI CH	Istituto Zooprofilattico Sperimentale dell'Abruzzo e Molise "G. Caporale"	Lorusso A, Marcacci M, Di Domenico M, Ancora M, Curini V, Mangone I, Rinaldi A, Di Pasquale A, Cammà C, Puglia I, Calistri P, Savini G
EPI_ISL_833289	Ospedale SS Annunziata Chieti	Istituto Zooprofilattico Sperimentale dell'Abruzzo e Molise "G. Caporale"	Lorusso A, Marcacci M, Di Domenico M, Ancora M, Curini V, Mangone I, Rinaldi A, Di Pasquale A, Cammà C, Puglia I, Calistri P, Savini G
EPI_ISL_833290	PRESIDIO TERRITORIALE CASOLI CH	Istituto Zooprofilattico Sperimentale dell'Abruzzo e Molise "G. Caporale"	Lorusso A, Marcacci M, Di Domenico M, Ancora M, Curini V, Mangone I, Rinaldi A, Di Pasquale A, Cammà C, Puglia I, Calistri P, Savini G
EPI_ISL_833291	SIESP DIPARTIMENTO DI PREVENZIONE DELL'AQUILA	Istituto Zooprofilattico Sperimentale dell'Abruzzo e Molise "G. Caporale"	Lorusso A, Marcacci M, Di Domenico M, Ancora M, Curini V, Mangone I, Rinaldi A, Di Pasquale A, Cammà C, Puglia I, Calistri P, Savini G
EPI_ISL_833292, EPI_ISL_833293, EPI_ISL_833294	SIESP CHIETI - DRIVE IN LANCIANO	Istituto Zooprofilattico Sperimentale dell'Abruzzo e Molise "G. Caporale"	Lorusso A, Marcacci M, Di Domenico M, Ancora M, Curini V, Mangone I, Rinaldi A, Di Pasquale A, Cammà C, Puglia I, Calistri P, Savini G
EPI_ISL_833295, EPI_ISL_833296	SIESP DIPARTIMENTO DI PREVENZIONE TERAMO	Istituto Zooprofilattico Sperimentale dell'Abruzzo e Molise "G. Caporale"	Lorusso A, Marcacci M, Di Domenico M, Ancora M, Curini V, Mangone I, Rinaldi A, Di Pasquale A, Cammà C, Puglia I, Calistri P, Savini G
EPI_ISL_833297	Ospedale Civile Giulianova	Istituto Zooprofilattico Sperimentale dell'Abruzzo e Molise "G. Caporale"	Lorusso A, Marcacci M, Di Domenico M, Ancora M, Curini V, Mangone I, Rinaldi A, Di Pasquale A, Cammà C, Puglia I, Calistri P, Savini G
EPI_ISL_833298	SIESP CHIETI - DRIVE IN LANCIANO	Istituto Zooprofilattico Sperimentale dell'Abruzzo e Molise "G. Caporale"	Lorusso A, Marcacci M, Di Domenico M, Ancora M, Curini V, Mangone I, Rinaldi A, Di Pasquale A, Cammà C, Puglia I, Calistri P, Savini G
EPI_ISL_833299	SIESP DIPARTIMENTO DI PREVENZIONE CHIETI	Istituto Zooprofilattico Sperimentale dell'Abruzzo e Molise "G. Caporale"	Lorusso A, Marcacci M, Di Domenico M, Ancora M, Curini V, Mangone I, Rinaldi A, Di Pasquale A, Cammà C, Puglia I, Calistri P, Savini G
EPI_ISL_833300	SIESP CHIETI - DRIVE IN LANCIANO	Istituto Zooprofilattico Sperimentale dell'Abruzzo e Molise "G. Caporale"	Lorusso A, Marcacci M, Di Domenico M, Ancora M, Curini V, Mangone I, Rinaldi A, Di Pasquale A, Cammà C, Puglia I, Calistri P, Savini G
EPI_ISL_833301	DIPARTIMENTO PREVENZIONE AVEZZANO-SERVIZIO DI IGIENE EPIDEMIOLOGIA E SANITA' PUBBLICA	Istituto Zooprofilattico Sperimentale dell'Abruzzo e Molise "G. Caporale"	Lorusso A, Marcacci M, Di Domenico M, Ancora M, Curini V, Mangone I, Rinaldi A, Di Pasquale A, Cammà C, Puglia I, Calistri P, Savini G
EPI_ISL_833302, EPI_ISL_833303, EPI_ISL_833304, EPI_ISL_833305	SIESP DIPARTIMENTO DI PREVENZIONE TERAMO	Istituto Zooprofilattico Sperimentale dell'Abruzzo e Molise "G. Caporale"	Lorusso A, Marcacci M, Di Domenico M, Ancora M, Curini V, Mangone I, Rinaldi A, Di Pasquale A, Cammà C, Puglia I, Calistri P, Savini G
EPI_ISL_833306, EPI_ISL_833307, EPI_ISL_833308, EPI_ISL_833309, EPI_ISL_833310	SIESP CHIETI - DRIVE IN ORTONA	Istituto Zooprofilattico Sperimentale dell'Abruzzo e Molise "G. Caporale"	Lorusso A, Marcacci M, Di Domenico M, Ancora M, Curini V, Mangone I, Rinaldi A, Di Pasquale A, Cammà C, Puglia I, Calistri P, Savini G
EPI_ISL_833311, EPI_ISL_833312	SIESP CHIETI- DRIVE IN CHIETI	Istituto Zooprofilattico Sperimentale dell'Abruzzo e Molise "G. Caporale"	Lorusso A, Marcacci M, Di Domenico M, Ancora M, Curini V, Mangone I, Rinaldi A, Di Pasquale A, Cammà C, Puglia I, Calistri P, Savini G
EPI_ISL_833313, EPI_ISL_833314, EPI_ISL_833315	SIESP CHIETI - DRIVE IN LANCIANO	Istituto Zooprofilattico Sperimentale dell'Abruzzo e Molise "G. Caporale"	Lorusso A, Marcacci M, Di Domenico M, Ancora M, Curini V, Mangone I, Rinaldi A, Di Pasquale A, Cammà C, Puglia I, Calistri P, Savini G
EPI_ISL_833316	SIESP CHIETI - DRIVE IN GISSI	Istituto Zooprofilattico Sperimentale dell'Abruzzo e Molise "G. Caporale"	Lorusso A, Marcacci M, Di Domenico M, Ancora M, Curini V, Mangone I, Rinaldi A, Di Pasquale A, Cammà C, Puglia I, Calistri P, Savini G
EPI_ISL_833317, EPI_ISL_833318	SIESP CHIETI - DRIVE IN CHIETI	Istituto Zooprofilattico Sperimentale dell'Abruzzo e Molise "G. Caporale"	Lorusso A, Marcacci M, Di Domenico M, Ancora M, Curini V, Mangone I, Rinaldi A, Di Pasquale A, Cammà C, Puglia I, Calistri P, Savini G
EPI_ISL_833319, EPI_ISL_833320	SIESP DIPARTIMENTO DI PREVENZIONE SULMONA	Istituto Zooprofilattico Sperimentale dell'Abruzzo e Molise "G. Caporale"	Lorusso A, Marcacci M, Di Domenico M, Ancora M, Curini V, Mangone I, Rinaldi A, Di Pasquale A, Cammà C, Puglia I, Calistri P, Savini G
EPI_ISL_833321	SIESP DIPARTIMENTO DI PREVENZIONE CHIETI	Istituto Zooprofilattico Sperimentale dell'Abruzzo e Molise "G. Caporale"	Lorusso A, Marcacci M, Di Domenico M, Ancora M, Curini V, Mangone I, Rinaldi A, Di Pasquale A, Cammà C, Puglia I, Calistri P, Savini G
EPI_ISL_833322	SIESP DIPARTIMENTO DI PREVENZIONE SULMONA	Istituto Zooprofilattico Sperimentale dell'Abruzzo e Molise "G. Caporale"	Lorusso A, Marcacci M, Di Domenico M, Ancora M, Curini V, Mangone I, Rinaldi A, Di Pasquale A, Cammà C, Puglia I, Calistri P, Savini G
EPI_ISL_833323	SIESP CHIETI - DRIVE IN CHIETI	Istituto Zooprofilattico Sperimentale dell'Abruzzo e Molise "G. Caporale"	Lorusso A, Marcacci M, Di Domenico M, Ancora M, Curini V, Mangone I, Rinaldi A, Di Pasquale A, Cammà C, Puglia I, Calistri P, Savini G
EPI_ISL_833324	SIESP DIPARTIMENTO DI PREVENZIONE CHIETI	Istituto Zooprofilattico Sperimentale dell'Abruzzo e Molise "G. Caporale"	Lorusso A, Marcacci M, Di Domenico M, Ancora M, Curini V, Mangone I, Rinaldi A, Di Pasquale A, Cammà C, Puglia I, Calistri P, Savini G
EPI_ISL_833325	Ospedale SS Annunziata Chieti	Istituto Zooprofilattico Sperimentale dell'Abruzzo e Molise "G. Caporale"	Lorusso A, Marcacci M, Di Domenico M, Ancora M, Curini V, Mangone I, Rinaldi A, Di Pasquale A, Cammà C, Puglia I, Calistri P, Savini G
EPI_ISL_833326	SIESP DIPARTIMENTO DI PREVENZIONE CHIETI	Istituto Zooprofilattico Sperimentale dell'Abruzzo e Molise "G. Caporale"	Lorusso A, Marcacci M, Di Domenico M, Ancora M, Curini V, Mangone I, Rinaldi A, Di Pasquale A, Cammà C, Puglia I, Calistri P, Savini G
EPI_ISL_833327	Ospedale SS Annunziata Chieti	Istituto Zooprofilattico Sperimentale dell'Abruzzo e Molise "G. Caporale"	Lorusso A, Marcacci M, Di Domenico M, Ancora M, Curini V, Mangone I, Rinaldi A, Di Pasquale A, Cammà C, Puglia I, Calistri P, Savini G
EPI_ISL_833328, EPI_ISL_833329, EPI_ISL_833330	SIESP CHIETI - DRIVE IN ORTONA	Istituto Zooprofilattico Sperimentale dell'Abruzzo e Molise "G. Caporale"	Lorusso A, Marcacci M, Di Domenico M, Ancora M, Curini V, Mangone I, Rinaldi A, Di Pasquale A, Cammà C, Puglia I, Calistri P, Savini G

EPI_ISL_833331	Ospedale Civile Atri	Istituto Zooprofilattico Sperimentale dell'Abruzzo e Molise "G. Caporale"	Lorusso A, Marcacci M, Di Domenico M, Ancora M, Curini V, Mangone I, Rinaldi A, Di Pasquale A, Cammà C, Puglia I, Calistri P, Savini G
EPI_ISL_833435, EPI_ISL_833436, EPI_ISL_833437, EPI_ISL_833438, EPI_ISL_833439, EPI_ISL_833440, EPI_ISL_833441, EPI_ISL_833442, EPI_ISL_833443, EPI_ISL_833444, EPI_ISL_833445, EPI_ISL_833446, EPI_ISL_833447, EPI_ISL_833448, EPI_ISL_833449, EPI_ISL_833450, EPI_ISL_833451, EPI_ISL_833452, EPI_ISL_833453, EPI_ISL_833454, EPI_ISL_833455			
see above	SC (UCO) Igiene e Sanità Pubblica (funzione integrata con SC Microbiologia e Virologia) e Laboratory of Molecular Virology of the International Centre for Genetic Engineering and Biotechnology (ICGEB)	ARGO Laboratorio Genomica ed Epigenomica	Licastro D, Dal Monego S, Degasperi M, Marcello A, D'Agaro P
EPI_ISL_833522, EPI_ISL_833523, EPI_ISL_833524, EPI_ISL_833525, EPI_ISL_833526, EPI_ISL_833527, EPI_ISL_833528, EPI_ISL_833529, EPI_ISL_833530, EPI_ISL_833531, EPI_ISL_833532, EPI_ISL_833533, EPI_ISL_833534, EPI_ISL_833535, EPI_ISL_833536, EPI_ISL_833537, EPI_ISL_833538, EPI_ISL_833539, EPI_ISL_833540, EPI_ISL_833541, EPI_ISL_833542, EPI_ISL_833543, EPI_ISL_833544, EPI_ISL_833545, EPI_ISL_833546, EPI_ISL_833547, EPI_ISL_833548, EPI_ISL_833549, EPI_ISL_833550, EPI_ISL_833551, EPI_ISL_833552, EPI_ISL_833553, EPI_ISL_833554, EPI_ISL_833555, EPI_ISL_833556, EPI_ISL_833557, EPI_ISL_833558, EPI_ISL_833559, EPI_ISL_833560, EPI_ISL_833561, EPI_ISL_833562, EPI_ISL_833563, EPI_ISL_833564, EPI_ISL_833565, EPI_ISL_833566, EPI_ISL_833567, EPI_ISL_833568, EPI_ISL_833569, EPI_ISL_833570, EPI_ISL_837248, EPI_ISL_837249, EPI_ISL_837250, EPI_ISL_837251, EPI_ISL_837252, EPI_ISL_837253, EPI_ISL_837254, EPI_ISL_837255, EPI_ISL_837256, EPI_ISL_837257, EPI_ISL_837258, EPI_ISL_837259, EPI_ISL_837260, EPI_ISL_837261, EPI_ISL_837262, EPI_ISL_837263, EPI_ISL_837264, EPI_ISL_837265, EPI_ISL_837266, EPI_ISL_837267, EPI_ISL_837268, EPI_ISL_837269, EPI_ISL_837270, EPI_ISL_837271, EPI_ISL_837272, EPI_ISL_837273, EPI_ISL_837274, EPI_ISL_837275, EPI_ISL_837276, EPI_ISL_837277, EPI_ISL_837278, EPI_ISL_837279, EPI_ISL_837280, EPI_ISL_837281, EPI_ISL_837282, EPI_ISL_837283, EPI_ISL_837284, EPI_ISL_837285, EPI_ISL_837286, EPI_ISL_837287, EPI_ISL_837288, EPI_ISL_837289, EPI_ISL_837290, EPI_ISL_837291, EPI_ISL_837292, EPI_ISL_837293, EPI_ISL_837294, EPI_ISL_837295, EPI_ISL_837296, EPI_ISL_837297, EPI_ISL_837298, EPI_ISL_837299, EPI_ISL_837300, EPI_ISL_837301, EPI_ISL_837302, EPI_ISL_837303, EPI_ISL_837304, EPI_ISL_837305, EPI_ISL_837306, EPI_ISL_837307, EPI_ISL_837308, EPI_ISL_837309, EPI_ISL_837310, EPI_ISL_837311, EPI_ISL_837312, EPI_ISL_837313, EPI_ISL_837314, EPI_ISL_837315, EPI_ISL_837316, EPI_ISL_837317, EPI_ISL_837318, EPI_ISL_837319, EPI_ISL_837320, EPI_ISL_837321, EPI_ISL_837322, EPI_ISL_837323, EPI_ISL_837324, EPI_ISL_837325, EPI_ISL_837326, EPI_ISL_837327, EPI_ISL_837328, EPI_ISL_837329, EPI_ISL_837330, EPI_ISL_837433, EPI_ISL_837434, EPI_ISL_837435, EPI_ISL_837436, EPI_ISL_837437, EPI_ISL_837438, EPI_ISL_837439, EPI_ISL_837440, EPI_ISL_837441, EPI_ISL_837442, EPI_ISL_837443, EPI_ISL_837444, EPI_ISL_837445, EPI_ISL_837446, EPI_ISL_837447, EPI_ISL_837448, EPI_ISL_837449, EPI_ISL_837450, EPI_ISL_837451, EPI_ISL_837452, EPI_ISL_837453, EPI_ISL_837454, EPI_ISL_837455, EPI_ISL_837456, EPI_ISL_837457, EPI_ISL_837458, EPI_ISL_837459, EPI_ISL_837460, EPI_ISL_837461, EPI_ISL_837462, EPI_ISL_837463, EPI_ISL_837464, EPI_ISL_837465, EPI_ISL_837466, EPI_ISL_837467, EPI_ISL_837468, EPI_ISL_837469, EPI_ISL_837470, EPI_ISL_837471, EPI_ISL_837472, EPI_ISL_837473, EPI_ISL_837474, EPI_ISL_837475, EPI_ISL_837476, EPI_ISL_837477, EPI_ISL_837478, EPI_ISL_837479, EPI_ISL_837480, EPI_ISL_837481, EPI_ISL_837482, EPI_ISL_837483, EPI_ISL_837484, EPI_ISL_837485, EPI_ISL_837486, EPI_ISL_837487, EPI_ISL_837488, EPI_ISL_837489, EPI_ISL_837490, EPI_ISL_837491, EPI_ISL_837492, EPI_ISL_837493, EPI_ISL_837494, EPI_ISL_837495, EPI_ISL_837496, EPI_ISL_837497, EPI_ISL_837498, EPI_ISL_837499, EPI_ISL_837500, EPI_ISL_837501, EPI_ISL_837502, EPI_ISL_837503, EPI_ISL_837504			
see above	Istituto Zooprofilattico Sperimentale del Mezzogiorno	TIGEM	Antonio Grimaldi, Patrizia Annunziata, Francesco Panariello, Biancamaria Pierri, Valentina Bouche, Chiara Colantuono, Maria Concetta Cuomo, Denise Di Concilio, Lucio Di Filippo, Anna Manfredi, Marcello Salvi, Antonio Limone, Pellegrino Cerino, Andrea Ballabio, Davide Cacchiarelli.
EPI_ISL_847825	Lab. Microbiologia e Virologia, Cotugno, A.O. dei Colli	Lab. Microbiologia e Virologia, Cotugno, A.O. dei Colli	Luigi Atripaldi, Claudia Tiberio, Anna Perfetti
EPI_ISL_849634	SIESP DIPARTIMENTO DI PREVENZIONE CHIETI	Istitituto Zooprofilattico Sperimentale dell'Abruzzo e Molise "G.Caporale"	Lorusso A, Marcacci M, Di Domenico M, Curini V, Ancora M, Cammà C, Rinaldi A, Mangone I, Di Pasquale A, Puglia I, Savini G.
EPI_ISL_849635, EPI_ISL_849636	DIPARTIMENTO PREVENZIONE AVEZZANO-SERVIZIO DI IGIENE EPIDEMIOLOGIA E SANITA' PUBBLICA	Istitituto Zooprofilattico Sperimentale dell'Abruzzo e Molise "G.Caporale"	Lorusso A, Marcacci M, Di Domenico M, Curini V, Ancora M, Cammà C, Rinaldi A, Mangone I, Di Pasquale A, Puglia I, Savini G.
EPI_ISL_849637, EPI_ISL_849638, EPI_ISL_849639, EPI_ISL_849640, EPI_ISL_849641, EPI_ISL_849642, EPI_ISL_849643, EPI_ISL_849644, EPI_ISL_849645, EPI_ISL_849646	SIESP DIPARTIMENTO DI PREVENZIONE CHIETI	Istitituto Zooprofilattico Sperimentale dell'Abruzzo e Molise "G.Caporale"	Lorusso A, Marcacci M, Di Domenico M, Curini V, Ancora M, Cammà C, Rinaldi A, Mangone I, Di Pasquale A, Puglia I, Savini G.
EPI_ISL_849647, EPI_ISL_849648, EPI_ISL_849649	SIESP- DRIVE IN CHIETI	Istitituto Zooprofilattico Sperimentale dell'Abruzzo e Molise "G.Caporale"	Lorusso A, Marcacci M, Di Domenico M, Curini V, Ancora M, Cammà C, Rinaldi A, Mangone I, Di Pasquale A, Puglia I, Savini G.
EPI_ISL_849650, EPI_ISL_849651, EPI_ISL_849652	Servizio di igiene epidemiologia e sanità pubblica (SIESP)-Chieti	Istitituto Zooprofilattico Sperimentale dell'Abruzzo e Molise "G.Caporale"	Lorusso A, Marcacci M, Di Domenico M, Curini V, Ancora M, Cammà C, Rinaldi A, Mangone I, Di Pasquale A, Puglia I, Savini G.
EPI_ISL_849653	Servizio di igiene e sanità pubblica (SIESP)-Teramo	Istitituto Zooprofilattico Sperimentale dell'Abruzzo e Molise "G.Caporale"	Lorusso A, Marcacci M, Di Domenico M, Curini V, Ancora M, Cammà C, Rinaldi A, Mangone I, Di Pasquale A, Puglia I, Savini G.
EPI_ISL_849654	Servizio Igiene Epidemiologia e Sanità Pubblica (SIESP)-L'Aquila	Istitituto Zooprofilattico Sperimentale dell'Abruzzo e Molise "G.Caporale"	Lorusso A, Marcacci M, Di Domenico M, Curini V, Ancora M, Cammà C, Rinaldi A, Mangone I, Di Pasquale A, Puglia I, Savini G.
EPI_ISL_849655	Servizio di igiene e sanità pubblica (SIESP)-Teramo	Istitituto Zooprofilattico Sperimentale dell'Abruzzo e Molise "G.Caporale"	Lorusso A, Marcacci M, Di Domenico M, Curini V, Ancora M, Cammà C, Rinaldi A, Mangone I, Di Pasquale A, Puglia I, Savini G.
EPI_ISL_849656, EPI_ISL_849657, EPI_ISL_849658, EPI_ISL_849659, EPI_ISL_849660, EPI_ISL_849661	Servizio Igiene Epidemiologia e Sanità Pubblica (SIESP)-L'Aquila	Istitituto Zooprofilattico Sperimentale dell'Abruzzo e Molise "G.Caporale"	Lorusso A, Marcacci M, Di Domenico M, Curini V, Ancora M, Cammà C, Rinaldi A, Mangone I, Di Pasquale A, Puglia I, Savini G.
EPI_ISL_849662, EPI_ISL_849663	Servizio di igiene epidemiologia e sanità pubblica (SIESP)-Chieti	Istitituto Zooprofilattico Sperimentale dell'Abruzzo e Molise "G.Caporale"	Lorusso A, Marcacci M, Di Domenico M, Curini V, Ancora M, Cammà C, Rinaldi A, Mangone I, Di Pasquale A, Puglia I, Savini G.
EPI_ISL_849763	Molecular Biology, IZS Sicilia	Molecular Biology, IZS Sicilia	Reale,S., Seidita,G., Di Gaudio,F., Scibetta,S. and Vitale,F.
EPI_ISL_852619, EPI_ISL_852622, EPI_ISL_852628, EPI_ISL_852629, EPI_ISL_852630, EPI_ISL_852631, EPI_ISL_852632, EPI_ISL_852813, EPI_ISL_852819, EPI_ISL_852821, EPI_ISL_852823, EPI_ISL_852825, EPI_ISL_852828			
see above	Lab. Microbiologia e Virologia, Cotugno, A.O. dei Colli	Lab. Microbiologia e Virologia, Cotugno, A.O. dei Colli	Luigi Atripaldi, Claudia Tiberio, Anna Perfetti
EPI_ISL_854303	Pharmgenetix GmbH	Bergthaler laboratory, CeMM Research Center for Molecular Medicine of the Austrian Academy of Sciences	Lukas Endler, Alexandra Popa, Benedikt Agerer, Jakob-Wendelin Genger, Alexander Lercher, Anna Schedl, Thomas Penz, Michael Schuster, Jan Laine, Martin Senekowitsch, Christoph Bock, Andreas Bergthaler
EPI_ISL_855550	Department of Infectious Diseases, Istituto Superiore di Sanità, Rome, Italy	Istituto Superiore di Sanità (ISS)	Paola Stefanelli, Angela Di Martino, Alessandra Lo Presti, Stefano Fiore, Gabriele Vaccari, Luca De Sabato, Ilaria Di Bartolo, Giovanni Ianiro, Manuela Marra, Maria Carollo, Marco Crescenzi
EPI_ISL_855552	Department of Infectious Diseases, Istituto Superiore di Sanità, Rome, Italy; Ospedali Riuniti, Laboratorio Virologia, Ancona, Italy	Istituto Superiore di Sanità (ISS)	Paola Stefanelli, Angela Di Martino, Alessandra Lo Presti, Stefano Fiore, Patrizia Bagnarelli, Gabriele Vaccari, Luca De Sabato, Ilaria Di Bartolo, Giovanni Ianiro, Manuela Marra, Maria Carollo, Marco Crescenzi
EPI_ISL_855553	Department of Infectious Diseases, Istituto Superiore di Sanità, Rome, Italy	Istituto Superiore di Sanità (ISS)	Paola Stefanelli, Angela Di Martino, Alessandra Lo Presti, Stefano Fiore, Gabriele Vaccari, Luca De Sabato, Ilaria Di Bartolo, Giovanni Ianiro, Manuela Marra, Maria Carollo, Marco Crescenzi
EPI_ISL_855554, EPI_ISL_855555	Department of Infectious Diseases, Istituto Superiore di Sanità, Rome, Italy; ASST G.O.M. Niguarda, Sezione Microbiologia e Virologia, Milano, Italy	Istituto Superiore di Sanità (ISS)	Paola Stefanelli, Angela Di Martino, Alessandra Lo Presti, Stefano Fiore, Carlo Federico Perno, Gabriele Vaccari, Luca De Sabato, Ilaria Di Bartolo, Giovanni Ianiro, Manuela Marra, Maria Carollo, Marco Crescenzi
EPI_ISL_855556, EPI_ISL_855573, EPI_ISL_855610, EPI_ISL_855611, EPI_ISL_855612, EPI_ISL_855613, EPI_ISL_855614, EPI_ISL_856679, EPI_ISL_856682, EPI_ISL_856694, EPI_ISL_856698			
see above	Lab. Microbiologia e Virologia, Cotugno, A.O. dei Colli	Lab. Microbiologia e Virologia, Cotugno, A.O. dei Colli	Luigi Atripaldi, Claudia Tiberio, Anna Perfetti
EPI_ISL_856712, EPI_ISL_856713, EPI_ISL_856714, EPI_ISL_856715, EPI_ISL_856716, EPI_ISL_856717, EPI_ISL_856718, EPI_ISL_856719, EPI_ISL_856720, EPI_ISL_856721	Infectious Diseases Unit, Department of Internal Medicine, Azienda Ospedaliera-Universitaria di Padova	Laboratory of Infectious Diseases, Department of Biomedical and Clinical Sciences L. Sacco, University of Milan	Anna Maria Cattelan, Lolita Sasset, Davide Leoni, Alessia Lai, Annalisa Bergna, Carla Della Ventura, Claudia Balotta, Massimo Galli, Gianguglielmo Zehender on behalf of SARS-CoV-2 ITALIAN RESEARCH ENTERPRISE-(SCIRE) Collaborative Group
EPI_ISL_856869	Virology Laboratory, Scientific Department, Army Medical Center	Virology Laboratory, Scientific Department, Army Medical Center	Silvia Fillo, Giovanni Faggioni, Riccardo De Sanctis, Antonella Fortunato, Anella Monte, Anna Anselmo, Vanessa Vera Fain, Francesco Giordani, Nino D'Amore, Filippo Molinari, Giancarlo Petralito, Florigio Lista
EPI_ISL_856870	Virology Laboratory, Scientific Department, Army Medical Center	Virology Laboratory, Scientific Department, Army Medical Center	Silvia Fillo, Giovanni Faggioni, Riccardo De Sanctis, Antonella Fortunato, Anella Monte, Anna Anselmo, Vanessa Vera Fain, Francesco Giordani, Nino D'Amore, Filippo Molinari, Giancarlo Petralito, Florigio Lista

EPI_ISL_856871	Department of Infectious Diseases, Istituto Superiore di Sanità, Roma, Italy; AORN dei Colli, UOC Microbiologia Virologia, Napoli, Italy	Virology Laboratory, Scientific Department, Army Medical Center	Paola Stefanelli, Angela Di Martino, Alessandra Lo Presti, Stefano Fiore, Luigi Atripaldi, Silvia Fillo, Giovanni Faggioni, Riccardo De Sanctis, Antonella Fortunato, Anna Anselmo, Francesco Giordani, Vanessa Vera Fain, Nino D'Amore, Florigio Lista
EPI_ISL_856872, EPI_ISL_856873, EPI_ISL_856874, EPI_ISL_856875	Virology Laboratory, Scientific Department, Army Medical Center	Virology Laboratory, Scientific Department, Army Medical Center	Silvia Fillo, Giovanni Faggioni, Riccardo De Sanctis, Antonella Fortunato, Anella Monte, Anna Anselmo, Vanessa Vera Fain, Francesco Giordani, Nino D'Amore, Filippo Molinari, Giancarlo Petralito, Florigio Lista
EPI_ISL_856876	Virology Laboratory, Scientific Department, Army Medical Center	Virology Laboratory, Scientific Department, Army Medical Center	Silvia Fillo, Giovanni Faggioni, Riccardo De Sanctis, Antonella Fortunato, Anella Monte, Anna Anselmo, Vanessa Vera Fain, Francesco Giordani, Nino D'Amore, Filippo Molinari, Giancarlo Petralito, Florigio Lista
EPI_ISL_856877, EPI_ISL_856878	Virology Laboratory, Scientific Department, Army Medical Center	Virology Laboratory, Scientific Department, Army Medical Center	Silvia Fillo, Giovanni Faggioni, Riccardo De Sanctis, Antonella Fortunato, Anella Monte, Anna Anselmo, Vanessa Vera Fain, Francesco Giordani, Nino D'Amore, Filippo Molinari, Giancarlo Petralito, Florigio Lista
EPI_ISL_856879	Virology Laboratory, Scientific Department, Army Medical Center	Virology Laboratory, Scientific Department, Army Medical Center	Silvia Fillo, Giovanni Faggioni, Riccardo De Sanctis, Antonella Fortunato, Anella Monte, Anna Anselmo, Vanessa Vera Fain, Francesco Giordani, Nino D'Amore, Filippo Molinari, Giancarlo Petralito, Florigio Lista
EPI_ISL_856880	Department of Infectious Diseases, Istituto Superiore di Sanità, Roma, Italy; Ospedali Riuniti, Laboratorio Virologia, Ancona, Italy	Virology Laboratory, Scientific Department, Army Medical Center	Paola Stefanelli, Angela Di Martino, Alessandra Lo Presti, Stefano Fiore, Patrizia Bagnarelli, Silvia Fillo, Giovanni Faggioni, Riccardo De Sanctis, Antonella Fortunato, Anna Anselmo, Francesco Giordani, Vanessa Vera Fain, Nino D'Amore, Florigio Lista
EPI_ISL_856881	Department of Infectious Diseases, Istituto Superiore di Sanità, Roma, Italy; PO Cardarelli, Laboratorio Analisi Microbiologia e Virologia, Campobasso, Italy	Virology Laboratory, Scientific Department, Army Medical Center	Paola Stefanelli, Angela Di Martino, Alessandra Lo Presti, Stefano Fiore, Massimiliano Scutellà , Silvia Fillo, Giovanni Faggioni, Riccardo De Sanctis, Antonella Fortunato, Anna Anselmo, Francesco Giordani, Vanessa Vera Fain, Nino D'Amore, Florigio Lista
EPI_ISL_856882	Virology Laboratory, Scientific Department, Army Medical Center	Virology Laboratory, Scientific Department, Army Medical Center	Silvia Fillo, Giovanni Faggioni, Riccardo De Sanctis, Antonella Fortunato, Anella Monte, Anna Anselmo, Vanessa Vera Fain, Francesco Giordani, Nino D'Amore, Filippo Molinari, Giancarlo Petralito, Florigio Lista
EPI_ISL_856883	Virology Laboratory, Scientific Department, Army Medical Center	Virology Laboratory, Scientific Department, Army Medical Center	Silvia Fillo, Giovanni Faggioni, Riccardo De Sanctis, Antonella Fortunato, Anella Monte, Anna Anselmo, Vanessa Vera Fain, Francesco Giordani, Nino D'Amore, Filippo Molinari, Giancarlo Petralito, Florigio Lista
EPI_ISL_856884	Department of Infectious Diseases, Istituto Superiore di Sanità, Roma, Italy; PO Santa Chiara, Microbiologia e Virologia, Trento, Italy	Virology Laboratory, Scientific Department, Army Medical Center	Paola Stefanelli, Angela Di Martino, Alessandra Lo Presti, Stefano Fiore, Paolo Lanzafame , Silvia Fillo, Giovanni Faggioni, Riccardo De Sanctis, Antonella Fortunato, Anna Anselmo, Francesco Giordani, Vanessa Vera Fain, Nino D'Amore, Florigio Lista
EPI_ISL_856885	Virology Laboratory, Scientific Department, Army Medical Center	Virology Laboratory, Scientific Department, Army Medical Center	Silvia Fillo, Giovanni Faggioni, Riccardo De Sanctis, Antonella Fortunato, Anella Monte, Anna Anselmo, Vanessa Vera Fain, Francesco Giordani, Nino D'Amore, Filippo Molinari, Giancarlo Petralito, Florigio Lista
EPI_ISL_856886	Department of Infectious Diseases, Istituto Superiore di Sanità, Roma, Italy; PO Spirito Santo, UOC Microbiologia e Virologia Clinica, Pescara, Italy	Virology Laboratory, Scientific Department, Army Medical Center	Paola Stefanelli, Angela Di Martino, Alessandra Lo Presti, Stefano Fiore, Paolo Fazii, Silvia Fillo, Giovanni Faggioni, Riccardo De Sanctis, Antonella Fortunato, Anna Anselmo, Francesco Giordani, Vanessa Vera Fain, Nino D'Amore, Florigio Lista
EPI_ISL_856887	Department of Infectious Diseases, Istituto Superiore di Sanità, Roma, Italy; AUOC Policlinico Bari, UOC Igiene, Bari, Italy	Virology Laboratory, Scientific Department, Army Medical Center	Paola Stefanelli, Angela Di Martino, Alessandra Lo Presti, Stefano Fiore, Maria Chironna, Silvia Fillo, Giovanni Faggioni, Riccardo De Sanctis, Antonella Fortunato, Anna Anselmo, Francesco Giordani, Vanessa Vera Fain, Nino D'Amore, Florigio Lista
EPI_ISL_856888	Department of Infectious Diseases, Istituto Superiore di Sanità, Roma, Italy; A.O.R. San Carlo di Potenza, Laboratorio Microbiologia e Virologia, Potenza, Italy	Virology Laboratory, Scientific Department, Army Medical Center	Paola Stefanelli, Angela Di Martino, Alessandra Lo Presti, Stefano Fiore, Antonio Picerno, Silvia Fillo, Giovanni Faggioni, Riccardo De Sanctis, Antonella Fortunato, Anna Anselmo, Francesco Giordani, Vanessa Vera Fain, Nino D'Amore, Florigio Lista
EPI_ISL_856889	Department of Infectious Diseases, Istituto Superiore di Sanità, Roma, Italy	Virology Laboratory, Scientific Department, Army Medical Center	Paola Stefanelli, Angela Di Martino, Alessandra Lo Presti, Stefano Fiore, Silvia Fillo, Giovanni Faggioni, Riccardo De Sanctis, Antonella Fortunato, Anna Anselmo, Francesco Giordani, Vanessa Vera Fain, Nino D'Amore, Florigio Lista
EPI_ISL_856890	Department of Infectious Diseases, Istituto Superiore di Sanità, Roma, Italy; AO Annunziata, UOC Microbiologia e Virologia, Cosenza, Italy	Virology Laboratory, Scientific Department, Army Medical Center	Paola Stefanelli, Angela Di Martino, Alessandra Lo Presti, Stefano Fiore, Cristina Giraldi, Silvia Fillo, Giovanni Faggioni, Riccardo De Sanctis, Antonella Fortunato, Anna Anselmo, Francesco Giordani, Vanessa Vera Fain, Nino D'Amore, Florigio Lista
EPI_ISL_856891, EPI_ISL_856892	Department of Infectious Diseases, Istituto Superiore di Sanità, Roma, Italy; AORN dei Colli, UOC Microbiologia Virologia, Napoli, Italy	Virology Laboratory, Scientific Department, Army Medical Center	Paola Stefanelli, Angela Di Martino, Alessandra Lo Presti, Stefano Fiore, Luigi Atripaldi, Silvia Fillo, Giovanni Faggioni, Riccardo De Sanctis, Antonella Fortunato, Anna Anselmo, Francesco Giordani, Vanessa Vera Fain, Nino D'Amore, Florigio Lista
EPI_ISL_856893, EPI_ISL_856894	Department of Infectious Diseases, Istituto Superiore di Sanità, Roma, Italy; ASUITS - Ospedale Maggiore, S.C. UOC Igiene e Sanità Pubblica, Trieste, Italy	Virology Laboratory, Scientific Department, Army Medical Center	Paola Stefanelli, Angela Di Martino, Alessandra Lo Presti, Stefano Fiore, Pierlanfranco D'Agaro, Silvia Fillo, Giovanni Faggioni, Riccardo De Sanctis, Antonella Fortunato, Anna Anselmo, Francesco Giordani, Vanessa Vera Fain, Nino D'Amore, Florigio Lista
EPI_ISL_856895, EPI_ISL_856896	Department of Infectious Diseases, Istituto Superiore di Sanità, Roma, Italy; Ospedali Riuniti, Laboratorio Virologia, Ancona, Italy	Virology Laboratory, Scientific Department, Army Medical Center	Paola Stefanelli, Angela Di Martino, Alessandra Lo Presti, Stefano Fiore, Patrizia Bagnarelli, Silvia Fillo, Giovanni Faggioni, Riccardo De Sanctis, Antonella Fortunato, Anna Anselmo, Francesco Giordani, Vanessa Vera Fain, Nino D'Amore, Florigio Lista
EPI_ISL_856897	Department of Infectious Diseases, Istituto Superiore di Sanità, Roma, Italy; PO Cardarelli, Laboratorio Analisi Microbiologia e Virologia, Campobasso, Italy	Virology Laboratory, Scientific Department, Army Medical Center	Paola Stefanelli, Angela Di Martino, Alessandra Lo Presti, Stefano Fiore, Massimiliano Scutellà, Silvia Fillo, Giovanni Faggioni, Riccardo De Sanctis, Antonella Fortunato, Anna Anselmo, Francesco Giordani, Vanessa Vera Fain, Nino D'Amore, Florigio Lista
EPI_ISL_856898, EPI_ISL_856899	Department of Infectious Diseases, Istituto Superiore di Sanità, Roma, Italy; ASL Città di Torino, S.C. Microbiologia e Virologia, Torino, Italy	Virology Laboratory, Scientific Department, Army Medical Center	Paola Stefanelli, Angela Di Martino, Alessandra Lo Presti, Stefano Fiore, Valeria Ghisetti, Silvia Fillo, Giovanni Faggioni, Riccardo De Sanctis, Antonella Fortunato, Anna Anselmo, Francesco Giordani, Vanessa Vera Fain, Nino D'Amore, Florigio Lista
EPI_ISL_856900, EPI_ISL_856901	Department of Infectious Diseases, Istituto Superiore di Sanità, Roma, Italy; AOUP V. Emanuele di Catania, P.O. Gaspare Rodolico, Catania, Italy	Virology Laboratory, Scientific Department, Army Medical Center	Paola Stefanelli, Angela Di Martino, Alessandra Lo Presti, Stefano Fiore, Guido Scalia, Silvia Fillo, Giovanni Faggioni, Riccardo De Sanctis, Antonella Fortunato, Anna Anselmo, Francesco Giordani, Vanessa Vera Fain, Nino D'Amore, Florigio Lista
EPI_ISL_856902	Department of Infectious Diseases, Istituto Superiore di Sanità, Roma, Italy; AS Alto Adige, Microbiologia e Virologia, Bolzano, Italy	Virology Laboratory, Scientific Department, Army Medical Center	Paola Stefanelli, Angela Di Martino, Alessandra Lo Presti, Stefano Fiore, Elisabetta Pagani, Silvia Fillo, Giovanni Faggioni, Riccardo De Sanctis, Antonella Fortunato, Anna Anselmo, Francesco Giordani, Vanessa Vera Fain, Nino D'Amore, Florigio Lista
EPI_ISL_856903	Department of Infectious Diseases, Istituto Superiore di Sanità, Roma, Italy; PO Santa Chiara, Microbiologia e Virologia, Trento, Italy	Virology Laboratory, Scientific Department, Army Medical Center	Paola Stefanelli, Angela Di Martino, Alessandra Lo Presti, Stefano Fiore, Paolo Lanzafame, Silvia Fillo, Giovanni Faggioni, Riccardo De Sanctis, Antonella Fortunato, Anna Anselmo, Francesco Giordani, Vanessa Vera Fain, Nino D'Amore, Florigio Lista
EPI_ISL_856904	Department of Infectious Diseases, Istituto Superiore di Sanità, Roma, Italy	Virology Laboratory, Scientific Department, Army Medical Center	Paola Stefanelli, Angela Di Martino, Alessandra Lo Presti, Stefano Fiore, Silvia Fillo, Giovanni Faggioni, Riccardo De Sanctis, Antonella Fortunato, Anna Anselmo, Francesco Giordani, Vanessa Vera Fain, Nino D'Amore, Florigio Lista
EPI_ISL_856905, EPI_ISL_856906	Department of Infectious Diseases, Istituto Superiore di Sanità, Roma, Italy; AO S.M. della Misericordia, S.C. Microbiologia, Perugia, Italy	Virology Laboratory, Scientific Department, Army Medical Center	Paola Stefanelli, Angela Di Martino, Alessandra Lo Presti, Stefano Fiore, Barbara Camilloni, Silvia Fillo, Giovanni Faggioni, Riccardo De Sanctis, Antonella Fortunato, Anna Anselmo, Francesco Giordani, Vanessa Vera Fain, Nino D'Amore, Florigio Lista
EPI_ISL_856907	Department of Infectious Diseases, Istituto Superiore di Sanità, Roma, Italy; AUSL Valle d'Aosta, Aosta, Italy	Virology Laboratory, Scientific Department, Army Medical Center	Paola Stefanelli, Angela Di Martino, Alessandra Lo Presti, Stefano Fiore, Massimo Di Benedetto, Silvia Fillo, Giovanni Faggioni, Riccardo De Sanctis, Antonella Fortunato, Anna Anselmo, Francesco Giordani, Vanessa Vera Fain, Nino D'Amore, Florigio Lista
EPI_ISL_856908	Department of Infectious Diseases, Istituto Superiore di Sanità, Roma, Italy; PO Madre Teresa di Calcutta, UOC. Laboratorio Analisi ULSS 6 Euganea, Padova, Italy	Virology Laboratory, Scientific Department, Army Medical Center	Paola Stefanelli, Angela Di Martino, Alessandra Lo Presti, Stefano Fiore, Giacomo Mezzapelle, Silvia Fillo, Giovanni Faggioni, Riccardo De Sanctis, Antonella Fortunato, Anna Anselmo, Francesco Giordani, Vanessa Vera Fain, Nino D'Amore, Florigio Lista

EPI_ISL_856909	Department of Infectious Diseases, Istituto Superiore di Sanità, Roma, Italy; ULSS 8 Berica Vicenza, UOC Microbiologia, Vicenza, Italy	Virology Laboratory, Scientific Department, Army Medical Center	Paola Stefanelli, Angela Di Martino, Alessandra Lo Presti, Stefano Fiore, Mario Rassu, Silvia Fillo, Giovanni Faggioni, Riccardo De Sanctis, Antonella Fortunato, Anna Anselmo, Francesco Giordani, Vanessa Vera Fain, Nino D'Amore, Florigio Lista
EPI_ISL_869166	Laboratory of Microbiology,ASST Settelaghi, Varese, Italy	Laboratory of Microbiology,ASST Settelaghi, Varese, Italy	Maggi,F., Novazzi,F., Genoni,A., Baj,A., Spezia,P.G., Focosi,D., Zago,C., Colombo,A., Cassani,G., Pasciuta,R., Tamborini,A.,Rossi,A., Prestia,M., Capuano,R., Azzi,L., Donadini,A., Catanoso,G., Grossi,P., Maffioli,L. and Bonelli,G.
EPI_ISL_869240	Laboratory of Microbiology, ASST Settelaghi, Varese, Italy	Laboratory of Microbiology, ASST Settelaghi, Varese, Italy	Novazzi,F., Genoni,A., Focosi,D., Baj,A., Spezia,P.G., Zago,C., Colombo,A., Cassani,G., Pasciuta,R., Tamborini,A.,Rossi,A., Prestia,M., Capuano,R., Azzi,L., Donadini,A.,Catanoso,G., Maggi,F.
EPI_ISL_873209	Medicine and Surgery, University of Insubria	Medicine and Surgery, University of Insubria	Maggi,F., Novazzi,F., Genoni,A., Baj,A., Spezia,P.G., Focosi,D.,Zago,C., Colombo,A., Cassani,G., Pasciuta,R., Tamborini,A.,Rossi,A., Prestia,M., Capuano,R., Azzi,L., Donadini,A.,Catanoso,G., Grossi,P., Maffioli,L. and Bonelli,G.
EPI_ISL_875566, EPI_ISL_875567, EPI_ISL_875568	SIESP L'AQUILA	Istituto Zooprofilattico Sperimentale dell'Abruzzo e Molise "G. Caporale"	Lorusso A, Marcacci M, Di Domenico M, Ancora M, Curini V, Mangone I, Rinaldi A, Scialabba S, Di Pasquale A, Cammà C, Puglia I, Calistri P, Savini G
EPI_ISL_883155	San Donato Arezzo Analysis Laboratory - Clinical Molecular Pathology sector	San Donato Arezzo Analysis Laboratory - Clinical Molecular Pathology sector	Alessandro Pancrazzi and Alice Moncada
EPI_ISL_883286, EPI_ISL_883287	SIESP DIPARTIMENTO DI PREVENZIONE CHIE	Istituto Zooprofilattico Sperimentale dell'Abruzzo e Molise "G. Caporale"	Lorusso A, Marcacci M, Di Domenico M, Ancora M, Curini V, Mangone I, Rinaldi A, Scialabba S, Di Pasquale A, Cammà C, Puglia I, Calistri P, Savini G
EPI_ISL_883288, EPI_ISL_883289	SIESP CHIETI-DRIVE IN ORTONA	Istituto Zooprofilattico Sperimentale dell'Abruzzo e Molise "G. Caporale"	Lorusso A, Marcacci M, Di Domenico M, Ancora M, Curini V, Mangone I, Rinaldi A, Scialabba S, Di Pasquale A, Cammà C, Puglia I, Calistri P, Savini G
EPI_ISL_883290	SIESP CHIETI-DRIVE IN CHIETI	Istituto Zooprofilattico Sperimentale dell'Abruzzo e Molise "G. Caporale"	Lorusso A, Marcacci M, Di Domenico M, Ancora M, Curini V, Mangone I, Rinaldi A, Scialabba S, Di Pasquale A, Cammà C, Puglia I, Calistri P, Savini G
EPI_ISL_883291	RP Guardiagrele-Ospedale di Comunità	Istituto Zooprofilattico Sperimentale dell'Abruzzo e Molise "G. Caporale"	Lorusso A, Marcacci M, Di Domenico M, Ancora M, Curini V, Mangone I, Rinaldi A, Scialabba S, Di Pasquale A, Cammà C, Puglia I, Calistri P, Savini G
EPI_ISL_883292	SIESP CHIETI-DRIVE IN ORTONA	Istituto Zooprofilattico Sperimentale dell'Abruzzo e Molise "G. Caporale"	Lorusso A, Marcacci M, Di Domenico M, Ancora M, Curini V, Mangone I, Rinaldi A, Scialabba S, Di Pasquale A, Cammà C, Puglia I, Calistri P, Savini G
EPI_ISL_883293	SIESP CHIETI-DRIVE IN LANCIANO	Istituto Zooprofilattico Sperimentale dell'Abruzzo e Molise "G. Caporale"	Lorusso A, Marcacci M, Di Domenico M, Ancora M, Curini V, Mangone I, Rinaldi A, Scialabba S, Di Pasquale A, Cammà C, Puglia I, Calistri P, Savini G
EPI_ISL_883294, EPI_ISL_883295	RP Guardiagrele-Ospedale di Comunità	Istituto Zooprofilattico Sperimentale dell'Abruzzo e Molise "G. Caporale"	Lorusso A, Marcacci M, Di Domenico M, Ancora M, Curini V, Mangone I, Rinaldi A, Scialabba S, Di Pasquale A, Cammà C, Puglia I, Calistri P, Savini G
EPI_ISL_883296	SIESP CHIETI-DRIVE IN ORTONA	Istituto Zooprofilattico Sperimentale dell'Abruzzo e Molise "G. Caporale"	Lorusso A, Marcacci M, Di Domenico M, Ancora M, Curini V, Mangone I, Rinaldi A, Scialabba S, Di Pasquale A, Cammà C, Puglia I, Calistri P, Savini G
EPI_ISL_883297	RP Guardiagrele-Ospedale di Comunità	Istituto Zooprofilattico Sperimentale dell'Abruzzo e Molise "G. Caporale"	Lorusso A, Marcacci M, Di Domenico M, Ancora M, Curini V, Mangone I, Rinaldi A, Scialabba S, Di Pasquale A, Cammà C, Puglia I, Calistri P, Savini G
EPI_ISL_883298, EPI_ISL_883299	SIESP DIPARTIMENTO DI PREVENZIONE CHIE	Istituto Zooprofilattico Sperimentale dell'Abruzzo e Molise "G. Caporale"	Lorusso A, Marcacci M, Di Domenico M, Ancora M, Curini V, Mangone I, Rinaldi A, Scialabba S, Di Pasquale A, Cammà C, Puglia I, Calistri P, Savini G
EPI_ISL_883300	SIESP CHIETI-DRIVE IN CHIETI	Istituto Zooprofilattico Sperimentale dell'Abruzzo e Molise "G. Caporale"	Lorusso A, Marcacci M, Di Domenico M, Ancora M, Curini V, Mangone I, Rinaldi A, Scialabba S, Di Pasquale A, Cammà C, Puglia I, Calistri P, Savini G
EPI_ISL_883301, EPI_ISL_883302	RP Guardiagrele-Ospedale di Comunità	Istituto Zooprofilattico Sperimentale dell'Abruzzo e Molise "G. Caporale"	Lorusso A, Marcacci M, Di Domenico M, Ancora M, Curini V, Mangone I, Rinaldi A, Scialabba S, Di Pasquale A, Cammà C, Puglia I, Calistri P, Savini G
EPI_ISL_883303, EPI_ISL_883304, EPI_ISL_883305	SIESP DIPARTIMENTO DI PREVENZIONE CHIE	Istituto Zooprofilattico Sperimentale dell'Abruzzo e Molise "G. Caporale"	Lorusso A, Marcacci M, Di Domenico M, Ancora M, Curini V, Mangone I, Rinaldi A, Scialabba S, Di Pasquale A, Cammà C, Puglia I, Calistri P, Savini G
EPI_ISL_884865	Medicine and Surgery, University of Insubria	University of Insubria	Novazzi,F., Genoni,A., Focosi,D., Baj,A., Spezia,P.G., Zago,C.,Colombo,A., Cassani,G., Pasciuta,R., Tamborini,A., Rossi,A.,Prestia,M., Capuano,R., Azzi,L., Donadini,A., Catanoso,G. and Maggi,F.
EPI_ISL_902754	University Hospital Sant'Andrea-Sapienza	INMI Lazzaro Spallanzani IRCCS	B Bartolini, E Giombini, M Rueca, O Butera, F Messina, C.E.M Gruber, M Simmaco, I Santino, A Di Caro, MR Capobianchi
EPI_ISL_902755	IRCCS San Raffaele	INMI Lazzaro Spallanzani IRCCS	E Giombini, M Rueca, O Butera, F Messina, C.E.M Gruber, B Bartolini, D Russo, D Limongi, MR Capobianchi, A Di Caro
EPI_ISL_902756	Fondazione Policlinico Universitario "A. Gemelli" IRCCS	INMI Lazzaro Spallanzani IRCCS	M Rueca, O Butera, F Messina, C.E.M Gruber, B Bartolini, E Giombini, P Cattani, M Sanguinetti, MR Capobianchi, A Di Caro
EPI_ISL_911525	Microbiology and Virology Unit, Florence Careggi University Hospital	Microbiology and Virology Unit, Florence Careggi University Hospital	Vincenzo Di Pilato, Marco Coppi, Fabio Morecchiato, Noemi Aiezza, Ilaria Baccani, Alberto Antonelli, Emanuele Gori, Gian Maria Rossolini
EPI_ISL_913446	Laboratorio Genzano - ASL RM 6	INMI Lazzaro Spallanzani IRCCS	Emanuela Giombini, Martina Rueca, Barbara Bartolini, Ornella Butera, Cesare E.M. Gruber, Francesco Messina, Grazia Tramini, Emanuela Conti, Antonino Di Caro, Maria R. Capobianchi
EPI_ISL_913447	Laboratorio Genzano - ASL RM 6	INMI Lazzaro Spallanzani IRCCS	Francesco Messina, Emanuela Giombini, Ornella Butera, Cesare EM Gruber, Martina Rueca, Barbara Bartolini, Grazia Tramini, Emanuela Conti, Maria R Capobianchi, Antonino Di Caro
EPI_ISL_918269	SIESP DIPARTIMENTO DI PREVENZIONE TERAMO	Istituto Zooprofilattico Sperimentale dell'Abruzzo e Molise "G. Caporale"	Lorusso A, Marcacci M, Di Domenico M, Ancora M, Curini V, Mangone I, Rinaldi A, Scialabba S, Di Pasquale A, Cammà C, Puglia I, Calistri P, Savini G
EPI_ISL_918410	Ospedale Di Venere - Carbonara	Istituto Zooprofilattico Sperimentale della Puglia e della Basilicata	Parisi A., Bianco A., Capozzi L., Del Sambro L., Simone D., Manzulli V, Rondinone V., Pace L., Cipolletta D., Galante D.
EPI_ISL_918483	Laboratory of Microbiology, ASST Settelaghi, Varese, Italy	Laboratory of Microbiology, ASST Settelaghi, Varese, Italy	Novazzi,F., Genoni,A., Baj,A., Focosi,D., Spezia,P.G., Zago,C., Colombo,A., Cassani,G., Pasciuta,R., Tamborini,A., Rossi,A., Prestia,M., Capuano,R., Maggi,F.
EPI_ISL_936486	Medicine and Surgery, University of Insubria	Medicine and Surgery, University of Insubria	Novazzi,F., Genoni,A., Baj,A., Spezia,P.G., Focosi,D., Zago,C.,Colombo,A., Cassani,G., Pasciuta,R., Tamborini,A., Rossi,A.,Prestia,M., Capuano,R. and Maggi,F.
EPI_ISL_940565, EPI_ISL_940631, EPI_ISL_940632, EPI_ISL_940739, EPI_ISL_949182	University of Bari Biomedical Sciences and Human Oncology	University of Bari Biomedical Sciences and Human Oncology	Chironna M., Sallustio A., Loconsole D., Accogli M.
EPI_ISL_949184	University of Bari Biomedical Sciences and Human Oncology	University of Bari Biomedical Sciences and Human Oncology	Chironna M., Sallustio A., Loconsole D., Accogli A.
EPI_ISL_949185	University of Bari Biomedical Sciences and Human Oncology	University of Bari Biomedical Sciences and Human Oncology	Chironna M., Sallustio A., Loconsole D., Accogli M.
EPI_ISL_949188	University of Bari Biomedical Sciences and Human Oncology	University of Bari Biomedical Sciences and Human Oncology	Chironna A., Sallustio A., Loconsole D., Accogli M.
EPI_ISL_949191	University of Bari Biomedical Sciences and Human Oncology	University of Bari Biomedical Sciences and Human Oncology	Chironna M., Sallustio A., Loconsole D., Accogli M.
EPI_ISL_961018	SIESP CHIETI - DRIVE IN CHIETI	Istituto Zooprofilattico Sperimentale dell'Abruzzo e Molise "G. Caporale"	Lorusso A, Marcacci M, Di Domenico M, Ancora M, Curini V, Mangone I, Rinaldi A, Scialabba S, Di Pasquale A, Cammà C, Puglia I, Calistri P, Savini G
EPI_ISL_961019, EPI_ISL_961020	SIESP CHIETI - DRIVE IN ORTONA	Istituto Zooprofilattico Sperimentale dell'Abruzzo e Molise "G. Caporale"	Lorusso A, Marcacci M, Di Domenico M, Ancora M, Curini V, Mangone I, Rinaldi A, Scialabba S, Di Pasquale A, Cammà C, Puglia I, Calistri P, Savini G

EPI_ISL_961021, EPI_ISL_961022, EPI_ISL_961023, EPI_ISL_961024, EPI_ISL_961025, EPI_ISL_961026	SIESP CHIETI - DRIVE IN CHIETI	Istituto Zooprofilattico Sperimentale dell'Abruzzo e Molise "G. Caporale"	Lorusso A, Marcacci M, Di Domenico M, Ancora M, Curini V, Mangone I, Rinaldi A, Scialabba S, Di Pasquale A, Cammà C, Puglia I, Calistri P, Savini G
EPI_ISL_961027	SIESP CHIETI-DRIVE IN VASTO	Istituto Zooprofilattico Sperimentale dell'Abruzzo e Molise "G. Caporale"	Lorusso A, Marcacci M, Di Domenico M, Ancora M, Curini V, Mangone I, Rinaldi A, Scialabba S, Di Pasquale A, Cammà C, Puglia I, Calistri P, Savini G
EPI_ISL_961028, EPI_ISL_961029, EPI_ISL_961030, EPI_ISL_961031	SIESP CHIETI - DRIVE IN CHIETI	Istituto Zooprofilattico Sperimentale dell'Abruzzo e Molise "G. Caporale"	Lorusso A, Marcacci M, Di Domenico M, Ancora M, Curini V, Mangone I, Rinaldi A, Scialabba S, Di Pasquale A, Cammà C, Puglia I, Calistri P, Savini G
EPI_ISL_961032	SIESP DIPARTIMENTO DI PREVENZIONE CHIETI	Istituto Zooprofilattico Sperimentale dell'Abruzzo e Molise "G. Caporale"	Lorusso A, Marcacci M, Di Domenico M, Ancora M, Curini V, Mangone I, Rinaldi A, Scialabba S, Di Pasquale A, Cammà C, Puglia I, Calistri P, Savini G
EPI_ISL_961033	SIESP CHIETI - DRIVE IN ORTONA	Istituto Zooprofilattico Sperimentale dell'Abruzzo e Molise "G. Caporale"	Lorusso A, Marcacci M, Di Domenico M, Ancora M, Curini V, Mangone I, Rinaldi A, Scialabba S, Di Pasquale A, Cammà C, Puglia I, Calistri P, Savini G
EPI_ISL_961034, EPI_ISL_961035	SIESP CHIETI - DRIVE IN CHIETI	Istituto Zooprofilattico Sperimentale dell'Abruzzo e Molise "G. Caporale"	Lorusso A, Marcacci M, Di Domenico M, Ancora M, Curini V, Mangone I, Rinaldi A, Scialabba S, Di Pasquale A, Cammà C, Puglia I, Calistri P, Savini G
EPI_ISL_961036	SIESP CHIETI - DRIVE IN ORTONA	Istituto Zooprofilattico Sperimentale dell'Abruzzo e Molise "G. Caporale"	Lorusso A, Marcacci M, Di Domenico M, Ancora M, Curini V, Mangone I, Rinaldi A, Scialabba S, Di Pasquale A, Cammà C, Puglia I, Calistri P, Savini G
EPI_ISL_961037, EPI_ISL_961038, EPI_ISL_961039, EPI_ISL_961040, EPI_ISL_961041	SIESP CHIETI - DRIVE IN CHIETI	Istituto Zooprofilattico Sperimentale dell'Abruzzo e Molise "G. Caporale"	Lorusso A, Marcacci M, Di Domenico M, Ancora M, Curini V, Mangone I, Rinaldi A, Scialabba S, Di Pasquale A, Cammà C, Puglia I, Calistri P, Savini G
EPI_ISL_961042	SIESP TERAMO	Istituto Zooprofilattico Sperimentale dell'Abruzzo e Molise "G. Caporale"	Lorusso A, Marcacci M, Di Domenico M, Ancora M, Curini V, Mangone I, Rinaldi A, Scialabba S, Di Pasquale A, Cammà C, Puglia I, Calistri P, Savini G
EPI_ISL_961043	SIESP CHIETI - DRIVE IN CHIETI	Istituto Zooprofilattico Sperimentale dell'Abruzzo e Molise "G. Caporale"	Lorusso A, Marcacci M, Di Domenico M, Ancora M, Curini V, Mangone I, Rinaldi A, Scialabba S, Di Pasquale A, Cammà C, Puglia I, Calistri P, Savini G
EPI_ISL_961044, EPI_ISL_961045, EPI_ISL_961046	SIESP CHIETI - DRIVE IN ORTONA	Istituto Zooprofilattico Sperimentale dell'Abruzzo e Molise "G. Caporale"	Lorusso A, Marcacci M, Di Domenico M, Ancora M, Curini V, Mangone I, Rinaldi A, Scialabba S, Di Pasquale A, Cammà C, Puglia I, Calistri P, Savini G
EPI_ISL_961047	SIESP CHIETI - DRIVE IN CHIETI	Istituto Zooprofilattico Sperimentale dell'Abruzzo e Molise "G. Caporale"	Lorusso A, Marcacci M, Di Domenico M, Ancora M, Curini V, Mangone I, Rinaldi A, Scialabba S, Di Pasquale A, Cammà C, Puglia I, Calistri P, Savini G
EPI_ISL_961048, EPI_ISL_961049	SIESP CHIETI - DRIVE IN ORTONA	Istituto Zooprofilattico Sperimentale dell'Abruzzo e Molise "G. Caporale"	Lorusso A, Marcacci M, Di Domenico M, Ancora M, Curini V, Mangone I, Rinaldi A, Scialabba S, Di Pasquale A, Cammà C, Puglia I, Calistri P, Savini G
EPI_ISL_961050, EPI_ISL_961051, EPI_ISL_961052, EPI_ISL_961053, EPI_ISL_961054	SIESP DIPARTIMENTO DI PREVENZIONE CHIETI	Istituto Zooprofilattico Sperimentale dell'Abruzzo e Molise "G. Caporale"	Lorusso A, Marcacci M, Di Domenico M, Ancora M, Curini V, Mangone I, Rinaldi A, Scialabba S, Di Pasquale A, Cammà C, Puglia I, Calistri P, Savini G
EPI_ISL_961055, EPI_ISL_961056	SIESP CHIETI - DRIVE IN ORTONA	Istituto Zooprofilattico Sperimentale dell'Abruzzo e Molise "G. Caporale"	Lorusso A, Marcacci M, Di Domenico M, Ancora M, Curini V, Mangone I, Rinaldi A, Scialabba S, Di Pasquale A, Cammà C, Puglia I, Calistri P, Savini G
EPI_ISL_961057, EPI_ISL_961058, EPI_ISL_961059, EPI_ISL_961060	SIESP CHIETI - DRIVE IN CHIETI	Istituto Zooprofilattico Sperimentale dell'Abruzzo e Molise "G. Caporale"	Lorusso A, Marcacci M, Di Domenico M, Ancora M, Curini V, Mangone I, Rinaldi A, Scialabba S, Di Pasquale A, Cammà C, Puglia I, Calistri P, Savini G
EPI_ISL_961061	SIESP CHIETI - DRIVE IN ORTONA	Istituto Zooprofilattico Sperimentale dell'Abruzzo e Molise "G. Caporale"	Lorusso A, Marcacci M, Di Domenico M, Ancora M, Curini V, Mangone I, Rinaldi A, Scialabba S, Di Pasquale A, Cammà C, Puglia I, Calistri P, Savini G
EPI_ISL_961062	SIESP CHIETI - DRIVE IN CHIETI	Istituto Zooprofilattico Sperimentale dell'Abruzzo e Molise "G. Caporale"	Lorusso A, Marcacci M, Di Domenico M, Ancora M, Curini V, Mangone I, Rinaldi A, Scialabba S, Di Pasquale A, Cammà C, Puglia I, Calistri P, Savini G
EPI_ISL_961063	SIESP DIPARTIMENTO DI PREVENZIONE CHIETI	Istituto Zooprofilattico Sperimentale dell'Abruzzo e Molise "G. Caporale"	Lorusso A, Marcacci M, Di Domenico M, Ancora M, Curini V, Mangone I, Rinaldi A, Scialabba S, Di Pasquale A, Cammà C, Puglia I, Calistri P, Savini G
EPI_ISL_961064, EPI_ISL_961065	SIESP CHIETI - DRIVE IN ORTONA	Istituto Zooprofilattico Sperimentale dell'Abruzzo e Molise "G. Caporale"	Lorusso A, Marcacci M, Di Domenico M, Ancora M, Curini V, Mangone I, Rinaldi A, Scialabba S, Di Pasquale A, Cammà C, Puglia I, Calistri P, Savini G
EPI_ISL_961066	SIESP CHIETI - DRIVE IN LANCIANO	Istituto Zooprofilattico Sperimentale dell'Abruzzo e Molise "G. Caporale"	Lorusso A, Marcacci M, Di Domenico M, Ancora M, Curini V, Mangone I, Rinaldi A, Scialabba S, Di Pasquale A, Cammà C, Puglia I, Calistri P, Savini G
EPI_ISL_961067, EPI_ISL_961068	SIESP DIPARTIMENTO DI PREVENZIONE CHIETI	Istituto Zooprofilattico Sperimentale dell'Abruzzo e Molise "G. Caporale"	Lorusso A, Marcacci M, Di Domenico M, Ancora M, Curini V, Mangone I, Rinaldi A, Scialabba S, Di Pasquale A, Cammà C, Puglia I, Calistri P, Savini G
EPI_ISL_961069	SIESP CHIETI - DRIVE IN CHIETI	Istituto Zooprofilattico Sperimentale dell'Abruzzo e Molise "G. Caporale"	Lorusso A, Marcacci M, Di Domenico M, Ancora M, Curini V, Mangone I, Rinaldi A, Scialabba S, Di Pasquale A, Cammà C, Puglia I, Calistri P, Savini G
EPI_ISL_961070	SIESP DIPARTIMENTO DI PREVENZIONE CHIETI	Istituto Zooprofilattico Sperimentale dell'Abruzzo e Molise "G. Caporale"	Lorusso A, Marcacci M, Di Domenico M, Ancora M, Curini V, Mangone I, Rinaldi A, Scialabba S, Di Pasquale A, Cammà C, Puglia I, Calistri P, Savini G
EPI_ISL_961071, EPI_ISL_961072, EPI_ISL_961073	SIESP CHIETI - DRIVE IN CHIETI	Istituto Zooprofilattico Sperimentale dell'Abruzzo e Molise "G. Caporale"	Lorusso A, Marcacci M, Di Domenico M, Ancora M, Curini V, Mangone I, Rinaldi A, Scialabba S, Di Pasquale A, Cammà C, Puglia I, Calistri P, Savini G
EPI_ISL_961074	SIESP CHIETI - DRIVE IN ORTONA	Istituto Zooprofilattico Sperimentale dell'Abruzzo e Molise "G. Caporale"	Lorusso A, Marcacci M, Di Domenico M, Ancora M, Curini V, Mangone I, Rinaldi A, Scialabba S, Di Pasquale A, Cammà C, Puglia I, Calistri P, Savini G
EPI_ISL_961075	SIESP DIPARTIMENTO DI PREVENZIONE CHIETI	Istituto Zooprofilattico Sperimentale dell'Abruzzo e Molise "G. Caporale"	Lorusso A, Marcacci M, Di Domenico M, Ancora M, Curini V, Mangone I, Rinaldi A, Scialabba S, Di Pasquale A, Cammà C, Puglia I, Calistri P, Savini G
EPI_ISL_961076	SIESP CHIETI - DRIVE IN ORTONA	Istituto Zooprofilattico Sperimentale dell'Abruzzo e Molise "G. Caporale"	Lorusso A, Marcacci M, Di Domenico M, Ancora M, Curini V, Mangone I, Rinaldi A, Scialabba S, Di Pasquale A, Cammà C, Puglia I, Calistri P, Savini G
EPI_ISL_961077	SIESP TERAMO	Istituto Zooprofilattico Sperimentale dell'Abruzzo e Molise "G. Caporale"	Lorusso A, Marcacci M, Di Domenico M, Ancora M, Curini V, Mangone I, Rinaldi A, Scialabba S, Di Pasquale A, Cammà C, Puglia I, Calistri P, Savini G
EPI_ISL_961078, EPI_ISL_961079, EPI_ISL_961080	DIP. PREV. AVEZZANO SERVIZIO DI IGIENE EPIDEMIOLOGIAE SANITA' PUBBLICA	Istituto Zooprofilattico Sperimentale dell'Abruzzo e Molise "G. Caporale"	Lorusso A, Marcacci M, Di Domenico M, Ancora M, Curini V, Mangone I, Rinaldi A, Scialabba S, Di Pasquale A, Cammà C, Puglia I, Calistri P, Savini G
EPI_ISL_961081	SIESP CHIETI - DRIVE IN CHIETI	Istituto Zooprofilattico Sperimentale dell'Abruzzo e Molise "G. Caporale"	Lorusso A, Marcacci M, Di Domenico M, Ancora M, Curini V, Mangone I, Rinaldi A, Scialabba S, Di Pasquale A, Cammà C, Puglia I, Calistri P, Savini G
EPI_ISL_961082	SIESP DIPARTIMENTO DI PREVENZIONE CHIETI	Istituto Zooprofilattico Sperimentale dell'Abruzzo e Molise "G. Caporale"	Lorusso A, Marcacci M, Di Domenico M, Ancora M, Curini V, Mangone I, Rinaldi A, Scialabba S, Di Pasquale A, Cammà C, Puglia I, Calistri P, Savini G
EPI_ISL_961083	DIP. PREV. AVEZZANO SERVIZIO DI IGIENE EPIDEMIOLOGIAE SANITA' PUBBLICA	Istituto Zooprofilattico Sperimentale dell'Abruzzo e Molise "G. Caporale"	Lorusso A, Marcacci M, Di Domenico M, Ancora M, Curini V, Mangone I, Rinaldi A, Scialabba S, Di Pasquale A, Cammà C, Puglia I, Calistri P, Savini G
EPI_ISL_961084	SIESP TERAMO	Istituto Zooprofilattico Sperimentale dell'Abruzzo e Molise "G. Caporale"	Lorusso A, Marcacci M, Di Domenico M, Ancora M, Curini V, Mangone I, Rinaldi A, Scialabba S, Di Pasquale A, Cammà C, Puglia I, Calistri P, Savini G

EPI_ISL_961085	SIESP CHIETI - DRIVE IN ORTONA	Istituto Zooprofilattico Sperimentale dell'Abruzzo e Molise "G. Caporale"	Lorusso A, Marcacci M, Di Domenico M, Ancora M, Curini V, Mangone I, Rinaldi A, Scialabba S, Di Pasquale A, Cammà C, Puglia I, Calistri P, Savini G
EPI_ISL_961086	SIESP CHIETI - DRIVE IN CHIETI	Istituto Zooprofilattico Sperimentale dell'Abruzzo e Molise "G. Caporale"	Lorusso A, Marcacci M, Di Domenico M, Ancora M, Curini V, Mangone I, Rinaldi A, Scialabba S, Di Pasquale A, Cammà C, Puglia I, Calistri P, Savini G
EPI_ISL_961087, EPI_ISL_961088, EPI_ISL_961089	SIESP DIPARTIMENTO DI PREVENZIONE CHIETI	Istituto Zooprofilattico Sperimentale dell'Abruzzo e Molise "G. Caporale"	Lorusso A, Marcacci M, Di Domenico M, Ancora M, Curini V, Mangone I, Rinaldi A, Scialabba S, Di Pasquale A, Cammà C, Puglia I, Calistri P, Savini G
EPI_ISL_961090, EPI_ISL_961091, EPI_ISL_961092	SIESP CHIETI - DRIVE IN CHIETI	Istituto Zooprofilattico Sperimentale dell'Abruzzo e Molise "G. Caporale"	Lorusso A, Marcacci M, Di Domenico M, Ancora M, Curini V, Mangone I, Rinaldi A, Scialabba S, Di Pasquale A, Cammà C, Puglia I, Calistri P, Savini G
EPI_ISL_961093	Ospedale SS Annunziata	Istituto Zooprofilattico Sperimentale dell'Abruzzo e Molise "G. Caporale"	Lorusso A, Marcacci M, Di Domenico M, Ancora M, Curini V, Mangone I, Rinaldi A, Scialabba S, Di Pasquale A, Cammà C, Puglia I, Calistri P, Savini G
EPI_ISL_961094, EPI_ISL_961095	DIP. PREV. AVEZZANO SERVIZIO DI IGIENE EPIDEMIOLOGIAE SANITA' PUBBLICA	Istituto Zooprofilattico Sperimentale dell'Abruzzo e Molise "G. Caporale"	Lorusso A, Marcacci M, Di Domenico M, Ancora M, Curini V, Mangone I, Rinaldi A, Scialabba S, Di Pasquale A, Cammà C, Puglia I, Calistri P, Savini G
EPI_ISL_961096	SIESP L'AQUILA	Istituto Zooprofilattico Sperimentale dell'Abruzzo e Molise "G. Caporale"	Lorusso A, Marcacci M, Di Domenico M, Ancora M, Curini V, Mangone I, Rinaldi A, Scialabba S, Di Pasquale A, Cammà C, Puglia I, Calistri P, Savini G
EPI_ISL_961097, EPI_ISL_961098, EPI_ISL_961099, EPI_ISL_961100, EPI_ISL_961101, EPI_ISL_961102, EPI_ISL_961103	SIESP TERAMO	Istituto Zooprofilattico Sperimentale dell'Abruzzo e Molise "G. Caporale"	Lorusso A, Marcacci M, Di Domenico M, Ancora M, Curini V, Mangone I, Rinaldi A, Scialabba S, Di Pasquale A, Cammà C, Puglia I, Calistri P, Savini G
EPI_ISL_961673	SIESP CHIETI DRIVE IN LANCIANO	Istituto Zooprofilattico Sperimentale dell'Abruzzo e Molise "G. Caporale"	Lorusso A, Marcacci M, Di Domenico M, Ancora M, Curini V, Mangone I, Rinaldi A, Scialabba S, Di Pasquale A, Cammà C, Puglia I, Calistri P, Savini G
EPI_ISL_961674, EPI_ISL_961675, EPI_ISL_961676, EPI_ISL_961677	SIESP CHIETI - DRIVE IN CHIETI	Istituto Zooprofilattico Sperimentale dell'Abruzzo e Molise "G. Caporale"	Lorusso A, Marcacci M, Di Domenico M, Ancora M, Curini V, Mangone I, Rinaldi A, Scialabba S, Di Pasquale A, Cammà C, Puglia I, Calistri P, Savini G
EPI_ISL_961678	SIESP TERAMO	Istituto Zooprofilattico Sperimentale dell'Abruzzo e Molise "G. Caporale"	Lorusso A, Marcacci M, Di Domenico M, Ancora M, Curini V, Mangone I, Rinaldi A, Scialabba S, Di Pasquale A, Cammà C, Puglia I, Calistri P, Savini G
EPI_ISL_961679, EPI_ISL_961680, EPI_ISL_961681, EPI_ISL_961682	SIESP CHIETI - DRIVE IN CHIETI	Istituto Zooprofilattico Sperimentale dell'Abruzzo e Molise "G. Caporale"	Lorusso A, Marcacci M, Di Domenico M, Ancora M, Curini V, Mangone I, Rinaldi A, Scialabba S, Di Pasquale A, Cammà C, Puglia I, Calistri P, Savini G
EPI_ISL_961683	SIESP TERAMO	Istituto Zooprofilattico Sperimentale dell'Abruzzo e Molise "G. Caporale"	Lorusso A, Marcacci M, Di Domenico M, Ancora M, Curini V, Mangone I, Rinaldi A, Scialabba S, Di Pasquale A, Cammà C, Puglia I, Calistri P, Savini G
EPI_ISL_961684	SIESP CHIETI - DRIVE IN CHIETI	Istituto Zooprofilattico Sperimentale dell'Abruzzo e Molise "G. Caporale"	Lorusso A, Marcacci M, Di Domenico M, Ancora M, Curini V, Mangone I, Rinaldi A, Scialabba S, Di Pasquale A, Cammà C, Puglia I, Calistri P, Savini G
EPI_ISL_961685	SIESP DIPARTIMENTO DI PREVENZIONE CHIETI	Istituto Zooprofilattico Sperimentale dell'Abruzzo e Molise "G. Caporale"	Lorusso A, Marcacci M, Di Domenico M, Ancora M, Curini V, Mangone I, Rinaldi A, Scialabba S, Di Pasquale A, Cammà C, Puglia I, Calistri P, Savini G
EPI_ISL_961686, EPI_ISL_961687	SIESP CHIETI - DRIVE IN ORTONA	Istituto Zooprofilattico Sperimentale dell'Abruzzo e Molise "G. Caporale"	Lorusso A, Marcacci M, Di Domenico M, Ancora M, Curini V, Mangone I, Rinaldi A, Scialabba S, Di Pasquale A, Cammà C, Puglia I, Calistri P, Savini G
EPI_ISL_961688	SIESP TERAMO	Istituto Zooprofilattico Sperimentale dell'Abruzzo e Molise "G. Caporale"	Lorusso A, Marcacci M, Di Domenico M, Ancora M, Curini V, Mangone I, Rinaldi A, Scialabba S, Di Pasquale A, Cammà C, Puglia I, Calistri P, Savini G
EPI_ISL_961689, EPI_ISL_961690	SIESP CHIETI - DRIVE IN CHIETI	Istituto Zooprofilattico Sperimentale dell'Abruzzo e Molise "G. Caporale"	Lorusso A, Marcacci M, Di Domenico M, Ancora M, Curini V, Mangone I, Rinaldi A, Scialabba S, Di Pasquale A, Cammà C, Puglia I, Calistri P, Savini G
EPI_ISL_961691, EPI_ISL_961692, EPI_ISL_961693	SIESP CHIETI - DRIVE IN ORTONA	Istituto Zooprofilattico Sperimentale dell'Abruzzo e Molise "G. Caporale"	Lorusso A, Marcacci M, Di Domenico M, Ancora M, Curini V, Mangone I, Rinaldi A, Scialabba S, Di Pasquale A, Cammà C, Puglia I, Calistri P, Savini G
EPI_ISL_961694	SIESP CHIETI - DRIVE IN CHIETI	Istituto Zooprofilattico Sperimentale dell'Abruzzo e Molise "G. Caporale"	Lorusso A, Marcacci M, Di Domenico M, Ancora M, Curini V, Mangone I, Rinaldi A, Scialabba S, Di Pasquale A, Cammà C, Puglia I, Calistri P, Savini G
EPI_ISL_961695	SIESP DIPARTIMENTO DI PREVENZIONE CHIETI	Istituto Zooprofilattico Sperimentale dell'Abruzzo e Molise "G. Caporale"	Lorusso A, Marcacci M, Di Domenico M, Ancora M, Curini V, Mangone I, Rinaldi A, Scialabba S, Di Pasquale A, Cammà C, Puglia I, Calistri P, Savini G
EPI_ISL_961696	SIESP SULMONA	Istituto Zooprofilattico Sperimentale dell'Abruzzo e Molise "G. Caporale"	Lorusso A, Marcacci M, Di Domenico M, Ancora M, Curini V, Mangone I, Rinaldi A, Scialabba S, Di Pasquale A, Cammà C, Puglia I, Calistri P, Savini G
EPI_ISL_961697, EPI_ISL_961698	SIESP CHIETI - DRIVE IN CHIETI	Istituto Zooprofilattico Sperimentale dell'Abruzzo e Molise "G. Caporale"	Lorusso A, Marcacci M, Di Domenico M, Ancora M, Curini V, Mangone I, Rinaldi A, Scialabba S, Di Pasquale A, Cammà C, Puglia I, Calistri P, Savini G
EPI_ISL_961699	SIESP CHIETI - DRIVE IN ORTONA	Istituto Zooprofilattico Sperimentale dell'Abruzzo e Molise "G. Caporale"	Lorusso A, Marcacci M, Di Domenico M, Ancora M, Curini V, Mangone I, Rinaldi A, Scialabba S, Di Pasquale A, Cammà C, Puglia I, Calistri P, Savini G
EPI_ISL_961700	SIESP CHIETI - DRIVE IN CHIETI	Istituto Zooprofilattico Sperimentale dell'Abruzzo e Molise "G. Caporale"	Lorusso A, Marcacci M, Di Domenico M, Ancora M, Curini V, Mangone I, Rinaldi A, Scialabba S, Di Pasquale A, Cammà C, Puglia I, Calistri P, Savini G
EPI_ISL_961701	Ospedale Civile Teramo	Istituto Zooprofilattico Sperimentale dell'Abruzzo e Molise "G. Caporale"	Lorusso A, Marcacci M, Di Domenico M, Ancora M, Curini V, Mangone I, Rinaldi A, Scialabba S, Di Pasquale A, Cammà C, Puglia I, Calistri P, Savini G
EPI_ISL_961702	SIESP CHIETI - DRIVE IN CHIETI	Istituto Zooprofilattico Sperimentale dell'Abruzzo e Molise "G. Caporale"	Lorusso A, Marcacci M, Di Domenico M, Ancora M, Curini V, Mangone I, Rinaldi A, Scialabba S, Di Pasquale A, Cammà C, Puglia I, Calistri P, Savini G
EPI_ISL_961703	SIESP DIPARTIMENTO DI PREVENZIONE CHIETI	Istituto Zooprofilattico Sperimentale dell'Abruzzo e Molise "G. Caporale"	Lorusso A, Marcacci M, Di Domenico M, Ancora M, Curini V, Mangone I, Rinaldi A, Scialabba S, Di Pasquale A, Cammà C, Puglia I, Calistri P, Savini G
EPI_ISL_961704	SIESP SULMONA	Istituto Zooprofilattico Sperimentale dell'Abruzzo e Molise "G. Caporale"	Lorusso A, Marcacci M, Di Domenico M, Ancora M, Curini V, Mangone I, Rinaldi A, Scialabba S, Di Pasquale A, Cammà C, Puglia I, Calistri P, Savini G
EPI_ISL_961705	Ospedale San Salvatore UOC Malattie Infettive	Istituto Zooprofilattico Sperimentale dell'Abruzzo e Molise "G. Caporale"	Lorusso A, Marcacci M, Di Domenico M, Ancora M, Curini V, Mangone I, Rinaldi A, Scialabba S, Di Pasquale A, Cammà C, Puglia I, Calistri P, Savini G
EPI_ISL_961706	SIESP CHIETI - DRIVE IN CHIETI	Istituto Zooprofilattico Sperimentale dell'Abruzzo e Molise "G. Caporale"	Lorusso A, Marcacci M, Di Domenico M, Ancora M, Curini V, Mangone I, Rinaldi A, Scialabba S, Di Pasquale A, Cammà C, Puglia I, Calistri P, Savini G
EPI_ISL_961707, EPI_ISL_961708	SIESP DIPARTIMENTO DI PREVENZIONE CHIETI	Istituto Zooprofilattico Sperimentale dell'Abruzzo e Molise "G. Caporale"	Lorusso A, Marcacci M, Di Domenico M, Ancora M, Curini V, Mangone I, Rinaldi A, Scialabba S, Di Pasquale A, Cammà C, Puglia I, Calistri P, Savini G
EPI_ISL_961709, EPI_ISL_961710, EPI_ISL_961711	SIESP CHIETI - DRIVE IN CHIETI	Istituto Zooprofilattico Sperimentale dell'Abruzzo e Molise "G. Caporale"	Lorusso A, Marcacci M, Di Domenico M, Ancora M, Curini V, Mangone I, Rinaldi A, Scialabba S, Di Pasquale A, Cammà C, Puglia I, Calistri P, Savini G
EPI_ISL_961712, EPI_ISL_961713	SIESP CHIETI DRIVE IN LANCIANO	Istituto Zooprofilattico Sperimentale dell'Abruzzo e Molise "G. Caporale"	Lorusso A, Marcacci M, Di Domenico M, Ancora M, Curini V, Mangone I, Rinaldi A, Scialabba S, Di Pasquale A, Cammà C, Puglia I, Calistri P, Savini G
EPI_ISL_961714	SIESP CHIETI - DRIVE IN CHIETI	Istituto Zooprofilattico Sperimentale dell'Abruzzo e Molise "G.	Lorusso A, Marcacci M, Di Domenico M, Ancora M, Curini V, Mangone I, Rinaldi A, Scialabba S, Di Pasquale A, Cammà C, Puglia I, Calistri P, Savini G

		Caporale"	
EPI_ISL_961715, EPI_ISL_961716	SIESP CHIETI - DRIVE IN ORTONA	Istituto Zooprofilattico Sperimentale dell'Abruzzo e Molise "G. Caporale"	Lorusso A, Marcacci M, Di Domenico M, Ancora M, Curini V, Mangone I, Rinaldi A, Scialabba S, Di Pasquale A, Cammà C, Puglia I, Calistri P, Savini G
EPI_ISL_961717	SIESP CHIETI - DRIVE IN CHIETI	Istituto Zooprofilattico Sperimentale dell'Abruzzo e Molise "G. Caporale"	Lorusso A, Marcacci M, Di Domenico M, Ancora M, Curini V, Mangone I, Rinaldi A, Scialabba S, Di Pasquale A, Cammà C, Puglia I, Calistri P, Savini G
EPI_ISL_961718, EPI_ISL_961719, EPI_ISL_961720, EPI_ISL_961721, EPI_ISL_961722	SIESP CHIETI - DRIVE IN ORTONA	Istituto Zooprofilattico Sperimentale dell'Abruzzo e Molise "G. Caporale"	Lorusso A, Marcacci M, Di Domenico M, Ancora M, Curini V, Mangone I, Rinaldi A, Scialabba S, Di Pasquale A, Cammà C, Puglia I, Calistri P, Savini G
EPI_ISL_961723	SIESP CHIETI DRIVE IN LANCIANO	Istituto Zooprofilattico Sperimentale dell'Abruzzo e Molise "G. Caporale"	Lorusso A, Marcacci M, Di Domenico M, Ancora M, Curini V, Mangone I, Rinaldi A, Scialabba S, Di Pasquale A, Cammà C, Puglia I, Calistri P, Savini G
EPI_ISL_961724	SIESP CHIETI - DRIVE IN ORTONA	Istituto Zooprofilattico Sperimentale dell'Abruzzo e Molise "G. Caporale"	Lorusso A, Marcacci M, Di Domenico M, Ancora M, Curini V, Mangone I, Rinaldi A, Scialabba S, Di Pasquale A, Cammà C, Puglia I, Calistri P, Savini G
EPI_ISL_961725	SIESP CHIETI DRIVE IN GISSI	Istituto Zooprofilattico Sperimentale dell'Abruzzo e Molise "G. Caporale"	Lorusso A, Marcacci M, Di Domenico M, Ancora M, Curini V, Mangone I, Rinaldi A, Scialabba S, Di Pasquale A, Cammà C, Puglia I, Calistri P, Savini G
EPI_ISL_961726, EPI_ISL_961727	SIESP CHIETI - DRIVE IN CHIETI	Istituto Zooprofilattico Sperimentale dell'Abruzzo e Molise "G. Caporale"	Lorusso A, Marcacci M, Di Domenico M, Ancora M, Curini V, Mangone I, Rinaldi A, Scialabba S, Di Pasquale A, Cammà C, Puglia I, Calistri P, Savini G
EPI_ISL_961728	SIESP DIPARTIMENTO DI PREVENZIONE CHIETI	Istituto Zooprofilattico Sperimentale dell'Abruzzo e Molise "G. Caporale"	Lorusso A, Marcacci M, Di Domenico M, Ancora M, Curini V, Mangone I, Rinaldi A, Scialabba S, Di Pasquale A, Cammà C, Puglia I, Calistri P, Savini G
EPI_ISL_961729	SIESP CHIETI - DRIVE IN CHIETI	Istituto Zooprofilattico Sperimentale dell'Abruzzo e Molise "G. Caporale"	Lorusso A, Marcacci M, Di Domenico M, Ancora M, Curini V, Mangone I, Rinaldi A, Scialabba S, Di Pasquale A, Cammà C, Puglia I, Calistri P, Savini G
EPI_ISL_961730	Dipartimento Prevenzione Avezzano-Servizio Igiene epidemiologia Sanità Pubblica	Istituto Zooprofilattico Sperimentale dell'Abruzzo e Molise "G. Caporale"	Lorusso A, Marcacci M, Di Domenico M, Ancora M, Curini V, Mangone I, Rinaldi A, Scialabba S, Di Pasquale A, Cammà C, Puglia I, Calistri P, Savini G
EPI_ISL_961731, EPI_ISL_961732	SIESP CHIETI - DRIVE IN CHIETI	Istituto Zooprofilattico Sperimentale dell'Abruzzo e Molise "G. Caporale"	Lorusso A, Marcacci M, Di Domenico M, Ancora M, Curini V, Mangone I, Rinaldi A, Scialabba S, Di Pasquale A, Cammà C, Puglia I, Calistri P, Savini G
EPI_ISL_961733	SIESP CHIETI - DRIVE IN ORTONA	Istituto Zooprofilattico Sperimentale dell'Abruzzo e Molise "G. Caporale"	Lorusso A, Marcacci M, Di Domenico M, Ancora M, Curini V, Mangone I, Rinaldi A, Scialabba S, Di Pasquale A, Cammà C, Puglia I, Calistri P, Savini G
EPI_ISL_961734	Dipartimento Prevenzione Avezzano-Servizio Igiene epidemiologia Sanità Pubblica	Istituto Zooprofilattico Sperimentale dell'Abruzzo e Molise "G. Caporale"	Lorusso A, Marcacci M, Di Domenico M, Ancora M, Curini V, Mangone I, Rinaldi A, Scialabba S, Di Pasquale A, Cammà C, Puglia I, Calistri P, Savini G
EPI_ISL_961735, EPI_ISL_961736	SIESP CHIETI - DRIVE IN CHIETI	Istituto Zooprofilattico Sperimentale dell'Abruzzo e Molise "G. Caporale"	Lorusso A, Marcacci M, Di Domenico M, Ancora M, Curini V, Mangone I, Rinaldi A, Scialabba S, Di Pasquale A, Cammà C, Puglia I, Calistri P, Savini G
EPI_ISL_961737	SIESP CHIETI- DRIVE IN VASTO	Istituto Zooprofilattico Sperimentale dell'Abruzzo e Molise "G. Caporale"	Lorusso A, Marcacci M, Di Domenico M, Ancora M, Curini V, Mangone I, Rinaldi A, Scialabba S, Di Pasquale A, Cammà C, Puglia I, Calistri P, Savini G
EPI_ISL_961738, EPI_ISL_961739	SIESP CHIETI DRIVE IN LANCIANO	Istituto Zooprofilattico Sperimentale dell'Abruzzo e Molise "G. Caporale"	Lorusso A, Marcacci M, Di Domenico M, Ancora M, Curini V, Mangone I, Rinaldi A, Scialabba S, Di Pasquale A, Cammà C, Puglia I, Calistri P, Savini G
EPI_ISL_961740	SIESP CHIETI - DRIVE IN CHIETI	Istituto Zooprofilattico Sperimentale dell'Abruzzo e Molise "G. Caporale"	Lorusso A, Marcacci M, Di Domenico M, Ancora M, Curini V, Mangone I, Rinaldi A, Scialabba S, Di Pasquale A, Cammà C, Puglia I, Calistri P, Savini G
EPI_ISL_961741	SIESP CHIETI- DRIVE IN VASTO	Istituto Zooprofilattico Sperimentale dell'Abruzzo e Molise "G. Caporale"	Lorusso A, Marcacci M, Di Domenico M, Ancora M, Curini V, Mangone I, Rinaldi A, Scialabba S, Di Pasquale A, Cammà C, Puglia I, Calistri P, Savini G
EPI_ISL_961742	SIESP CHIETI - DRIVE IN CHIETI	Istituto Zooprofilattico Sperimentale dell'Abruzzo e Molise "G. Caporale"	Lorusso A, Marcacci M, Di Domenico M, Ancora M, Curini V, Mangone I, Rinaldi A, Scialabba S, Di Pasquale A, Cammà C, Puglia I, Calistri P, Savini G
EPI_ISL_961743	SIESP CHIETI DRIVE IN GISSI	Istituto Zooprofilattico Sperimentale dell'Abruzzo e Molise "G. Caporale"	Lorusso A, Marcacci M, Di Domenico M, Ancora M, Curini V, Mangone I, Rinaldi A, Scialabba S, Di Pasquale A, Cammà C, Puglia I, Calistri P, Savini G
EPI_ISL_961744	SIESP CHIETI - DRIVE IN ORTONA	Istituto Zooprofilattico Sperimentale dell'Abruzzo e Molise "G. Caporale"	Lorusso A, Marcacci M, Di Domenico M, Ancora M, Curini V, Mangone I, Rinaldi A, Scialabba S, Di Pasquale A, Cammà C, Puglia I, Calistri P, Savini G
EPI_ISL_961745	SIESP CHIETI DRIVE IN GISSI	Istituto Zooprofilattico Sperimentale dell'Abruzzo e Molise "G. Caporale"	Lorusso A, Marcacci M, Di Domenico M, Ancora M, Curini V, Mangone I, Rinaldi A, Scialabba S, Di Pasquale A, Cammà C, Puglia I, Calistri P, Savini G
EPI_ISL_961746	SIESP CHIETI - DRIVE IN CHIETI	Istituto Zooprofilattico Sperimentale dell'Abruzzo e Molise "G. Caporale"	Lorusso A, Marcacci M, Di Domenico M, Ancora M, Curini V, Mangone I, Rinaldi A, Scialabba S, Di Pasquale A, Cammà C, Puglia I, Calistri P, Savini G
EPI_ISL_961747	Dipartimento Prevenzione Avezzano-Servizio Igiene epidemiologia Sanità Pubblica	Istituto Zooprofilattico Sperimentale dell'Abruzzo e Molise "G. Caporale"	Lorusso A, Marcacci M, Di Domenico M, Ancora M, Curini V, Mangone I, Rinaldi A, Scialabba S, Di Pasquale A, Cammà C, Puglia I, Calistri P, Savini G
EPI_ISL_961748	SIESP CHIETI DRIVE IN LANCIANO	Istituto Zooprofilattico Sperimentale dell'Abruzzo e Molise "G. Caporale"	Lorusso A, Marcacci M, Di Domenico M, Ancora M, Curini V, Mangone I, Rinaldi A, Scialabba S, Di Pasquale A, Cammà C, Puglia I, Calistri P, Savini G
EPI_ISL_961749	SIESP DIPARTIMENTO DI PREVENZIONE CHIETI	Istituto Zooprofilattico Sperimentale dell'Abruzzo e Molise "G. Caporale"	Lorusso A, Marcacci M, Di Domenico M, Ancora M, Curini V, Mangone I, Rinaldi A, Scialabba S, Di Pasquale A, Cammà C, Puglia I, Calistri P, Savini G
EPI_ISL_961750	Ospedale Cvile Atri Med. Interna	Istituto Zooprofilattico Sperimentale dell'Abruzzo e Molise "G. Caporale"	Lorusso A, Marcacci M, Di Domenico M, Ancora M, Curini V, Mangone I, Rinaldi A, Scialabba S, Di Pasquale A, Cammà C, Puglia I, Calistri P, Savini G
EPI_ISL_961751	SIESP DIPARTIMENTO DI PREVENZIONE CHIETI	Istituto Zooprofilattico Sperimentale dell'Abruzzo e Molise "G. Caporale"	Lorusso A, Marcacci M, Di Domenico M, Ancora M, Curini V, Mangone I, Rinaldi A, Scialabba S, Di Pasquale A, Cammà C, Puglia I, Calistri P, Savini G
EPI_ISL_961752, EPI_ISL_961753	SIESP SULMONA	Istituto Zooprofilattico Sperimentale dell'Abruzzo e Molise "G. Caporale"	Lorusso A, Marcacci M, Di Domenico M, Ancora M, Curini V, Mangone I, Rinaldi A, Scialabba S, Di Pasquale A, Cammà C, Puglia I, Calistri P, Savini G
EPI_ISL_961754	Presidio Ospedaliero Sulmona	Istituto Zooprofilattico Sperimentale dell'Abruzzo e Molise "G. Caporale"	Lorusso A, Marcacci M, Di Domenico M, Ancora M, Curini V, Mangone I, Rinaldi A, Scialabba S, Di Pasquale A, Cammà C, Puglia I, Calistri P, Savini G
EPI_ISL_961755, EPI_ISL_961756, EPI_ISL_961757, EPI_ISL_961758	SIESP SULMONA	Istituto Zooprofilattico Sperimentale dell'Abruzzo e Molise "G. Caporale"	Lorusso A, Marcacci M, Di Domenico M, Ancora M, Curini V, Mangone I, Rinaldi A, Scialabba S, Di Pasquale A, Cammà C, Puglia I, Calistri P, Savini G
EPI_ISL_965025	S.C. Microbiologia e Virologia Laboratorio Virologia -Speciale Centro Influenza - AOU di Sassari - Viale san Pietro 43/B Palazzo Infettivologia	Laboratorio specialistico UOC Ematologia - Ospedale "San Francesco" - ATS-ASSL Nuoro	Piras Giovanna, Malune Paolo, Asproni Rosanna, Monne Maria Itria, Palmas Angelo Domenico Serra Caterina, Rimini Elena, Rubino Salvatore
EPI_ISL_965028, EPI_ISL_965031, EPI_ISL_965114	Laboratorio Biologia Molecolare Sars Cov2 - UOC Laboratorio Analisi - Servizio Medicina di Laboratorio, Ospedale "San Francesco" - ATS-ASSL Nuoro	Laboratorio specialistico UOC Ematologia - Ospedale "San Francesco" - ATS-ASSL Nuoro	Piras Giovanna, Asproni Rosanna, Malune Paolo, Fiamma Maura, Monne Maria Itria, Palmas Angelo Domenico, Lo Maglio Iana, Mameli Giuseppe
EPI_ISL_965127	INMI Lazzaro Spallanzani IRCCS	INMI Lazzaro Spallanzani IRCCS	CEM Gruber, B Bartolini, E Giombini, M Rueca, O Butera, F Messina, A Di Caro, MR Capobianchi

EPI_ISL_965128	INMI Lazzaro Spallanzani IRCCS	INMI Lazzaro Spallanzani IRCCS	M. Rueca, E Giombini, B Bartolini, O Butera, C.E.M Gruber, F Messina, A Di Caro, MR Capobianchi
EPI_ISL_965140	UOC "Microbiologia e Virologia" AOU "Policlinico Umberto I", Roma; Dipartimento di Medicina Molecolare Sapienza Università di Roma	INMI Lazzaro Spallanzani IRCCS	F Messina, C.E.M Gruber, B Bartolini, E Giombini, M Rueca, O Butera, G Antonelli, O Turriziani, MR Capobianchi, A Di Caro
EPI_ISL_965223	SIESP SULMONA	Istituto Zooprofilattico Sperimentale dell'Abruzzo e Molise "G. Caporale"	Lorusso A, Marcacci M, Di Domenico M, Ancora M, Curini V, Mangone I, Rinaldi A, Scialabba S, Di Pasquale A, Cammà C, Puglia I, Calistri P, Savini G
EPI_ISL_965224	OSPEDALE SS. ANNUNZIATA CHIETI	Istituto Zooprofilattico Sperimentale dell'Abruzzo e Molise "G. Caporale"	Lorusso A, Marcacci M, Di Domenico M, Ancora M, Curini V, Mangone I, Rinaldi A, Scialabba S, Di Pasquale A, Cammà C, Puglia I, Calistri P, Savini G
EPI_ISL_965225, EPI_ISL_965226	USCA SULMONA	Istituto Zooprofilattico Sperimentale dell'Abruzzo e Molise "G. Caporale"	Lorusso A, Marcacci M, Di Domenico M, Ancora M, Curini V, Mangone I, Rinaldi A, Scialabba S, Di Pasquale A, Cammà C, Puglia I, Calistri P, Savini G
EPI_ISL_965227, EPI_ISL_965228, EPI_ISL_965229	SIESP CHIETI - DRIVE IN CHIETI	Istituto Zooprofilattico Sperimentale dell'Abruzzo e Molise "G. Caporale"	Lorusso A, Marcacci M, Di Domenico M, Ancora M, Curini V, Mangone I, Rinaldi A, Scialabba S, Di Pasquale A, Cammà C, Puglia I, Calistri P, Savini G
EPI_ISL_965230	SIESP TERAMO DIP PREV	Istituto Zooprofilattico Sperimentale dell'Abruzzo e Molise "G. Caporale"	Lorusso A, Marcacci M, Di Domenico M, Ancora M, Curini V, Mangone I, Rinaldi A, Scialabba S, Di Pasquale A, Cammà C, Puglia I, Calistri P, Savini G
EPI_ISL_965231	Ospedale San Salvatore UOC MED INT.	Istituto Zooprofilattico Sperimentale dell'Abruzzo e Molise "G. Caporale"	Lorusso A, Marcacci M, Di Domenico M, Ancora M, Curini V, Mangone I, Rinaldi A, Scialabba S, Di Pasquale A, Cammà C, Puglia I, Calistri P, Savini G
EPI_ISL_965232, EPI_ISL_965233	SIESP CHIETI - DRIVE IN CHIETI	Istituto Zooprofilattico Sperimentale dell'Abruzzo e Molise "G. Caporale"	Lorusso A, Marcacci M, Di Domenico M, Ancora M, Curini V, Mangone I, Rinaldi A, Scialabba S, Di Pasquale A, Cammà C, Puglia I, Calistri P, Savini G
EPI_ISL_965234	SIESP SULMONA	Istituto Zooprofilattico Sperimentale dell'Abruzzo e Molise "G. Caporale"	Lorusso A, Marcacci M, Di Domenico M, Ancora M, Curini V, Mangone I, Rinaldi A, Scialabba S, Di Pasquale A, Cammà C, Puglia I, Calistri P, Savini G
EPI_ISL_965235	SIESP TERAMO DIP PREV	Istituto Zooprofilattico Sperimentale dell'Abruzzo e Molise "G. Caporale"	Lorusso A, Marcacci M, Di Domenico M, Ancora M, Curini V, Mangone I, Rinaldi A, Scialabba S, Di Pasquale A, Cammà C, Puglia I, Calistri P, Savini G
EPI_ISL_965236, EPI_ISL_965237, EPI_ISL_965238, EPI_ISL_965239	SIESP CHIETI - DRIVE IN CHIETI	Istituto Zooprofilattico Sperimentale dell'Abruzzo e Molise "G. Caporale"	Lorusso A, Marcacci M, Di Domenico M, Ancora M, Curini V, Mangone I, Rinaldi A, Scialabba S, Di Pasquale A, Cammà C, Puglia I, Calistri P, Savini G
EPI_ISL_965240	SIESP CHIETI	Istituto Zooprofilattico Sperimentale dell'Abruzzo e Molise "G. Caporale"	Lorusso A, Marcacci M, Di Domenico M, Ancora M, Curini V, Mangone I, Rinaldi A, Scialabba S, Di Pasquale A, Cammà C, Puglia I, Calistri P, Savini G
EPI_ISL_965241, EPI_ISL_965242	USCA SULMONA	Istituto Zooprofilattico Sperimentale dell'Abruzzo e Molise "G. Caporale"	Lorusso A, Marcacci M, Di Domenico M, Ancora M, Curini V, Mangone I, Rinaldi A, Scialabba S, Di Pasquale A, Cammà C, Puglia I, Calistri P, Savini G
EPI_ISL_965243, EPI_ISL_965244	SIESP CHIETI-DRIVE IN ORTONA	Istituto Zooprofilattico Sperimentale dell'Abruzzo e Molise "G. Caporale"	Lorusso A, Marcacci M, Di Domenico M, Ancora M, Curini V, Mangone I, Rinaldi A, Scialabba S, Di Pasquale A, Cammà C, Puglia I, Calistri P, Savini G
EPI_ISL_965245	OSPEDALE CIVILE TERAMO, DIV. PNEUMOLOGIA, PIA	Istituto Zooprofilattico Sperimentale dell'Abruzzo e Molise "G. Caporale"	Lorusso A, Marcacci M, Di Domenico M, Ancora M, Curini V, Mangone I, Rinaldi A, Scialabba S, Di Pasquale A, Cammà C, Puglia I, Calistri P, Savini G
EPI_ISL_965246	OSP CIV GIULIANOVA, MED INT	Istituto Zooprofilattico Sperimentale dell'Abruzzo e Molise "G. Caporale"	Lorusso A, Marcacci M, Di Domenico M, Ancora M, Curini V, Mangone I, Rinaldi A, Scialabba S, Di Pasquale A, Cammà C, Puglia I, Calistri P, Savini G
EPI_ISL_965247, EPI_ISL_965248, EPI_ISL_965249, EPI_ISL_965250, EPI_ISL_965251, EPI_ISL_965252, EPI_ISL_965253	SIESP CHIETI	Istituto Zooprofilattico Sperimentale dell'Abruzzo e Molise "G. Caporale"	Lorusso A, Marcacci M, Di Domenico M, Ancora M, Curini V, Mangone I, Rinaldi A, Scialabba S, Di Pasquale A, Cammà C, Puglia I, Calistri P, Savini G
EPI_ISL_965254, EPI_ISL_965255	SIESP CHIETI - DRIVE IN CHIETI	Istituto Zooprofilattico Sperimentale dell'Abruzzo e Molise "G. Caporale"	Lorusso A, Marcacci M, Di Domenico M, Ancora M, Curini V, Mangone I, Rinaldi A, Scialabba S, Di Pasquale A, Cammà C, Puglia I, Calistri P, Savini G
EPI_ISL_965256	SIESP CHIETI-DRIVE IN VASTO	Istituto Zooprofilattico Sperimentale dell'Abruzzo e Molise "G. Caporale"	Lorusso A, Marcacci M, Di Domenico M, Ancora M, Curini V, Mangone I, Rinaldi A, Scialabba S, Di Pasquale A, Cammà C, Puglia I, Calistri P, Savini G
EPI_ISL_965257	SIESP CHIETI-DRIVE IN GISSI	Istituto Zooprofilattico Sperimentale dell'Abruzzo e Molise "G. Caporale"	Lorusso A, Marcacci M, Di Domenico M, Ancora M, Curini V, Mangone I, Rinaldi A, Scialabba S, Di Pasquale A, Cammà C, Puglia I, Calistri P, Savini G
EPI_ISL_965258, EPI_ISL_965259	SIESP CHIETI-DRIVE IN VASTO	Istituto Zooprofilattico Sperimentale dell'Abruzzo e Molise "G. Caporale"	Lorusso A, Marcacci M, Di Domenico M, Ancora M, Curini V, Mangone I, Rinaldi A, Scialabba S, Di Pasquale A, Cammà C, Puglia I, Calistri P, Savini G
EPI_ISL_965260	SIESP CHIETI-DRIVE IN GISSI	Istituto Zooprofilattico Sperimentale dell'Abruzzo e Molise "G. Caporale"	Lorusso A, Marcacci M, Di Domenico M, Ancora M, Curini V, Mangone I, Rinaldi A, Scialabba S, Di Pasquale A, Cammà C, Puglia I, Calistri P, Savini G
EPI_ISL_965261	SIESP CHIETI	Istituto Zooprofilattico Sperimentale dell'Abruzzo e Molise "G. Caporale"	Lorusso A, Marcacci M, Di Domenico M, Ancora M, Curini V, Mangone I, Rinaldi A, Scialabba S, Di Pasquale A, Cammà C, Puglia I, Calistri P, Savini G
EPI_ISL_965262	SIESP CHIETI, DISTRETTO SAN CHIETI	Istituto Zooprofilattico Sperimentale dell'Abruzzo e Molise "G. Caporale"	Lorusso A, Marcacci M, Di Domenico M, Ancora M, Curini V, Mangone I, Rinaldi A, Scialabba S, Di Pasquale A, Cammà C, Puglia I, Calistri P, Savini G
EPI_ISL_965263	SIESP CHIETI-DRIVE IN ORTONA	Istituto Zooprofilattico Sperimentale dell'Abruzzo e Molise "G. Caporale"	Lorusso A, Marcacci M, Di Domenico M, Ancora M, Curini V, Mangone I, Rinaldi A, Scialabba S, Di Pasquale A, Cammà C, Puglia I, Calistri P, Savini G
EPI_ISL_965265	SIESP CHIETI - DRIVE IN CHIETI	Istituto Zooprofilattico Sperimentale dell'Abruzzo e Molise "G. Caporale"	Lorusso A, Marcacci M, Di Domenico M, Ancora M, Curini V, Mangone I, Rinaldi A, Scialabba S, Di Pasquale A, Cammà C, Puglia I, Calistri P, Savini G
EPI_ISL_965266	SIESP CHIETI	Istituto Zooprofilattico Sperimentale dell'Abruzzo e Molise "G. Caporale"	Lorusso A, Marcacci M, Di Domenico M, Ancora M, Curini V, Mangone I, Rinaldi A, Scialabba S, Di Pasquale A, Cammà C, Puglia I, Calistri P, Savini G
EPI_ISL_965267	SIESP CHIETI, DISTRETTON SAN CHIETI	Istituto Zooprofilattico Sperimentale dell'Abruzzo e Molise "G. Caporale"	Lorusso A, Marcacci M, Di Domenico M, Ancora M, Curini V, Mangone I, Rinaldi A, Scialabba S, Di Pasquale A, Cammà C, Puglia I, Calistri P, Savini G
EPI_ISL_965268, EPI_ISL_965269	SIESP CHIETI, DISTRETTO SAN CHIETI	Istituto Zooprofilattico Sperimentale dell'Abruzzo e Molise "G. Caporale"	Lorusso A, Marcacci M, Di Domenico M, Ancora M, Curini V, Mangone I, Rinaldi A, Scialabba S, Di Pasquale A, Cammà C, Puglia I, Calistri P, Savini G
EPI_ISL_965270	SIESP CHIETI	Istituto Zooprofilattico Sperimentale dell'Abruzzo e Molise "G. Caporale"	Lorusso A, Marcacci M, Di Domenico M, Ancora M, Curini V, Mangone I, Rinaldi A, Scialabba S, Di Pasquale A, Cammà C, Puglia I, Calistri P, Savini G
EPI_ISL_965271	SIESP CHIETI, DISTRETTO SAN CHIETI	Istituto Zooprofilattico Sperimentale dell'Abruzzo e Molise "G. Caporale"	Lorusso A, Marcacci M, Di Domenico M, Ancora M, Curini V, Mangone I, Rinaldi A, Scialabba S, Di Pasquale A, Cammà C, Puglia I, Calistri P, Savini G
EPI_ISL_965272	SIESP CHIETI	Istituto Zooprofilattico Sperimentale dell'Abruzzo e Molise "G. Caporale"	Lorusso A, Marcacci M, Di Domenico M, Ancora M, Curini V, Mangone I, Rinaldi A, Scialabba S, Di Pasquale A, Cammà C, Puglia I, Calistri P, Savini G
EPI_ISL_965273, EPI_ISL_965274, EPI_ISL_965275	SIESP CHIETI, DISTRETTO SAN CHIETI	Istituto Zooprofilattico Sperimentale dell'Abruzzo e Molise "G. Caporale"	Lorusso A, Marcacci M, Di Domenico M, Ancora M, Curini V, Mangone I, Rinaldi A, Scialabba S, Di Pasquale A, Cammà C, Puglia I, Calistri P, Savini G

EPI_ISL_965276	OSP CIV ATRI	Istituto Zooprofilattico Sperimentale dell'Abruzzo e Molise "G. Caporale"	Lorusso A, Marcacci M, Di Domenico M, Ancora M, Curini V, Mangone I, Rinaldi A, Scialabba S, Di Pasquale A, Cammà C, Puglia I, Calistri P, Savini G
EPI_ISL_965278, EPI_ISL_965279, EPI_ISL_965280, EPI_ISL_965281, EPI_ISL_965282, EPI_ISL_965283, EPI_ISL_965284, EPI_ISL_965285, EPI_ISL_965286, EPI_ISL_965287, EPI_ISL_965288, EPI_ISL_965289, EPI_ISL_965290, EPI_ISL_965291, EPI_ISL_965292, EPI_ISL_965293, EPI_ISL_965294, EPI_ISL_965295, EPI_ISL_965296, EPI_ISL_965297, EPI_ISL_965298, EPI_ISL_965299, EPI_ISL_965300			
see above	P.O.CARDARELLI	Istituto Zooprofilattico Sperimentale dell'Abruzzo e Molise "G. Caporale"	Scutellà M, Niro G, Lorusso A, Marcacci M, Di Domenico M, Ancora M, Curini V, Mangone I, Rinaldi A, Scialabba S, Di Pasquale A, Cammà C, Puglia I, Calistri P, Savini G
EPI_ISL_969131	Laboratorio Microbiologia e Virologia P.O. Cotugno A.O. dei Colli	Laboratorio Microbiologia e Virologia P.O. Cotugno A.O. dei Colli	Luigi Atripaldi, Claudia Tiberio, Anna Perfetti,
EPI_ISL_969227	Laboratorio Microbiologia e Virologia P.O. Cotugno A.O. dei Colli	Laboratorio Microbiologia e Virologia P.O. Cotugno A.O. dei Colli	Luigi Atripaldi, Claudia Tiberio, Anna Perfetti
EPI_ISL_969297	Laboratorio Microbiologia e Virologia, P.O. Cotugno, A.O. dei Colli	Laboratorio Microbiologia e Virologia, P.O. Cotugno, A.O. dei Colli	Luigi Atripaldi, Claudia Tiberio, Anna Perfetti
EPI_ISL_969884	Laboratorio Microbiologia e Virologia P.O. Cotugno A.O. dei Colli	Laboratorio Microbiologia e Virologia P.O. Cotugno A.O. dei Colli	Luigi Atripaldi,Claudia Tiberio, Anna Perfetti
EPI_ISL_970647	Laboratorio Microbiologia e Virologia P.O. Cotugno A.O. dei Colli	Laboratorio Microbiologia e Virologia P.O. Cotugno A.O. dei Colli	Luigi Atripaldi, Claudia Tiberio,Anna Perfetti
EPI_ISL_974745	Laboratorio Microbiologia e Virologia P.O. Cotugno A.O. dei Colli	Laboratorio Microbiologia e Virologia P.O. Cotugno A.O. dei Colli	Luigi Atripaldi, Claudia Tiberio, Anna Perfetti,
EPI_ISL_977495, EPI_ISL_977496, EPI_ISL_977497, EPI_ISL_977498	University of Bari Biomedical Sciences and Human Oncology	University of Bari Biomedical Sciences and Human Oncology	Chironna M., Sallustio A., Loconsole D., Accogli M.
EPI_ISL_977598	Laboratorio Microbiologia e Virologia P.O. Cotugno A.O. dei Colli	Laboratorio Microbiologia e Virologia P.O. Cotugno A.O. dei Colli	Luigi Atripaldi, Claudia Tiberio, Anna Perfetti
EPI_ISL_977601, EPI_ISL_977603	Laboratorio Microbiologia e Virologia P.O. Cotugno A.O. dei Colli	Laboratorio Microbiologia e Virologia P.O. Cotugno A.O. dei Colli	Luigi Atripaldi, Claudia Tiberio, Anna Perfetti,
EPI_ISL_977604, EPI_ISL_977605, EPI_ISL_977606, EPI_ISL_977607, EPI_ISL_977608, EPI_ISL_977609, EPI_ISL_977610, EPI_ISL_977611	SC (UCO) Igiene e Sanità Pubblica (funzione integrata con SC Microbiologia e Virologia) e Laboratory of Molecular Virology of the International Centre for Genetic Engineering and Biotechnology (ICGEB)	ARGO Laboratorio Genomica ed Epigenomica	Licastro D, Dal Monego S, Degasperi M, Marcello A, D'Agaro P, De Rosa R
EPI_ISL_977612, EPI_ISL_977613, EPI_ISL_977614, EPI_ISL_977615, EPI_ISL_977616, EPI_ISL_977617, EPI_ISL_977618, EPI_ISL_977619, EPI_ISL_977620	SC (UCO) Igiene e Sanità Pubblica (funzione integrata con SC Microbiologia e Virologia) e Laboratory of Molecular Virology of the International Centre for Genetic Engineering and Biotechnology (ICGEB)	ARGO Laboratorio Genomica ed Epigenomica	Licastro D, Dal Monego S, Degasperi M, Marcello A, D'Agaro P, Pipan C
EPI_ISL_977621	SC (UCO) Igiene e Sanità Pubblica (funzione integrata con SC Microbiologia e Virologia) e Laboratory of Molecular Virology of the International Centre for Genetic Engineering and Biotechnology (ICGEB)	ARGO Laboratorio Genomica ed Epigenomica	Licastro D, Dal Monego S, Degasperi M, Marcello A, D'Agaro P
EPI_ISL_977622, EPI_ISL_977623, EPI_ISL_977624, EPI_ISL_977625, EPI_ISL_977626, EPI_ISL_977627, EPI_ISL_977628, EPI_ISL_977629, EPI_ISL_977630, EPI_ISL_977631, EPI_ISL_977632, EPI_ISL_977633, EPI_ISL_977634, EPI_ISL_977635, EPI_ISL_977636, EPI_ISL_977637, EPI_ISL_977638, EPI_ISL_977639, EPI_ISL_977640			
see above	SC (UCO) Igiene e Sanità Pubblica (funzione integrata con SC Microbiologia e Virologia) e Laboratory of Molecular Virology of the International Centre for Genetic Engineering and Biotechnology (ICGEB)	ARGO Laboratorio Genomica ed Epigenomica	Licastro D, Dal Monego S, Degasperi M, Marcello A, Segat L, Piscianz E, D'Agaro P
EPI_ISL_977641, EPI_ISL_977642, EPI_ISL_977643, EPI_ISL_977644, EPI_ISL_977645, EPI_ISL_977646, EPI_ISL_977647, EPI_ISL_977648, EPI_ISL_977649, EPI_ISL_977650	SC (UCO) Igiene e Sanità Pubblica (funzione integrata con SC Microbiologia e Virologia) e Laboratory of Molecular Virology of the International Centre for Genetic Engineering and Biotechnology (ICGEB)	ARGO Laboratorio Genomica ed Epigenomica	Licastro D, Dal Monego S, Degasperi M, Marcello A, D'Agaro P, Lombardo F
EPI_ISL_977651	Laboratorio Microbiologia e Virologia P.O. Cotugno A.O. dei Colli	Laboratorio Microbiologia e Virologia P.O. Cotugno A.O. dei Colli	Luigi Atripaldi, Claudia Tiberio, Anna Perfetti,
EPI_ISL_983096, EPI_ISL_983097, EPI_ISL_983098	Microbiology and Virology Unit, Florence Careggi University Hospital	Microbiology and Virology Unit, Florence Careggi University Hospital	Vincenzo Di Pilato, Marco Coppi, Fabio Morecchiato, Noemi Aiezza, Ilaria Baccani, Alberto Antonelli, Emanuele Gori, Gian Maria Rossolini
EPI_ISL_983325	INMI Lazzaro Spallanzani IRCCS	INMI Lazzaro Spallanzani IRCCS	M Rueca, O Butera, F Messina, CEM Gruber, B Bartolini, E Giombini, A Di Caro, MR Capobianchi
EPI_ISL_983326	Laboratorio di Genetica Medica Ospedale Belcolle	INMI Lazzaro Spallanzani IRCCS	F Messina, C.E.M Gruber, B Bartolini, E Giombini, M Rueca, O Butera, F Natoni, G Pessina, A Di Caro, MR Capobianchi
EPI_ISL_983327	Dipartimento di Prevenzione ASL Roma 4	INMI Lazzaro Spallanzani IRCCS	O Butera, F Messina, CEM Gruber, B Bartolini, E Giombini, M Rueca, S Ursino, MR Capobianchi, A Di Caro
EPI_ISL_983328	Azienda Ospedaliera San Camillo Forlanini	INMI Lazzaro Spallanzani IRCCS	E Giombini, M. Rueca, B Bartolini, O Butera, C.E.M Gruber, F Messina, G Parisi, ML Guarino, A Di Caro, MR Capobianchi
EPI_ISL_983329	Istituto Zooprofilattico Sperimentale Lazio e Toscana "M. Aleandri"	INMI Lazzaro Spallanzani IRCCS	CEM Gruber, B Bartolini, E Giombini, M Rueca, O Butera, F Messina, MT Scicluna, G Manna, A Cersini, A Di Caro, MR Capobianchi
EPI_ISL_984995, EPI_ISL_984996	U.O. Igiene, Ospedale Policlinico San Martino	U.O. Igiene, Ospedale Policlinico San Martino	Bruzzone Bianca, Caligiuri Patrizia, De Pace Vanessa, Domnich Alexander, Orsi Andrea, Ricucci Valentina, Icardi Giancarlo
EPI_ISL_984997, EPI_ISL_984998, EPI_ISL_984999	S.C. Laboratorio Analisi, ASL 3 Liguria	U.O. Igiene, Ospedale Policlinico San Martino	Bruzzone Bianca, Caligiuri Patrizia, De Pace Vanessa, Domnich Alexander, Orsi Andrea, Ricucci Valentina, Spitaleri Antonino, Icardi Giancarlo
EPI_ISL_985000	U.O. Igiene, Ospedale Policlinico San Martino	U.O. Igiene, Ospedale Policlinico San Martino	Bruzzone Bianca, Caligiuri Patrizia, De Pace Vanessa, Domnich Alexander, Orsi Andrea, Ricucci Valentina, Spitaleri Antonino, Icardi Giancarlo
EPI_ISL_985001, EPI_ISL_985002, EPI_ISL_985003, EPI_ISL_985004, EPI_ISL_985005, EPI_ISL_985006, EPI_ISL_985007, EPI_ISL_985008, EPI_ISL_985009, EPI_ISL_985010, EPI_ISL_985011, EPI_ISL_985012, EPI_ISL_985013, EPI_ISL_985014, EPI_ISL_985015, EPI_ISL_985016			
see above	S.C. Laboratorio Analisi, ASL 3 Liguria	U.O. Igiene, Ospedale Policlinico San Martino	Bruzzone Bianca, Caligiuri Patrizia, De Pace Vanessa, Domnich Alexander, Orsi Andrea, Ricucci Valentina, Spitaleri Antonino, Icardi Giancarlo
EPI_ISL_985017	S.C. Patologia Clinica, Ospedale Sant'Andrea, ASL 5	U.O. Igiene, Ospedale Policlinico San Martino	Bruzzone Bianca, Battolla Enrico, Caligiuri Patrizia, De Pace Vanessa, Domnich Alexander, Orsi Andrea, Ricucci Valentina, Icardi Giancarlo
EPI_ISL_985018, EPI_ISL_985019, EPI_ISL_985020, EPI_ISL_985021, EPI_ISL_985022, EPI_ISL_985023, EPI_ISL_985024	S.S.D. Microbiologia, Stabilimento ospedaliero di Sanremo, ASL 1 Liguria	U.O. Igiene, Ospedale Policlinico San Martino	Bruzzone Bianca, Caligiuri Patrizia, De Pace Vanessa, Domnich Alexander, Dusi Pier Andrea, Orsi Andrea, Ricucci Valentina, Icardi Giancarlo
EPI_ISL_985025, EPI_ISL_985026	S.S.D. Microbiologia, Ospedale Santa Corona di Pietra Ligure, ASL 2 Liguria	U.O. Igiene, Ospedale Policlinico San Martino	Bruzzone Bianca, Caligiuri Patrizia, De Pace Vanessa, Domnich Alexander, Orsi Andrea, Ricucci Valentina, Valle Caterina, Icardi Giancarlo

EPI_ISL_985027, EPI_ISL_985028, EPI_ISL_985029, EPI_ISL_985030, EPI_ISL_985031	Laboratorio di Patologia Clinica, Ospedale San Paolo in Valloria, ASL 2 Liguria	U.O. Igiene, Ospedale Policlinico San Martino	Bruzzone Bianca, Caligiuri Patrizia, De Pace Vanessa, Domnich Alexander, Lillo Flavia, Orsi Andrea, Ricucci Valentina, Icardi Giancarlo
EPI_ISL_985032	U.O. Igiene, Ospedale Policlinico San Martino	U.O. Igiene, Ospedale Policlinico San Martino	Bruzzone Bianca, Caligiuri Patrizia, De Pace Vanessa, Domnich Alexander, Orsi Andrea, Ricucci Valentina, Icardi Giancarlo
